# Series of advanced backcross populations uncovers diversity and complexity in genetic control of grain length and grain width in Asian cultivated rice

**DOI:** 10.1270/jsbbs.25071

**Published:** 2026-06-11

**Authors:** Kazufumi Nagata, Tsuyu Ando, Tatsumi Mizubayashi, Ayahiko Shomura, Noriyuki Kitazawa, Yoshiyuki Mukai, Utako Yamanouchi, Shuichi Fukuoka

**Affiliations:** 1 National Agriculture and Food Research Organization (NARO) Institute of Crop Science, 2-1-2 Kannondai, Tsukuba, Ibaraki 305-8518, Japan

**Keywords:** quantitative trait locus (QTL), grain length, grain width, genetic diversity, *Oryza sativa* L.

## Abstract

The use of genetic variations in Asian cultivated rice is essential for crop improvement. Advanced technologies and accumulated genome information support this effort to ensure sustainable production under changing environmental conditions. Grain length and grain width are important agronomic traits in rice. Extensive studies have revealed their complex genetic control. To further clarify the genetic basis of grain length and grain width in rice, we constructed a comprehensive catalog of QTLs for these traits based on QTL analysis of advanced backcross populations from crosses between a *japonica* rice cultivar ‘Koshihikari’ and 12 diverse donor cultivars. It comprised 469 QTLs (average 39.1 QTLs per donor) and provided estimates of allelic effects within a uniform *japonica* genetic background. To validate and delimit QTLs, we used sub-CSSLs—a set of plant materials with one or no segregating regions from these donors in a homogeneous genetic background. New QTLs were validated by genetic mapping in three chromosomal regions previously unreported in other mapping populations. Our results underscore the diversity and complexity of genetic control of grain length and grain width in Asian cultivated rice, and these insights will facilitate more precise genetic improvement of rice grain traits.

## Introduction

Asian cultivated rice (*Oryza sativa* L.) exhibits remarkable genetic diversity, accumulated throughout its history of domestication and cultivation (Long *et al.* 2024, Vaughan *et al.* 2008). This genetic diversity is crucial for exploring and characterizing beneficial agronomic traits to improve rice for future food security (Leung *et al.* 2015, Nourollah 2016). The accumulation of large-scale varietal genome data combined with next-generation sequencing (NGS)-based approaches such as genome-wide association studies (GWAS) and quantitative trait locus sequencing (QTL-seq) has greatly facilitated the discovery of advantageous alleles (Han and Huang 2013, Ma *et al.* 2025, Takagi *et al.* 2013, Wang *et al.* 2018, Yang *et al.* 2025, Yano *et al.* 2016). These technological advancements are expected to support the prediction of agronomic performance and the selection of genotypes to meet future demands (Bartholomé *et al.* 2022, Yang *et al.* 2024).

Agronomic traits in rice are regulated by multiple QTLs with varying effect sizes (Wang *et al.* 2020, Yamamoto *et al.* 2009, Yano and Sasaki 1997). To date, 904 gene loci have been curated as agronomically important in the Rice Annotation Project Database (RAP-DB) (Kawahara *et al.* 2013, Sakai *et al.* 2013). Genome-wide DNA variation data from 685 cultivars are also available through the TASUKE+ platform (Kumagai *et al.* 2019). However, the number of genes needed to accurately predict complex traits likely exceeds those currently identified. Genomic prediction studies show that increasing the number of genetic markers to several thousand improves prediction accuracy (Bartholomé *et al.* 2022, e Sousa *et al.* 2019, Iwata *et al.* 2015). To address this challenge, expanding experimental datasets that capture allelic variation in causal genes is essential. Such data, validated through plant materials, will enhance predictive models for rice breeding.

The use of plant materials for genetic mapping has greatly advanced our understanding of the genetic control of agronomic traits in rice, including the identification of causal genes (Fukuoka *et al.* 2010, Lu *et al.* 2015). Sophisticated populations such as nested association mapping and multi-parent advanced generation inter-cross (MAGIC) populations enable high-resolution allele detection from multiple parental cultivars owing to short linkage disequilibrium (Bandillo *et al.* 2013, Fragoso *et al.* 2017, Nawade *et al.* 2024, Ogawa *et al.* 2018). MAGIC populations broaden trait variation through diverse allelic combinations but may introduce noise due to their heterogeneous genetic backgrounds, limiting their utility for analyzing loci with small effects. In contrast, advanced backcross populations such as chromosome segment substitution lines (CSSLs), which contain one to a few donor segments in a recurrent genetic background, are well suited to detecting minor-effect genes (Yamamoto *et al.* 2009). CSSLs with a *japonica* background have been developed using donors representing the genetic diversity of Asian cultivated rice (Nagata *et al.* 2023). Selecting plant materials appropriate to research objectives can optimize genetic analysis outcomes.

Grain shape is a key agronomic trait closely linked to grain productivity and varietal discrimination owing to its high phenotypic diversity. It is a complex trait governed by multiple genes, with over 200 QTLs identified and more than 90 genes cloned or molecularly characterized (Fang *et al.* 2025, Gao *et al.* 2021, Huang *et al.* 2013, Jiang *et al.* 2024, Li *et al.* 2019, 2024a, 2024b, Liu *et al.* 2025, Luo *et al.* 2025, Ma *et al.* 2025, Tang *et al.* 2023, Xin *et al.* 2024, Xuedan *et al.* 2023, Yang *et al.* 2025, Zhang *et al.* 2024, Zheng *et al.* 2024). RapMap, which uses mapping populations from cultivars with subtle phenotypic differences, identified novel grain shape alleles at *GS3* and *GL1* (Zhang *et al.* 2021). Advanced backcross populations covering the donor genome detected 65 QTLs for grain length (GL) and grain width (GW) in a *japonica* × *indica* cross (Nagata *et al.* 2015), while multiple linear regression analyses of 12 CSSL sets revealed over 400 putative QTLs (Nagata *et al.* 2023). These findings highlight the genetic complexity of GL and GW and address the need for systematic research to clarify QTL distribution and allelic diversity, which are essential for precise genetic control of GL and GW in rice breeding programs.

To advance our understanding of the genetic control of GL and GW in rice, we developed a catalog of QTLs for GL and GW based on analyses of BC_3–5_F_2_ advanced backcross populations derived from crosses between *japonica* ‘Koshihikari’ and 12 genetically diverse donor cultivars. To validate and delimit newly identified QTLs, we selected sub-CSSLs—plant materials containing one or no segregating donor regions within a homogeneous genetic background. Using these sub-CSSLs and their progeny lines, we successfully validated and precisely delimited three QTLs located in chromosomal regions where GL and GW QTLs have not been previously reported in other cross combinations.

## Materials and Methods

### Plant materials

We sowed seeds of 398 backcrossed populations (BC_3–5_F_2_) derived from crosses between the recurrent parent ‘Koshihikari’ and each of 12 donors (‘LAC23’, ‘Owarihatamochi’, ‘Bei Khe’, ‘Tupa 121-3’, ‘Khao Nam Jen’, ‘Khau Mac Kho’, ‘Deng Pao Zhai’, ‘Naba’, ‘Bleiyo’, ‘Qiu Zhao Zong’, ‘Muha’, ‘Basilanon’), 10 of which were selected from the World Rice Core Collection (Kojima *et al.* 2005) to represent the genetic diversity of Asian cultivated rice (Fig. 1). These populations derived from individual donors, each comprising 1272 to 1668 plants, provided genome-wide coverage of the respective donor genomes. The seeds were raised in previous studies (Abe *et al.* 2013, Mizuno *et al.* 2018, Nagata *et al.* 2023). We selected BC_3–5_F_2_ plants with fewer and smaller segregating chromosomal regions than the corresponding chromosomal regions in the respective CSSLs. BC_3–5_F_3_ seeds were used as research materials designated as sub-CSSLs to validate and delimit the QTLs of interest, which also included BC_4_F_3_ seeds derived from ‘IR64’ as a donor parent (Nagata *et al.* 2015) (Fig. 1). We selected 14 sub-CSSLs to validate 3 new QTLs. These QTLs were chosen based on the following criteria: (1) donor alleles that significantly increased trait values in the recurrent genetic background, and (2) QTL alleles present in multiple cultivars, making them potentially useful for diverse breeding programs. The selected lines were derived from crosses between ‘Koshihikari’ and ‘IR64’, ‘Deng Pao Zhai’, or ‘Bei Khe’. The first QTL, *qGW1-1*, is located at ~0.3 Mb on chr. 1, where the ‘IR64’ allele increases GW (Nagata *et al.* 2015). The second QTL was located at ~3.1 Mb on chr. 7, where the ‘Deng Pao Zhai’ allele increased GW. The third QTL was found at ~5.0 Mb on chr. 12, where the ‘Bei Khe’ allele increased GL. We chose these QTLs because no genes related to GL and GW have previously been reported in these regions. To further delimit *qGW1-1*, we used 6 BC_4_F_5_ progeny lines derived from sub-CSSLs carrying the ‘IR64’ donor segment with recombination events near the *qGW1-1* region.

We used 44 or 45 plants in the BC_3–5_F_2_generation (population‑dependent), 45 or 88 plants in BC_4_F_3_, and 16 plants in BC_4_F_5_ for genotyping and trait measurements. All plants were grown in an experimental field at NARO, Tsukuba, Japan (36.03°N, 140.11°E). Basal fertilizer was applied at 56 kg/ha N, 56 kg/ha P, and 56 kg/ha K. Month-old seedlings were transplanted in mid May at one seedling per hill in plots with a double row for each line, at 18 cm between plants and 36 cm between rows. Rice grains were harvested in September or October and were air-dried before analysis.

### Measurement of GL and GW

We obtained data for GL and GW as described previously (Nagata *et al.* 2023). We scanned ~200 grains per plant at 600 dpi on an image scanner and measured the GL and GW in SmartGrain grain shape analysis software (Tanabata *et al.* 2012).

### DNA extraction and marker analysis

Fresh leaves were harvested from field-grown plants, and total DNA was extracted from 1- to 3-cm sections by crushing in 30 μL 0.5 M NaOH and diluting in 120 μL of 1 M Tris·HCl (pH 8.0). We used 932 simple sequence repeat (SSR) markers and a single-nucleotide polymorphism (SNP) marker for *GS3* (IDGS3_001_3) to detect QTLs in the BC_3–5_F_2_ populations. The DNA samples of BC_3–5_F_2_ plants were obtained from previous studies (Abe *et al.* 2013, Mizuno *et al.* 2018, Nagata *et al.* 2015, 2023), and genotypes were determined at mean intervals of 2.7 Mb per marker (Supplemental Table 1). We added 3 SSR markers, 3 insertion–deletion markers, and 22 SNP markers to the region around the QTLs on chr. 1 to determine the position in ‘IR64’, on chr. 7 in ‘Deng Pao Zhai’, and on chr. 12 in ‘Bei Khe’ (Supplemental Tables 1, 2).

### Statistical analysis

We constructed a genetic map in MAPMAKER/EXP v. 3.0 software (Lander *et al.* 1987). QTL analysis for GL and GW was performed in QTL Cartographer v. 2.5 software (Basten *et al.* 2005), and the threshold was obtained by using 1000 permutations at α = 0.05 for each population. Two-tailed *t*-tests were conducted to evaluate differences between the genotypes in each BC_4_F_5_ population.

Cluster analysis was performed using Ward’s method, based on the Euclidean distance using additive effect values, to examine the relationships between cultivars, taking into account the magnitude of these values, in the R package “dendextend” (Galili 2015, R Core Team 2023). The values were standardized by subtracting the mean from each input value and dividing the difference by the standard deviation. It was also performed using binary data indicating the presence or absence of QTL alleles for GL and GW that differ from those of the recurrent cultivar. QTL alleles within a chromosome interval whose additive effect values were in opposite directions were treated as alleles at independent loci. An example was when QTLs with opposite effects were detected in populations segregating only for the upstream or downstream portion of the same chromosomal interval. For the respective QTL alleles in ‘Koshihikari’, a value of 0 was used.

## Results

### Variation of GL and GW in BC_3–5_F_2_ populations

As expected, the means of both GL and GW of 398 backcrossed populations were close to those of the recurrent parent (Table 1, Supplemental Fig. 1). However, the range of values within the backcrossed populations varied widely as a proportion of the parental values, from 63% to 483% in GL and 64% to 464% in GW (Table 1).

### QTLs for GL in BC_3–5_F_2_ populations

QTL analysis for GL in 398 backcrossed populations identified 232 QTLs, with 13 to 27 QTLs per donor, in 43 intervals (Table 2, Supplemental Tables 3–14). The LOD threshold (α = 0.05) for GL ranged from a minimum of 1.5 to a maximum of 8.5, with a mean of 2.4 (SD = 0.7). Notably, five populations segregating chromosomal regions around *GS3*—two in KNK, two in LAK, and one in MUK—showed thresholds more than twice the overall average. The average absolute value of the additive effect was 0.094 (Table 2). Among them, 7 alleles had absolute additive effects of >0.20 mm (3.0% of the total) and 25 had effects of >0.15 mm (10.8%) (Table 2, Fig. 2A). *Japonica* donors had more alleles with effects of >0.2 mm (0.8 per donor) than *indica* donors (0.5), despite *indica* contributing more alleles overall (Table 2). These alleles were found in QTLs on chr. 3, including the *GS3* region (Table 2, Supplemental Tables 6, 8, 9, 11, 13, 14). QTLs with effects of >0.15 mm were located in 15 regions across 7 chromosomes where genes for GL and GW have been reported, and were found in populations from 9 donors (Table 2). QTLs were detected in both *indica* and *japonica* donors in 33 of the 43 intervals (Table 2). The remaining intervals had QTLs only from *indica* donors (Table 2). Estimated GL, calculated from cumulative additive effects across the 12 donors, correlated with measured length (*R*^2^ = 0.31).

### QTLs for GW in BC_3–5_F_2_ populations

QTL analysis for GW in 398 backcrossed populations identified 237 QTLs, with 15 to 26 QTLs per donor, assigned to 49 intervals (Table 3, Supplemental Tables 3–14). The LOD threshold (α = 0.05) for GW ranged from a minimum of 1.5 to a maximum of 4.3, with a mean of 2.3 (SD = 0.4). The average absolute value of the additive effect was 0.048 (Table 3). Among these, 9 alleles had absolute additive effects of >0.15 mm (3.9% of the total) and 24 had effects of >0.075 mm (10.4%) (Table 3, Fig. 2B). *Indica* donors contributed more alleles than *japonica* donors, both in total and in those with effects of >0.15 mm (Table 3). These alleles were found in the 0.45–5.88-Mb region on chr. 5, where *GS5* and *qGW5* are located, in populations from 8 *indica* and 1 *japonica* donors (Table 3, Supplemental Tables 3–11). QTLs with effects of >0.075 mm were detected in 13 regions across 7 chromosomes, all but 1 of which correspond to previously reported genes related to GL and GW, in populations from 11 donors (Table 3). QTLs were detected in both *indica* and *japonica* donors in 33 of the 49 intervals (Table 3). In the remaining intervals, QTLs were found only in *indica* donors in 13 intervals and only in *japonica* donors in 3 intervals (Table 3). Estimated GW, calculated from cumulative additive effects across the 12 donor populations, had a strong correlation with measured width (*R*^2^ = 0.72).

### Genetic relationship among donors depicted by GL and GW QTLs

In total, we identified 469 QTLs related to GL and GW (232 for GL and 237 for GW) across 12 populations. Of these, 318 (68%) overlapped with regions of cloned genes when overlap was defined as the region within 3 Mb (±1.5 Mb) around each QTL’s LOD peak. Based on these QTLs, dendrograms constructed using additive effect values for GL or GW QTLs divided donors and recurrent cultivars into two groups based on the presence or absence of QTLs with the largest effects—specifically, QTLs for GL in the *GS3* region on chr. 3 and for GW in the *GS5/qGW5* region on chr. 5 (Fig. 3A, 3B). To assess the similarity in the presence or absence of QTL alleles, we constructed dendrograms using binary data of QTLs (Fig. 3C, 3D). The dendrogram for GL divided the cultivars into 2 groups: one consisting solely of *indica* cultivars, and the other consisting of *japonica* cultivars and the remaining *indica* cultivars (Fig. 3C). Similarly, the dendrogram constructed using binary data of QTLs for GW divided donors and recurrent cultivars into 2 groups corresponding to *indica* and *japonica* cultivars, although 2 *indica* cultivars (‘Tupa 121-3’ and ‘Muha’) were grouped with *japonica* (Fig. 3D).

### Selection of sub-CSSLs as research materials for dissecting agricultural traits in rice

From the BC_3–5_F_2_ populations, we selected ~28% of plants with reduced heterozygosity (average 3.0%) compared to the original population (6.2%). These plants, with segregating regions mostly confined to a single chromosome, were used to establish sub-CSSLs for subsequent QTL validation (Table 4).

The number of lines representing each chromosome was generally proportional to the length of the chromosome (*R*^2^ = 0.84, Table 4). However, the number of lines selected for each chromosome varied by donor, with certain chromosomes having more (e.g., IRK_chr. 1, OWK_chr. 4) or fewer (e.g., BAK_chr. 4, KMK_chr. 9) lines than the average (Table 4). These differences were associated with factors such as the number of recombinants obtained or the absence of seeds due to sterility.

### Validation of GL and GW QTLs by using sub-CSSLs

We analyzed 5 sub-CSSLs containing ‘IR64’ chromosome segments around *qGW1-1* with partial overlap (Fig. 4A). QTL analysis confirmed the presence or absence of *qGW1-1* in each sub-CSSL and localized the QTL within a 1.63-Mb interval between marker loci RM3148 and RM6324 (Fig. 4A). QTLs for GL were identified on both sides of *qGW1-1*, with additive effects in opposite directions (Supplemental Fig. 2A).

We analyzed 5 sub-CSSLs containing ‘Deng Pao Zhai’ chromosome segments around 3.14 Mb on chr. 7. Three sub-CSSLs detected a QTL for GW, designated as *qGW7-2* (Fig. 4B). The consensus region of *qGW7-2* was located within a 491-kb interval between marker loci KA1894 and RM5711 (Fig. 4B). Consistent with the analysis in BC_3–5_F_2_, a QTL for GL was identified distal to *qGW7-2* (Supplemental Fig. 2B).

We analyzed 4 sub-CSSLs containing ‘Bei Khe’ chromosome segments around 5.04 Mb on chr. 12. Three sub-CSSLs detected a QTL for GL, designated as *qGL12-2* (Fig. 4C). The consensus region of the QTL was located within a 3.99-Mb interval between marker loci IDR1566 and KA3544 (Fig. 4C). Another QTL for GL was detected proximal to *qGL12-2* (Supplemental Fig. 2C).

### Delimitation of *qGW1-1* by using progeny lines of sub-CSSLs

To further delimit *qGW1-1*, we used 6 progeny lines (BC_4_F_5_) where recombination occurred within the target chromosomal region of the QTL. We compared GWs between homozygous recombinant plants and control non-recombinant plants within the lines to test whether the alleles differed. The analysis delimited *qGW1-1* to an interval between marker loci FA6836 and RM5423, corresponding to 477-kb in the ‘Nipponbare’ IRGSP-1.0 genome (Fig. 5).

## Discussion

We developed a comprehensive catalog of QTLs for GL and width in Asian cultivated rice and revealing genetic variations underlying these traits among diverse donor cultivars. The use of sub-CSSLs enabled efficient validation and delimitation of QTLs, including those in previously unreported chromosomal regions.

These results broaden our understanding of the genetic control of GL and width in rice, which will support more precise strategies for improving rice grain traits. More QTLs were derived from *indica* donors than from *japonica* donors, reflecting a greater genetic difference from the recurrent parent (Tables 2, 3). Major-effect QTLs such as *GS3* for GL and *qGW5* for grain width (Shomura *et al.* 2008, Takano-Kai *et al.* 2009) were key factors in distinguishing varietal groups when additive effects were considered (Fig. 3A, 3B), while the overall architecture may reflect genome-wide divergence (Fig. 3C, 3D). Such observations suggest that genetic variation in GL and width is shaped by minor-effect QTL alleles accumulated through historical hybridization and differentiation events (Civáň *et al.* 2015, Santos *et al.* 2019, Vaughan *et al.* 2008), together with spontaneous mutations, rather than by strong selection on individual alleles.

The coincidence of QTLs for GL and width is well documented (Huang *et al.* 2013, Ogawa *et al.* 2018), and many cloned genes affect both traits, either in the same or in opposite directions (Xuedan *et al.* 2023). In our study, progeny testing using sub-CSSLs indicated that independent loci within the same chromosomal region may regulate these traits (Fig. 4, Supplemental Fig. 2). However, limited resolution prevented us from determining whether the observed variation among donors at QTLs within a chromosomal interval (Table 2, 3) reflected allelic differences at a single locus (Sun *et al.* 2022, Wang *et al.* 2015a) or tightly linked multiple QTLs and copy number variations (Wang *et al.* 2015b). Approximately 68% (318 out of 469) of the detected QTLs overlapped with regions harboring cloned genes, yet the presence of multiple cloned genes within a single chromosomal region and the extensive sequence variation among donor cultivars complicate the prediction of genotype–phenotype relationships. These considerations suggest that the number of QTLs may be underestimated and highlight the need for higher-resolution mapping and detailed characterization of causal variants to improve trait prediction and support crop improvement.

Even in highly homogenous backcrossed populations, unidentified small introgressions from donors can introduce background noise in QTL analysis. In some populations, we were unable to detect QTLs, despite phenotypic variation comparable to that in populations where QTLs were detected. Incorporating genotype information obtained through re-sequencing can help mitigate this issue. Another challenge is gene interaction, such as the combined effect of *GW7* and *GW8* (Yang *et al.* 2023), which cannot be estimated in a recurrent genetic background. The discrepancy between the estimated values based on cumulative additive effects of QTLs and the actual measured values highlights the need for complementary approaches or genetic materials, such as nested association mapping or MAGIC populations, to achieve more robust trait prediction.

Advances in resequencing and large-scale phenotyping have improved variant detection and enable sophisticated workflows (Han and Huang 2013, Leung *et al.* 2015, Wang *et al.* 2018, Yang *et al.* 2025, Zhang *et al.* 2021). However, predicting the effects of DNA variations, including those in non-coding regions, across diverse cultivars remains challenging due to their abundance and complexity (Hou *et al.* 2019, Yocca and Edger 2022, Wang *et al.* 2020). Pan-genomics approaches have uncovered previously undetected variations in reference genomes, including in stress-response genes, highlighting the potential of diverse genetic resources to explain missing heritability (Guo *et al.* 2025, Qin *et al.* 2021, Zhao *et al.* 2018) and strengthen prediction models (Zhou *et al.* 2022). Integrating genomic approaches and genetic studies with systematic use of advanced mapping populations such as sub-CSSLs can accelerate the identification of key variations, which is critical for improving prediction models (Zhang *et al.* 2014) and developing resilient crop cultivars for sustainable production under changing environmental conditions.

## Author Contribution Statement

SF conceived and designed the experiments. KN, TA and the other authors determined the genotypes. KN and SF developed the plant materials and phenotyped agronomic traits. KN, TA and SF analyzed the data. KN and SF wrote the paper. All authors have reviewed drafts of the paper and approved the final version.

## Supplementary Material

Supplemental Figures

Supplemental Tables

## Figures and Tables

**Fig. 1.**
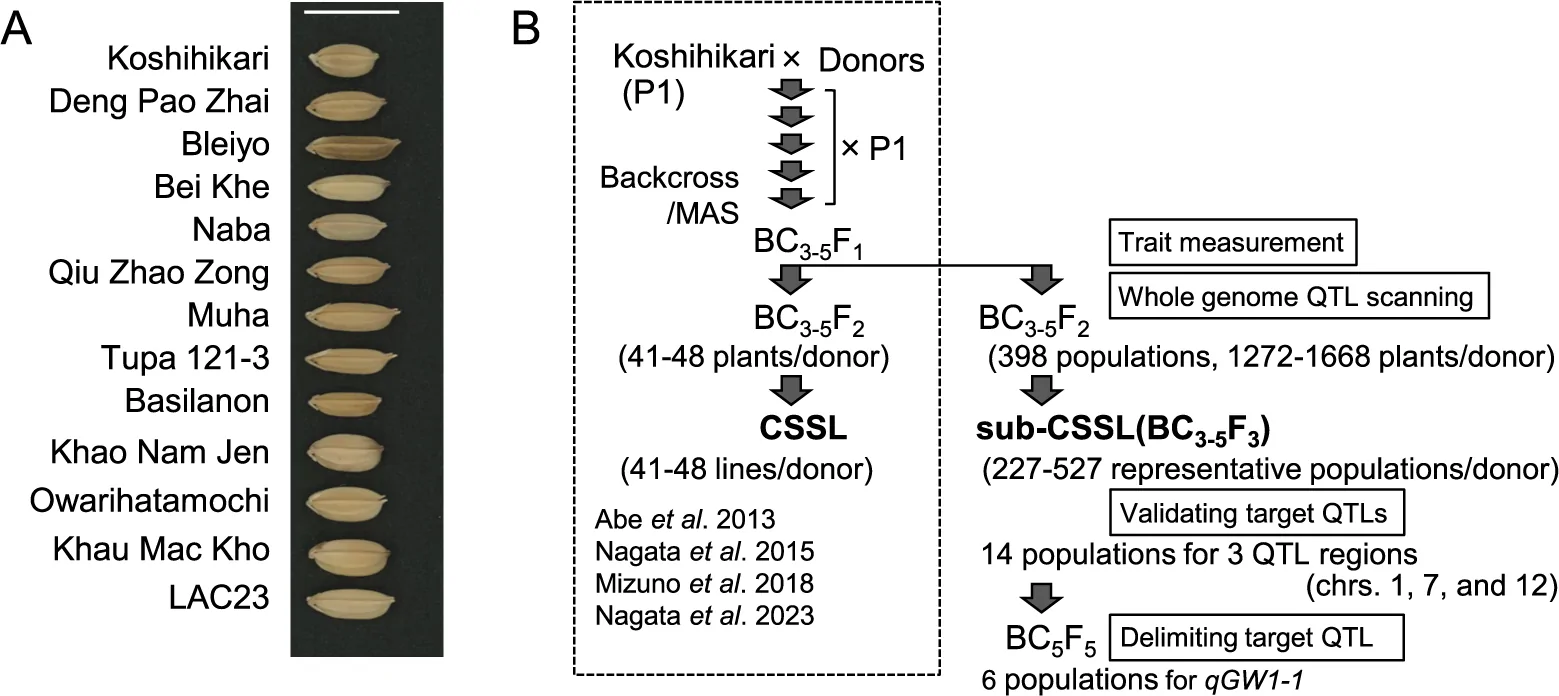
Plant materials used in this study. (A) Seeds of recurrent and donor parents, named on the left. White bar, 10 mm. (B) Schematic illustration of the development of the plant materials used. Backcrossed populations were derived from indicated studies. CSSL, chromosome segment substitution line; MAS, marker-assisted selection; QTL, quantitative trait locus.

**Fig. 2.**
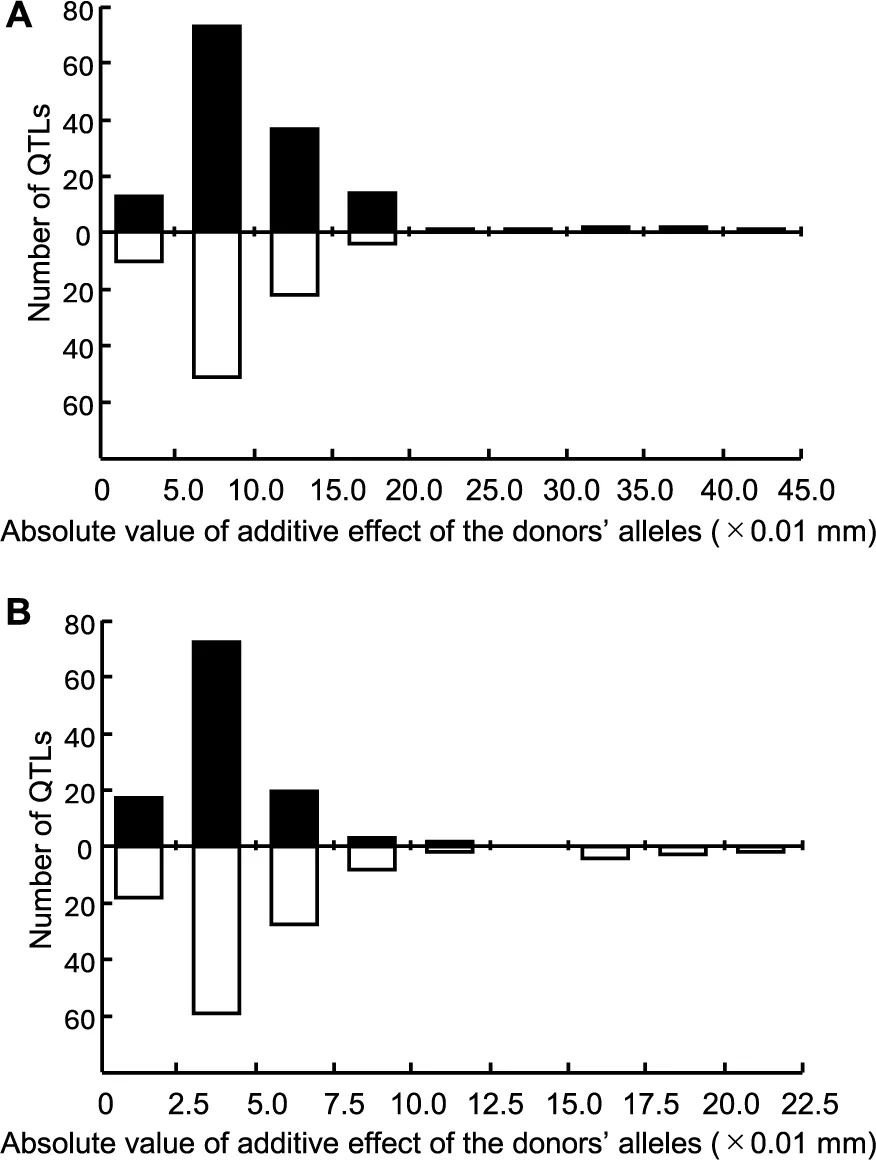
Numbers of QTLs detected in BC_3–5_F_2_ populations and their additive effects. A total of 398 BC_3–5_F_2_ progeny were derived from crosses between ‘Koshihikari’ and 12 donor parents. Bars indicate ‘Koshihikari’ alleles that ■ increased or □ decreased (A) GL and (B) GW relative to donor alleles.

**Fig. 3.**
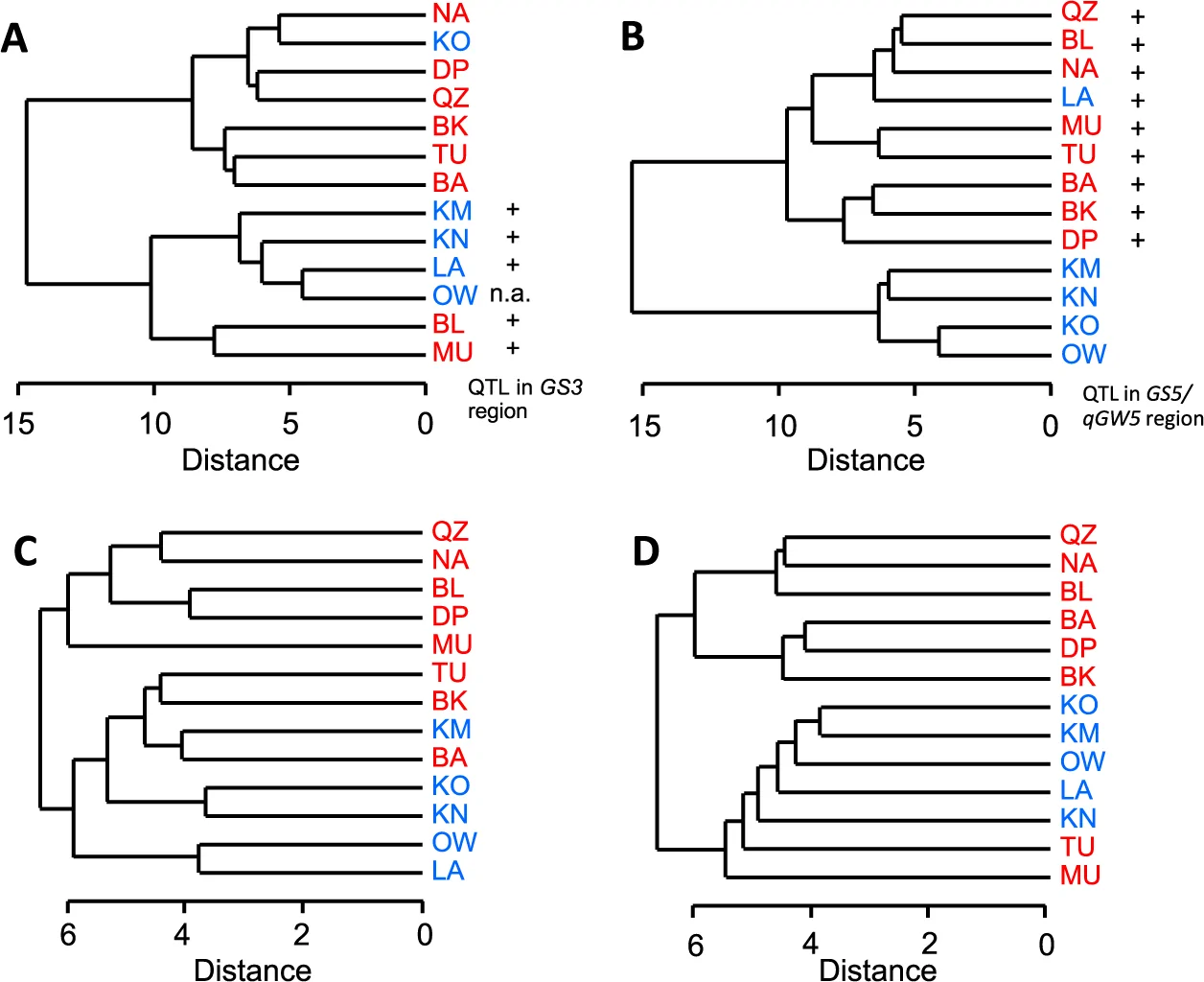
Relationships among rice cultivars classified by GL and GW QTLs. Dendrograms were constructed using (A, B) additive effect values or (C, D) binary data of QTLs for (A, C) GL and (B, D) GW. + Cultivars carrying alleles that alter ‘Koshihikari’ phenotypes at *GS3* or *GS5/qGW5*. Cultivar codes are shown on the right (see Table 1); red, *indica*; blue, *japonica*.

**Fig. 4.**
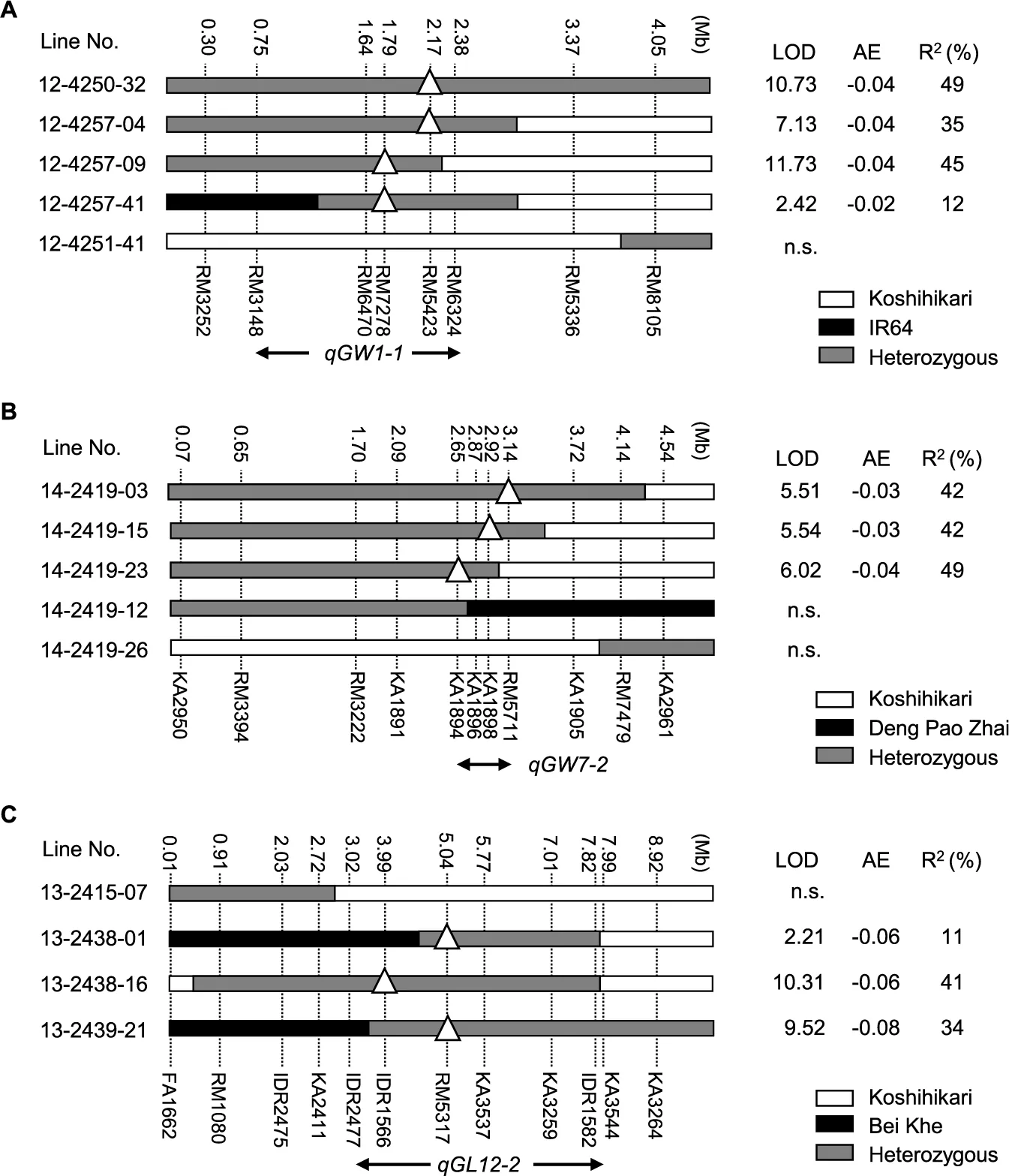
Validation of QTLs for GL and GW detected in BC_3–5_F_2_ populations by use of sub-CSSLs. Graphical genotypes of sub-CSSLs and the positions of QTLs detected in each sub-CSSL are shown. (A) QTLs for GW detected around 0.30–0.75 Mb on chr. 1 in the population with ‘IR64’ as the donor (Nagata *et al.* 2015). (B) QTLs for GW detected in the region around 3.14 Mb on chr. 7 in the population with ‘Deng Pao Zhai’ as the donor. (C) QTLs for GL detected in the region around 5.04 Mb on chr. 12 in the population with ‘Bei Khe’ as the donor. ■ Homozygous for donor alleles; □ homozygous for ‘Koshihikari’ alleles; ■ heterozygous. △ Donor alleles that increase trait values. LOD, logarithm of odds; AE, additive effect; *R*^2^, explained variance; n.s., not significant.

**Fig. 5.**
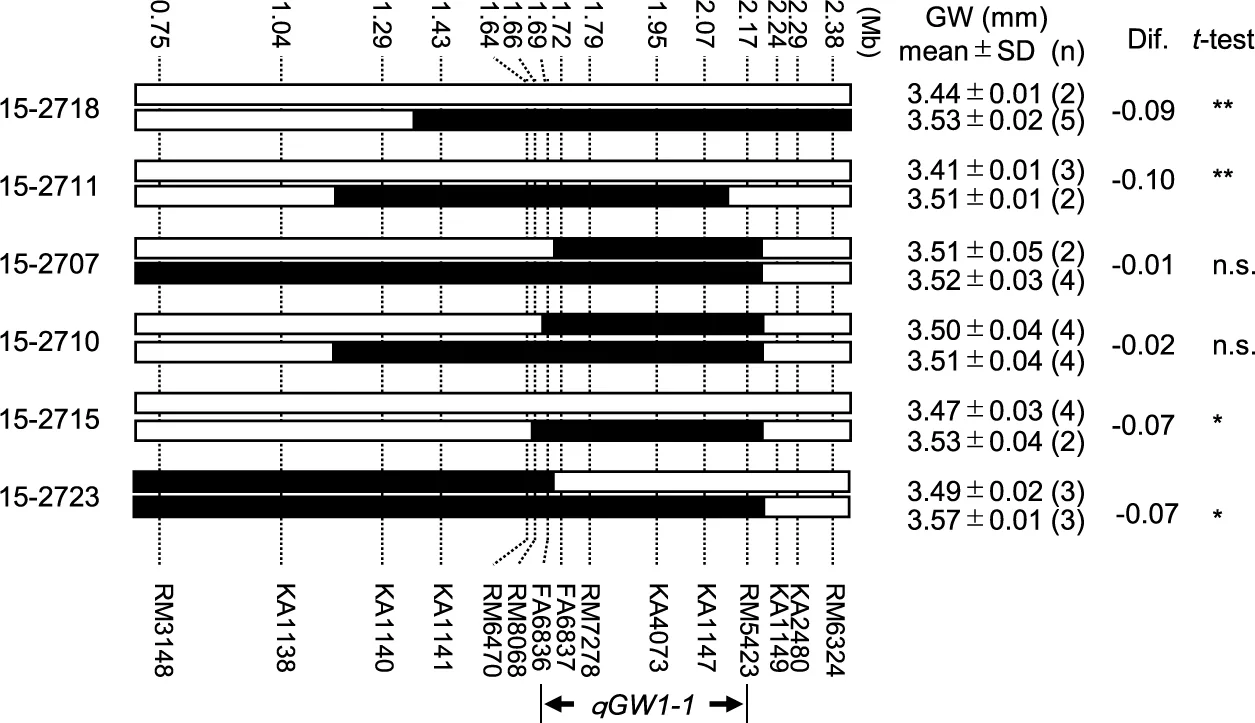
Delimitation of *qGW1-1* for GW using progeny lines of sub-CSSLs. Graphical genotypes of six lines derived from sub-CSSLs are shown. Plants were genotyped, and two genotype classes—homozygous lines differing in the size of ‘IR64’-derived introgressions—were selected for GW measurement. ■ Homozygous for donor (‘IR64’) alleles; □ homozygous for ‘Koshihikari’ alleles. Values are presented as mean ± SD (n), where n denotes the number of individuals analyzed. Dif., difference in mean GW between the two genotype classes. Significant differences by *t*-test: *0.01 < *P* < 0.05, **P < 0.01; n.s., not significant.

**Table 1.** Ranges and mean values of grain length and grain width in 12 sets of backcrossed populations and their parental lines

Code	Donor			Recurrent	Number of populations	Year	Grain length (mm)						Grain width (mm)					
	Cultivar name*^a^*	Origin	Subspecies	Cultivar name			Donor	Recurrent	Backcrossed population				Donor	Recurrent	Backcrossed population			
							Av. ± SD	Av. ± SD	Av. ± SD	Max	Min	Range (%)*^b^*	Av. ± SD	Av. ± SD	Av. ± SD	Max	Min	Range (%)*^b^*
LAK	LAC23 (LA)	Liberia	*japonica*	Koshihikari (KO)	31	2012	9.06 ± 0.10	7.21 ± 0.08	7.21 ± 0.20	8.34	6.75	1.59 (86)	3.30 ± 0.03	3.41 ± 0.06	3.41 ± 0.07	3.62	3.11	0.51 (464)
OWK	Owarihatamochi (OW)	Japan	*japonica*	–	29	–	8.27 ± 0.14	7.12 ± 0.06	7.16 ± 0.11	7.50	6.75	0.75 (63)	3.60 ± 0.04	3.41 ± 0.04	3.39 ± 0.05	3.56	3.22	0.34 (179)
BKK	Bei Khe (BK)*	Cambodia	*indica*	–	39	2013	8.17 ± 0.12	6.91 ± 0.12	6.96 ± 0.18	7.56	6.37	1.19 (94)	2.65 ± 0.02	3.37 ± 0.06	3.33 ± 0.11	3.61	2.86	0.75 (104)
TUK	Tupa 121-3 (TU)*	Bangladesh	*indica*	–	34	–	8.08 ± 0.12	7.01 ± 0.08	7.06 ± 0.21	7.82	6.46	1.36 (127)	2.70 ± 0.04	3.40 ± 0.04	3.38 ± 0.11	3.64	2.94	0.70 (100)
KNK	Khao Nam Jen (KN)*	Laos	*japonica*	–	33	–	7.95 ± 0.14	6.97 ± 0.19	6.98 ± 0.15	7.99	6.98	1.01 (103)	3.97 ± 0.06	3.40 ± 0.10	3.42 ± 0.06	3.72	3.25	0.47 (82)
KMK	Khau Mac Kho (KM)*	Vietnam	*japonica*	–	35	–	8.05 ± 0.10	6.92 ± 0.08	6.98 ± 0.15	7.84	6.55	1.29 (114)	3.98 ± 0.05	3.37 ± 0.04	3.39 ± 0.07	3.71	3.16	0.55 (90)
DPK	Deng Pao Zhai (DP)*	China	*indica*	–	29	2014	7.86 ± 0.07	6.93 ± 0.07	7.04 ± 0.16	7.59	6.54	1.05 (113)	3.08 ± 0.02	3.39 ± 0.03	3.34 ± 0.11	3.64	2.85	0.79 (255)
NAK	Naba (NA)*	India	*indica*	–	32	–	8.16 ± 0.08	6.95 ± 0.08	7.05 ± 0.16	7.63	6.16	1.47 (119)	2.77 ± 0.02	3.40 ± 0.03	3.34 ± 0.11	3.62	2.89	0.73 (116)
BLK	Bleiyo (BL)*	Thailand	*indica*	–	31	–	8.97 ± 0.18	6.96 ± 0.06	7.05 ± 0.20	8.27	6.57	1.70 (85)	2.51 ± 0.05	3.39 ± 0.03	3.36 ± 0.08	3.58	2.98	0.60 (64)
QZK	Qiu Zhao Zong (QZ)*	China	*indica*	–	33	–	8.15 ± 0.17	6.99 ± 0.07	7.05 ± 0.15	7.69	6.59	1.10 (95)	2.93 ± 0.04	3.38 ± 0.02	3.38 ± 0.10	3.61	2.87	0.74 (164)
MUK	Muha (MU)*	India	*indica*	–	36	2015	8.67 ± 0.09	7.01 ± 0.09	7.11 ± 0.20	7.93	6.48	1.45 (87)	2.95 ± 0.04	3.42 ± 0.05	3.40 ± 0.09	3.67	3.01	0.66 (140)
BAK	Basilanon (BA)*	Philippines	*indica*	–	36	–	7.19 ± 0.13	6.89 ± 0.10	7.02 ± 0.17	7.68	6.23	1.45 (483)	2.48 ± 0.03	3.36 ± 0.07	3.33 ± 0.10	3.59	2.99	0.60 (68)
					CV(%)		6.1	1.3	1.0	3.5	3.5		17.2	0.5	1.0	1.3	4.7	

*^a^* Cultivar names marked with asterisks were selected from the World Rice Core Collection (WRC) maintained by the NARO Genebank. Abbreviations for the cultivar names are indicated in parentheses. *^b^* Values in parentheses indicate the range as a proportion of that of the respective parent.

**Table 2.** A QTL catalogue for grain length in rice, showing the additive effect of QTLs detected in 398 BC_3–5_F_2_ populations derived from the cross between 12 donor cultivars and the recurrent parent, ‘Koshihikari’

Chr.	Chromosome interval for QTL peak (Mb)		Additive effects (mm)*^c^*													Cloned or molecularly characterized genes assigned to chromosomal intervals and adjacent regions*^e^*
		QTL name*^b^*	IRK*^b^*	BKK	DPK	NAK	BLK	QZK	TUK	MUK	BAK	LAK	OWK	KNK	KMK	
			*indica*	*indica*	*indica*	*indica*	*indica*	*indica*	*indica*	*indica*	*indica*	*japonica*	*japonica*	*japonica*	*japonica*	
1	1.67–7.57*^a^*				0.090	−0.075						−0.095	−0.096	−0.147	−0.163	*SERL5*, *GSE1.2*, *D2*
													−0.058			
1	12.49–15.12	*qGL1-1*	0.102				0.052	0.074		0.059						*miR408*
1	19.12–24.74				0.053			0.061					−0.084			
1	26.91–34.51*^a^*	*qGL1-3*	−0.117	−0.091	−0.103	−0.116	−0.144	−0.137	−0.154	−0.094	−0.166	−0.112	−0.105		−0.152	*BG3*, *D61*
				0.125												
1	36.47–41.11	*qGL1-4*	0.116	0.070	0.116	0.123	0.088	0.140			0.092		0.089		0.057	*OsMYB73*, *ARF4*, *CKX4*, *qGSE1.2*
2	0.12–4.41	*qGL2-1*	0.076	0.083					0.091	0.097	0.147	0.119	0.090	0.077	0.103	*RGG2*, *SPL4*
2	10.19–14.59							−0.054	−0.069	−0.062		−0.098	−0.061	0.063		*miR529a*
2	24.02–31.20*^a^*	*qGL2-3*	0.080			−0.035		−0.130					−0.051			*GS2*
						0.096										
2	33.08–35.26	*qGL2-4*	−0.023		0.032										−0.085	*SMG1*, *qTGW2*, *miR396f*
3	0.12–3.29			−0.100	−0.080	−0.095	**−0.229**	−0.153	−0.121	−0.193		−0.048	−0.039			*OsMADS47*, *VQ13*, *LG3*
3	5.53–8.55								−0.091			−0.060	−0.027	−0.110		*LGY3*, *GSW3.1*, *HDR3*
3	14.51–18.41*^a^*	*qGL3-2*	−0.304				**−0.392**			0.064		**−0.387**	n.a.*^d^*	**−0.412**	**−0.317**	*GS3*, *SMG3*, *IGL1*
										**−0.307**						
3	25.11–28.78	*qGL3-3*	−0.102	−0.145	−0.122			−0.061	**−0.257**		−0.090	−0.049	n.a.*^d^*		−0.056	*brd1*, *RGG1*, *MIS2*, *GL3.1*, *RGB1*, *GF14f*, *DDM1b*, *GIF1*
3	32.44–36.16			−0.076		0.061					−0.094	−0.015	−0.093	−0.154		*GSA1*, *OsGL3.6*, *TGW3*
4	2.03					0.057						0.080	0.100			
4	6.94–13.88			0.100						0.103		0.113	0.091		0.117	
4	19.9–23.84					−0.115		−0.097	−0.092	−0.136		−0.084	−0.061	−0.104		*An-1*, *GIF1*, *D11*
4	29.89–34.89				−0.081		−0.099					0.050	0.072			*SMG2*, *CYP704A3*, *XIAO*, *qGL4*, *AGO2*, *miR396e*
5	0.17–5.79	*qGL5-1*	−0.159	−0.128	−0.104	−0.121		−0.160	−0.119	−0.113	−0.143			0.065		*MKP1*, *GW5*, *GSK2*
5	13.43–19.00					−0.071	−0.089	−0.057			−0.182		0.060			*D1*, *WRKY53*, *SMOS1*
5	23.91–29.61	*qGL5-3*	−0.092	0.042	−0.061	0.043	−0.078		−0.078		−0.054				−0.078	*GL5*, *qGSN5*, *qGL5.2*, *PUP7*
6	0.21–2.27	*qGL6-1*	0.093	0.100	0.030	0.104		0.054	0.114		0.080		−0.055			*MAPK6*, *DA1*
6	8.75–13.89				−0.058	0.058	−0.055	0.084		0.172	0.106	−0.051	−0.061		0.057	*BZIP47*, *GW6*
6	19.70–24.63	*qGL6-3*	0.069					0.063			−0.126					*TGW6*
6	25.83–30.97	*qGL6-4*	−0.096	−0.089	−0.045	−0.067	−0.121	−0.102	−0.101	−0.157		−0.040	−0.085		−0.171	*GW6a*, *SGD1*, *GL6*, *FD2*
7	0.65–7.69				0.055	−0.065	−0.043							0.068		
7	12.71–19.36	*qGL7*	−0.105	−0.168	−0.111		−0.084	0.072	−0.066		−0.120	−0.084			−0.067	*GLW7*
7	21.61–23.65					−0.089		−0.080		−0.121				0.059		*GW7/GL7*, *GE*
7	26.46–29.04								0.171	0.168	0.186			−0.093	−0.103	*SDR7-6*
8	0.13–7.49	*qGL8-1*	−0.078		−0.070	−0.074				0.059			0.050		−0.086	
8	21.58–28.25*^a^*			−0.088	−0.143		−0.162	−0.112	−0.052	−0.055						*GGC2*, *GAD1*, *GW8*, *WTG1*, *SLG*
										−0.075						
9	1.55–6.59	*qGL9-1*	0.051	0.050					0.086	−0.029				0.038		*GSE9*
9	9.21–16.94			0.060	−0.051		−0.051	−0.064		−0.066		0.106				*DEP1*, *GS9*
9	17.89–20.55	*qGL9-2*	0.072		0.109			0.122		0.050	−0.102					
10	2.74									0.020						
10	10.63–11.74									−0.051			−0.035			*GS10*
10	17.47–23.11*^a^*		*−0.088*	−0.055	0.109	0.076	0.079		0.058	−0.103	0.098	0.065		0.073	0.118	*GW10*, *GL10*
		*qGL10*	0.116													
11	3.83–8.11						−0.078			−0.127						*RCN1*, *Cyclin-T1;3*, *GL11*, *SRS5*
11	14.53–20.14			−0.069						0.082	−0.047	−0.105			0.134	
11	21.52–28.96			−0.119					0.115	0.047		0.105				*OsGIF1*
12	3.38–5.04	*qGL12*	−0.058	−0.124		−0.045					−0.053				−0.074	
12	9.10–12.24				−0.094				−0.166							*qGW12*
12	19.96–24.01			−0.079				0.087		0.086						*OsGIF2*, *GL12*, *miR167a*
Number of QTLs detected				21	21	20	16	21	18	27	17	20	21	13	17	
							*indica*		*japonica*		total					
	Total QTLs detected in 12 donors															
		Number of QTLs (QTLs per donor)					161	(20.1)	71	(17.8)	232	(18.8)				
		Mean absolute value of additive effects					0.095		0.094		0.094					
	QTLs whose absolute value of additive effect >0.2															
		Number of QTLs (QTLs per donor)					4	(0.5)	3	(0.8)	7	(0.6)				
		Mean absolute value of additive effects					0.296		0.372		0.329					
	QTLs whose absolute value of additive effect >0.15															
		Number of QTLs (QTLs per donor)					18	(2.3)	7	(1.8)	25	(2.2)				
		Mean absolute value of additive effects					0.197		0.251		0.212					

*^a^* A chromosomal interval that may contain multiple QTLs. *^b^* Data are taken from Nagata *et al.* (2015). One QTL allele in italics, located in the region 17.47–23.11 Mb on chr. 10, was not assigned in that paper. *^c^* Additive effects of the ‘Koshihikari’ allele on grain length: absolute values **>0.2 mm** and >0.15 mm. For QTLs with additive effects in the same direction detected within an interval across multiple populations from a donor, the value from the population with the largest phenotypic variation explained by the QTL was used. *^d^* The region from 14.28 to 27.89 Mb on chromosome 3 in OWK was not analyzed owing to a loss of the donor’s chromosome in an early backcross generation. *^e^* Includes genes reported in the review articles by Li *et al.* (2019) and Xuedan *et al.* (2023), as well as in research articles cited in this paper. Gene symbols were selected from RAP-DB synonyms.

**Table 3.** A QTL catalogue for grain width in rice, showing the additive effects of QTLs detected in 398 BC_3–5_F_2_ populations derived from the crosses between 12 donor cultivars and the recurrent parent, ‘Koshihikari’

Chr.	Chromosome interval for QTL peak (Mb)		Additive effects (mm)*^c^*													Cloned or molecularly characterized genes assigned to chromosomal intervals and adjacent regions*^e^*
		QTL name*^b^*	IRK*^b^*	BKK	DPK	NAK	BLK	QZK	TUK	MUK	BAK	LAK	OWK	KNK	KMK	
			*indica*	*indica*	*indica*	*indica*	*indica*	*indica*	*indica*	*indica*	*indica*	*japonica*	*japonica*	*japonica*	*japonica*	
1	0.30–4.97	*qGW1-1*	−0.055	−0.040	−0.034	−0.047		−0.039	−0.053	−0.058	−0.025	−0.035		−0.086	−0.110	*SERL5*, *GSE1.2*, *GW5L*, *D2*
1	7.28–9.94												−0.053		−0.042	
1	19.12–20.94						−0.042			0.041				0.050		
1	25.07–29.90	*qGW1-3*	0.026	0.035		0.019		−0.019	0.032				0.014			*UBC13*, *BG3*, *D61*
1	30.09–34.69	*qGW1-4*	−0.015	0.044			−0.043	0.034		−0.065	0.029	0.033				
1	40.21–42.93		0.046	0.044			0.023					−0.035		−0.043		*OsMYB73*, *ARF4*, *CKX4*
2	0.12–3.83	*qGW2-1*	0.064	0.060		0.042			0.052		0.040			0.025		*RGG2*, *SPL4*
2	7.48–10.19	*qGW2-2*	0.026		0.106			0.060				0.034			0.039	*FUWA*, *GW2*, *UBP15*, *SLG2*
2	11.23–15.19		*0.062*					0.067	0.051	0.067		0.036				*miR529a*
2	18.46–22.80			0.058	0.091	0.071	0.055	0.025			0.075		0.030			*WG1*, *SGW5*
2	25.83–27.93	*qGW2-3*	0.063			0.064										*GS2*
2	33.08–35.77				0.050	0.083	0.020	0.029	0.020	0.067						*TGW2*, *SMG1*, *qTGW2*, *miR396f*
3	0.12–1.16	*qGW3-1*	−0.043	−0.040			−0.035	−0.035	−0.026				−0.015	−0.036		*VQ13*
3	5.86–10.62		*0.028*	0.027		0.075	0.065	0.062	0.064		0.044	0.037	0.047	−0.045		*OsMADS47*, *PUP1*, *LGY3*, *HDR3*, *GSW3.1*
3	16.73–22.40	*qGW3-3*	0.011	0.061	0.077		0.034				0.061		n.a.*^d^*	−0.041	0.028	*GS3*, *SMG3*, *brd1*, *RGG1*, *MIS2*
3	27.43–28.78						−0.060	−0.082	−0.047			−0.035	−0.016		−0.054	*GL3.1*, *RGB1*, *GF14f*, *DDM1b*, *GIF1*
3	32.44–36.35	*qGW3-4*	0.044	0.066	0.041					0.038	0.068	0.004	0.032			*GSA1*, *TGW3*
4	0.06–4.04					−0.019	−0.032	−0.024	−0.032				−0.029	−0.032		
4	13.08					−0.027										
4	17.10–24.25	*qGW4-2*	−0.022	−0.032		0.022	0.025	0.022	−0.027	0.057		0.050	0.030		0.043	*An-1*, *GIF1*, *D11*
4	28.76–34.89				−0.069	−0.023	−0.035	−0.061	0.037				0.020			*SMG2*, *CYP704A3*, *qGL4*, *miR396e*
5	0.45–5.88	*qGW5-1*	0.150	**0.208**	**0.178**	**0.175**	**0.164**	**0.204**	**0.197**	**0.160**	**0.161**	**0.160**	0.028	−0.059		*MKP1*, *GS5*, *qGW5*, *GSK2*
5	20.06–22.11			−0.030											−0.040	*WRKY53*, *SMOS1*
5	25.15–29.54	*qGW5-2*	0.019		0.035		0.049		−0.022		0.051			−0.065		*qGSN5*, *qGL5.2*, *PUP7*, *WOX9C*
6	0.21–3.17*^a^*	*qGW6-1*	0.026	0.076		−0.035		−0.033		−0.021						*MAPK6*, *DA1*, *HGW*
		*qGW6-2*	−0.041													
6	7.62–12.45*^a^*	*qGW6-3*	0.027			−0.035		−0.037	0.056	−0.016	0.077				0.027	*BZIP47*, *GW6*
								0.029		0.057						
6	16.09–22.30	*qGW6-5*	0.037	0.063		0.015	0.050									
6	25.83–30.97	*qGW6-7*	−0.039	−0.041	−0.046	−0.037	−0.037	−0.042	−0.017		−0.038		−0.034		−0.037	*GW6a*, *SGD1*, *FD2*
7	3.14–4.14				−0.036	−0.057	−0.026	−0.031								
7	9.08–13.44	*qGW7-1*	−0.052	−0.062							−0.023		−0.033		−0.052	
7	17.47–19.36			−0.039	−0.047		−0.034		−0.042		−0.060	−0.040				*GLW7*
7	23.65–29.04								0.039	0.064	0.056			−0.035	−0.064	*GW7/GL7*, *GE*, *SDR7-6*, *WG7*
8	0.13–2.96			−0.045		0.015										*SG2*
8	6.16–7.57													−0.033	−0.055	
8	17.82–22.17	*qGW8*	−0.035	−0.070	−0.050	−0.065	−0.048	−0.064			−0.081					
8	24.63–28.24						0.036		0.112	0.099	0.056					*GW8*, *WTG1*, *SLG*
9	1.55–3.91						−0.027	−0.028		−0.040				−0.011		*GSE9*
9	10.47–16.24							−0.023	−0.033			0.042	−0.033			*GS9*
9	17.89–22.58*^a^*	*qGW9-1*	0.037	0.023		−0.017			−0.010	0.034		−0.048	−0.047	−0.036	−0.040	
										−0.045						
10	3.66				0.014											
10	7.94–10.63							0.027					0.032			*GS10*
10	17.47–23.11	*qGW10-2*	0.026	−0.025	−0.102				0.043	0.017	0.047	0.041		0.023		*GW10*, *GW10.2*
11	0.22–3.83	*qGW11-1*	0.028	0.034	0.027			0.034		−0.034		0.035		0.025		*RCN1*
11	17.49–20.14									−0.041		−0.030				
11	23.47–26.03	*qGW11-2*	0.043					−0.032	0.032		−0.049					
12	0.40–0.91			−0.046			−0.023									
12	11.34														0.023	*qGW12*
12	19.12–24.42	*qGW12-3*	−0.050			0.040	0.045	0.048		0.046			0.046		0.047	*qTGW12a*
12	27.34				0.023	0.039			0.053							
Number of QTLs detected				24	17	22	23	26	23	20	18	16	17	16	15	
							*indica*		*japonica*		total					
	Total QTLs detected in 12 donors															
		Number of QTLs (QTLs per donor)					173	(21.6)	64	(16.0)	237	(19.8)				
		Mean absolute value of additive effects					0.050		0.040		0.048					
	QTLs whose absolute value of additive effect >0.15															
		Number of QTLs (QTLs per donor)					8	(1.0)	1	(0.3)	9	(0.8)				
		Mean absolute value of additive effects					0.181		0.160		0.179					
	QTLs whose absolute value of additive effect >0.075															
		Number of QTLs (QTLs per donor)					21	(2.6)	3	(0.8)	24	(2.0)				
		Mean absolute value of additive effects					0.123		0.119		0.123					

*^a^* A chromosomal interval that may contain multiple QTLs. *^b^* Data are taken from Nagata *et al.* (2015). Two QTL alleles in italic in regions 11.23–15.19 Mb on chr. 2 and 5.86–10.62 Mb on chr. 3 were not assigned in that paper. *^c^* Additive effects of the ‘Koshihikari’ allele on grain width: absolute values **>0.15 mm** and >0.075 mm. For QTLs with additive effects in the same direction detected within an interval across multiple populations from a donor, the value from the population with the largest phenotypic variation explained by the QTL was used. *^d^* The region from 14.28 to 27.89 Mb on chromosome 3 in OWK was not analyzed owing to loss of the donor’s chromosome in an early backcross generation. *^e^* Includes genes reported in the review articles by Li *et al.* (2019) and Xuedan *et al.* (2023), as well as in research articles cited in this paper. Gene symbols were selected from RAP-DB synonyms.

**Table 4.** Total number of lines and number of lines per chromosome in each set of sub-CSSLs derived from 13 donor cultivars. The additional donor set corresponds to ‘IR64’-derived sub-CSSLs that were included specifically for QTL validation and delimitation analyses, whereas the genome-wide QTL catalog was constructed using 12 donor cultivars

Code	Total	Chr. 1	Chr. 2	Chr. 3	Chr. 4	Chr. 5	Chr. 6	Chr. 7	Chr. 8	Chr. 9	Chr. 10	Chr. 11	Chr. 12	Hetero. (%)*^b^*
IRK	463 (11.0)*^a^*	91 (30.3)	33 (8.3)	43 (14.3)	43 (14.3)	39 (13.0)	35 (7.0)	41 (8.2)	23 (7.7)	27 (9.0)	38 (9.5)	21 (10.5)	29 (7.3)	4.6
LAK	501 (10.9)	73 (10.4)	53 (10.6)	65 (10.8)	40 (13.3)	46 (11.5)	54 (13.5)	22 (11.0)	26 (13.0)	25 (8.3)	27 (13.5)	41 (10.3)	29 (7.3)	2.9
OWK	527 (12.0)	69 (17.3)	40 (8.0)	35 (17.5)	91 (30.3)	41 (13.7)	50 (12.5)	28 (5.6)	22 (5.5)	47 (15.7)	42 (14.0)	33 (6.6)	29 (9.7)	2.7
BKK	400 (9.3)	45 (9.0)	40 (20.0)	41 (13.7)	22 (7.3)	28 (9.3)	58 (19.3)	50 (12.5)	15 (7.5)	29 (7.3)	26 (5.2)	29 (7.3)	17 (3.4)	2.6
TUK	512 (10.7)	35 (5.8)	66 (16.5)	74 (18.5)	44 (7.3)	60 (20.0)	25 (8.3)	47 (15.7)	24 (4.8)	41 (10.3)	29 (9.7)	30 (6.0)	37 (18.5)	2.7
KNK	429 (10.7)	59 (9.8)	24 (8.0)	57 (19.0)	36 (12.0)	37 (12.3)	34 (11.3)	35 (8.8)	22 (7.3)	32 (16.0)	26 (13.0)	33 (11.0)	34 (6.8)	2.6
KMK	416 (9.0)	51 (12.8)	56 (11.2)	47 (9.4)	15 (3.8)	21 (7.0)	25 (12.5)	53 (13.3)	43 (14.3)	13 (3.3)	28 (7.0)	34 (8.5)	30 (7.5)	2.4
DPK	317 (8.8)	35 (8.8)	44 (22.0)	35 (17.5)	15 (5.0)	21 (7.0)	40 (8.0)	32 (10.7)	18 (6.0)	14 (3.5)	21 (10.5)	23 (11.5)	19 (6.3)	3.3
BLK	277 (6.8)	31 (7.8)	21 (3.5)	34 (17.0)	31 (10.3)	29 (5.8)	19 (6.3)	18 (4.5)	24 (8.0)	16 (8.0)	15 (7.5)	24 (8.0)	15 (3.8)	2.9
NAK	359 (8.8)	42 (14.0)	38 (12.7)	19 (4.8)	27 (9.0)	36 (9.0)	28 (5.6)	23 (11.5)	39 (9.8)	34 (8.5)	27 (9.0)	30 (15.0)	16 (4.0)	3.1
QZK	376 (8.7)	61 (15.3)	24 (4.0)	54 (18.0)	44 (11.0)	21 (5.3)	22 (5.5)	29 (7.3)	15 (5.0)	20 (10.0)	27 (9.0)	30 (10.0)	29 (9.7)	3.1
MUK	423 (10.1)	43 (8.6)	23 (5.8)	35 (7.0)	49 (16.3)	29 (9.7)	29 (7.3)	42 (14.0)	44 (11.0)	26 (13.0)	28 (14.0)	55 (18.3)	20 (5.0)	2.4
BAK	463 (12.9)	52 (13.0)	54 (27.0)	55 (27.5)	11 (3.7)	58 (19.3)	45 (9.0)	39 (13.0)	17 (5.7)	27 (6.8)	18 (9.0)	51 (25.5)	36 (12.0)	3.2
Mean	420 (10.0)	53 (11.6)	40 (10.1)	46 (13.5)	36 (10.6)	36 (10.6)	36 (9.3)	35 (10.0)	26 (7.9)	27 (8.6)	27 (9.5)	33 (10.3)	26 (7.1)	3.0

*^a^* Values in parentheses indicate the proportion relative to those in the corresponding CSSLs previously reported by Abe *et al.* (2013), Nagata *et al.* (2015), Mizuno *et al.* (2018), and Nagata *et al.* (2023). *^b^* Mean heterozygosity that reflect the average proportion of heterozygous loci per line within each sub-CSSL set.
