# Supplementary material for: Series of advanced backcross populations uncovers diversity and complexity in genetic control of grain length and grain width in Asian cultivated rice
Source: Breed Sci. 2026 Jun 11;76(3):251–62. doi: 10.1270/jsbbs.25071 (PMC13709637; doi:10.1270/jsbbs.25071)
Supplement: Supplementary file 1 [file 76_251_s1.pdf]

doi: 10.1270/jsbbs.25071

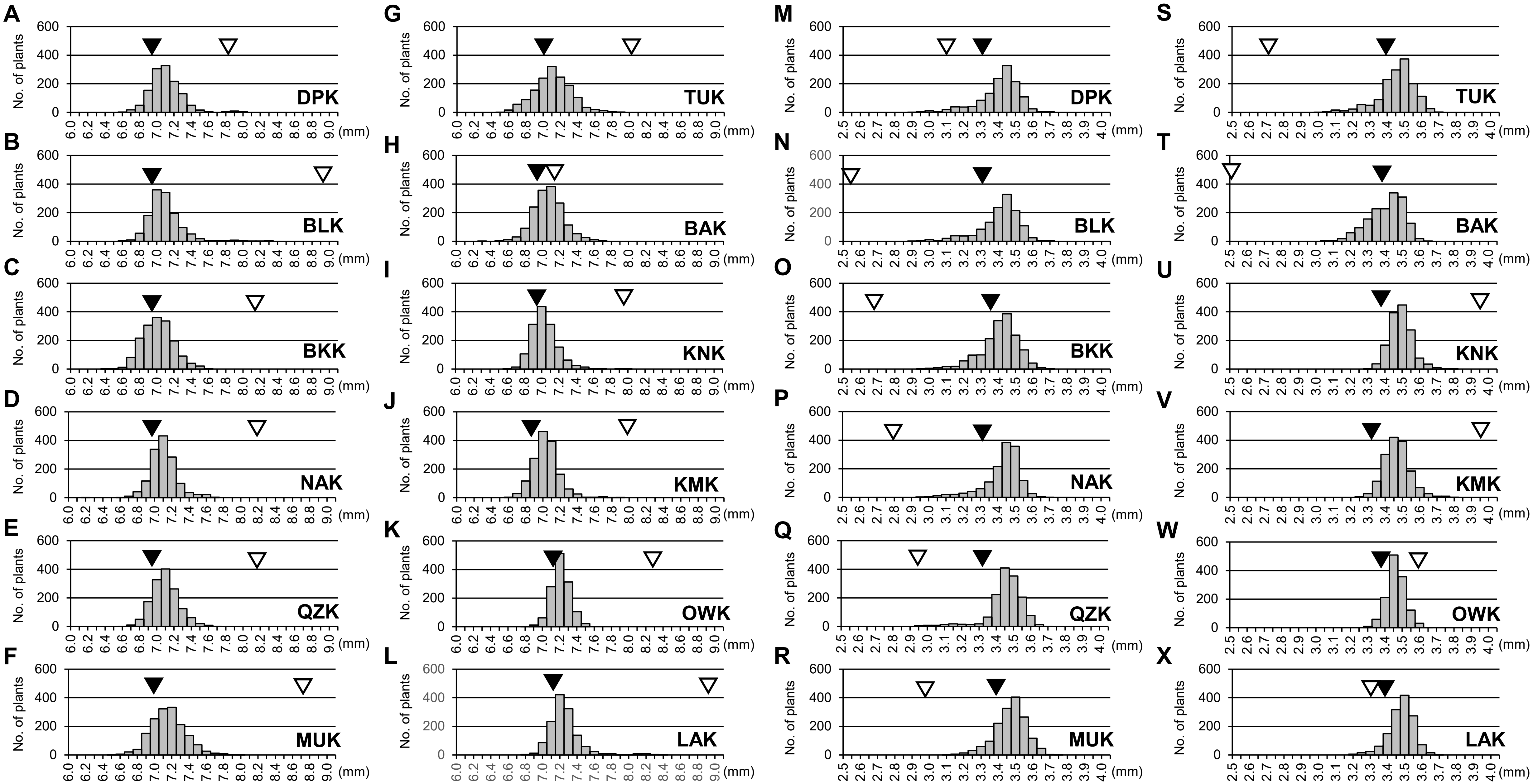

Supplemental Fig. 1 Frequency distributions of (A–L) GL and (M–X) GW in 12 backcrossed populations. Triangles: mean values of ▼ recurrent and ▽ donor parents. Population codes are indicated in the lower right corner of each graph; see Table 1 for details.

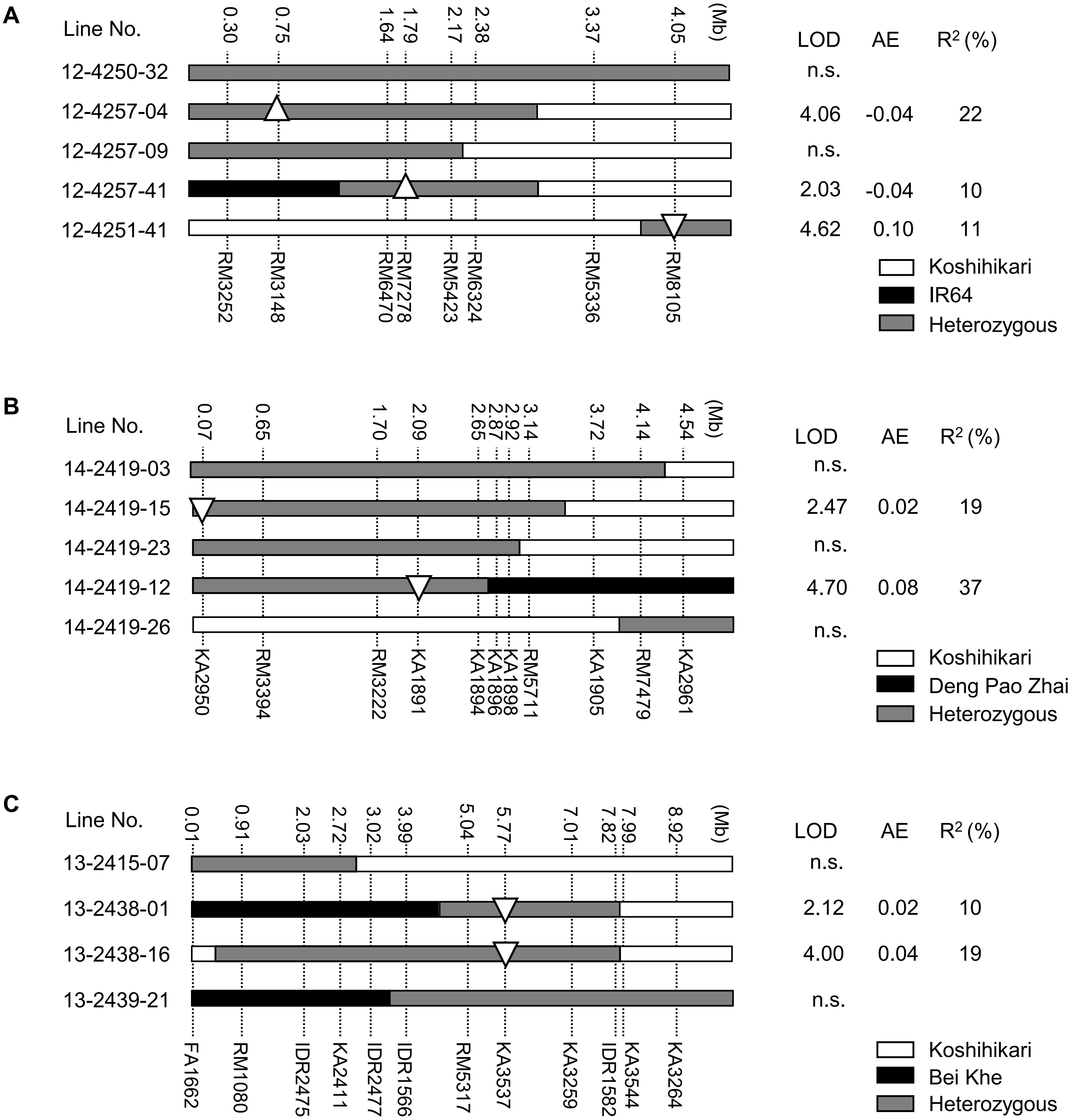

Supplemental. Fig. 2. Validation of QTLs for GL and GW detected in $BC_{3–5}F_2$ populations by using sub-CSSLs. Graphical genotypes of sub-CSSLs and the positions of QTLs detected in each sub-CSSL are shown. (A) QTLs for GL detected near *qGW1-1* for GW (0.75–2.38 Mb) on chr. 1 in the population with 'IR64' as the donor. (B) QTLs for GL detected near *qGW7-2* for GW (2.65–3.14 Mb) on chr. 7 in the population with 'Deng Pao Zhai' as the donor. (C) QTLs for GW detected near *qGL12-1* for GL (3.02–7.82 Mb) on chr. 12 in the population with 'Bei Khe' as the donor. ■ Homozygous for donor alleles; □ homozygous for 'Koshihikari' alleles; ■ heterozygous. LOD, logarithm of odds; AE, additive effect; R^2, explained variance; n.s., not significant. Triangles: donor alleles that △ increased or ▽ decreased trait values.
