# Supplementary material for: Series of advanced backcross populations uncovers diversity and complexity in genetic control of grain length and grain width in Asian cultivated rice
Source: Breed Sci. 2026 Jun 11;76(3):251–62. doi: 10.1270/jsbbs.25071 (PMC13709637; doi:10.1270/jsbbs.25071)
Supplement: Supplementary file 2 [file 76_251_s2.pdf]

Nagata et al./Breeding Science
doi: 10.1270/jsbbs.25071

Supplemental Table 1. List of primers used for QTL detection in 398 backcrossed populations ($BC_{3\text{-}5}F_2$) and for validation in sub-CSSLs.

Marker name	Chr.	Position (Mb)[a]	Size (bp)[b]	Primer sequences (5' to 3') Forward	Reverse	
RM3252	1	0.30	174	AGGTAACTTTGTTCCCATGCC	GGTCAATCATGCATGCAAGC	
RM8047	1	0.30	193	ATATTATTCTGAAATGCATGTAGG	ATACTATTAAACTTGCTCTCAAACT	
RM4959	1	0.46	149	AGTACAATATTTTTGGTAGGA	CAACCAGCTTAACTAATTAA	
RM3148	1	0.75	167	AGACTATTGCTCGAACACTTTG	GTCTGCTTTGGTATTTGC	
RM6470	1	1.64	175	ACCTTTCCCATGGTGGAATC	ATACCTTCGACGGGAACG	
RM8068	1	1.66	128	ACAAACCTCTCGCTGTAATTAG	GAACATTTATTGATATGGTAAA	
RM1331	1	1.67	142	ACACCAGCTTCATGCATGC	AGCACTCAACTGATGCAGTG	
RM7278	1	1.79	176	ATGCTAGTCTGATGAATGCCG	AGAACACCACATGGCAGC	
RM5423	1	2.17	202	ATCCCACTTGCAGACGTAGG	ACAGCAGCAAGGTGCCTC	
RM6324	1	2.38	148	ATGTACAAGAACGGCAGCAAC	GCACCACCAAACAGAGAC	*[d]
RM3740	1	2.80	136	ATCCCAACTCTAAGCCACCC	CTACCCGTCACCAACTCACC	
RM5336	1	3.37	117	ATCGATTTGGTCGCGATTG	AGAAATCCCCGACCACCTC	
RM7383	1	3.48	176	AGCGCACTCCACTCTCTCTC	AAGGGCTGGAGAAGGAGAAG	
RM6902	1	3.80	234	ACGGAGAAGACCGAGACGTAG	CCCGTGTAAAAGTTGGGAC	
RM8105	1	4.05	100	ATCAGCATTTTCAAGCCTAAG	GGCTGGCGTGTAGGATAC	*
RM1329	1	3.95	152	AGAGCTCAATCGAATCTAGACC	ATTGACATTCCTTTGCTTTG	
RM5302	1	4.15	143	ATATGGGTGACACATTGGGAC	GTGACGTTTGAGAGCTGG	
RM3521	1	4.40	210	ACCTTCGAGTGCATGTCCC	GAACACGAGGAGAATGAGCC	
RM5641	1	4.97	145	ATGATGGCACAGCTTCAGAAC	CTAATTCTGGGCGAGCAAAG	
RM3233	1	5.06	118	AGTGGTGAGTAAACAGTGGTGG	GAGAGCAGAGCAGAGGCAAC	
RM3746	1	6.16	110	ACAAATGGGCTTCCTCCTCTTC	CAGCCTTGATCGGAAGTAGC	
RM5443	1	6.23	147	ATACGGCTTACCCATAGCAGC	AAACGGAGGGAGTATTTCCC	
RM6599	1	7.08	162	AGGACAGCTCGGATCATCAAC	CCTGATGAGGAAAGACGAGC	
RM8078	1	7.28	159	ACAATCTTTTCCTCCTCCGTAGGT	ACCTAGCAAGACGACAATGAATCAG	
RM3598	1	7.35	106	ACGACTTCTCCTCCATTTTCG	CAAATTCACGCAGTGACCAC	
RM5800	1	7.40	217	ATGTGGTGGAAGAGAGGGAAG	CGCCATCATCGAGCTACC	
RM8142	1	7.46	120	ATTCATATATATTGGCTAGAGTGGCTGT	AAGCCAATATATGTAGAGATGAATGGAA	
RM3234	1	7.57	102	ACAAAGACGACGATGGGTCAAC	GTGAGGTTCTTGGGTGGAAG	
RM3780	1	8.02	163	ATATCCACCTTCTCCATTGGC	CGAGACCAAATAGCGAATCC	
RM8133	1	9.39	153	ACAAAACTGACTGTTTGTTTAAATGAAAT	GTTACTGCTGTAATGTGAATTGCT	
RM8071	1	9.49	146	AGTGAAGAGGGCTACCGGAAAG	GGGATTATCGCCACTACCACCT	
RM8046	1	9.94	195	AGTACGATTTCTGTCAGCGTTGCTTAGT	GGATGAAAGTTGATGGATGATCTACTTGTT	
RM3627	1	10.31	116	AGGCTACTCGAGCAAGCTCTG	ACCTACCCGTCATCCCTCTC	
RM1287	1	10.84	157	AGGAAGCATCATGCAATAGCC	GGCCGTAGTTTTGCTACTGC	
RM8094	1	11.24	211	ACAAGTTTGTACACATCGTATACA	CGCGACCAGTACTACTACTA	
RM8095	1	11.24	111	ATTCCGTGGACATGATGAATC	AAGGTTTAGAACATACACACCGTT	
RM3412	1	11.58	211	ACAAAGCAGGTTTTCCTCCTCC	CCCATGTGCAATGTGTCTTC	
IDR0045	1	11.63	142	ATTCCCTCACTCGCATATCTGT	CGGACACCCTTCTCTTTCTATG	
IDR0049	1	12.12	111	CACTAGCTAGCAAAGCAAAGCA	GAAAGGAACCGAGAATGACAAG	
RM10812	1	12.94	166	CTGCATGTGTACGAAGAATGAACG	TAGCTGGTGCCATCTTTCC	
RM10850	1	13.96	299	GTAGGCACACCAAGCACCATCG	CCATAATTCATCGGCCCTTTGC	
RM5365	1	14.52	181	ATCTGTTCGATGTTCCCATCG	AAACTCAAACAGGCTGGGC	
RM7075	1	15.12	155	ATATGGACTGGAGCAAACCTC	GGCACAGCACCAATGTCTC	
RM6681	1	15.90	237	ACAACCGGGACTATTGTGGAG	ACCCCACCCAGCCATTAC	
RM6740	1	17.28	192	AGATGGGATAGACAAGCGCAC	CCATCTAGGAGTACTAGTCTTCGC	
RM5964	1	17.58	118	ATGATCACCTGCAGGAGCAG	AAGGAGCGGAAGATCTCCC	
RM5422	1	18.76	173	ACTTGCAGGGGTTGCAATTAG	GAGGAGAGGATGAACATGAGG	
RM6092	1	19.12	144	ACAGCTCGTGGACACTCGTC	CCAAGTCCGCCCCTCTAG	
RM11069	1	19.37	280	GGTACAATGAAGCTTGGCAACG	CGGTGGAGTAGAACCACGAAGC	
RM11099	1	20.06	266	CTAGGTCTGTACACGCACTGAAGG	CAAATAGCTCGACACAGGAGATCG	
RM5638	1	20.94	203	AGGCTTCCTCATCGCCATC	CTGAGCAGCATTCCAGTCTG	
RM8090	1	21.43	160	AGAAAACATATGGAAATTTTCTGACTGTA	ATATTGAAAACGGTAACCATTGGTAA	
RM1196	1	21.90	188	AGCTGCCGTGAGCCTCAAG	CCAAAACGCTCTCTTCGTC	
RM5853	1	22.74	200	AGTAGCAGTAGTAGTACCACCGACG	ACCAGTGTGGTTTCTTTGGG	
RM5718	1	22.93	257	ATCCCGTGTGTTATTTCGGAC	CTCTGTCAGATGCCCTTAACG	
RM6716	1	23.12	153	ATCCATATTCCTTCCCCTCG	CTGGACCGAAGAAGAACAGC	
RM7124	1	24.39	168	ATCGGTTAAGCTGAAACCCC	AACCGTGAACACACACATGC	
RM7266	1	24.45	206	ATGGCTTTTTGGAACGTGC	CGGCTTGAGAGCGTTTTTAG	
RM5919	1	24.74	237	AAACAGTCAGCGGCTTTGTC	ATAGCGTTTGACGGGACAAC	
RM1349	1	25.07	161	ACGTTCCAATATTCAGACACAG	CCATCTCGAGAAGCTC	
IDR0110	1	25.51	150	GGGTACCAACATCAACATCTCA	CTCTGTTTCCTTTCGCTTTCTG	
RM3475	1	26.04	150	AGTCGGTTTGCCTAGTTGAGC	CCTCGGTGTATGGGTCTC	
RM3143	1	26.82	98	AGCCTGGATAAGATGGTTCG	CGAGAAGACCCAGTTTCTGC	
RM5461	1	26.91	134	AGCCTCCCATATAAACCGGC	AGCGAAGGGAACACGACAG	
RM3440	1	27.19	197	ATGTTCGAAGGAAGCTGGATC	AGCTGAAGCCAAAGCCTTC	
RM5497	1	27.54	218	AGGGCAACAACAACAACGAAC	AGGGCGTATGGCTTGAG	
RM7566	1	27.77	171	ATGGCGTAGTGGTACGTGAAC	AGAAAAAGCTAGCACGCCG	
RM1297	1	28.61	170	AGTGCCTTACAACTCAACGAC	CACTCCCAGTTCAGTACGTC	
RM3336	1	28.62	102	ATCCTGTGCTTGTGTCCTTTG	AGTCATTTGGCTCAACAGCC	
RM1244	1	28.69	144	ATTCTGGTTGACCTTGTGCTG	ACTGAGCAAACTCGACACAG	
RM3817	1	28.90	207	AAGGAAGGGGAAGCTGAGAG	CGGACTCGAGAAAAAAGCAG	
RM5931	1	29.28	194	ACTCTCCTGCCTGACAAAAGC	GTTTTAGCGGATGTGGCATC	
RM8126	1	29.28	231	ACTCCCCTCTCTTCTCATCTCTCTCCT	GGTAATATGGGCGGCTGTTTTA	
RM7371	1	29.81	156	ATTCTCCCCGTATTCTTGTGC	CACGGTAACATCGACGAC	
RM3614	1	29.83	110	AGTATCAGTTAGCCCCCGAGC	GAAGGAAGCAGAAGCAGGTG	
IDR0129	1	29.90	129	GGGAGGAAGATGTAGCAGTAGC	TTGCATAGATACACCTAAAGGAGA	

Marker name	Chr.	Position (Mb)[a]	Size (bp)[b]	Primer sequences (5' to 3') Forward	Reverse
RM5954	1	30.00	158	ACTCGTCCTCAAGTGCCTCTC	CGACTCCTACTCCGACTCC
RM1152	1	30.09	183	AGCCTTTGTCCTTCAGTAGGC	AGAGCGCCTGGGTATAATTG
RM1183	1	30.98	146	AGGGCACGAATAAAACCAGAG	GGGATGGTCCAATGACAAAG
RM5914	1	31.51	212	ATCTCCACATTTGACTCACGG	AACGGTGTGATAAGGGATGC
ID18-24	1	31.90	219	CGTGGCGTCTATGCGTATCTT	CGGTCGAGAGCTAACCATGA
RM3709	1	31.95	113	ATATATTGAGGGAGCAAGCCG	CATCCCAAAGCTAGAACCCC
RM3416	1	32.89	123	AGGCAGCAATCCCTGGATC	CACTTCTCCCCTTCCAAG
RM1003	1	33.48	129	AGATTCTTCCTCCCCTTCGTG	CCTGTCAGAACAGGGAGC
IDR0151	1	34.17	148	ATGCCATCTCTATCTCGTGGAT	AGCAGAACTGCAGGAATAGCTT
RM6703	1	34.52	198	ACAGCAAACCAAACCAAGCC	GCGAGGAGGAGGAGAAAAAG
RM5501	1	34.55	137	AGCGCTTCTACTTCCACAAGG	GGTTGGCGTACGTAGAGAGG
RM6547	1	34.69	165	ATCCATCCTTCTCCTCTCGTG	AGCCACCCCCATATATAGCC
RM3304	1	34.96	128	AGACTGCAAGCTTGTAAGCCC	AGCCACATCAACAGCTTGC
RM3403	1	35.00	121	ACAACGACTGCTCCCTCTTCAG	AGCTTGCAAGGCATTAGCTC
RM5781	1	35.77	219	ACCTCCTAAGCTCCAAACCC	AACGTCTTGTCCTCCACGAC
RM3825	1	36.47	151	ACAAAGCCCCCAAAAGCAGTAC	GTGAAACTCTGGGGTGTTCG
RM5448	1	37.40	129	AGGGGCCCTTAGAGTTTTTAC	CTGCTACAGGACAAAACCAG
RM6696	1	38.22	209	ATGGTCCACACGTGATGAGTC	GAAGATAGGGGAGTGGGGAG
RM11998	1	38.79	246/306	GGGAACTCCATACTGCTATGTCG	AGTAGGGTATCGTCGGTGTCAGG
RM6504	1	38.99	119	AGAGAGGCGGTAGTATGCTCG	CTCCAATTTCACCGTCACC
RM6292	1	39.22	199	AGAGTCCTTCCTAGCTTCCTCG	AGTCCCAGAAGCTGCTCAAG
RM3482	1	39.72	108	ATTGTTGTCAAGCTACGGTGG	CTGCTTCGTGATGTTGTTGG
RM1387	1	40.21	198	AGTGGCTGGCTGATCGATC	AATCAACCCAGCTACCATGC
RM8099	1	41.10	118	AGCACATCCACTTTCCTCTCC	GGGGGTGATGCAAACAT
RM5310	1	41.20	166	ATAGACAAAGCAACGGGTTCC	CGGAAGCAGGAGAATCGTAG
RM8087	1	41.68	177	AGAATGTATTGCACTCCAAA	GTAACCGTCCATAATGAATAAC
RM8137	1	42.93	178	AGTAATTGAATTTCACTGCTGCT	ACGTACGTGACGTGCTTATG
RM6840	1	43.17	191	ATACCAAGACTCCGCTATGGC	GAAGAAGGGATCATGGATCG
RM6831	1	43.18	157	ACTAATTTGGGTGTTGGTGCC	AGGAGAACAAGACGACGGC
RM6842	2	0.12	197	ATAAATCGAAGGAGGGGGAAG	GGAAGAAGGAGGAGGAGGTG
RM263	2	0.26	199	CCCAGGCTAGCTCATGAACC	GCTACGTTTGAGCTACCACG
RM7622	2	0.29	152	ATCCTTTCCCACAAAGGGAG	ATGTGAAAGGGCATCTCCAG
RM7451	2	0.65	154	ATAATACGAGCAGCGATCGTG	GCTAATTGCAGCTTGTGTCG
IDR0197	2	0.71	136	AGCCTCTTCTTCCCATATCTCC	TCTGTAGCACATCATCCTCAGC
RM2770	2	0.81	191	ATAGGCCCTGATTAGTTTCC	ATATATGTGTCCCTTCTCCATAC
RM6938	2	0.98	206	ACTCCGCGGAGTCGATTTTA	AACCAACCACTGATTATTCCAACT
RM6367	2	1.13	203	ACAGACAGAACAGCGGTCAAG	GATGGATGGATGGATTGGAG
RM7562	2	1.88	155	AGACATGCCAATGTGATGGC	CGGTAGTATGGGGCTTGTC
RM5529	2	2.22	167	AGCCGAAACTACATTCGGTG	GTGTAGTTGGCACGCTTC
RM3188	2	3.45	115	ATCACGAGTCGTTCGTTCTTG	CTTGCTGCTCAAGTGGTGAG
RM1075	2	3.83	200	ACCAGTTCAGTAGTTCACACACC	GTTGGGTTGCTGTGTTGTTC
RM3703	2	3.86	125	AGAGAGAGAGGGAAGGGAAGG	GCTCCCCGACATTTAAACTG
RM5345	2	4.21	207	ACAGATACCCTCGCAAAAAGG	CGGACCCCAAAGAAAGAAAG
RM4355	2	4.26	144	AGGGATGAGAGTAGAAGGCA	ATATGGCAAGCCTAGCG
RM3865	2	4.41	226	AGCAACCATGGACAGTTGAAC	CTCCGACAAGAACTTCCTCG
RM5862	2	6.01	224	ATTAGTACCTCATCATAGCTG	CTCTAATCTTCTCTCATTATCA
RM4702	2	6.22	113	ACAAATCATGCATGTGAAAG	GAGAAGCAACTCTAATCAAAC
RM5897	2	6.73	142	AGGCATCTTCCCCTCTCTCTC	CCAACCCAAACCAGTCTACC
RM12729	2	6.75	200	TGGAGCAGCTCAAGATAGTAGACC	TATTTGGATTCCGTTCCAGACG
RM5340	2	7.48	158	ATGGGCCCTAAGTCATATTGC	ACCCAACGAGATGTACCTCG
RM3505	2	7.54	160	AGATGAGGTGGGACGACGAC	CTTCACAGTGACGAAACCG
RM12811	2	7.77	255	ACAGTACACTAGGACATGGTTACG	GTCTAATTGCAGAATGCAGACC
RM6853	2	8.96	134	ACAACACGCACATCCTGTACC	CTCCAAAGACGAGACCAAGG
RM5699	2	8.98	167	ATCGTTTCGCATATGTTT	ATCGGTAAAAGATGAGCC
RM7288	2	9.03	150	ATTTCTCAACTGAAACAACAT	AGTTTAAGAGCGTTTCTAGG
RM6639	2	9.42	66	AGGCGTGAAATAAACACTCCC	CGGAAGGGATACTTATCAGC
RM6375	2	9.56	160	ATAACGACAAGAGATGCACGTG	CGGGGATAGATCGACAAC
RM1358	2	10.19	180	AGATCGATGCAGCAGCATATG	ACGTGTGGCTGCTTTTGC
RM3501	2	10.19	219	ATCCTAGTGCATCAGCACAGC	GTCCGTTTCAGCAAGCAAAC
RM5791	2	10.75	104	AGAAGCAGAATACGCTTTCGC	ACGTCACAAAGGGTTCTTGC
IDR0237	2	10.81	138	AACCCTTATTCCTGCTCTCCTC	AGAAGAAAGCCATTAGCAGCAG
RM1313	2	11.26	95	ATGTGTCTGAAAACCAAGGGG	CGTCCAAGCTGTTCGTTCTC
RM1234	2	11.34	141	ACACCAGAGTACATAGGCTAGAGG	ACTGCTGATGATCTGGCCAC
RM13039	2	11.67	289	ATGGGAGAGCAATGTAGATAGG	CGTGCTATCCTTATACTTCTGG
RM5619	2	12.01	117	ACAAAAACACAGGAGGACTGCG	ACTTTTTGGGGTCTTGAGGG
RM13084	2	12.94	372	GCATGCTAACTTGACATGACTGC	TACAGTGTGTTGTGCATGTTGG
RM13105	2	13.51	160	GTGGATATTGTTGGAGCCCATACTCC	GGATTGGCCCATGGGAGAGG
RM13106	2	13.52	189	ATGGAGAGATCGGGAAGAGC	GCTGCAACAGGTAGAGAACTGG
IDR0248	2	13.59	116	CCGTCCCTGATATAACCCTTCT	TGACGACAAAGGATATGGCTAA
RM13113	2	13.96	248	CCTTTCCCGCGTCACATAGG	GAGGGCGGTGATGCTTAAATAGG
RM3443	2	14.33	118	AGGCCGCACATGATAGTACC	ACACGCCTGTAGCTCGTTG
RM1037	2	14.59	219	ACAAGGACTATGAGCCCCATC	GGCACCCCAGTCATGGTAG
RM6374	2	15.19	128	ATGAGGACGCTGATTGTCAAC	GCTGCCCCTATTATTTCACC
RM5812	2	15.90	144	ACGCTGACATCTTGCCCTC	GTAGGACCCACGTGTCATCC
IDR0271	2	16.25	150	AAGAAAGAAACAGAAGGGATCG	CGTACGATAGCAATGCTGCTG
RM13228	2	17.10	261	CGGCTGAAACCAAATAGTGTCG	GTGGAACGGAGGGAGTAGTTTGC
RM6942	2	17.44	286	ACTAGAAAAATGCCCGTGCG	GAGACGATCGAACTCCC
RM1211	2	18.46	162	AGGCAGACACGGGTTATCAG	GGAGCCATCACGCATGTTAG

Marker name	Chr.	Position (Mb)[a]	Size (bp)[b]	Primer sequences (5' to 3')	
				Forward	Reverse
RM5578	2	18.64	200	ATATTCTTCTGGTGTGTGCC	CATAGACCACGGTTTGTGTA
RM3858	2	19.19	132	AGGGGACAAGAAACAGCAAAC	AAGCAAGTACTACCACTGCTGC
RM6509	2	19.34	150	AGGTGTTTTGTGGTGTTGTGC	CTCGAACTGCGAGTAGGACC
RM1379	2	20.06	182	ATTGGAGCCGAGGACCAAG	ATGTGCTACTGCTAGTGCTAGC
RM2634	2	20.50	158	AGATTGAAAATTAGAGTTTGCAC	GCCGAGATTTAGTCAACTA
RM5614	2	21.06	108	ACCCTCCTCTCTACTCTCTCCG	CCACCATTCCACCAACTAGC
RM5427	2	21.52	142	ACGCTGCTGTTGACACTTGAC	CCAAATACGAGCTCTCCACC
IDR0301	2	22.05	135	TCAACCAACCAATCAGTGTCTG	ATGCACGCAGATGACACATAAT
RM3688	2	22.40	112	AGTTGAATCAAGCTGTGCAGC	AGCTAGGCAAAGCATGCATG
RM13534	2	22.76	235	AAGGAGTTGGGTCGTGTGAAGG	TACTTCTTTATAGCCCGCTCGTTGC
RM5430	2	22.80	181	ATAAAAACTGAGCCGTGAGCC	ACCATGGGGAGCTGCTTC
RM5363	2	23.00	143	ATCCCTCCCTGGCTTTTTTAC	AGCAACGCGGTGAGAGAC
RM6379	2	23.57	234	ATAAGTCCACTCATGCTGATGC	GATGGCCACCTCTTCTTC
RM3515	2	24.02	124	ACGCTTGTGGTGTTTAATAC	CACTGTGAATACACAGGAAC
RM1920	2	25.47	130	ACAAACACAGTGTTGACAGAA	GCTATTGACTTATCCGTTCA
RM3730	2	26.24	187	ATGCGAGTATCTTCAAGGCAG	ATTGAGGGGGCTAATCATCC
IDR0315	2	26.39	119	ATGATGACTGATGAGCTTTGGA	ACTCGTCTCTCTGCTTTGCTTT
RM1385	2	26.65	177	ATGACAGGTAAGGTGTGGTG	GAACATCATCTTCGAATCC
RM13728	2	26.81	296	AGCCTCGGTTGTCTAGGACTATCTCC	CACTGGTCGCTGAGTTGAGTGG
RM3793	2	26.84	200	ACCCCCCCCTTCTTTTTAAAC	ACGCGATGCCAGAGTATAGC
RM1367	2	27.07	160	AGTGTGTACGTAGGATCGGAG	GCTACTCCTAGCTGCTACC
RM3508	2	27.08	211	ACAGGTAGCAGAGCCAGACG	CTACGGCTAGCAACTGCCTC
RM3512	2	27.32	154	ATCTGATCTCACGAGTCACAT	AAGATAGGGGTGAACACTTG
RM3763	2	27.93	154	ATCTCTGAACACACCCACACC	GTTTTGATCTCAGCTCCCC
RM5631	2	28.27	125	ACGTCCAAGAAATATTGCAGT	GTGAGACAGAATCCTTACGC
RM3212	2	28.87	181	AGACGACAAACACCTGCCTC	CAAACACAAACGCAGCCTC
RM6933	2	29.31	177	AGTAGCAGAAACCAATGCTC	ATTCGCGATAAATATGGACT
RM6290	2	29.88	136	ACAGGAGTGAGTAGCAGAGCG	CATCGGACGGTCACGAAG
RM3421	2	29.92	153	ACAACTCTCCTAAACCTCCCCG	ACCACGTATGTATTGCACGC
RM5472	2	30.64	151	ACACTCAAGACCAGACCTGTACG	CGGCACGTCATTGTAGTGAC
RM13958	2	31.20	283	GAAGCAGGATATACGACGTGTTCG	CCACACGCGCTAGTTTCATGG
RM8029	2	32.49	144	ACTTCATTTCAGAATGTAAGA	ACCCACATTTATTTAGATGT
RM8030	2	32.49	133	ATACATTATGAAACGGATGAA	GCCTAATAACAAAACCACAC
RM7286	2	33.00	134	ACAGAACAATTCGACCGCTTC	GGCTTGAGAGCGTTTGTAGG
RM6636	2	33.08	134	ACTCCAGCGGAAGAATCAGAG	CCCACTCCACTAGCTTGCTC
RM5607	2	33.27	108	AGAGTAGGAGGAGCCGAGGAG	ACGTGGCCAACTGGCTATAC
IDR0343	2	33.60	110	GCCGATGTCTAGATTCGTAGTT	ATCGACCGTAGAACAACCTAGC
RM3789	2	34.69	193	ATTCCCGAATTAAGCAGATATA	CTGTAGACCATTGACTGGTG
RM1255	2	35.26	117	ACATCTGCTTCTGCTAAGCTAGG	CCCAATAAGCAGCTAAGCTC
RM2265	2	35.41	193	ACAACTGACCGTATATTAGCCA	GACCGCCTCTATTATATTG
RM3850	2	35.43	110	ACAAGTTGAGAATGAGGGACAA	CGGAAGTGAAAAGGTAAT
RM7388	2	35.48	164	ATGTCCCTACCTGATTCGGC	GAAGGCACATGCAGTTGAAC
RM6923	2	35.70	161	ATGTTGGAGAGTTTGTGCCTG	AATGTCGGGGGAACGTAAAC
RM6137	2	35.77	168	ACGGCGACTTTGACTCTCC	GACTCCCACTCGTCAACCAC
RM6139	2	35.85	149	AGTGGATCTGAACGCTGCC	GGTAAGCAAGAGGGAGGGAG
IDR0351	2	35.93	119	GCACTGCACAACTCTACCTCAG	TTTGTTTCGAGCTCAATGGTTT
IDR0352	3	0.12	144	CTCAAAGAATGACACCGATGC	GCTGTGCCTATGGAAACATACT
RM3413	3	0.37	218	AGAGGAGATGATGGTGCAACG	GTCCCAATATCCATCTCCC
RM3387	3	0.38	138	ATGTAGCAGCAGCTGGACAAC	GCGAGATCGAGGCAAATAC
RM7332	3	0.41	209	ACACTGTACACCACACTTCAGC	CAGGGAAATGACACTGTCCC
RM4108	3	0.54	142	AGTCCCTCGCTTTATATCTAG	CAACTCTGCTAAACGAATTA
RM14311	3	1.16	219	AGCTTGACTTCACACCCACTCG	GGACGACCTACCGATCAAGACG
RM7072	3	2.57	170	ACTAATCCTATTGATTTAGGG	AGTCTAGTGTCAACCTTCTC
RM6829	3	2.88	148	ACGATAAATGGCAATGCGC	CGATGAAGAGCCAATCCTTC
RM14407	3	3.05	286	GCAGAATATCCTAGCGTCAATGG	TCTTGTAACCTGAAACGGATGG
RM6883	3	3.10	187	ATGCTCCCTGTGCGTACTTTC	GCACATGAGTCTGCTACAAGG
RM6849	3	3.29	125	ACGTCAACTGCATCACCACC	CCGACTGATCATCATCGAC
RM3807	3	3.53	123	ACACACTCTTTTTCAACTCCT	GGCCTATGGCTCTATTATA
RM5474	3	3.80	159	ACAAAGTGTTGGTGAGCATAGC	GTGTTTGGAGAGACGAG
RM3392	3	3.83	164	AGTCCAATGATTCGTTCCCAC	CTTCACCGTTCACCAATTCC
RM4352	3	4.32	99	AGTTGTTGCACCATAGTCAGA	ATACATTCATGAAACCTGCC
RM2791	3	4.57	147	AGCATGAAACTTTGCACTATC	AATAAGGTTTTCTCCACCAG
RM4992	3	4.72	214	ACAGCCTGCTAATTTAGTATT	ACTCGAAATCCTTCTCTATA
RM5442	3	5.53	172	AGGAGACAGGAAAGCCTTCC	CGAGTCGACCAGGCTAGAAC
RM3864	3	5.86	176	AGTCAACCTTGGGGGTAAGG	AGATACTGCCCGTGTCATCC
RM5755	3	5.96	253	AGGAATCGTTGCTCTCGTTTC	CAGACACCGGCCTTAATCAC
RM3467	3	6.00	123	ATAATGGCAGGGTTGTCTCG	CTCGGTGAGCCTCCTACAAC
RM5513	3	6.28	227	AGCTTTCTGGCCAATCTGATG	CCAAACCTGGACATCGACAG
RM5347	3	6.43	202	ATACGTGCGCCGGAATTAC	ATGAGATCGTAGCACTCCGG
RM5477	3	6.55	150	ACACGCATTGTTCGTGATAGG	GCTAATACCGAGCGAGATGG
RM3029	3	6.71	208	ACGGTATAAACGTAAATATTC	GGAGGTACTTTTAAGCTAGT
RM3872	3	6.85	181	AGGAAGAAAGGATCTATATCA	ACGATTTGTTTAAGTTCAA
RM1022	3	7.23	151	ACATGGGATGAGGGAGTAATG	CTTTGATAGCGGCTTTGTCC
RM3134	3	7.24	178	AGCAGGCACAAAAGCAAAGAG	AGGTGAAGGTGCATTGTGTG
RM5639	3	8.20	124	AGGAAGAACAGAGTTGCTCGG	GTGCCATTTATTTCCGTCCC
RM1338	3	8.39	143	AGAGGGAATTAGATTGGATT	GGTCCACTTCTTCCTTCTAT
RM4996	3	8.48	190	ATATTGCACTATATCACACCC	GTGTTTTATACCTTGAAGCC
RM5896	3	8.55	141	GTAGCAGCTGCCAAGGTGAAGC	AACCCAACCCAAACCAGTCTACC

Marker name	Chr.	Position (Mb)[a]	Size (bp)[b]	Primer sequences (5' to 3') Forward	Reverse
RM6783	3	9.32	127	ATGCTGGTCTGGTCCTTCAC	GGTTGTTGGTGGTTTGTTGG
RM7197	3	9.89	159	ACAACGTGGGAATTTCTAGCCC	GTTTTGGGCCTAAACGAGTG
RM1371	3	9.94	174	AGAGAAACGTTCCTTTCGCAG	ACGATGGCATCGTCTATCG
RM14778	3	10.05	187	GTCACAAGCCACTATTAACAGCTTCG	AGATTAGCGCCCATTCTCATGC
RM4321	3	10.17	189	AGAAGGAAAGGTGATGATGA	CCAACGTGACGTTTATAACA
RM1284	3	10.62	151	ACACTCCCCTTTTGTAAAGC	CTAGAACTACGGCATTTTCG
RM5178	3	10.66	161	ACGTGGTACCAATTGTGTAGT	GTTTATAAAGCTAGCCGGAC
RM3434	3	10.78	149	AGAAATGGGATGGGAGAAGG	AAAGGGAAAGCAAGAGGAGC
RM3562	3	11.68	151	AGCACTCGTCTTTCGCTTCTC	AGGATTCCACAAAGGGGG
RM5748	3	12.33	167	ACAGTTGGCAATTGTCACGAG	CGAACATATCCAAGCCTCC
RM5551	3	12.93	118	AGACTAGTCCGGCCGTACATG	AGTTTCTTGTGTGACGCGTG
RM6929	3	12.93	192	AGTTGAACATATTGTGGGGGC	GGTTCTGGACTACATCGAACG
RM5489	3	13.31	184	ATGCTGCGTGTTAATGCTAGG	ATCATAGCCTCAGCCCTAAG
RM3291	3	13.60	185	ACCTGCACAACAACAACAACC	GTCTGTCCATCGATCCATG
RM6080	3	13.93	128	ACAGAGGAAGCAAGGAGATCG	CCATCGGGAGAGAAAGAGAG
RM2917	3	14.39	131	ACGGATCACCATGCTAGTTAT	ACACCCAAAGTTGAAGCTAG
RM6676	3	14.49	147	AGTTCACGGTCCAATAAGAAT	CTTTCAAGCTTACGAAAACA
RM6959	3	14.51	106	ATCCTATGGAGGATTGTTGCC	CGGAGGAGCAGAACAAAAAC
RM3204	3	14.99	94	AGCAACCCTTTCTTCCTCCTC	CCAAGGAGAGCGCACTAGC
IDR0421	3	15.87	117	GTATGCAGCATCAGTCCACAGT	TTAAGCAGTGATGCTCCACAAG
GS3 UD001 3[c]	3	16.73	91		
RM6881	3	16.88	78	ACAAGGCACCTCCTCCTCCTAC	AAGCAGAGGAAGACGACGAC
RM3400	3	17.27	185	ATCTCTCTCCTCTCTCGCTCG	AAAACCGAAGTGCTCTCGC
RM6914	3	17.29	226	ACTCATCGTCATCCTGTGCAC	GAACTGAAGAACCCACCTGC
RM1334	3	17.42	179	AGCATCTACTGCCAGTAACACTG	ACTCTTCTGCGGCGTGTG
IDR0429	3	18.07	148	GAAAGAAGGGAGAGAGAAAGAGAA	CGACAATGCCGGACTCTAA
RM1940	3	18.16	176	ACTATCGATCAAAATGCTAG	AAACGAATGGTTAAATGTTA
RM5684	3	18.41	141	ATTCACCACATCGAGGATCTG	ATTCTGACGCACCAGTACCC
RM7431	3	19.52	145	ACGGGACTTCTCATAGGCATG	ACTACTGGCTTCCCACAACG
RM2453	3	20.25	161	ATAGGTGTTCAGGAGTAAAGA	AAACCAGTATTGCTTACAAG
RM7370	3	21.15	112	AGTAGCTGCATCTTGCAGTGC	CATCGGGCATAGTATGCATG
RM5488	3	21.39	138	ACTCCCTCTTCCTCTGTGTGC	CTCAGAGGAACAGCTGGGTC
RM3698	3	21.78	177	ACAGTTCCGTTACAACCTAGTCG	AATCTCCAGCTGCTGTAGCG
RM3646	3	22.00	139	ACTAGAGCACCCTCGCTGAG	CTCAGCCACCCCATCAAC
RM15381	3	22.15	180	AAGGTAGAAGGGAAGGGAAGG	ACATCTTGGCATGTATGGATGG
RM6832	3	22.40	182	AGTTGTAAATGCCTGAGTGC	AAAGAGCTAAACCGCTAGG
RM6266	3	23.82	160	ACCGTCACCTTCTTGAGAAGC	GACATCGAGAGCGAGGACAG
IDR0460	3	24.17	143	ACCCTAGCGTGAAGAGCCTACT	AAGGGATACTGATCACCTCGAC
RM3513	3	25.11	145	ATACTCCTATCCTGCCATGGC	GTAGTAGACGAGAGGCCGG
RM15630	3	26.08	286	AACTCGAAGGATCTCGCCCAACC	ACCCACCTCCTCACGCTGTACG
RM2334	3	26.75	155	ACATGCATCTGATCTGATTAT	GTGAAGAGTACAAGTAGGG
RM1292	3	27.33	257	AGTAGCAGTTTTGGAGTTCGG	GTTATCCGGAGACGAAAAGG
RM2614	3	27.43	176	ATGGCACAAATCATTATGATC	GATTGCAATGCAGCATATAG
RM1350	3	28.68	168	ACGCCCTAGTAGATAGGTAATTG	AAATCAGCAAGAAAGCTCTG
RM3856	3	28.78	140	ATTGCATAACAAGATGAACAA	AATTGCGGATTTTTTATT
IDR0489	3	29.99	150	TACTCACATCTCTCCCATCGTG	TTAGTGGCCATGGTTCAACTTT
RM3525	3	30.39	179	ACACTCTCAGCTCATCAAGACC	GGGCAAGTGGTCAAATCTTG
RM5475	3	30.58	227	AGTCTGTACTGAGCTTTCTTGGC	CACGGCCATAAGGAGAAC
RM5813	3	30.98	146	AGCAGCCCTAGCAATTCAGTC	CTCCCTTTCCCTCCACCAC
RM3867	3	31.75	120	ATTGACTGGAACATCGAGCTC	ATCCCCTCTACACCGTACCC
RM6817	3	31.93	131	ACTTGACGTGTTGAGCTGAGC	CACACCTACGCAGCTGATC
RM1238	3	32.08	134	ACTGTAATTGGGGTGGTGGTC	GAAAGAAGAATGGCAGCAGC
RM1352	3	32.35	209	AGTTGTACTCTGGTTGCTTCCG	ATCTTGCTGCAAGACGAGG
RM6970	3	32.44	116	ATCGCTTGTGTTTTCTGGGTC	GGAGAATTTGGAGGCTGC
RM3564	3	33.43	190	ACATAAACCGCTCGGCATTG	CTGACTCACAAGACAACAGGG
RM7076	3	33.66	199	AGAGCAGGGTCCATGAAATTC	CGATGCCTCGATGTATGTG
RM1373	3	33.72	150	AGTTGGATATATAATGCAGG	CATGCTATGTTTTCATGATA
RM6135	3	35.26	141	ACACAGGTTTTAGCACCGGAC	AGGAGGAGGCAGTAAGGCTC
RM3329	3	35.60	120	AGCACATACAGAAATGGTGAA	GGCAAGGGACATGTAGTAAC
RM4404	3	35.60	105	ACAGTGTCAAGCTAAACAGGT	AATGTGATGAGGATTTTGAG
RM1221	3	35.68	186	AGAGTAGAGAGAGATGGCGGC	AGGATTAGCAGCGTTAAGCG
RM7389	3	36.16	112	AGCGACGGATGCATGATC	GAGCCGGAGGTAGTCTTG
RM7324	3	36.35	206	AGAGAGAGAGAGAGGAGAGGCG	GATGCACATCTCGACAGCTC
IDR0514	3	36.41	124	ATAAAGGTCGCACCCTACAGAA	CTAGGGACGGGAGCGACAC
RM16252	4	0.06	186	ATGGTGTGGGATCAGATCAAAGC	TCTCGATCATCTCCTCGTTAAACC
ID15-45	4	0.10	243	CTTCGGTTGCAAGATGAACG	CCCAACCAACCCAAGTCCAT
RM16254	4	0.11	175	CTGTGATTGCGTGGATGACTCG	GAGTGCAGCACGAATCCTAAAGC
RM16284	4	0.52	292	ACACTGCTCGGGAGTTACTGACC	CAATAAGCAGGGCTACAACATGC
RM1867	4	0.92	177	ATACTTGGGTATCAGTCCTTG	CGATCACCTGAATTTAAGAA
IDR0518	4	1.01	96	CAGGATATGTAGCGACCAAACA	CCAGATTGGAGATGAAGAGGTC
RM7535	4	1.17	125	AGACGAAAACCGGTCGAATTC	CCAACAAGAGTGAGCATGC
RM5414	4	2.03	116	ACCATGGTTCAAGAGTGAAA	ACAGCTCAACCTGTTGAGTG
RM16354	4	2.06	259	ACAAGTGATCACAATGGCTCTCC	CTTATGCTCTCATGGTAGTGTGTTGC
RM16367	4	2.41	281	GTTCCCAACCGGAACAGTACG	CCCTTAGGCTGTGTTTGACATCC
IDR0531	4	3.65	150	TGTGTAAAGGAAGATGGCATTG	GACGAGTGCACAAGTGAAACAT
RM7200	4	4.04	177	ATCGATGGTGACGATGATACG	ACACAACAAAGGGATGGTCC
RM16434	4	4.67	153	AGGCCATCCAACAAACTAGTCACC	GTCAGCTATTTAGGCTCCACTTGC
RM16449	4	4.91	202	TCGCATGCTAACTTGAGACG	CAGGAACATGTGTGCAATCG

Marker name	Chr.	Position (Mb)[a]	Size (bp)[b]	Primer sequences (5' to 3') Forward	Reverse
RM3658	4	5.61	165	AGGCGAGGGTACGGATCTC	CTCTCTCCTCCTTCCCC
RM1305	4	5.66	117	ACCTGCATCGTATGTGTGTG	CTGGGGTAAACACTGGGAG
IDR0541	4	5.99	140	TGATCATGGATGTGTGGAGATT	GCAGCTTATTCTCAGGATTAGGG
RM16507	4	6.94	296	TTCACATCCATTCAGCGACATGC	GGGTGCTACATTCAGCATCAATCC
RM7472	4	7.10	112	AGCCACGTGACGGTTTAAGAG	CAAGTGGCACGTATGAGTCC
RM7504	4	7.25	231	AGGCTCTGTTTCTGAATTCGG	ACGTGGCAGCTTGAGAGC
RM16534	4	8.04	215	GGAGCGAGGACGAGGAGTATGG	TGCCCGAGAATTGTGTAACCTTCG
RM16540	4	8.22	209	CGGTAGACATCCAACATATTCC	ACCACATGGGATCTTATTGC
RM16554	4	8.68	129	GCAACCAAAGTTGGTAACGAGAGC	CCGGCGCAATCTATTAGACACC
RM5953	4	9.39	127	ACAACTTTCTGTGATGGTATC	ATCCTTGTCTAGAATTGACA
IDR0561	4	9.72	106	TAGAATGTGCATTCAAGGGAAA	TTTGGACGGTAAACCTTTATTA
RM16606	4	11.57	276	TGCACTTCTTTAGAGTAGGAGGAAGC	CATGCATGTGTCCAAAGATTCG
RM16616	4	12.05	200	TAGGCACTGGGCTCAAGTAGTGG	TTCTCATCCTTCGCCATCTAACC
RM16621	4	12.36	237	TTTGGGTGCAGGTGCACTTTGG	CTGTCGGTCCCATACACAAACAACC
RM5633	4	13.08	211	AGTGTAGCTGCTAGGCCGAAC	CCTTTCGCTACGTTGGAC
IDR0579	4	13.62	140	TTATTATTGCAAGTTACTCCCTTC	CATCATAAATTTCCAGGCCATA
RM5893	4	13.77	406	ATTCTACGTTTGACCGGCATC	GGTGGCTTGAGAGTGTTTG
RM7279	4	13.77	203	ATGAAGGAGATACACCGAAAC	GGCTTGAGAGCGTTTGTAG
RM16670	4	13.88	177	TGAACTCGAGTGAAAGATGAGG	CTACAAGGAAACCTCCCTATTTGG
RM1236	4	15.35	113	AGCATCCAACGAGGTACAAG	GGAGTGCTAGGGATGTCGAC
IDR0596	4	16.34	133	AAAGTGTCCCAGTCCATTCATC	GGAGCACAATTGCTCTGATGT
RM16752	4	16.90	297	TGTGAGGAGTGCCACATAGATACG	GCTTAGATAGGGAACTTGTGGTTTGC
RM16753	4	16.92	80	AGCTGCAACGACCAACTTTATGG	CCCGGGAGTACATAATAGCCTTCC
RM16757	4	17.07	268	AACGGAAATGGCTCATTGAAGC	GGCATGAGGGCGTTTGTAGG
RM16761	4	17.10	289	GTCCTCCCAACAACGCTCTCC	ACCCTGTTAAACGGCTGAGTCC
RM16791	4	18.12	280	GGCTGCTTCAGAGCGTTTGTAGG	AAATACGCCATCAAGTTGCACACC
IDR0606	4	18.14	91	GGGATGTATCCCTGAAAGAAAG	TGATGACAAATGCCTCTATTGC
RM16828	4	18.79	286	GCGCCTATAGAGAGGAGATAAGC	TTGGGTATACCAACCATTCACC
RM3308	4	19.18	147	ATACTTTTCTCTCCCCCCTCG	GGGAGGAGATTGGTTGGC
RM16855	4	19.22	370	GCGAGCCTAGTTCTGATTCTAGC	TGGAGTACGTATGTTGGGTATGC
RM16868	4	19.46	199	ATATTAGAGCATCTCCGACAGC	AATCTTGGAGCCCATACTCC
IDR0612	4	19.48	89	TATTGGGAATAAATGGCAGAGC	GTAAATTATGGGAGGCAACCAA
RM5586	4	19.90	135	ACTCCATAATCAAGGAAGCTA	ATGAGTTCTTTCGTCAGTGT
RM6679	4	19.90	141	ATTTAGGCCGTAAGAGCGAAC	GAATTTGAGTAGCTGGCTCC
RM3742	4	19.92	76	ACTCTTCATCCCCCAAGCC	GAGAAGAAGAACAGAGCTGCG
RM1359	4	20.03	135	ACTCGCGAGGAAGAAGACAAC	CGCCGGCTGGTTAATTAATC
RM5979	4	20.77	172	ATGCTGGACCTCACTGTTCTG	ACGTGGCTCAATCAGGAAAC
RM3708	4	20.83	160	ACAAGCGTAAGACGACGAGACC	CATTTGGGCTGCACC
RM6997	4	21.27	156	ACAACGCGGCAGTAAATTTGC	GGCCTTGTCAGTCTACATGC
RM5757	4	21.42	170	ACCTGAGACCATATGCTGCTG	GAGGGAGCATCATTAGCTGG
RM3524	4	22.89	130	ACGGAGCTGGTCTAGCCATC	GTCTCCGTCTTCCTCACTCG
RM3042	4	23.02	182	ACAAAAAGGAATCAATGTGAA	GGCTGTTGAGAGGTAGAGAA
RM2521	4	23.22	183	ATACGACTGCCTACATGATAT	GTTGCCAGTTTTTTTATGTC
RM1165	4	23.84	135	ACTAGGACGAAGGGGAAGGAC	ACCACCACCATTCCAAACTC
RM3839	4	24.09	219	ACAATGGGACCAGAAAGCACAC	AAAAAGAGCATGGGGGCTAC
RM3785	4	24.25	138	ACCTTTTCTTGGCTTGAGGG	GCTTTTGCTACTTTTGGGGG
RM3367	4	24.28	129	AGGATCCATCCATCCACTGAC	GGATATGTGCTGCTGTGTGC
RM1388	4	25.22	239	ATTCAATGAGGCAAAGGTAAG	ATTGTAGCTTGGACTAGGGG
IDR0636	4	25.83	137	TCAGACCATCTAAGCAAATGAAA	GCTGATGACCATAGAAGGGCTA
RM1354	4	26.30	149	ACACGTAAATAATACCCGGCG	AAGGTATCTGTCGTGGTGGG
RM5714	4	27.45	245	CGATTTGAGCCGATACATTAGGG	TGTTGGTGTCGGATTTCTCAACC
RM3820	4	27.81	122	ACTCTGCTAGCCTGCACACAG	GTGGCTTTCAATGGTTGGAG
RM2441	4	28.05	141	ACCATGTGAAAATTCAC	ATTAACAGATGATGCAAATC
RM3781	4	28.76	146	ATTTAGGGTTGGTGCTGCAG	AGTTGGGCAACAGATCCATC
RM3916	4	28.76	228	ACGATGTAGGTAGAGGGACACC	GTCGTACGTCCAGATCTAGTCG
RM6089	4	29.59	170	ACCACCGAATCGAATAACCAC	ATGGCCAGCGTGATCTCC
RM1703	4	29.61	208	ACATACCGTATGTATGATGTA	GTGTAAAGCTTAAAATCAAG
RM6365	4	29.83	193	AGCTTGCATGGAGGAGAGAAC	GGAAGAACATCTCAAGCCC
RM6480	4	29.88	121	ACTGCGGGTACGTGAAGAAC	GAGCCCAAGTGTGATGCC
RM3474	4	29.89	174	ATTGGTTGCTTCCTCCCATAC	GGCATTGTACGACGGATCTC
RM7208	4	30.14	179	ATTATTAGCGCGCCACGTG	ACCAGGTTATTGTTCCAGGC
RM5503	4	30.36	192	AGGGTTTTGGAAGGGAAGAAG	ACCCCTAGCATAGGCCTCC
RM6748	4	30.87	159	ATTGGGTTTCTCATATTATG	CCAACACTCCTAACTAGTTC
RM5511	4	31.11	227	AGAGCTCCCGACAGAAAACAC	ACATCGAGGTGAGCGAGC
RM3843	4	31.68	172	ACCCTACTCCCAACAGTCCC	GGGGTCGTACGCTCATGTC
RM6629	4	31.74	86	ATAAACGGTGTGCAGCTTCTG	ATTATGGGCGGTCGCTAAC
RM3836	4	31.81	126	ACTGTGGAGTACAGGTCGGC	GAAACGGAAACGAAACCCTC
RM6909	4	32.06	168	AAGTACTCTCCCGTTTCAAA	CCTCCCATAAAAATCTTGTC
RM1153	4	33.03	114	ACCAACGCCAAAAGCTACTG	ACTCGCCCTGCATGAGC
RM3648	4	33.31	186	ATACCCTTTCTTCCCCAAACC	ACCTCCTCCTCCACTTCTCC
RM5478	4	33.48	130	ATCCAATGCGATGCTACTCC	CATCACGAGACCACGACAAG
RM2799	4	34.32	157	ACTCCGTTTCAGGATATAAGA	AGATGTTAATGAATTCAGGC
RM17632	4	34.35	268	ACAGCATGCGCACCACATAAAGG	CGTGGTTCACACACTTACATTGTTGG
RM6006	4	34.89	134	ACTCGGCGATGAACAGCTC	AGAAGATCATGAAGCGGTCG
RM3332	4	34.90	115	ATTCTCCTCGCTTCCTCCTC	AAAGAGAGAGCCGAGCACTG
RM1841	4	35.10	141	ATTGGAATACCTAAAAGCTA	GGATGCCTTATATGAAATAT
RM2431	4	35.10	131	AGATGCCTTATATGAAATATC	CCTAAAAGCTATTCTGTATG
RM17686	4	35.28	228	GAACGAAGTGAACAAGCCAATCC	CCCATTACGGCTTAGGCTCAGG

Marker name	Chr.	Position (Mb)[a]	Size (bp)[b]	Primer sequences (5' to 3') Forward	Reverse
RM1272	4	35.30	135	ATCTATGGATCTGCATGCTGG	CTGCCCTGTCCTTTTAATCG
RM5608	4	35.36	149	AGTATCTTTGATCGCGCGC	ACTGGTAGAGAGCCCTGCTG
RM7030	4	35.38	78	ATGTGAAAATCCTGGTCCCAG	CTGGCATCAACATGATCAGG
RM1248	5	0.09	136	ACAAGCAGCTAATGGTTGGG	GTGATTTTGGCTCAGGTCAG
RM3529	5	0.11	148	ACGCGCCACCTCGATATATAC	GCTCAGGTTAACCAAGGTGG
RM17726	5	0.17	179	AGCATTATCCCTCCCACACAAGG	CGGTTTCTCTCTCCCTTCC
RM5816	5	0.28	181	ACTGCATGCATGACTGCATC	GACTCTGATCGATCCATGG
RM4777	5	0.45	152	AGGCCCTATCGTACATATTTG	AGCATTTGTTTTTAGATCGC
RM5693	5	0.46	200	ACTCTTTGTGTCTACAAAAAC	GGTTATTTTAATAAGATGC
RM6320	5	0.47	164	AGAGCTGGACCTCCTCGACAC	CATGCATCACCGAATGAGTC
RM2010	5	1.18	118	ATCTTCTAGGAAATCGAGGA	GTTGGCAACTTGTAGTCTTG
RM5579	5	1.92	137	ACAAATATTGGCAAATAAACT	ATATTGCCTCATGGTAATAA
RM2488	5	2.15	161	ATGCTGTTTATGTGTCTACTC	GAATTTTGTACAGTGTGTTG
RM17867	5	2.22	176	CATCGATAGAGCCAGATAGATGC	TTTCTACAGGAAGTCAGGAACG
RM6517	5	2.91	182	ATTCTTCCTCCTTTTCCCTCG	ATTGGTCAGATCGAACCTGG
RM1366	5	2.92	157	ATTTGAGGGAGTGAAAGGGG	CCCTTTTGCTCTCTGATGC
IDR0688	5	3.31	119	AGGGAAGGAGGAGCAGAGAC	ATGGAGTTCTGGAAGCAGCTC
IDR1751	5	3.50	154	TTCTAGCGTTCTTGCCTTCTCGG	GCCTTCATTCTGTCTGATCTCTTCTGCC
RM3777	5	4.17	172	ATGCTTATGTCGCAAGACTAG	ATCTCACCTCTTCCCTACAA
RM3322	5	4.26	122	ACTTCTCCACCCATGCCAC	CCTGCAACGAACACCCAC
RM3193	5	4.97	126	ATAACGCCTATATTAACGCGCC	CGACGTGGAGGAGAGAAGAG
IDR0697	5	5.03	122	TTCAGTTCAGTTCAGGGCAGT	GAGCTGACTTAGCTGAATTTCCA
RM5541	5	5.21	146	ACCGGGACTAAAGATCGGTG	ACACATATGCGCACGCG
RM1089	5	5.36	240	ACAGAAGGATTATCTCGATACC	AATAGGGCTTGAAATAAATTG
IDR1752	5	5.45	142	TTGTCCCTGTTCGCCCCAGATG	AGAAGTGTGTCCTGGATGTGGCG
IDR0702	5	5.79	117	CCTACCATACCGACATACTCCA	AGCCTGGTTGGATAGCTGTAAA
RM18055	5	5.88	296	AGATCTCCTCTCAGAGTCTACCG	CACTGAGTATAATCCCTGCAACC
RM7118	5	6.09	97	ACGTTGGGTATCTGACGAGG	ATCTGCACGCGTAGCTAAGC
RM18107	5	6.83	295	CGTATGGACTTGCCTTGAGTCG	TCCAATCTGCCAAGCTTTACACC
RM2422	5	7.02	123	ACAACATGGGAAACACTAATAA	AAGATTTGAACCACAGTAGA
RM3683	5	7.18	111	AGAAGGCTCGCCGTAGCTC	CTATCGATCCAATCCAACGC
RM2998	5	7.24	196	AGTCTAGAGCTAGAAAGAAAT	GTAGTACTGACAAATATGGG
RM2744	5	8.30	157	ATGGCACTGTAACTAAACAC	ACTTACAACAATTGGGATC
RM6082	5	8.90	147	ACAACCCTAGAATCGGCGCTG	CACCGATGACAACGAGGAC
RM5844	5	9.15	195	ATGACTAACGTGGCATCCATG	GCTAGGAGCCATTGTCGAAG
RM18194	5	9.35	296	CCTCTTCCAATGTTCTCAAGATCG	TATTTCCACGGACAAGAGTAGGG
RM18239	5	10.67	194	TCTTGTTTCAAGCCCGCTATTTGC	ATCGGCGCCGTATAGAATTCAGC
RM8039	5	13.43	256	ACGTACGTACTTATATCTCAT	AAATCTAATGTATCTGAGGT
RM2676	5	13.54	151	ATACTCTTCTGGATGTGTGTA	CATAACAGACAAATGAAAAC
RM6229	5	13.61	101	ACGAGGGGATTCGATCGAC	ATCCCTTCACACTGCTCCAC
RM6724	5	13.83	136	ACCGAGGATAGGGAAATTGTC	ATACATGCACACGCCATCC
RM18353	5	14.01	287	AGATCTCACTATTGAGTAGCCCATGC	CACCTTGCCCTTAAATACCAACC
RM6742	5	14.88	178	ACATCCGATACTTCCTCCGAG	CGAACGTGATATCTCCGTCC
RM3838	5	16.55	198	AGATGTTGCCAGTTTGCCTG	AGTGTCTTTGTGCAAGCCG
RM18465	5	16.62	192	TCGACGCGTTACCATCCTCAGC	CTCTCCACCACATCGCTCTCTCC
RM18467	5	16.63	155	GACATTGCCTACAGGATCACAGG	CACCATGGAGCCCTATCTGG
IDR0755	5	16.74	148	TCGTGATCTGAAATTTATTCTCTT	CATGTGCAAACGTGTGATTAAA
RM6024	5	17.81	180	ACATTCGTCCAGGGATTCAC	GTGGTTGCTCACCTCTTG
RM1237	5	18.02	144	ACTCCGCGAGCTTTAGAAGAG	CACATACTCTGGCTCTCCCG
RM1868	5	18.46	124	ATGGATACTTCCACTTCCAC	CACACATGTGCAAGATGTAC
RM2185	5	18.53	162	AGTAAGAATGCAAATGTGCT	ACTTTTTGTTTGTGGATGTG
RM6621	5	18.84	155	ATCCCTGATCAAGAACGGATC	ACTCACTCACCCACCCACAC
RM3969	5	19.00	142	AGGCTAAGTTCATATCCAAC	ACCCTATAAAGCATAAAGGA
IDR0769	5	19.98	120	TGTGTAGGACCTGATGATGGAG	TGCTAATGTGCCCTTATTCTCA
RM1386	5	20.06	197	ATATTCAAGGTTGGCTGCCTC	CAATCACTCCATGGCTGATG
RM5329	5	20.09	116	ACTCCTCCTCTGCAGGTGAAG	CATCTCCATTTCTCACACGC
RM3351	5	20.76	134	ATGGAAGGAATGGAGGTGA	GAAACGTAGCCAGGCAATG
RM3663	5	21.43	126	ACATCAACCTCCACGAACATG	CTCGGTGGTGATCCTCCTC
RM3800	5	21.45	134	AGAATGAACCATTCCAAATGA	CCTGGAAGTGCTGTATGATC
RM4501	5	22.11	114	AGCACAAATGTCTCTGTCTAA	AGAGTACGAAACGGTACAAG
RM3295	5	22.33	95	ATCGTGTCATGCGATCGAC	GCTTCGACTCGACCAAGATC
RM3870	5	22.96	193	ATACATCTCCGGCGTTTACAC	CCAAGGTTGAAACAGGAAGC
RM5575	5	23.41	140	AGGCAAGGCAGAAGAACAAAC	ATTGTGTGGCTGCTGCTAGG
RM3476	5	23.91	132	AGATTCTCGTCGTAATCAAGA	ATCCACGGTTAAGATAAATG
RM6598	5	24.13	182	AGCTAAAACAAGGCCGCTAAG	AGTTAGTTAGCCGAGGCGC
RM18926	5	24.48	249	CTACCTCTCCGTCCGCATCC	AAGAGGGACTCAGAGAGCACTTCC
RM3348	5	25.15	158	ATACAGCCACTATGCTGAGCG	CTAATTCAAGCCCTGATCGG
RM18959	5	25.23	260	GAGCTATTCCATTACAGGTAGTGAGG	GGGTGAGATATGGGATAAGAACG
IDR0793	5	26.02	123	TAACGAGCTGACATTTCAACGA	GGCTTGCTATAGGGCAATCTTT
RM3616	5	26.34	136	AGTGCGGATTTCTCCTCTCTC	GCCGGTCCATTTCTAGAAG
RM6400	5	26.55	184	AGTACGAGAGCGACCTCGAC	CGTCGTCGTCGTACACCTC
RM3809	5	26.65	135	ACAGAGAAGAACATGGGGAGG	ACGAGGAAACGCAGAGTGAG
RM7452	5	26.99	94	AGAGGCCATGAACGGTCAC	ACCCAATTATGGTAGCGTGC
RM19062	5	27.30	188	CCTCTAGCAGCCTGAAATGAGC	GCACAGATGGGTTTCAAGAAGG
IDR0801	5	27.49	116	AATCTGTATCTAGGCCAACTCA	GCGACATGCATTTGTGTAGACT
RM5784	5	27.86	139	AGAACGCACAAACGTCCATTC	CACTCCAGTTCCTCCACC
RM6360	5	28.14	91	ACAACAGATAAGTGGCCGAGAT	AACGTGGAATCCAAATTGAC
RM6339	5	28.15	188	ATCGAATGTGGGTACTAGGTAGG	ATCCGTTGTGCTAGCTACGC

Marker name	Chr.	Position (Mb)[a]	Size (bp)[b]	Primer sequences (5' to 3') Forward	Reverse
RM6313	5	28.34	107	ATCCAGATCCACTTTGACCG	GGAGGACTTCTACCATCCTTG
IDR0806	5	28.97	121	GAGCTTTGCAGTTAACCTCGTT	CTCGGAGCATGTACTGACGTA
RM1054	5	29.23	155	ATGCATATGTACCGCAACCTC	CTGCATGATCCCCTCTG
RM3286	5	29.54	163	AGTACCCTGCCACGGTACAG	CATGCTGAAGTAAAACCGGG
RM5907	5	29.61	190	ATGCTGTCTCCACTTCCCTTC	AAGGAGGCGTGCTTAACAGG
RM6273	6	0.13	199	ATGCCTTCGCACTCCAGTC	GACGAGAAGATCCTTGTCGG
RM6775	6	0.21	193	AGCAGATCAAGTATGCCTGCC	CGCTAGATAGGGGATGTGG
RM6467	6	0.22	114	AGGCAATCTCTCCGAATCTTC	CTAGCTGCTCTGCTCTGCTG
RM7158	6	0.22	197	AGTTAGCAGCGTACCTGGAGC	ATCCTCCCCTCGATTTCATC
RM8107	6	0.48	146	ATTGACCTGATGTATGTAATATATCAAG	AGAACAAGAAAGCCTATCACTATATATC
RM8120	6	0.48	157	ACAAGATGAGTAAGTTTAATTGACCTGAT	GAAAGCCTATCACTATATATCTAACTAAGC
RM4332	6	0.72	181	ACAGATTGTGAAAATGACAAA	GCTGAATACTGTCCATAAGC
RM5199	6	2.27	118	ACAATTCATCATATGATGATCG	CTCTGGTCAAACTAGGTGAT
RM3805	6	2.85	110	AGAGGAAGAAGCCAAGGAGG	CATCAACGTACCAACCATGG
RM8125	6	3.17	165	ACTCGTACCATCGGCTGT	AACTTACTGTGACTGACTTGGTCA
RM1163	6	4.19	130	ATCTAGGGTTAGGGTTTCGCC	AGGTCGGTTTCCTTTTGTCC
RM4608	6	4.30	137	ACAGGTAATAGTCATACTCCT	GGAAACTAGATTAGCTCATA
RM8258	6	4.74	182	ACCCTCTATCTGTGCATCAA	CTCGACTAAACTCGAAATCCT
RM5754	6	5.23	187	AGCGTCTTTGACACACAATGG	GTGCCCATGTTGGTAGTTTG
IDR0829	6	5.43	144	CTGACACGTTTAGCTGCGTAGT	TCAATGGTCGTATCTTCTCCCTA
RM2126	6	5.91	172	ACAGATTCAGGCCTTGTTTA	CTTGCAGTTTACAGACGAA
RM2615	6	5.96	166	ACAGAGTGCTTTAGACAATCA	AAATTGGTAAGAGATTCTGC
RM6359	6	6.44	177	ACCGAATCCCACTCTTCCTC	AGGAGGAGGAGGATGAGCTC
RM6857	6	6.57	163	AGGCTTTGGCAACTTGTCTTC	GAGGAAAAGATGAGGGAAGC
RM4128	6	6.65	142	AGTAACTCGATCAAACTAAC	AGAGTCCATATAGAATTTCA
RM5855	6	7.41	230	ACAACTAGCAAGCTGTAGGCCG	CGGTCGTGAAAAGCGTAAG
RM19725	6	8.12	255	GATCCGAAGATGTGCCAGACAGG	GCCATGGCGAGGAGATTTGG
RM3431	6	8.75	143	ACCAATGGTGCAGTTAACGTG	AACATTCTGGAAGACACGGG
RM5963	6	8.82	87	ACTGCCTAGCTTCCGTTTCTC	AGTTACGGGAAATGTGTGGC
RM2523	6	8.92	147	ACCGTAGGTCTTCAAGTGATA	GCAAAACCGAACTAAAATTA
RM6836	6	9.31	171	ATTGTTGTATACCTCATCGAC	AGGGTAAGACGTTTAACTTG
RM19807	6	9.86	106	CGGTTTGTACGTAAGTAGCATCAGG	TCCAATGCCAACAGCTATACTCG
RM7213	6	10.61	212	ACTATAGCCAGCGACGAGGAC	CTGCACCCATCTCTCTCTCC
IDR0854	6	11.35	126	AAAGCACAACCACCATGACC	GGTTGCTAAGAAGACTGGGCTA
RM19863	6	11.45	274	ATGATTTCACTTGGCGGATGG	ACCATCACGACAGTACGATAGGG
RM19875	6	11.74	251	CTAATGAACCGCTCCGTTTACC	TGTGCTCTACTTGTTGTCACATGC
RM19890	6	11.95	249	TTTAGCATGGGCATCGATTTGC	GCAAAGGTGGGATCAAGATTGG
RM19902	6	12.09	232	ACGAGTGCAGACACAATCACATGC	CACATCAAAGACTAATGCGGAAGACC
IDR0858	6	12.25	114	GTGGGTCTTCAAAGCTCTCACT	ATGCATGTTTGTGTGTGCTAAA
RM3183	6	12.45	141	AGCTCCACAGAAAAGCAAAGC	GCAACAGTAGCTGTAGCCG
IDR0863	6	13.49	145	TGTCATATGTGATTCCTTGGCTA	TACGTGTGTTGTACATGCGTGA
RM19980	6	13.69	269	AAAGGACAAGAGAGGTCAGTGG	AAGGTTCACCTAGAGGACCATACC
RM1161	6	13.75	81	ACAAACTGTTTTACCCCTGGCC	ATCCCCTTCTGCGGTAAAAC
RM19985	6	13.89	192	AGCAGATATCACACACAGCATTGG	GGAGCTTCATTTGTGATGAACCTAGC
RM20023	6	15.84	203	CTGACCTGACGGCTGACATGACC	CAAGCAACCTTTCGGGATTTGC
RM20039	6	16.04	173	GCTTGCTGCCTGTTAATACACTATGC	GCTTGGTAGTTGGGCTGATGG
RM20045	6	16.05	188	CACATGCATTCGGTGATTTGG	TGTGTGGCGATGGGTAGTGC
RM20046	6	16.09	198	AACGAGCGGCTGAGATTTATGG	ATGTACCAGCCCTTTGAGAGTGG
RM6818	6	16.58	131	AGTCGCATTCGTCTCCACC	ACCATTTCCAGATGACTCGG
RM5087	6	16.68	163	ACAAGGAGTTAGTGGGGGATAA	GAGATGAGATCCGAACCTCT
RM20084	6	17.07	258	GCTGAGGTGTTACTACTGAAGTGC	AGTTCCACCGTTCTATACTCACC
RM20162	6	19.75	211	GCATGACTTTGGCATTTCATGG	GAGGAAGACCCTCGTCTTCTACG
RM20163	6	19.75	184	CCATCGAGGAAACCAAAGTAAAGG	TTCTCACGCACCTTAGTCAATGG
RM20182	6	20.23	271	CCTTATTGGGCCAGAGATAGTTGG	CAGTGTTGTCGACGGTACAATGC
RM7193	6	20.26	140	ATGTGGGAATTTCTAGCCCC	CCCTAGTTTTCCAAATGGCC
RM20185	6	20.32	259	AAACCGCTCAACTTTAGCTAGG	ACCTCTACTCGTTTGTGTCTGG
RM3498	6	20.98	175	AGTGAAAGTCGGTGACGATGG	ACTTAGGGGATCAGGGGATG
RM3827	6	22.30	115	ACACCAGCTTCACTTCATCTC	CCTTTCTTCAATCTGCATTC
RM1340	6	23.34	164	ATCCAAACTAGTGGGAACGC	CTCAACGCCATGAACCTC
RM3272	6	23.62	122	ATTTTCTCCCGATCGGTGG	AACCCTAGCTCCTCGACTCC
RM7269	6	23.68	142	ACAAAACCGACACGATACGGAG	CGTGGTGCCTCCTTTTAAAG
RM7434	6	23.94	144	AGGAGGAAAGGTTGGAGAAGG	CCCGTATTCCATGAGCC
RM6202	6	24.26	106	ACTGTGACATTGGCATCCATC	ACACACACACACACACACCG
IDR0922	6	24.63	144	TACTTGAGAGGGACTCCTGGTC	GGCAGTCGGTGCTCCTTAG
RM5371	6	25.83	143	AGGCTAGCTTTAGCTGCGTTG	ACCCAGATCGAAACAACTGC
RM3567	6	26.00	159	ATTGACATCCCCATCTAAGCC	ATGAAACTGCCACAACCTCC
RM6395	6	26.00	110	ACTTCGGCTTCTGAACTAGCG	CAGTGCCGATGATCCTCTTC
IDR0931	6	27.02	146	CGAATTATCACGGTCAACTGAA	TAGACTGCCATGTAGGACCAAA
RM20518	6	27.12	249	GGATAGGCTGGCGTCTATCATCC	ACCTCTGGTCCAGTTGTTTCTGG
RM3430	6	27.43	156	ACATTGCTGCAACTCGATCAC	CGAGAGCCACCTAATCTTGG
RM7243	6	27.57	158	ACGAAGTTCTTCACGGGTTG	AAGATGGCGTGCGTACGTAC
RM5509	6	27.83	255	AGATGATCCATGCTTTGGCC	CCAGCAGAAAGAAGACGC
RM3138	6	28.47	106	ATTGACAAGAGATCAAGGCGG	GTGAATGTTGAGCTGCATGG
RM1370	6	28.53	173	ACAAACGAGAACCAACCGACAC	GGAGGGAGGAATGGGTACAC
RM6926	6	28.59	181	ACAGATGCAGCAGCTAGCAG	AATCTCTCATCGATCGGCC
RM6811	6	29.23	173	AGGTGATCACCAGCAACACAC	AGCGTGTGACTTCATTGCAC
RM3765	6	29.72	122	AGGTCTTAGGCCTCCCTGAG	GGATGCTTCCAATCCTCTC
RM1150	6	30.38	148	ACAGTGGCCACAGTGTGTTG	GGATTCGGGAGGTTGACG

Marker name	Chr.	Position (Mb)[a]	Size (bp)[b]	Primer sequences (5' to 3') Forward	Reverse	
RM3509	6	30.97	173	AGTGGTACATCCTCAAGGATCG	GTTGAGGAAGGGGGCTAGAG	
RM5753	6	30.97	203	ACAACATGCTCAACTTCTGGGC	GCTAGGTACGATCCAGCTGC	
RM1031	6	31.09	128	AGTGAAGGCACACCAACCG	GACGAGGATCGAATTCGAAG	
IDR0948	6	31.17	142	TGCAATTAATATATCCCACGCTTA	CTGTAAGTTTCTCAGCCCATCC	
RM20788	7	0.16	259	CCAAGAAGCAATCACGGATAAGG	AGCAGGTCGAGTACCAATTCAGC	
IDR0950	7	0.36	150	CATTGCCCTGCAACCAAA	ACTTCCCTTGGTTTGACATTT	
RM4584	7	0.50	203	ACCTATTTAATATAGCACCAG	CCATTTAAACATAGAAAAAC	
RM3394	7	0.65	204	ACCCTTACGTGCAGTACATTG	ATGCAGGCTACTTACTAGCG	
RM3222	7	1.70	98	AGAGGCATGGTAGGGACAAAC	CTGGTCTGGTGTACTGTCTGC	*
RM3831	7	1.17	180	ACTCCACGTTCTCCGACGAG	GCGGCAACTCCTACATATCC	
RM3325	7	2.22	175	AGGAGCCCTGAACTTTTTGTG	GGGGAATCCTACTTGCTTCC	
RM2381	7	2.42	143	ACAACCTCAAATATTTAAACTC	GCTAGAGAAAATAGAGAAAC	
RM5752	7	2.57	98	ACATCTCAACCTGCGAGGAAG	CTGAGAGAGACACACACAGCG	
RM5055	7	2.65	162	ACGTAGGTGATATATTGATCC	GTTCAAATTTTAACTAGCCA	
RM5711	7	3.14	145	AGTCCATGCATCCATCTCTAG	ACGGAAGGAATACGTCTGTA	
RM1353	7	3.31	196	ATGAGGTTCAAAATGAGACG	AAGCTACTGTCTGCCTCC	
IDR0964	7	3.37	149	TTTGACACGATAGTGCAGAGAA	TTTGTTCGGCCTAATCTTTCAG	
RM1243	7	3.56	158	ATTTCGGGAGGGATTATGACC	GTGACCCCCGATACAAACAC	
RM1134	7	3.59	145	ACACCCAACTTTTCTCACGC	AGCTAGGGTTTCGATCTCCC	
RM7479	7	4.14	216	ACCAGTTGCAACAAAGCTCTG	AGGAGCGTTTGTAGGAGCG	
RM2006	7	4.20	121	AGTGTGACTCATCCTAATACA	AGTACAACGAATCTGGATAT	
RM6872	7	4.66	96	AGGATGAACACTGATGATGGC	ACCTCCACCACGATATCCAC	
RM6728	7	5.73	144	AGGGTATGTGTCGCTATTTTA	GAAATCTGGAATTTTCCCTA	
RM5672	7	6.38	210	ACACCCTACAAGGAAACAAGC	GCCCAATATAGAGGCAACC	
RM21237	7	6.75	292	GTTTGACCGCGGTTTGACTAACG	TTTGAGTGGCATGCAAACAACG	
RM6776	7	6.84	169	AGCCCGGACATGCAAAAC	GAAGCAGGCGAAATCTCCTC	
RM1253	7	6.97	175	ACTGAACTTGCCTGAGAACTC	GACGACCTCTCCATGCTCG	
RM8006	7	7.69	278	ATGCCGGTTCTTAATTTTATC	AATGGTCCACATTACTCCAC	
RM8007	7	7.71	126	ACAATAGGATGGATCATGGATA	CATCTCATCAGGAACCTAAC	
RM21324	7	8.84	280	CCAGGTGAGAGAGAAGACATGACC	CCATCAGTCACACAGATCGACTCC	
RM3859	7	8.88	193	ATTGCAGATCGGTTTCCACTG	GGTCCTGGATTCATGGTGTC	
RM7074	7	9.01	168	AGGCTAGCGATTATTGTTGGG	AAGCTTAAAGCTGACGGCC	
RM3766	7	9.04	132	ACCTTCCATCCTTGCCGTC	CATCACACCTCTCCCTCCTC	
RM2256	7	9.15	167	AGTGCTTGCATATAACCTATA	AGATCAACCTTCTTATTCAG	
IDR0991	7	9.83	134	ACCTAGTGGCCAGCAGCAG	GCTCTGCCTCCTCTTTCTCAA	
RM5499	7	9.99	240	ATGGAGTACGACGTGATCGTG	CAGAAACGGGAGGGGATC	
RM21364	7	10.25	247	CACTTGTTTACGAGGAATGACC	ATCTAATACGTGGCACCTGTAGC	
RM3635	7	11.09	109	ACGTGAGAGCGTGAGAGACAG	ACTTTGGTGTTCCCTCCCTC	
RM3832	7	12.71	163	AGTAAGGCAGAGGAGACACGC	CGCTCCTTTCTAGCTCCATC	
RM8025	7	13.00	148	AGGTTATTCTTGTCAGTCATT	CAAACTTTGACATAAATGTG	
RM7273	7	13.43	158	ATGTCCAGTGCGCAATACTG	ATAGGATGTCCATGTGGCG	
RM3670	7	13.44	112	AGATCAAGAAGCAGGACACCG	GATCTGGAGTCGCGTCAAAC	
RM3755	7	14.73	185	ATTGTTCAGAGCATCCTGCAG	ATCACCAACATCGGAGAAGC	
RM5543	7	15.75	167	ACCACTTGCTGGAATCCTTG	GCAAATTCTGGGCTATCTGC	
RM21560	7	16.99	218	CCGTGCTTTGAATTGACTACTGC	TTAGTCAAGTAAGCCGATCGTGTAGG	
IDR1024	7	17.00	134	AGGGTTCAATCTGAGCATGTTT	TCGTTGTAGCCTTCCGAATAAT	
RM3795	7	17.19	118	ACATTTGCATGGAGAGGATAG	CATCTTCATTTCATTTCACC	
RM6767	7	17.47	185	ACATTCTTGATCTACGTGGC	AATTATGGTTGCTAGGTTGG	
RM4952	7	17.80	166	ACGCTTCTTCTTTTTTTAGC	GGGTGAACATCATCTCTAT	
RM6394	7	18.39	129	AGGGCACACTGCTTGATCC	CGACGTCCACGATGACTAC	
RM5380	7	19.36	107	ACCACCCTGTTCATCTCGC	ATGGCGATACCACCACTCTC	
RM7087	7	19.36	115	AGCTAGCAGCTATTGCCTGC	CAGTGAGTGAGTGCACTGGC	
RM21701	7	19.58	251	GCGGGTGCCTACACATTTAAGG	GGCCGAATATTTAGCTCAACAGC	
RM3404	7	20.11	136	AGTCCTGAGTCTCCTGTCCT	CCTGTTCGATCTTGAACTTC	
IDR1040	7	20.13	138	GCAGATCGCTGACAATCTAGTG	TGTTGGTACTTCATCGGTAGGA	
RM1048	7	20.17	139	ACAAGCCTATAATGTGAATTG	AATTTTTAGTTTGGGGTAGA	
RM3826	7	20.81	206	ATTAGCTTTCCTCCAGTCTCC	ACGGGTATCTGAAACACAAC	
RM1279	7	21.61	182	AGGGTATAAAATGCGTGGCAC	ATGGATGGTACGAGGACGAG	
IDR1050	7	22.05	127	AGCAACTGATGGTCTTCTCGAT	GTACGCATGAAGGAATTTGTTG	
RM6152	7	22.13	206	AGAATTCACCGCTCTCCAGTC	AGGAGGATCTCCTCCAGGAG	
RM6885	7	23.01	135	ATTCGGGATCTTACATAGCGC	CCTTCTCCTCTACCTTCTCC	
RM5508	7	23.56	177	ATCGCACACTAGCTCGATCAG	GGTCCTCTTCTCCATCCAG	
RM5847	7	23.65	194	ATGAGATGAGAGATAGACTCC	AACAGATGAAGGCTATTTTA	
RM3753	7	23.66	140	ACCGAGATTAAAACTCAAATG	ACTTTTGTAGATTCCTCGAA	
RM6326	7	24.20	223	ATAGCTTGGTTCGGTCAAACC	ACCAGATTGTTAGCAACCCG	
RM1330	7	25.66	162	ACAGAGCTGTGGAAGAGGAG	CTCCTCCAAACCGACTTC	
RM5426	7	25.93	196	AGAGAGTAGTGGCTGTTCCGC	GCGGTTTCGATCTCTACGAG	
RM6216	7	26.40	151	AGGAGCGCATGTGGAAGTC	GTTGAACTGATGAAACCCCG	
RM5455	7	26.46	178	ACTCGGCCTGACTAGTCGATC	GATGGCGATCCTGTGATAG	
RM1364	7	26.78	159	ACAAGAAATTCAAAACACATGA	AAAACATCTACTTTGATCCA	
RM6063	7	27.61	157	AGTCGCTAGTTCACTGCCAAG	GGGGGAGGATAGGATTAGG	
RM5720	7	28.67	196	ACCTGATAAATTGACAGTTAC	GAGAGTAGGAGTTGATAACA	
RM1306	7	28.95	105	ATGCCAATTACCTTCCCGTAC	GCTCCGTATTGCTGCTATG	
RM7601	7	29.04	134	AGCCTCGCTGTCGCTAATATC	CAGCCTCTCCTTGTGTTGTG	
IDR1081	7	29.43	118	CAACATTGTCATCATGTGTTGG	CCACCTTGAGTAGAACCACCAT	
IDR1082	7	29.63	149	GCGGGTGAGTACAATTTGGATA	AGACAGCCATCAGAAAGAAACC	
RM5911	8	0.08	152	ACCCTCTTTTTAAGTCTGGGG	GGTGCCTCCTTTCAAAGTTG	
RM6369	8	0.13	118	ACAAGCTAGGGCTGCATAAGC	GCTTCACCTACCTACCTCACC	

Marker name	Chr.	Position (Mb)[a]	Size (bp)[b]	Primer sequences (5' to 3') Forward	Reverse
RM7034	8	0.15	150	AGCGTTGGACAACTGCTGTAG	ACATGAAACGTCCGTCTAGC
RM1381	8	0.42	218	ACAATGCTTGCATTCGTTACAC	AAATGGGAGTTCTTGTGC
RM8018	8	2.17	121	ACAATTCATACACACTTGTGCC	ATTTGCTTGAGCAAGCTTAT
RM4955	8	2.96	165	AGCATCCAGCAATATAATCAA	CAAGGATTTTGTTAAGTGGG
RM6670	8	3.00	115	ATGGAGATGCATGCCTATACG	AGCAGCCATGTCAACCATG
RM3819	8	3.02	208	ACCTCACCTGTGGATCTTGG	CAATCCCCTTCTCTCCTTCC
RM5434	8	3.13	166	AGTTGATTCTGCGCGAGTTTC	GAAACCGCCCACGCAAAC
RM1376	8	3.17	200	ACATGTGTGATGACTGACAGG	GGTGCTGTGATGATTCTTTC
RM1148	8	3.74	125	AGCTATGCAGGAGAGAGTGGG	GACTTTCTCTTTGTTGCCGC
RM6999	8	3.99	159	ATTATCTGGGATCCATCGAGC	GTGAATTTCCTTGGAGGGAC
RM22502	8	4.64	272	AATCTTCACGCACCCATTCC	GCAGATACACACATTGCATACCC
RM6208	8	5.79	144	ATCGAGCAGTACGTGGATCTG	CACACGTACATCTGCAAGGG
RM6838	8	5.85	120	ATTAATACCGCTACCACGCG	CCTCCTCCACCTCAATCAC
RM3507	8	6.16	123	ACCCCTATCGATCAACCCTC	CGTTTGGTGTTAGGGGC
RM22634	8	7.33	209	TTTCACCACTGTAGTCTCTCTCC	CTCGACAGTTTCTTAGCTAGTCC
RM2584	8	7.57	153	ACAAGATGAAGTTCACTCTGGA	ACTTGAGATGATCAGATTGC
RM3214	8	8.57	150	AGTGGGGAGCAAGACAGAATC	CAGTAACCAACCAGCATGG
RM8243	8	8.94	208	ACTCGTGCAACCATTATATTC	ACCTTAGCTGTCCTGAATTG
RM6027	8	9.11	135	AAGCTCAACAGCTACCTCGG	CCCGTACACCACCGGAAAC
RM7267	8	10.19	153	ATTCTCTCCCTCCTCTCCTCC	ACGTGGCAGCTTGAGAGC
RM3395	8	10.29	97	ACCTCATGTCCAGGTGGAAG	AGATTAGTGCCATGGCAAGG
IDR1130	8	10.36	132	ACGAAACAAGCCTGAACAGAAT	GAGTAGGGAATACCGTTCGTGA
RM5514	8	10.41	120	ACTTCGAGCCCATACTCCATG	ACTCCGAACGTTACTCTCGC
RM6032	8	11.90	96	ATCAAAACCCTCTTCGGCTC	GGAGGCAGACGAGCATAAAG
RM22837	8	12.38	277	ACCTGGGTCAGATGTCTGTTTGG	GGTAGAGCTCCATCCATCTTAGTGC
IDR1139	8	12.46	149	TTCTCGTAGTAGTGGCAAGATCC	TACTGGGCAGAACTTTGTCGTA
RM3383	8	13.49	165	AGGTCGTCCCCTACCTCTAGC	AAAGAGGAGGAAAGGAACCG
IDR1151	8	14.05	142	GCTGGTCATGTGATAGTGAGTTG	TGCACATACACAAATATACAAGC
RM6990	8	14.36	131	AGGTGTGATCCTTTCTGATGC	ACGGGTGTGATCCCAGATAC
IDR1161	8	15.50	148	CAAATCGGTGAATTTACGAAGC	ATGATACTCCCTCCGTCGTTT
RM22934	8	15.97	219	CATGCACATCTGCAATTGATCG	CCTTGAACGATGCCTGAGTTGC
RM22965	8	16.64	192	GTGGTTGTGAGGTTGTATCAGG	CGTGTGTGCTTGTATAGTTCTGC
IDR1170	8	17.82	147	CCTTGGATCTGTGGTTTGTTTC	GCAACCACTCCTTATGACCTGT
RM7285	8	18.76	186	AGCGGCTATTGTAAGTGTTTG	ATATGAGTGCCACATGACG
RM5767	8	18.82	99	ACTAGCAGCCACATCAAGCAG	CTCATCCTCTCCACGCTCTC
RM3153	8	18.99	155	ACGGTTCTTTTCACATGGTCG	ATCACAACAAGCTCGACGTG
RM4815	8	19.01	145	AGTAAATTTCACAAAACTTC	GTGATACAATGCATTAAATA
RM6215	8	19.07	163	ACAGCAGAGAGATGACGCAAG	AAACCCAAAACCCTCGTCTC
RM3491	8	19.62	122	ACAACCCCAACAAGGACTCAC	CTGATGTTCCCTCTCTGCC
RM3459	8	20.65	187	ATGGACTTTCGAGAATGTTG	GAGTACGAAATGAAGGCAAG
RM5887	8	20.97	381	ATTTCAGGGTTACAGCCCTACC	AAATGCCACATCTATCCCTACC
RM3634	8	21.58	153	ATTAGTGGAGCTGATCGAGCC	GCCCACAACCTCGTCATC
RM6635	8	22.17	172	AGACCGTACCGAAGTCTCTCG	ACTGTTGCTGTCGATTCGG
RM7556	8	22.21	131	ACGCTAATACGGCGCAGTAG	AGGACTGACAGTGAGAGAGTCG
IDR1186	8	22.48	150	GGTTGAGAACATTCAATATCAACA	TTTGAAAGCTCACCGTATTAAA
RM23251	8	22.48	260	TCCGATACTCCCATAGTTAGACC	ATGTGGGTTGGCTATAGTCTAGG
RM5485	8	24.07	144	ACTTCCACAAGCTTGGCTAGG	AATGCCATCCCCTACTCATG
RM8058	8	24.57	136	ATATGATTTTCTCAAACAAC	CCAAACTACTAAACAGTACA
RM5891	8	24.63	143	ACAAAGAGCTGATTATGTGTT	AATAGGCCTGTAGTTTTTTC
RM3452	8	24.78	190	AGGCAGCCCATCAACTAGATC	GCAAACCCTAGTCCAAGC
IDR1200	8	25.74	141	TCTCAATTCGGTTACCTTCCAT	AACGATTAACTGATGGCGATTT
RM1345	8	26.15	124	ACCACCACGCCATTAGAGAC	GAGCATCCCGTGCTGTC
RM5493	8	26.15	155	AGCAGGACACAGTCACACAGG	AGATTCTTTCACCGGTGACG
RM3840	8	27.93	196	AGTTGGAACTTACTTGGAAGG	GTCGATGATGAGCTTGTCC
IDR1209	8	28.10	114	TATGTCGACGTCTCTCATCCTC	CAATACCAATTAGGGCAGGTGT
RM4997	8	28.24	178	ACCTTCACGGAAAATTAATGA	GTACGACAAGATCAGTGTGC
RM4154	8	28.25	152	AGAAGATCGATCGAAGAAAC	ATCATGAAACACAATAAGGC
IDR1212	9	0.50	135	CATTCCTCCGACAGGTTCAT	GGATGTCCAAGAAGCGATTATG
RM23668	9	0.65	160	TGCATAGCATATCAACTAGCCCTACC	GCTGAAACAGAATGAAAGCACAGC
RM3609	9	1.16	158	AGGTGCACACATACTGTCGC	AGGACATGGCATCTTCTTGG
RM4413	9	1.17	143	ACAAGCTCTAAACTACTCAAGC	ATGACAATGTGATCTAACAC
RM8219	9	1.55	170	ACAACCATTGTTGAGCAAATTC	GATAAGCAGGGATTGGAAAG
RM23736	9	2.46	199	GGCGATACCTGCCATAGTTTCC	CCGAAAGCAATCTATGAGACACC
IDR1224	9	2.56	146	ATCATCGCAAAGGACTGAAACT	ACAACCTTGATGCTGATGAATG
RM23766	9	3.64	198	AATCTGATGCCTGACCATTACGC	GAAACATTAAAGCCTCCCATGACG
RM5799	9	3.80	199	ATCGAACCATCCAGGATGAC	GCACAAGAGGCAACACTC
RM2855	9	3.84	256	AGGAGCTTAGAATCTCACCTA	CGCATTTTCCTATACATACA
IDR1231	9	3.91	101	CAGTGGCAACGACACAACTC	ATTGGTCACAATTGTCGCTTC
RM3502	9	5.03	157	ACTATGGAGGCCTAACCCTCC	GATCGATTCAGGGTTCTTGG
RM5899	9	5.12	205	AGCGTTGTTTAACCGTGGTC	CCACTAAAGCCACCTCGAG
RM23823	9	5.13	149	AACTTGGACGTGACACTAACAAGAGC	GCGATACGGGAGCGTGTATAGG
IDR1242	9	5.77	145	GGGTCCAGATCAATAGGACTGT	GGGACAGAGCTCAACACTTTCT
RM7212	9	6.60	151	ATTGTAGGAGCGCCATATGG	GAGCTGGGTAACGAGTCGAG
RM6920	9	7.01	209	AGAGCGTACCACAAATGAGG	AATCGTATTGCCAGCGAGAC
RM5515	9	7.15	126	ACGCACAGGAGAAAATCCAAC	AGCATTATGGGGTGGAAGG
RM23914	9	7.24	197	GAGGATCCTTACCATCAAACTTCG	CCAAGAACCTGCATTCTTCAAGG
RM5526	9	7.31	171	ATCAGCCTGGCCTCTCTTATC	ATGATCCTCCACCCACTAGC
RM23924	9	7.43	184	TAATACGTGGCGCCTGTAATACC	GATCAGTGAACATGATGGAACACG

Marker name	Chr.	Position (Mb)[a]	Size (bp)[b]	Primer sequences (5' to 3')	
				Forward	Reverse
IDR1250	9	7.64	119	ATGTAGTGTTCCACGTCCATGA	TGACCAAAGATGATGGAGAACTT
RM23941	9	7.82	285	AGAATCGAACCCTAACACATGC	TATCGCTTGATTCTTGGACAGC
RM1328	9	9.21	206	AGAATGGGATTAGACGATTTG	CCATGAGTGACATCAAAAGG
RM7364	9	9.56	204	ATTCGTGGATGGAGGGAGTAC	AGCGTTTGTAGGAGTGCCAC
IDR1261	9	9.61	141	AGTCCGGAACAGCTAATGAGAA	GTGGTGACGACAACAATCGTG
RM7387	9	10.14	170	AGCAGTAGGGAGCATGGAAAG	AAACGAGTCCTCTTCAGGGG
RM7390	9	10.47	141	ACTGGTTAACGTGAGAGCTCG	GCAGATCAATTGGGGAGTAC
RM3907	9	10.49	133	AGGAGGCCAAGGAAGAGGTAG	CGTCAATGGGGTAGGTCTTG
RM1817	9	10.62	131	ATAGTATTCTTTCCTTACAGA	ATTGAAAACTTAACAAATAG
IDR1269	9	11.20	94	ACAGACCCTTGCATCACATAAA	TCCAAGAAATCCATGTGACAGT
RM3769	9	11.75	103	ATGCATGCTTCGTTCAGCTAG	GTCTCCGAGCTCCTCAGGTC
RM1896	9	11.77	93	AGGACAGGGTAAAGTGTTAGA	CCTAAGACCTATCAACTCCA
RM6475	9	12.84	209	AGATCAAAGCAACGGCTAGC	GAACAGAGAGGGGACGTGTC
IDR1277	9	13.10	149	ACACACTGAAGGTCTCCGGTTC	AATTCAGTGAGTTCGGAGGAAA
RM409	9	14.37	183	CCGTCTCTTGCTAGGGATTC	GGGGTGTTTTGCTTTCTCTG
RM5657	9	14.37	144	ATATGTGCATTTGTAAGGTGA	GCTTTAGATTATTGAGCGAG
RM6854	9	14.37	136	ACTGTGGGAGAAATCTTTGTA	AGAGATGATCAATCCGTCTC
RM6839	9	14.57	105	ACTACTGTTGCAGGCTTGCAG	CAGAGGAGGAGATCGAGAGG
RM5652	9	14.79	201	AGTCCTGAGTGACTCGATAGCG	CACGCGTACGTAGCAGAGAG
RM5122	9	15.25	162	ACTCGCAATTTATACGTAATC	CTCACGAAATAAAATGAGTG
RM3700	9	15.43	143	ACAAATGCCCCATGCACAAC	GTCAGATTGTCACCAGGG
RM1189	9	16.27	178	ACAACTGCCCATTTGTCGTC	GACTCCGGACTAGACCAATC
RM3025	9	16.39	203	AGGTGGCAAGAAGTTCCTAAT	GATTTCCATACAACCTGTGC
RM3492	9	16.50	155	ATCTCTCTCGCTCCCTTTCC	GAAGGCAAAGACGGTGGC
RM7048	9	16.94	166	ACAACCCCTAATTTCACGCTC	GACTTCACTGGCACTGGATG
RM7289	9	17.26	203	AGAGGAGGCTGTTAGAAGGCC	AACGTGGTGTCTCCTTTTCG
RM3165	9	17.81	223	AGTTCCGGTCGGGACTAGTTC	GTGGATGGGAGCAGGTGTAC
RM3533	9	17.89	122	ATTCCAACCTGTCAGGGAATC	CATTTCCCTTCCCTCTCCTC
RM24559	9	18.45	198	AGTTGAGTGGCAAACCACAGAGC	CTTCACTTGGGTTTGGGTGATGG
IDR1301	9	18.57	139	GGCTTTAATCCTTTAACCATGA	ACCACCTCTTGGTAGCTCTTCA
RM5661	9	18.96	168	AGTTGCTGGGCTTGATCTTTG	CTGTCATGGCCCCTCATTAC
RM5786	9	19.49	183	CCCAAATCAGGAAAGTTTCTCAGC	AGTCATCCTTGGCTACCAGATGC
RM3909	9	19.54	181	ACCTCTTTGGACATAATGGGC	GTCCTCCCCACAGAACACAC
RM3787	9	20.04	86	ACGAAAAACGAGCGAGCAC	GACGCTGGTAAGCAAAGCTC
RM24650	9	20.14	282	TAAGAGATCAGCTGGGATTACG	GATCTAAACAAGGCCTTACACG
RM3808	9	20.55	120	ACGTTAGCGAAACGAACAGTG	CAGTGGCTCGGTAATCGC
RM1553	9	21.00	163	ACAATTAGAGGGTCCACATGTC	ATTACCCTCATTTTCTACGC
IDR1309	9	21.26	105	AGAGAGAATCACCGGGAGAAGT	CTACTCACGGTGCTACCAGATG
RM6971	9	21.89	202	ATTTGCGAACTAGACAAGGCC	GCGTCATTCTCGACGAGC
RM5384	9	22.07	138	AGTACTACTGCTGTTGCTGTG	ATTAAAACCAATGACAGTGC
RM2482	9	22.58	142	ACATGTGCTTTCACAGAAAGT	GGCTCAATGACAACTAAACA
RM2255	9	22.59	198	ATTGACATGATTATGTATGA	ATCAATCTTTTCCTATCTAA
RM6797	9	22.71	152	ACCTCCTCCATCAGGATCATC	GCTAGGTTGAATGCCCGTAC
IDR1314	9	22.91	125	AATTACCAGCTCCTCCTCTTGA	TACGCCCTTCCAGTTGATCTAT
RM5095	10	0.05	181	ACTATATGACTATGCGAATGG	ACAAATGCAACTAAGGTAGA
RM6364	10	0.05	257	ACCTTCTCCGCTCATCCCTACC	ATTTCGTCCTTCTCGGATTTGC
RM7492	10	0.05	146	AGATGGTTGCCAAGAGCATG	GTCACGTGGCGATTTAGGAG
RM6370	10	0.35	91	ATTGACAAGCCACACACACAG	GTCCTCCCTTGGTTCTTTCC
RM24955	10	2.16	174	GCCTGTGTGCATAGGATACGC	CGATGAACAAAGTCACCTACAACC
RM3882	10	2.74	100	AGGTGCCCAATTTAGCAGAAC	CGGTGGGTTCCGAAATTTC
IDR1326	10	2.85	127	CAGTTAGCTTGTGTTGGCATTT	AATGAACATCGATCACATCTCG
RM25024	10	3.59	271	ACCCAGTAACTCCCATGACATCC	GGTTGACCTTTCATAGCATGTGG
RM25031	10	3.66	196	GCTGTATCAGCTGACAACGAGTACC	GACATTCATGAAGTGAAGGGATGC
RM7545	10	3.79	437	GTTTCCATATCCGTGCTATTCG	CACGATTCCTACAATACGAGAGC
RM7217	10	4.63	211	ATTTGTAGGATGACACGTGGC	CGGGATTTCAGTACCTCACG
RM6271	10	4.78	77	ACCTCAAGATTCCAGCTGTC	AAGCGGAAATGCTGCAGTAG
IDR1336	10	4.85	82	ATGCACTCATAGCTACCGGTTT	AGGCACCAATAATCCCTCAACT
RM25074	10	4.87	198	GGCTGTATCAGGTGCTGTTTAATACG	GATCAGATGAACGGTGGATGG
RM25101	10	5.72	148	ATCTTGGAGCCCATACTCTATGTAGC	GTGGATGGGAGCAGGAGTACG
IDR1341	10	6.01	138	TGCTAGATTAACGGAGGCATTT	TGGGTGATTTAACTCGGTCCTA
IDR1356	10	7.55	140	TCACGTTGTCCCATTGAAATAC	GACGACGGTGCTATTCATCAT
RM2504	10	7.60	186	ATAACACAACAATAGCGTCAG	AGGAAGAACTGAAGAAGCA
RM25151	10	7.62	223	GTGGGATATCCGTTTCACATGC	CTCGCATATATCCAAGTACCAACG
RM25158	10	7.94	209	ACATGTAGGGTGTAGATTGTGG	ATTGTGCTAACTCCTCTGAGC
RM25188	10	9.18	94	AAGGGCATGTGAAGTGACAAGG	CCAACTAAACAAGGCCTAACAACG
RM25209	10	9.50	574	ACGATTCCAGCAAGACGAGAGC	GGGATTTGGGACCTAGGAAAGG
RM25217	10	9.75	364	TGGCAGCCTCTATGTTAGACC	GATGCATATCGGTGATTTGG
RM1126	10	9.77	139	AGAAAAGGCTGCATCAGTGC	CCAACGACAGACTGTACGG
RM25261	10	10.93	295	GACATTGCATCTTGCCTTGTATCC	ATGCTTGAGATGGCATTCAGTCC
ID11-43	10	11.14	169	TCGCCATCAGTAACAGTGGT	CTTGGCGAGTTCAACGATTT
RM6833	10	11.41	132	ACCTTTCTCTCCCTTTACCAC	GAGGTAGCTTTGGCTCCATG
RM4455	10	11.74	118	ACTCTCAAAGAACTAGGACTC	GAGAAGGTATGATAACCAAT
RM25302	10	12.03	264	CAATCCATCGCTGTCCTACGC	GCCTTCAACTACGCACACTAAATTGC
RM5758	10	12.99	198	AGAGGCCCTGATATTCAATGG	ATGGCTTAGCGTTAGACCG
RM5689	10	13.55	106	AGCACATGGTGAGACGTCCTC	AAGTCCTGTAGTAGGTCACACCG
RM8201	10	13.83	163	ATCTGTTTATAAGCGCAGCAC	GCCGGCGAGCTACTACTAC
RM5708	10	14.53	187	ACAATTAGAACCATCTGAATTG	GTATTTAAGGTCACGTATCG
RM5806	10	14.56	166	ACTAATTGCGGTTGAAGCCTC	CCTCCCAATCTTTGCACATC

Marker name	Chr.	Position (Mb)[a]	Size (bp)[b]	Primer sequences (5' to 3') Forward	Reverse
RM25425	10	14.94	155	CCAGCCCAAACAGCTCTTGC	GGGCACTGTTTGTCTTTCTGTGC
IDR1389	10	15.24	92	CCTGTCATATGCTGTCAGGTGT	GACTCTTGGGATCTTCAGCTTG
IDR1392	10	15.92	113	GTTAACCGGGTACTGTCCAGAT	CAAAGCAACCAAAGAGACCAAC
RM1375	10	16.72	181	ACTACACGCGCAAACTCTGTC	ATGAAGGTCTAGGCTGCACC
RM5620	10	17.47	177	ATCGACTTGAAGCATCACACC	CTGAAATGTCAAGTGGGCC
RM1873	10	17.89	152	ACTGACAGGACATTAAAAAAC	CCTCATCCTTAATCTCTTTA
RM5274	10	17.92	121	ACAATGTTTATCCAACTCATGT	ATTTAGAAACGAGTGTTGTG
RM1937	10	18.00	120	ACAATAAATAAAAATCCAGCAC	AGATCAGATATGGCATTAAG
RM5756	10	18.00	144	ACAGACTGACAGTGACCATGG	AAGAGGAGCTACCATGACGC
RM6737	10	18.78	188	ACATTGGGGGTGGATAAAGAG	ATCCTCTACTCCCTCGGCC
IDR1403	10	19.08	104	TCGGTTTCTGTACATCCATCAA	GGAAATGAGTGAGGCTGACAAT
RM2371	10	19.65	230	ATAAGTACTTGAAAAGGATGA	AGCAGACATAATCTAGATCA
RM3773	10	19.97	153	ACTGGATGAAAGGATACAACA	CACATTATCTGTCAAGGTCC
RM7300	10	20.00	102	ATCCGTATCCTAGTCGCGATC	CGCCGTCATGACTCATACTC
RM25771	10	20.71	298	CCTCTGTGGCTCTGTGACGTACC	GCATTTGGCTCTTATTCGTTGTCC
RM4477	10	21.17	167	AGTAAACATGTCTTCGGGAT	CAGTGCATATTCCACTGGTA
IDR1413	10	21.65	149	TGTTAAGCAACTTGCCTCTTCA	ACTTTAATGACTGATCGCACAA
RM6673	10	23.08	148	ACATCGCATCGTATCGTATCG	GCTTCAAACACGCCTTCTTC
RM4771	10	23.09	143	ACGTTGATTTCATTCAGGTC	ACGCTAACTGAGAAACATGG
RM6824	10	23.11	131	AGAGAGAACCTGGTGGTGGAG	AGTGGTAGAAGATCCGAGATCG
IDR1420	11	0.22	130	TTGCAAGGAACTACAAATTTCTTC	AGCTACTGCCTTTCAAGGTGAT
RM1761	11	0.31	143	ACGCTTAAAGAACATTTGAT	GCGATTAACTTTTAACCATT
RM6327	11	0.36	212	ACAGCCTAGGGCGTCATAGAC	GATTGGGTGATGGATAGCAC
RM3863	11	0.90	137	ATTGATCCCGTGCAAGTAGG	GCATTCTGCGTAGGTTTTGC
RM7203	11	1.08	107	ACGTGGTGCCTTCTTTCAAG	CTCTGGCCTTCTACTCATGG
RM3668	11	1.46	100	AGGAGGGAATCGTTCACAAG	GATCGTCTTCAACCTCCAGC
RM1812	11	2.41	136	ACAGCTAGTGAGCTCCTAGTG	GCTAACCCACCAACTTATTC
RM2459	11	2.41	128	AGTTTGAAGTTTGTCTTGAA	AGTTACCAAAAGTTTAATCG
RM5918	11	3.03	195	ATGGCTGTCGATTGGCTATC	CTGCTAGTGCTTGCAACCAG
RM26123	11	3.08	279	CACGTCTAGCCGAAATCATAGAGG	CCCGACAAATGGATAGACAGTCC
IDR1431	11	3.25	123	TATGAATCCCAAACGGTGTACG	CGGCTTAGCATTCGTTCTCTT
IDR1433	11	3.83	136	TCGATTTCTGGATGGATGCTAT	GTCCTCCGTGCGTTACTAAATC
RM5599	11	3.83	144	ACTCACAATATCACCATCCAC	AATTTTGTGCTGTTGTTGAA
ID07-43	11	4.02	191	AAGCCATGAAGCACCTTGAC	AATATGACGGGCTGGAACTG
RM3339	11	4.14	98	ATGCTGCTCGTCTCCATGG	GAAACCAACGAGCAAACGG
RM5704	11	5.40	390	TTGATCACCTACACGTACACATGC	AAGGTCCTTTGAGCATTCAGTCG
RM5128	11	5.48	178	ATAAGTAATGATCATTGGTAA	GTGCCATATATATATGTTGA
RM3133	11	6.19	100	ATCAATAGACACACGGGCATG	CGATTTTGCTCACTGCACAG
RM3137	11	6.19	197	AGTTAGGAATTCCATGCTGCG	GCCCGTGCTCGATAAGG
RM4469	11	6.26	202	ACAATTTCTCATGTTTTCTTCC	AGTTATTCTAAGGGAGGGAC
RM3625	11	6.67	143	ACTTGCAATTCAATTGCTTAC	GGTGGCCTAGTGAAACTAAA
IDR1449	11	7.54	150	TGCGCGGTCCCTAATAGC	GAAGAACTCTCGTGCTCATGG
RM26341	11	7.73	183	AGGGCCTGTGTGCATAGGATACG	CCTCGATCGATCTGACGTACTCG
RM3701	11	8.11	176	AGAGCTAGAGGGAGGAGGTGC	GACTGATAGCCGATTGGG
RM7283	11	9.12	184	AGGGGCATATAACGCAAACAC	ATTTTAGGAGGCTCACGTGG
RM1206	11	9.77	89	AGTGGCCCTTTGAGGCTTG	GTGCTTGTCCAGGCTAAACC
RM5731	11	9.96	202	ACGATGCACCTAGCGCATC	CCTCCACCCTAAGCTTCTCC
RM4862	11	9.99	160	ACAACTTTCTGGCATAAACTA	GGTGAAAGATATTTCAGAC
RM7463	11	10.20	187	ACGAGGATCACACCAGTTTTG	GCAGAGCCAAGAAAGGAAG
IDR1467	11	11.58	131	AGATCCAATTCATTGTCAACCA	CACACGTGTCCAGCATAGAAAT
RM5590	11	11.69	195	ATAAGCGATTGAGGTAGCTGG	CCCGTTATAATGAGGGAGGG
RM5857	11	11.86	156	ACAGCTTGTCTTTATTTCCTG	GATGGTAATCCAGGTTGTTG
IDR1473	11	12.84	112	AGCAAACAATTCCAAACTGTCA	TGTCTTCTCCACATTGTTTAAGG
RM26567	11	13.39	293	CTGTGGTAGGACCGGCTCTATGAAGG	GGGAGGTTGGTGTGACCCTTGG
RM7226	11	14.51	166	AGCGGCGTATTAGCGTTGTAC	CCATAAGGTTCTAGCCCATG
RM5824	11	14.53	191	AGACCTGCAGTCGTTCGATTC	ACCGATCAATTAGGAGCTCG
IDR1487	11	15.37	130	CATGTTCTACTCTTTGGCGTGA	GGAAAGGTCACTACAGCAATATC
RM5960	11	17.49	153	ATGGAGGAGATCAAGGAGCTC	CTATCTCCTAGGTGCAGCGG
RM5312	11	17.63	174	ACAGCACCAGCCCTATGAAC	ATATCTTCCCTGCAACCGTG
RM1355	11	17.67	127	ACCTCCACATAAGCGAAGAGC	GACGCCCAGAAGAGGTATC
RM209	11	18.27	127	ATATGAGTTGCTGTCGTGCG	CAACTTGCATCCTCCCCTCC
RM5582	11	18.31	167	AGGCGTGTGTGTGTCTTTTG	GAAACTCTGTACCTCCCTTGTG
RM6897	11	18.67	149	ATATCCGATGTGACACGCAG	AGGATAAATTGGGTGGGGAC
RM6680	11	19.08	174	AGGAGGACGTTGATATGTCTCG	GCGACACAGTTACAAAGCC
RM5349	11	19.65	123	AGGGCATGCTTACATCCAAC	CATTTGCTTCTATGCCCCAG
RM1341	11	20.14	184	AACCTGGAGGTGCTGGTCTC	CTCCCCCCCAACCAC
RM1219	11	21.52	156	AGAGGAATGGAGGAGTTTGGG	CCGGCAAGGAAAAGGAAC
RM6534	11	21.52	186	ATCATAGTCATGGAGAGGGGC	CCCAAACTCCTCCATTCCTC
RM26969	11	21.97	266	CTCACACTTGCAACATCCTAGC	AAGGCTCTAGTTGGTGAAGACC
IDR1516	11	22.31	117	CGCATATTGCGACTCCATATTT	TCAAGAAGAATGCAAAGGTCAA
RM4601	11	23.11	95	ACATACATGTGAACCTGACTG	CTAGCTTAGCATCTCCTCAA
IDR1519	11	23.15	147	AGATCAGCTCGACGCATTTACT	CCCGTGTGCATAAGATACATACA
RM5997	11	23.18	186	AGCGACGACGAAGAAGCTAAC	CCCATCGATAGGGTTTCCTC
RM5716	11	23.47	211	AGTAACACCAAGTTTCACTTA	ACGTGTATAAATGGGATAA
RM6499	11	24.08	154	ATAGCGATGGGGGTCAAGTC	CACCATGGAGTCCCATTGAG
RM27096	11	24.33	286	AGTTAGGATCGCTTCCAGGTTCC	TCCAACTGGAATATCGTCTTGTAGGC
RM7277	11	24.68	160	AGCTGAACGTTTCAATATGTA	GTTTGTAGGGAGTTTAATGG
RM4112	11	25.11	154	ATGGCAAAGTCAGTAGTCCTTCCACAA	GCCATTCCCCCAACAGCTCC

Marker name	Chr.	Position (Mb)[a]	Size (bp)[b]	Primer sequences (5' to 3') Forward	Reverse	
RM2191	11	25.13	181	AGATAAGCATTTTAGAACACA	ACTAGACCAAGGAATTATTG	
IDR1530	11	25.19	85	TCATCAGAGATCCACATCTACCC	TACCATCTCATCCTAACGACCA	
IDR1533	11	26.03	110	CGCCGCACTTTAATTTGTC	ACGAAAGAAGCTGCCATGC	
RM2064	11	27.49	171	AGCTACCTTAGCTAGGTGATC	ATGTAAAATTTGCATGTTTG	
RM7654	11	27.67	174	ACTCATGGTTGTGTCGTGGTC	GTGCAGTGCCAGTGGTACG	
RM144	11	28.80	237	TGCCCTGGCGCAAATTTGATCC	GCTAGAGGAGATCAGATGGTAGTGCATG	
RM2136	11	28.93	154	ATGTTTGAGAAAATGCAGAC	CACTAAGCTCGTTTTCAAAG	
RM6094	11	28.93	184	ATGCTTGATCTGTGTTCGTCC	AGCAGCACCAGCATGAAAG	
RM5926	11	28.96	170	ATATACTGTAGGTCCATCCA	AGATAGTATAGCGTAGCAGC	
IDR1548	11	28.99	143	CTTGTTGATTCCCATTCCAAAC	GGTCATTGTTGGAACAGTATGC	
IDR1549	12	0.10	148	TTTCTGCGGATCTGACTCACTA	GCAGTAGACTTTGTCAAGACGAGA	
RM27400	12	0.11	241	CTCGTTCCTCCTTCCTCATCACC	GGTGAAGTACTCACATTCCGATGG	
RM5367	12	0.17	185	AGTACCTCTCACTCGCCTGC	GTCAGCTGTGAGTGAAGTCG	
ID12-20	12	0.38	210	CAGTTGATCACCAGATGTAGGG	CTCTGCATACTTTGCGATGC	
RM27412	12	0.40	177	TCATGATCACTAGACGGTGAGACG	GTGTTGCCCAAGGAATTGAGG	
RM27413	12	0.40	177	TCATGATCACTAGACGGTGAGACG	GTGTTGCCCAAGGAATTGAGG	
RM27414	12	0.40	182	GCATCAACGGTTCACATTCTAGG	ATTACACACCAACCCGCTAATGG	
RM1880	12	0.75	128	ACCACTAAATAAGCACATAC	GGCATCATACATTAAAATAC	
RM1080	12	0.91	201	AGAGCCCTCGTAAGCCAAAG	GGTCGTGAATCTCCTCCAAG	
RM3323	12	0.98	132	AGTCAGCACTGACGAGTCGAG	AGGGTGTATCCGACTCATCG	
IDR2475	12	2.03	119	TCCCCGACCAATCAACTAGAGCA	CCAATTCCGGCCAAATTCCGCTT	*
RM27505	12	2.19	287	TCCCAACTTTGATCATCTCC	TGATTGATGCTGTGTTCTCC	
RM5927	12	2.20	164	ATGGTCTCGTCTCTCATGTGC	AGCCCGGAAGTATGATCCC	
RM3747	12	2.31	146	AGCAATGCACTCCCTTGATC	GTCTTCCTCCTTGGTTTGG	
IDR2477	12	3.02	161	GCTAGGACCCCAAGACTCCCAAG	ACCAGCTGAGCTCGGATTTTGGC	*
RM6296	12	3.20	155	ATCTTGCCTCGCTAGGGTTAG	CCCACGTTTCTCTTGTCCTC	
RM27574	12	3.29	244	CAAGTGCAACAGGATGACTGC	TATACGCGGAAAGCGATAAGAGG	
RM27589	12	3.38	209	CTATTTGGGTTTCCCACGATAGC	TTTCCCATCCTCTCAAACTCAGC	
RM27593	12	3.43	114	CGTGCTGTCTCGGTCTCTCTCC	CAGTTACAGGAGGGAATGGATGC	
RM27627	12	3.84	246	CCAACGATAGTTTCTAGCTAGGTTCC	TGGAGGTAATAGCTCATCAGTTGC	
IDR1566	12	3.99	145	CATACTAGCCGAAACCACAACA	GGGCATGAGGTGTATGAATGTA	*
RM6998	12	4.75	213	AGATGATAAGCTTGCGGACC	ATGCAGATGAGTCCCTCCAC	
RM7619	12	4.83	196	ACTTGGTATGTATTGGCAGCG	GAGGCAATAGGAGGGGAGAG	
RM5317	12	5.04	153	AGCAAACGAGCATTCCGTG	CGCGTTGTGTGTGTTGG	
RM27723	12	5.47	210	GCCATGCCCTATATCAAAGTTGC	GCATAGAATTCGAAGCCATAGCC	
RM27724	12	5.51	184	CCTATGTGCACGTCCTTCATGC	GCAAACCAGAGAAGATCAGCAGAGG	
RM6905	12	6.75	189	AGCACTCATCGATGGTAGCAG	AGAACGGGACCTGAATTGTC	
RM7003	12	6.78	101	AGGCAGACATACAGCTTATAGGC	GCAAATGAACCCCTCTAGC	
RM27792	12	7.10	194	GAAGAAGAGAGACTAGGGAGAAGACG	CTTGTACCAGCAATTCTCTGTCC	
RM2935	12	7.43	166	ACAGCAAATTTGTTACTTATG	GCTATGTTTTTTTATAACG	
RM2529	12	7.57	135	ACATTAAAATCAGTGGGACTG	AGGCATTTCCTGATATGATC	
IDR1582	12	7.82	127	TGAAGACGTGGAAGAAATGAAA	TTTGCTTATTGATCTCTGTTGTT	*
ID12-44	12	8.80	231	TCATTTGTCGATTGCCGTCT	GATCTGCTTGGCCTGATCAAA	
RM1036	12	8.80	146	ACTCATTTGTCGATTGCCGTC	ATGGGAGGAGTGATCAAACG	
RM3246	12	9.10	192	AGCCACTCATATAAGCAAATG	GGTTAATGGTCAGAACCTG	
RM27900	12	9.57	255	CAAATATAACCGCATGGAGACACG	AGCAGTACTCCCTCCCTCCTTCC	
RM7195	12	9.90	139	ACGCGTGAGAGCTCCTAAAAG	CCTTGTGTAACTACCGCCC	
IDR1595	12	10.07	107	GTGAACCAAAGTGCAGTTGCTA	TACGGTGGAGATAAGGAGGATG	
RM6973	12	10.08	165	ACAACTCCAGCTTCGCCAAC	CGGCCACACCTAAATAAACG	
RM27920	12	10.14	267	AAAGCGAGAAATCCGGAGATGG	TCCTCTCTCAAATCTCCTCGAAGC	
RM27924	12	10.21	265	CTATGCTAGCAAACCAGCTATGG	TATTACAACGGGACGCTAATGG	
RM27943	12	10.86	269	TCAACAGTGGTTACGATCCTTCC	GAGGGACATGATGGTTTAGAAGC	
RM27971	12	12.24	216	ATTCCTGGACGAGGGTTTAACG	GAAGACGATTGGACTCCTAACAGC	
RM7102	12	13.21	170	ATAGGAGTGTTTAGAGTGCCA	CGGTTTGCTTATACATCAG	
RM28037	12	13.74	298	ATGAACGATGAGGGTTCAAAGG	ATCTGTCGTCCTGATGTACTTTGC	
IDR1614	12	13.80	97	AATGGAAAGTGTTCGACGAAAT	CCGTTGGATAGAAGCACT	
RM28053	12	14.34	116	TGAATCTTGGAACCCATACTCC	ATATGGCCGATAGCTCAAAGC	
RM28068	12	14.74	198	TCGTATCCCTCCTTCCTTTGTCC	CGCTTCACTTCATCTTCAACTTCAGG	
RM28070	12	14.93	199	AAGGCACCAGGAATATGACAAGC	GGGATGTGGGATTTGGAGAGG	
RM28102	12	15.91	169	CACTAATTCTTCGGCTCCACTTTAGG	GTGGAAGCTCCGAGAAAGTGC	
RM28112	12	16.28	284	TCAGCATCGAATTCACCACTTTGC	CGATCAAACCAACTTGCCAACC	
IDR1629	12	16.58	143	TACTCTCTGGTGGATGATGGTG	GAAGCAAAGAAAGGCTCTGAAA	
ID13-02	12	17.01	249	CATGGTGTCACATCCACCTAAA	GAGGAATTGGGTTTTGGAAAGG	
RM1261	12	17.54	169	AGTCCATGCCCAAGACACAAC	GTTACATCATGGGTGACCCC	
RM28166	12	17.61	195	TGCTTGCAAACATTGCTTCTGG	ACTGATGTACTGAACACGGGAAGG	
RM7344	12	17.95	185	AGACTGCCTCAGCCTTTTCAC	CGACGCCATATATGCCTTTC	
RM28195	12	18.11	96	CCTAGTTCAGCTCCTGCTTACC	CTCAGATGTAGGGAATGTTTGC	
RM1246	12	19.12	163	ACTCGATCCCCTAGCTCTC	CACCTCGTTCTCGATCC	
RM28305	12	19.96	142	GTCATCTTCGCAAATGGTGATGG	GGTCGTCGTGGTGTTATTCTTGG	
IDR1649	12	21.01	149	ACGAGATTGTTTGTAGGTGCAG	CGCTGTCTTATAGCATCTGTGC	
RM1986	12	21.25	156	ATAACGGAGGGAGTAGTTTTC	GAACCTACATATCGAGAGCA	
RM7376	12	23.48	195	ATCACCGTCACCTCTTAAGTC	GGTGGTTGTGTTCTGTTTGG	
RM3331	12	23.49	129	ACCTCCTCCATGAGCTAATGC	AGGAGGAGCGGATTTCTCTC	
RM6693	12	23.80	237	ATATGAGCTCTCTGAGTTGGACC	CGATACCCTTGAAGTTTCCG	
RM6947	12	24.01	155	ATTAAACGTCCACTGCTGGC	GCTAGGTTAGTGGTGCAGGG	
RM5479	12	24.41	152	ACTAAGCTCACCATAGCAATC	ATACACTTCTCCCCTCTCTG	
RM5481	12	24.41	152	ACTAAGCTCACCATAGCAATC	ATACACTTCTCCCCTCTCTG	
RM28585	12	24.42	200	TGTCGATCTCTCTTACTCTCTTGG	CATATACAACAGTCGCAGAAGC	

Marker name	Chr.	Position (Mb)[a]	Size (bp)[b]	Primer sequences (5' to 3') Forward	Reverse
RM5715	12	25.03	480	AAATCGTGTGGACAAGGTGAGC	CTTTCTTCACTAGAGCCACCTCAAGC
RM1300	12	26.00	165	ACAGCCATGAATGTTGGCTAC	GCCATGTCCATTTATGGTGC
RM1226	12	27.34	103	ATCCCTCACCCTCACTCTCAC	CTTGTTGCTTGTGCTGTCC
RM1227	12	27.34	137	ATGGTAGAGACGAGAGATGG	GGACCACTCCAACAATTTTA
RM2197	12	27.38	161	ACTGAGAACTTTAATCATCG	GAACAACTTTGAAGAGAAAC
IDR1672	12	27.52	146	AGCCACACATACAGCCACATAA	AAGAGGAATTGGAATTGGACCT

[a]Map positions (in Mb) of markers based on the IRGSP 1.0 rice reference genome (http://rapdb.dna.affrc.go.jp/).

[b]Expected PCR product size (in bp) in Nipponbare.

[c]Primer sequences for the SNP marker GS3_ID001_3:5′-GCAGGCTGGCTTACTCCCTT-3′ (forward, specific to Nipponbare/Koshihikari allele), 5′-TTTTTTTTTTTTTTTTTTTTGCAGGCTGGCTTACTCTGTG-3′ (forward, specific to alternative allele), 5′-ACAACTGTATATATATTTCTTGCAGGGT-3′ (reverse).

[d]Markers used exclusively in the analysis of sub-CSSLs.

Supplemental Table 2. List of primers used for SNP genotyping in sub-CSSLs and their derived progeny lines.

Marker name	Chr.	Position (bp)	Allele1	Allele2	Allele 1–specific primer[b]	Allele 2–specific primer[b]	Locus-specific primer
KA2950	7	68,275	T	G	GTTAAGACTTATGGGTTTTTTCACAACCA	TTAAGACTTATGGGTTTTTTCACAACCC	TTTTATTCATTTTTAACCTAAGAATATATGGGAGTTAAGT
KA1891	7	2,087,168	A	G	TCACAGCTACCATGAGGCG	CTTCACAGCTACCATGAGGCA	GGTCAAGAGTGGGGAGATGGT
KA1894	7	2,651,062	T	G	GTTAAAAAGTCAATTGCGTCCTACCC	GTTAAAAAGTCAATTGCGTCCTACCA	ACGTATGTGTACAAATTAAGTACTCCCTTCG
KA1896	7	2,865,195	A	G	CCATGTCTTCATCTCCGTTTCAATACA	CATGTCTTCATCTCCGTTTCAATACG	AGGGATCCGTAGCTAGCTAGGA
KA1898	7	2,922,713	G	C	TTGGTAAAATTACGATGGCATGAATCC	TTGGTAAAATTACGATGGCATGAATCG	TCCACCAGTGTAAATACATGATTTTACTAGGC
KA1905	7	3,719,818	A	G	GCTCCGCCACTGTATATAATCATGTAG	GCTCCGCCACTGTATATAATCATGTAA	CCTTCTCTAGTTTATATCAGAATTTGAAGGGTAGTT
KA2961	7	4,539,307	G	T	TCTAGGATAACACTAGTCGAATCGACTATC	CTCTAGGATAACACTAGTCGAATCGACTATA	TCAGCCCAAAAGGTGGATCCT
FA1662	12	1,470	C	G	ACTAACCAACACAGCGGAGAATC	CTAACCAACACAGCGGAGAATG	GATGCTGCATACATGTTCTGTTCTGT
KA2411	12	2,686,857	C	T	CCACTAGCAGAAGGGATCCTG	CCCACTAGCAGAAGGGATCCTA	TCCTGGGTCACACTGCTCC
KA3537	12	5,774,262	A	G	CCAAGGCATTTTCCTATGGCTAGA	CAAGGCATTTTCCTATGGCTAGG	CGGTCGATTCTCGATACATGACTGAAA
KA3259	12	7,010,394	A	G	CCGCAGCTACTGTAAATATGTCGATAA	CCGCAGCTACTGTAAATATGTCGATAG	TTGCATGTGGTAGAGCCTCGA
KA3544	12	7,987,137	T	G	GTAAATCAATACTCCCTCCATCCCA	GTAAATCAATACTCCCTCCATCCCC	GTTTTGTACAAAAACCAATCCTACCTGGA
KA3264	12	8,917,711	G	A	TTGGCGATTGGCGAGGTC	TTGGCGATTGGCGAGGTT	TTGCGTCTTTGTCGGTTCGC
KA1138	1	1,043,843	C	T	ACACAGCACATTGACATGTACAGT	CACAGCACATTGACATGTACAGC	TTTAAAAAAAGCGACAAGAATCAGGTGAAGT
KA1140	1	1,286,084	T	C	CAACGACAACTAAACCGGGAC	CCAACGACAACTAAACCGGGAT	TCTGTAAGGACGTGGCGCA
KA1141	1	1,427,907	A	C	CCAGTTCACCATTACAGCATGTG	CCAGTTCACCATTACAGCATGTT	CTGATATACGGTCGTTCATCAGCTTCT
FA6836	1	1,693,197	G	A	TGTTCATAGGGGTAGGATGCG	TGTTCATAGGGGTAGGATGCA	AAACGCGGACACTCATAACG
FA6837	1	1,719,531	T	C	CTCCACTACCATCGGCCA	CTCCACTACCATCGGCCG	GCAGCAGTGTTTTGAGGAGA
KA4073	1	1,949,598	A	T	CCTATAAATATGTGCACCGCTCTTGT	CCTATAAATATGTGCACCGCTCTTGA	TGTGTTCCTCGGTGGGTTTCA
KA1147	1	2,073,706	A	G	CTTAACGTCATCTTCCTGAACTGAAAG	ACTTAACGTCATCTTCCTGAACTGAAAA	ACGAAAACGACCAACATATATCCAGTTCT
KA1149	1	2,241,746	T	C	TCAACCTCCCGAATGAATGGG	TTTATATCAACCTCCCGAATGAATGGA	CTCCTCTAGCTAGCTGGGCA
KA2480	1	2,289,661	C	T	GACCTAGCTGATTGCGTTGTG	TGACCTAGCTGATTGCGTTGTA	TGCACGTTAATATATTGGAAGAAAAAAGTGTTTGT

Chr., chromosome number.

[a]Position of the SNPs in the 'Nipponbare' reference genome (IRGSP-1.0)

[b]Tag sequences for the fluorescent label are not shown.

Supplemental Table. 3. Mean grain length and grain width in 39 BKK-$BC_{3\text{-}5}F_2$ populations, along with graphical genotypes of their parental $BC_{3\text{-}5}F_1$ plants.

Line name			13-2403	13-2404	13-2405	13-2406	13-2407	13-2408	13-2409	13-2410	13-2411	13-2412	13-2413	13-2414	13-2415	13-2416	13-2417	13-2418	13-2419	13-2420	13-2421	13-2422	13-2423	13-2424	13-2425	13-2426	13-2427	13-2428	13-2429	13-2430	13-2431	13-2432	13-2433	13-2434	13-2435	13-2436	13-2437	13-2438	13-2439	13-2440	13-2441	Positions of QTL[c] L	Positions of QTL[c] W
Grain length	Mean±SD (mm)		6.82±0.31	6.85±0.32	7.01±0.32	6.79±0.33	6.90±0.33	6.88±0.33	6.96±0.32	6.91±0.30	6.96±0.31	7.09±0.32	7.08±0.35	7.07±0.32	6.96±0.30	6.95±0.31	7.00±0.37	6.73±0.33	6.73±0.32	6.75±0.34	7.04±0.31	6.95±0.32	7.07±0.32	7.04±0.34	6.98±0.34	7.17±0.33	6.83±0.31	6.77±0.30	6.95±0.33	6.80±0.30	7.35±0.37	6.95±0.37	6.97±0.36	7.01±0.37	7.24±0.35	7.05±0.35	6.91±0.33	7.07±0.32	6.97±0.32	6.86±0.31	6.91±0.31		
	QTL detection (Y/N)[a]		N	Y	N	Y	Y	N	Y	Y	N	Y	Y	Y	Y	N	Y	Y	Y	Y	Y	Y	Y	Y	Y	Y	N	Y	Y	N	Y	Y	Y	N	Y	Y	Y	Y	N	N	N		
Grain width	Mean±SD (mm)		3.36±0.16	3.35±0.16	3.32±0.14	3.33±0.16	3.35±0.15	3.25±0.15	3.23±0.16	3.33±0.14	3.31±0.14	3.37±0.14	3.47±0.15	3.44±0.15	3.31±0.14	3.35±0.14	3.17±0.16	3.29±0.16	3.45±0.15	3.26±0.17	3.15±0.15	3.43±0.16	3.45±0.14	3.45±0.14	3.25±0.16	3.19±0.14	3.32±0.14	3.34±0.15	3.44±0.14	3.37±0.14	3.25±0.17	3.33±0.17	3.35±0.16	3.40±0.15	3.38±0.14	3.27±0.14	3.24±0.14	3.39±0.14	3.31±0.15	3.26±0.16	3.36±0.17		
	QTL detection (Y/N)[a]		N	Y	Y	Y	Y	Y	Y	Y	Y	Y	Y	Y	Y	N	Y	Y	Y	Y	Y	Y	Y	Y	Y	Y	Y	N	Y	Y	Y	Y	Y	N	Y	Y	Y	Y	Y	N	N		
Genotypes[b]																																											L	W
Marker	Chr. No.	Position (Mb)																																										
RM7278	1	1.79	H	A	A	H	-	A	A	A	A	A	H	A	A	A	A	A	A	-	A	A	A	A	A	A	A	A	A	A	A	A	A	A	A	A	A	A	A	A	A		1/3 (red)
RM3233	1	5.06	H	-	A	A	A	A	A	A	A	A	A	A	A	A	A	A	A	A	A	A	A	A	A	A	A	A	A	A	A	A	A	A	A	A	A	A	A	A	A		
RM3234	1	7.57	H	H	A	A	A	A	A	A	A	A	A	A	A	A	A	A	A	A	A	A	A	A	A	A	A	A	A	A	A	A	A	A	A	A	A	A	A	A	A		
RM1287	1	10.84	H	H	A	A	A	A	A	A	A	A	A	A	A	A	A	A	A	A	A	A	A	A	A	A	A	A	A	A	A	A	A	A	A	A	A	A	A	A	A		
RM7075	1	15.12	A	H	A	A	A	A	A	A	A	A	A	A	A	A	A	A	A	A	A	A	A	A	A	A	A	A	A	A	A	A	A	A	A	A	A	A	A	A	A		
RM6092	1	19.12	A	H	H	A	A	A	A	A	A	A	A	A	A	A	A	A	A	A	A	A	A	A	A	A	A	A	A	A	A	A	A	A	A	A	A	A	A	A	A		
RM5718	1	22.93	A	A	H	A	A	A	A	A	A	A	A	A	A	A	A	A	A	A	A	A	A	A	A	A	A	A	A	A	A	A	A	A	A	A	A	A	A	A	A		
RM3475	1	26.04	A	A	H	A	A	A	A	A	A	A	A	A	A	A	A	A	A	A	A	A	A	A	A	A	A	A	A	A	A	A	A	A	A	A	A	A	A	A	A		1/1 (blue)
RM1297	1	28.61	A	A	H	H	A	A	A	A	A	A	A	A	A	A	A	A	A	A	A	A	A	A	A	A	A	A	A	A	A	A	A	A	A	A	A	A	A	A	A	1/2 (red)	
RM1183	1	30.98	A	A	A	H	A	A	A	A	A	A	A	A	A	A	A	A	A	A	A	A	A	A	A	A	A	A	A	A	A	A	A	A	A	A	A	A	A	A	A		
RM6703	1	34.52	A	A	A	H	A	A	A	A	A	A	A	A	A	A	A	A	A	A	A	A	A	A	A	A	A	A	A	A	A	A	A	A	A	A	A	A	A	A	A	1/1 (blue)	1/1 (blue)
RM5448	1	37.40	A	A	A	H	-	A	A	A	A	A	A	A	A	A	A	A	-	A	A	A	A	A	A	A	A	A	A	A	A	A	A	A	A	A	A	A	A	A	A		
RM6333	1	38.01	-	-	-	-	H	-	-	-	-	-	-	-	-	-	-	-	-	-	-	-	-	-	-	-	-	-	-	-	-	-	-	-	-	-	-	-	-	-	-		
RM8099	1	41.10	A	A	A	H	H	A	A	A	A	A	A	A	A	A	A	A	A	H	A	A	A	A	A	A	A	A	A	A	A	A	A	A	A	A	A	A	A	A	A	1/3 (blue)	1/3 (blue)
RM6840	1	43.17	A	A	A	A	H	A	A	A	A	A	A	A	A	A	A	A	A	H	A	A	A	A	A	A	A	A	A	A	A	A	A	A	A	A	A	A	A	A	A		
RM6842	2	0.12	A	A	A	A	A	H	A	A	A	A	A	A	A	A	A	A	A	A	A	A	H	A	H	A	A	A	A	A	A	A	A	A	A	A	A	A	A	A	A	1/3 (blue)	3/3 (blue)
RM3188	2	3.45	A	A	A	A	A	H	A	A	A	A	A	A	A	A	A	A	A	A	A	A	A	A	A	A	A	A	A	A	A	A	A	A	A	A	A	A	A	A	A		
RM5340	2	7.48	A	A	A	A	A	H	A	A	A	A	A	A	A	A	A	A	A	A	A	A	A	A	A	A	A	A	A	A	A	A	A	A	A	A	A	A	A	A	A		
RM1313	2	11.26	A	A	A	A	A	H	H	A	A	A	A	A	A	A	A	A	A	A	A	A	A	A	A	A	A	A	A	A	A	A	A	A	A	A	A	A	A	A	A		
RM3443	2	14.33	A	A	A	A	A	-	H	A	A	A	A	A	A	A	A	A	A	A	A	A	A	A	A	A	-	A	A	A	A	A	A	A	A	A	A	A	A	A	A		
RM1211	2	18.46	A	A	A	A	A	A	H	A	A	A	A	A	A	A	A	A	A	A	A	A	A	A	A	A	H	A	A	A	A	A	A	A	A	A	A	A	A	A	A		2/2 (blue)
RM3688	2	22.40	A	A	A	A	A	A	H	-	A	A	A	A	H	A	A	A	A	A	A	A	A	A	A	A	-	A	A	A	A	A	A	A	A	A	A	A	A	A	A		
RM3515	2	24.02	A	A	A	A	A	A	-	H	A	A	A	A	-	A	A	A	A	A	A	A	A	A	A	A	A	A	A	A	A	A	A	A	A	A	A	A	A	A	A		
RM1920	2	25.47	A	A	A	A	A	A	H	-	A	A	A	A	H	A	A	A	A	A	A	A	A	A	A	A	A	A	A	A	A	A	A	A	A	A	A	A	A	A	A		
RM5631	2	28.27	A	A	A	A	A	A	H	A	A	A	A	A	H	A	A	A	A	A	A	A	A	A	A	A	A	A	A	A	A	A	A	A	A	A	A	A	A	A	A		
RM5472	2	30.64	A	A	A	A	A	A	H	A	A	A	A	A	H	A	-	-	A	A	A	A	A	A	A	A	A	A	A	A	A	A	A	A	A	A	A	A	A	A	A		
RM7388	2	35.48	A	A	A	A	A	A	H	A	A	A	A	A	H	A	H	H	A	A	A	A	A	A	A	A	A	A	A	A	A	A	A	A	A	A	A	A	A	A	A		
RM3413	3	0.37	A	A	A	A	A	A	A	-	A	A	A	H	A	A	A	A	A	A	A	A	A	A	H	A	A	A	H	A	A	A	A	A	A	A	A	A	A	A	A	2/3 (red)	2/3 (red)
RM3387	3	0.38	A	A	A	A	A	A	A	H	A	A	A	A	A	A	A	A	A	A	A	A	A	A	A	A	A	A	A	A	A	A	A	A	A	A	A	A	A	A	A	1/1 (red)	
RM5474	3	3.80	A	A	A	A	A	A	A	H	A	A	A	A	A	A	A	A	A	A	A	A	A	A	A	A	A	A	A	A	A	A	A	A	A	A	A	A	A	A	A		
RM3766	3	6.93	A	A	A	A	A	A	A	H	A	A	A	A	A	A	A	A	A	A	A	A	A	A	A	A	A	A	A	A	A	A	A	A	A	A	A	A	A	A	A		
RM4321	3	10.17	A	A	A	A	A	A	A	H	A	A	A	A	A	A	A	A	A	A	A	A	A	A	A	A	A	A	A	A	A	A	A	A	A	A	A	A	A	A	A		1/1 (blue)
RM6676	3	14.49	A	A	A	A	A	A	A	H	H	A	A	A	A	A	A	A	A	A	A	A	A	A	A	A	A	A	A	A	A	A	A	A	A	A	A	A	A	A	A		
RM1334	3	17.42	A	A	A	A	A	A	A	A	H	H	A	A	A	A	A	A	A	A	A	A	A	A	A	A	A	A	A	A	A	A	A	A	A	A	A	A	A	A	A		
RM6832	3	22.40	A	A	A	A	A	A	A	A	H	H	A	A	A	A	A	A	A	A	A	A	A	A	A	A	A	A	A	A	A	A	A	A	A	A	A	A	A	A	A		2/2 (red)
RM2334	3	26.75	A	A	A	A	A	A	A	A	H	H	A	A	A	A	A	A	A	A	A	A	A	A	A	A	A	A	A	A	A	A	A	A	A	A	A	A	A	A	A	1/2 (red)	
RM1352	3	32.35	A	A	A	A	A	A	A	H	-	H	A	A	A	A	A	A	A	A	A	A	A	A	A	A	A	A	A	A	A	A	A	A	A	A	A	A	A	A	A		
RM6135	3	35.26	A	H	A	A	A	A	A	A	A	H	A	A	A	A	A	A	A	A	A	A	A	A	A	A	A	A	A	A	A	A	A	A	A	A	A	A	A	A	A	1/2 (red)	
RM7324	3	36.35	A	-	A	A	A	A	A	H	A	H	A	A	A	A	A	A	A	A	A	A	A	A	A	A	A	A	A	A	A	A	A	A	A	A	A	A	A	A	A		1/2 (blue)
RM16284	4	0.52	A	A	A	A	A	-	-	A	A	A	A	H	A	A	A	A	A	A	A	A	A	A	A	A	A	A	A	A	A	A	A	A	A	A	A	A	A	A	A		
RM7200	4	4.04	A	A	A	A	A	A	A	A	A	A	A	H	A	A	A	A	A	A	A	A	A	A	A	A	A	A	A	A	A	A	A	A	A	A	A	A	A	A	A		
RM16534	4	8.04	A	A	A	A	A	A	A	A	A	A	A	H	H	A	A	A	A	A	A	A	A	A	A	A	A	A	A	A	A	A	A	A	A	A	A	A	A	A	A	2/2 (blue)	
RM7279	4	13.77	A	A	A	A	A	-	A	A	A	A	A	H	H	A	A	A	A	A	A	A	A	A	A	A	A	A	A	A	A	A	A	A	A	A	A	A	A	A	A		
RM16757	4	17.07	A	A	A	A	A	A	A	A	A	A	A	H	H	A	A	A	A	A	A	A	A	A	A	A	A	A	A	A	A	A	A	A	A	A	A	A	A	A	A		
RM5979	4	20.77	A	A	A	A	A	A	A	-	A	A	A	A	H	A	A	A	A	A	A	A	A	A	A	A	A	A	A	A	A	A	A	A	A	A	A	A	A	A	A		
RM3839	4	24.09	A	A	A	A	A	A	A	H	-	-	A	A	H	A	A	A	A	A	A	A	A	A	A	A	A	A	A	A	A	A	A	A	A	A	A	A	A	A	A		1/2 (red)
RM3820	4	27.81	A	A	A	A	A	A	A	A	A	A	A	A	H	A	A	A	A	A	A	A	A	A	A	A	A	A	A	A	A	A	A	A	H	A	A	A	A	A	A		
RM7208	4	30.14	A	A	A	A	A	A	A	A	A	A	A	A	-	-	A	A	A	A	A	A	A	A	A	A	A	A	A	A	A	A	A	A	H	A	A	A	A	A	A		
RM3836	4	31.81	A	A	A	A	A	A	A	A	A	A	A	A	H	H	A	A	A	A	A	A	A	A	A	A	A	A	A	A	A	A	A	A	-	A	A	A	A	A	A		
RM5608	4	35.36	A	A	A	A	A	A	A	A	A	A	A	A	-	H	A	A	A	A	A	A	A	A	A	A	A	A	A	A	A	A	A	A	H	H	H	A	A	A	A		
RM5816	5	0.28	A	A	A	A	A	A	A	A	A	A	A	A	A	A	H	A	A	A	A	A	A	A	A	A	A	A	A	A	H	H	A	A	A	H	A	A	A	A	A		
RM2488	5	2.15	-	-	-	-	-	-	-	-	-	-	-	-	-	-	-	-	-	-	-	-	-	-	-	-	-	-	-	-	H	-	-	-	-	-	-	-	-	-	-		
RM1089	5	5.36	A	A	A	A	A	A	A	A	A	A	A	A	A	A	H	A	A	A	H	-	A	A	A	H	A	A	A	A	H	A	A	A	A	A	A	A	A	A	A	4/4 (red)	4/4 (blue)
RM5844	5	9.15	A	A	A	A	A	A	A	A	A	A	A	A	A	A	H	H	A	A	A	A	A	A	A	H	A	A	A	A	A	A	A	A	A	A	A	A	A	A	A		
RM6742	5	14.88	A	A	A	A	A	A	A	A	A	A	A	A	A	A	H	H	A	A	A	A	A	A	A	H	A	A	A	A	A	A	A	A	A	A	A	A	A	A	A		
RM6621	5	18.84	A	A	A	A	A	A	A	A	A	A	A	A	A	A	A	H	H	A	A	A	A	A	A	A	A	A	A	A	A	A	A	A	A	A	A	A	A	A	A		
RM4501	5	22.11	A	A	A	A	A	A	A	A	A	A	A	A	A	A	A	H	H	A	A	A	A	A	A	A	A	A	A	H	A	A	A	A	A	A	A	A	A	A	A		2/3 (red)
RM3348	5	25.15	A	A	A	A	A	A	A	A	A	A	A	A	A	A	A	A	H	A	A	A	A	A	A	A	A	A	A	H	A	A	A	A	A	A	A	A	A	A	A	1/2 (blue)	
RM3664	5	27.86	-	-	-	-	-	-	-	-	-	-	-	-	-	-	-	-	H	-	-	-	-	-	-	-	-	-	-	-	-	-	-	-	-	-	-	-	-	-	-		
RM3286	5	29.54	A	A	A	A	A	A	A	A	A	A	A	A	A	A	A	A	A	A	A	A	A	A	A	A	A	A	A	H	A	A	A	A	A	A	A	A	A	A	A		
RM6467	6	0.22	A	A	A	H	A	A	A	A	A	A	A	A	A	A	H	H	H	H	A	H	A	A	A	A	A	A	A	A	A	H	A	A	A	A	A	A	A	A	A	6/7 (blue)	
RM8125	6	3.17	A	A	A	H	A	A	A	A	A	A	A	A	A	A	H	H	H	H	A	-	A	A	H	A	A	A	-	A	A	-	A	-	-	-	-	A	A	A	A		5/6 (blue)
RM5754	6	5.23	A	A	A	H	A	A	A	A	A	A	A	A	A	A	H	H	H	H	A	A	A	A	H	A	A	A	H	A	A	H	A	H	-	H	H	A	A	A	A		
RM2615	6	5.96	A	A	A	H	A	A	A	A	A	A	A	A	A	A	-	H	H	H	A	A	A	A	H	A	A	A	-	A	A	-	A	-	A	-	-	A	A	A	A		
RM5963	6	8.82	A	A	A	H	A	A	A	A	A	A	A	A	A	A	A	H	A	H	H	A	A	A	H	A	A	A	A	A	A	A	A	A	A	H	H	A	A	A	A		
RM19902	6	12.09	A	A	A	A	A	A	A	A	A	A	A	A	A	A	A	A	A	A	H	A	A	A	H	A	A	A	A	A	A	A	A	A	A	H	H	A	A	A	A		
RM20023	6	15.84	A	A	A	A	A	A	A	A	A	A	A	A	A	A	A	A	A	A	H	A	A	A	H	A	A	A	A	A	A	A	A	A	A	A	A	A	A	A	A		
RM20163	6	19.75	A	A	A	A	A	A	A	A	A	A	A	A	A	A	A	A	A	A	H	A	A	A	H	A	A	A	A	A	A	A	A	A	A	A	A	A	A	A	A		
RM3827	6	22.30	A	A	A	A	A	A	A	A	A	A	A	H	A	A	A	A	A	A	H	H	A	A	H	A	A	A	A	A	A	A	A	A	A	A	A	A	A	A	A		(blue)
RM6395	6	26.00	A	A	A	A	A	A	A	A	A	A	A	H	A	A	A	A	A	A	H	H	A	A	H	A	A	A	A	A	A	A	A	A	A	A	A	A	A	A	A	3/4 (red)	
RM3138	6	28.47	A	A	A	A	A	A	A	A	A	A	A	H	A	A	A	A	A	A	A	H	A	A	-	A	A	A	A	A	A	A	A	A	A	A	A	A	A	A	A		1/2 (red)
RM3394	7	0.65	A	A	A	A	A	A	A	A	A	A	A	A	A	A	A	A	A	A	A	-	H	A	A	A	A	A	A	A	A	A	A	A	A	A	A	A	H	A	A		
RM3325	7	2.22	A	A	A	A	A	A	A	A	A	A	A	A	A	A	A	A	A	A	A	-	H	A	A	A	A	A	A	A	A	A	A	A	A	A	A	A	H	A	A		
RM6776	7	6.84	A	A	A	A	A	A	A	A	A	A	A	A	A	A	A	A	A	A	A	A	H	H	A	A	A	A	A	A	A	A	A	A	H	A	A	A	H	A	H		
RM3635	7	11.09	A	A	A	A	A	A	A	A	A	A	A	A	A	A	A	A	A	A	A	A	H	H	A	A	A	A	A	A	A	A	A	A	H	A	A	A	H	A	A		2/4 (red)
RM3670	7	13.44	A	A	A	A	A	A	A	A	A	A	H	A	A	A	A	A	A	A	A	A	-	-	A	A	A	A	A	A	A	A	A	A	-	A	A	A	-	A	A		
RM5543	7	15.75	A	A	A	A	A	A	A	A	A	A	-	A	A	A	A	A	A	A	A	A	H	H	A	A	A	A	A	A	A	A	A	A	-	A	A	A	H	A	A		
RM6767	7	17.47	A	A	A	A	A	A	A	A	A	A	H	A	A	A	A	A	A	A	A	A	-	-	A	A	A	A	A	A	A	A	A	A	A	A	A	A	-	A	A	1/1 (red)	
RM7087	7	19.36	A	A	A	A	A	A	A	A	A	A	-	A	A	A	A	A	A	A	A	A	H	H	A	A	A	A	A	A	A	A	A	A	A	A	A	A	A	A	A	2/2 (red)	2/2 (red)
RM3404	7	20.11	A	A	A	A	A	A	A	A	A	A	H	A	A	A	A	A	A	A	A	A	-	-	A	A	A	A	A	A	A	A	A	A	A	A	A	A	A	A	A		
RM6885	7	23.01	A	A	A	A	A	A	A	A	A	A	-	A	A	A	A	A	A	A	A	A	H	H	A	A	A	A	A	A	A	A	A	A	A	A	A	A	A	A	A		
RM5847	7	23.65	A	A	A	A	A	A	A	A	A	A	H	A	A	A	A	A	A	A	A	A	-	-	A	A	A	A	A	A	A	A	A	A	A	A	A	A	A	A	A		
RM5455	7	26.46	A	A	A	A	A	A	A	A	A	A	A	A	A	A	A	A	A	A	A	A	-	H	A	A	A	A	A	A	A	A	A	A	A	A	A	A	A	A	A		
RM7601	7	29.04	A	A	A	A	A	A	A	A	A	A	A	A	A	A	A	A	A	A	A	A	A	H	A	A	A	A	A	A	A	A	A	A	A	A	A	A	A	A	H		
RM6369	8	0.13	A	A	A	H	A	A	A	A	A	A	H	A	A	A	A	A	A	A	A	A	H	A	H	A	A	A	A	A	A	A	A	A	A	A	A	A	H	A	A		1/5 (red)
RM6208	8	5.79	A	A	A	A	A	A	A	A	A	A	A	A	A	A	A	A	A	A	A	A	A	A	A	H	A	-	A	A	A	A	A	A	A	A	A	A	-	A	A		
RM3214	8	8.57	A	A	A	A	A	A	A	A	A	A	A	A	A	A	A	A	A	A	A	A	A	A	A	H	A	A	A	A	A	A	A	A	A	A	A	A	A	A	A		
RM3383	8	13.49	A	A	A	A	A	A	A	A	A	A	A	A	A	A	A	A	A	A	A	A	A	A	A	H	A	A	A	A	A	A	A	A	A	A	A	A	A	A	A		
RM6215	8	19.07	A	A	A	A	A	A	A	A	A	A	A	A	A	A	A	A	A	A	A	A	A	A	A	H	A	A	A	A	A	A	A	A	A	A	A	A	A	A	A		1/1 (red)
RM8265	8	20.49	-	-	-	-	-	-	-	-	-	-	-	-	-	-	-	-	-	-	-	-	-	-	-	H	-	-	-	-	-	-	-	-	-	-	-	-	-	-	-		
RM5485	8	24.07	A	A	A	A	A	A	A	A	A	A	A	A	A	A	A	A	A	A	A	A	A	A	A	H	A	A	A	A	A	A	A	A	A	A	A	A	A	A	A		
RM4154	8	28.25	A	A	A	A	A	A	A	A	A	A	A	A	H	A	A	A	A	A	A	A	A	A	A	H	A	A	A	A	A	A	A	A	A	A	A	A	A	A	A	1/2 (red)	
RM8219	9	1.55	A	A	A	A	A	A	A	A	A	A	A	A	A	A	A	A	A	A	A	A	A	A	A	A	H	H	H	A	A	A	A	A	-	A	A	A	A	A	A		
RM5899	9	5.12	A	A	A	A	A	A	A	A	A	A	A	A	A	A	A	A	A	A	A	A	A	A	A	A	H	H	H	A	A	A	A	A	A	H	A	A	A	A	A	1/4 (blue)	
RM1328	9	9.21	A	A	A	A	A	A	A	A	A	A	A	A	A	A	A	A	A	A	A	A	A	A	A	A	H	H	H	A	A	A	A	A	A	H	A	A	A	A	A		
RM5657	9	14.37	A	A	A	A	A	A	H	A	A	A	A	A	A	A	A	A	A	A	A	A	A	A	A	A	H	A	A	H	H	A	A	A	A	A	A	A	A	A	A		
RM7048	9	16.94	A	A	A	A	A	A	H	A	A	A	A	A	A	A	A	A	A	A	A	A	A	A	A	A	H	A	A	H	H	A	A	A	A	A	A	A	A	A	A	1/4 (blue)	
RM5786	9	19.79	A	A	A	A	A	A	A	A	A	A	A	A	A	A	A	A	A	A	A	A	A	A	A	A	A	A	A	H	H	A	A	A	A	A	A	A	A	A	A		1/2 (blue)
RM6797	9	22.71	A	A	A	A	A	A	A	A	A	A	A	A	A	A	A	A	A	A	A	A	A	A	A	A	A	A	A	H	H	A	A	A	A	A	A	A	A	A	A		
RM7492	10	0.05	A	A	A	A	A	A	A	A	A	A	A	A	A	A	A	A	A	A	A	A	A	A	A	A	A	A	A	A	H	H	A	A	A	A	A	A	A	A	A		
RM25024	10	3.59	A	A	A	A	A	A	A	A	A	A	A	A	A	A	A	A	A	A	A	A	A	A	A	A	A	A	A	A	H	H	A	A	A	A	A	A	A	A	A		
RM25158	10	7.94	A	A	A	A	A	A	A	A	A	A	A	A	A	A	A	A	A	A	A	A	A	A	A	A	A	A	A	A	H	H	A	A	A	A	A	A	A	A	A		
RM4455	10	11.74	A	A	A	A	A	A	A	A	A	A	A	A	A	A	A	A	A	A	A	A	A	A	H	A	A	A	H	A	H	A	H	A	A	A	A	A	A	A	A		1/5 (red)
RM5620	10	17.47	A	A	A	A	A	A	A	A	A	A	A	A	A	A	A	A	A	A	A	A	A	A	H	A	A	A	H	A	H	A	H	H	A	A	A	A	A	A	A	1/5 (red)	
RM4477	10	21.17	A	A	A	A	A	A	A	A	A	A	A	A	A	A	A	A	A	A	A	A	A	A	A	A	A	A	A	A	H	A	A	H	H	H	H	A	A	A	A		
RM6673	10	23.08	A	A	A	A	A	A	A	A	A	A	A	A	A	A	A	A	A	A	A	A	A	A	A	A	A	A	A	A	A	H	A	H	H	H	H	A	A	A	A		
RM7203	11	1.08	A	A	A	A	A	A	A	A	A	A	A	A	A	A	H	A	A	A	A	A	A	A	A	A	A	A	A	A	A	A	H	A	A	A	A	A	A	A	A		1/2 (blue)
RM5599-1	11	3.83	-	-	-	-	-	-	-	-	-	-	-	-	-	-	-	-	-	-	-	-	-	-	-	-	-	-	-	-	-	-	-	-	H	-	-	-	-	-	-		
RM4469	11	6.26	A	H	A	A	A	A	A	A	A	A	A	A	A	A	A	A	A	A	A	A	A	A	A	A	A	A	A	A	A	A	A	A	H	H	H	A	A	A	A		
RM3701	11	8.11	-	-	-	-	-	-	-	-	-	-	-	-	-	-	-	-	-	-	-	-	-	-	-	-	-	-	-	-	-	-	-	-	H	-	-	-	-	-	-		
RM7463	11	10.20	A	A	A	A	A	A	A	A	A	A	A	A	A	A	A	A	A	A	A	A	A	A	A	A	A	A	A	A	A	A	A	A	H	H	H	A	A	A	A		
RM5824	11	14.53	A	A	A	A	A	A	A	A	A	A	A	A	A	A	A	A	A	A	A	A	A	A	A	A	A	A	A	A	A	A	A	A	H	H	H	A	A	A	A	1/3 (red)	
RM1355	11	17.67	A	A	A	A	A	A	A	A	A	A	A	A	A	A	A	A	A	A	A	A	A	A	A	A	A	A	A	A	A	A	A	A	H	H	H	A	A	A	A		
RM6534	11	21.52	A	A	A	A	A	A	A	A	A	A	A	A	A	A	A	A	A	A	A	A	A	A	A	A	A	A	A	A	A	A	A	A	H	H	H	A	A	A	A	1/3 (red)	
RM2191	11	25.13	A	A	A	A	A	A	A	A	A	A	A	A	A	A	A	A	A	A	A	A	A	A	A	A	A	A	A	A	A	A	A	A	H	H	A	A	A	A	A		
RM6094	11	28.93	A	A	A	A	A	A	A	A	A	A	A	A	A	A	A	A	A	A	A	A	A	A	A	A	A	A	A	A	A	A	A	A	H	H	A	A	A	A	A		
RM1080	12	0.91	A	A	A	A	A	A	A	A	A	A	A	A	A	A	A	A	A	A	A	A	A	A	A	A	A	A	A	A	A	A	A	A	A	A	A	H	H	A	A		1/2 (red)
RM3323	12	0.98	A	A	A	A	A	A	A	H	A	H	A	A	H	A	A	A	A	A	A	A	A	A	A	A	A	A	A	A	A	A	A	A	A	A	A	-	-	A	A		
RM5317	12	5.04	A	A	A	A	A	A	A	-	A	A	A	A	-	A	A	A	A	A	A	A	A	A	A	A	A	A	A	A	A	A	A	A	A	A	A	H	H	A	A	1/2 (red)	
RM7195	12	9.90	A	A	A	A	A	A	A	A	A	A	A	A	A	A	A	A	A	A	A	A	A	A	A	A	A	A	A	A	A	A	A	A	A	A	A	A	H	H	A		
RM28070	12	14.93	A	A	A	A	A	A	A	A	A	A	A	A	A	A	A	A	A	A	A	A	A	A	A	A	A	A	A	A	A	A	A	A	A	A	A	A	H	H	A		
RM1246	12	19.12	A	A	A	A	A	A	A	A	A	A	A	A	A	A	A	A	A	A	A	A	A	A	A	A	A	A	A	A	A	A	A	A	A	A	A	A	H	H	A		
RM1986	12	21.25	A	A	A	A	A	A	A	A	A	A	A	A	A	A	A	A	A	A	A	A	A	A	A	A	A	A	A	A	H	H	A	A	A	A	A	A	-	-	H	1/3 (red)	
RM3331	12	23.49	A	A	A	A	A	A	A	A	A	A	A	A	A	A	A	A	A	A	A	A	A	A	A	A	A	A	A	A	A	A	A	A	A	A	A		H	A	H		
RM1227	12	27.34	A	A	A	A	A	A	A	A	A	A	A	A	A	A	A	A	A	A	A	A	A	A	A	A	A	A	A	A	A	A	A	A	H	H	A	A	H	A	H		

[a]Y indicates the presence, and N the absence, of a QTL.

[b]A indicates a homozygous Koshihikari allele; H indicates a heterozygous allele.

[c]QTLs with Koshihikari alleles that decrease trait values are shown in red boxes, while those that increase trait values are shown in blue boxes.
The numbers to the right of each box indicate the number of populations in which the QTL was detected, relative to the total number of populations examined.

Supplemental Table 4. Mean grain length and grain width in 29 DPK-BC$_4$F$_2$ populations, along with graphical genotypes of their parental BC$_4$F$_1$ plants.

Line name			14-2403	14-2404	14-2405	14-2406	14-2407	14-2408	14-2409	14-2410	14-2411	14-2412	14-2413	14-2414	14-2415	14-2416	14-2417	14-2418	14-2419	14-2420	14-2421	14-2422	14-2423	14-2424	14-2425	14-2426	14-2427	14-2428	14-2429	14-2430	14-2431	Positions of QTL[c]	
Grain length	Mean±SD (mm)		6.83±0.29	6.84±0.32	6.91±0.30	6.97±0.32	6.94±0.33	7.01±0.32	7.16±0.33	7.05±0.33	6.96±0.31	6.90±0.33	7.30±0.36	7.14±0.35	7.14±0.33	7.01±0.33	6.99±0.32	7.03±0.35	7.00±0.32	7.14±0.32	7.07±0.34	7.05±0.34	7.19±0.31	7.04±0.33	7.04±0.31	6.91±0.32	7.03±0.32	6.94±0.34	7.18±0.32	7.31±0.32	7.11±0.33		
	QTL detection (Y/N)[a]		Y	Y	Y	Y	Y	N	Y	Y	Y	Y	Y	Y	Y	N	Y	Y	Y	Y	Y	Y	Y	N	N	Y	N	Y	N	Y	Y		
Grain width	Mean±SD (mm)		3.42±0.14	3.30±0.15	3.35±0.14	3.31±0.16	3.24±0.15	3.26±0.15	3.34±0.16	3.27±0.16	3.45±0.15	3.35±0.15	3.10±0.14	3.09±0.17	3.34±0.15	3.21±0.14	3.39±0.15	3.24±0.18	3.44±0.16	3.43±0.14	3.36±0.16	3.34±0.16	3.47±0.15	3.40±0.15	3.29±0.15	3.39±0.15	3.39±0.15	3.39±0.16	3.31±0.13	3.30±0.14	3.44±0.16		
	QTL detection (Y/N)[a]		Y	Y	N	Y	Y	Y	Y	Y	N	Y	Y	Y	Y	Y	N	Y	Y	Y	N	N	Y	N	Y	N	Y	Y	Y	Y	Y		
Genotypes[b]																																L	W
Marker	Chr. No.	Position (Mb)																															
RM3252	1	0.30	H	A	A	A	A	A	A	A	A	A	A	A	A	A	A	A	A	A	A	A	A	A	A	A	A	A	A	A	A		
RM6902	1	3.80	H	A	A	A	A	A	A	A	A	A	A	A	A	A	A	A	A	A	A	A	A	A	A	A	A	A	A	A	A	blue 1/1	red 1/1
RM5800	1	7.40	H	H	A	A	A	A	A	A	A	A	A	A	A	A	A	A	A	A	A	A	A	A	A	A	A	A	A	A	A		
RM3627	1	10.31	A	H	A	A	A	A	A	A	A	A	A	A	A	A	A	A	A	A	A	A	A	A	A	A	A	A	A	A	A		
RM5365	1	14.52	A	H	H	A	A	A	A	A	A	A	A	A	A	A	A	A	A	A	A	A	A	A	A	A	A	A	A	A	A		
RM6092	1	19.12	A	A	H	A	A	A	A	A	A	A	A	A	A	A	A	A	A	A	A	A	A	A	A	A	A	A	A	A	A	blue 1/1	
RM5718	1	22.93	A	A	H	A	A	A	A	A	A	A	A	A	A	A	A	A	A	A	A	A	A	A	A	A	A	A	A	A	A		
RM5461	1	26.91	A	A	H	H	A	A	A	A	A	A	A	A	A	A	A	A	A	A	A	A	A	A	A	A	A	A	A	A	A	red 2/2	
RM3614	1	29.83	A	A	A	H	A	A	A	A	A	A	A	A	A	A	A	A	A	A	A	A	A	A	A	A	A	A	A	A	A		
RM1003	1	33.48	A	A	A	H	A	A	A	A	A	A	A	A	A	A	A	A	A	A	A	A	A	A	A	A	A	A	A	A	A		
RM3825	1	36.47	A	A	A	H	A	A	A	A	A	A	A	A	A	A	A	A	A	A	A	A	A	A	A	A	A	A	A	A	A	blue 1/1	
RM3482	1	39.72	A	A	A	H	A	A	A	A	A	A	A	A	A	A	A	A	A	A	A	A	A	A	A	A	A	A	A	A	A		
RM6840	1	43.17	A	A	A	H	A	A	A	A	A	A	A	A	A	A	A	A	A	A	A	A	A	A	A	A	A	A	A	A	A		
RM2770	2	0.81	A	A	A	A	H	A	A	A	A	A	A	A	A	A	A	A	A	A	A	A	A	A	A	A	A	A	A	A	A		
RM3703	2	3.86	A	A	A	A	H	A	A	A	A	A	A	A	A	A	A	A	A	A	A	A	A	A	A	A	A	A	A	A	A		
RM5340	2	7.48	A	A	A	A	H	H	A	A	A	A	A	A	A	A	A	A	A	A	A	A	A	A	A	A	A	A	A	A	A		blue 1/1
RM5791	2	10.75	A	-	A	A	H	H	A	-	A	-	A	A	A	-	A	-	A	A	A	A	A	A	A	A	A	A	A	A	A		
RM3443	2	14.33	A	H	A	A	H	H	A	H	A	H	A	A	A	H	A	H	A	A	A	A	A	A	A	A	A	A	A	A	A		
RM1211	2	18.46	A	H	A	A	H	H	A	H	A	H	A	A	A	H	A	H	A	A	A	A	A	A	A	A	A	A	A	A	A		blue 6/7
RM5427	2	21.52	A	H	A	A	H	H	A	A	A	H	A	A	A	H	A	H	A	A	A	A	A	A	A	A	A	A	A	A	A		
RM3515	2	24.02	A	-	A	A	A	H	A	A	A	-	A	A	A	-	A	-	A	A	A	A	A	A	A	A	A	A	A	A	A		
RM1367	2	27.07	A	A	A	A	A	H	A	A	A	A	A	A	A	A	A	A	A	A	A	A	A	A	-	A	A	A	A	A	A		
RM6290	2	29.88	A	A	A	A	A	H	A	A	A	A	A	A	A	A	A	A	A	A	A	A	A	A	H	A	A	A	A	A	A		
RM6636	2	33.08	A	A	A	H	A	H	A	A	A	A	A	A	A	A	A	A	A	A	A	A	A	A	H	A	A	A	A	A	A	blue 1/3	
RM6137	2	35.77	A	A	A	H	A	H	A	A	A	A	A	A	A	A	A	A	A	A	A	A	A	A	H	A	A	A	A	A	A		
RM6139	2	35.85	A	A	A	H	A	H	A	A	A	A	A	A	A	A	A	A	A	A	A	A	A	A	H	A	A	A	A	A	A		blue 1/3
RM3413	3	0.37	A	A	A	A	A	A	H	A	A	A	A	A	A	A	-	A	A	A	A	A	A	A	A	A	A	A	A	A	A		
RM6849	3	3.29	A	A	A	A	A	A	H	A	A	A	A	A	A	A	-	A	A	A	A	A	A	A	A	A	A	A	A	-	A	red 1/1	
RM5755	3	5.96	A	A	A	A	A	A	H	A	A	A	A	A	A	A	A	A	A	A	H	A	A	A	A	A	A	A	A	H	A		
RM6783	3	9.32	A	A	A	A	A	A	H	A	A	A	A	A	A	A	A	A	A	A	A	A	A	A	A	A	A	A	A	H	A		
RM5551	3	12.93	A	A	A	A	A	A	H	A	A	A	A	A	A	A	A	A	A	A	A	A	A	A	A	A	A	A	A	H	A		
RM6881	3	16.88	A	A	A	A	A	A	H	A	A	A	A	A	A	A	A	A	A	A	A	A	A	A	A	A	A	A	A	H	A		
RM5684	3	18.41	A	A	A	A	A	A	H	A	A	A	A	A	A	A	A	A	A	A	A	A	A	A	A	A	A	A	A	H	A		
RM3646	3	22.00	A	A	A	A	A	A	H	A	A	A	A	A	A	A	A	A	A	A	A	A	A	A	A	A	A	A	A	H	A		blue 2/2
RM3513	3	25.11	A	A	A	A	A	A	H	H	A	A	A	A	A	A	A	H	A	A	A	A	A	A	A	A	A	A	A	H	A	red 2/4	
RM1350	3	28.68	A	A	A	A	A	A	A	H	A	A	A	A	A	A	A	A	A	A	A	A	A	A	A	A	A	A	A	H	A		
RM6970	3	32.44	A	A	A	A	A	A	A	H	A	A	A	A	A	A	A	A	A	A	A	A	A	A	A	A	A	A	A	A	A		blue 1/1
RM3329	3	35.60	A	A	A	A	A	A	A	H	A	A	A	A	A	A	A	A	A	A	A	A	A	A	A	A	A	A	A	A	A		
RM16252	4	0.06	A	A	A	A	A	A	A	A	H	A	A	A	A	A	A	A	A	A	A	A	A	A	A	A	A	A	A	A	A		
RM16354	4	2.06	A	A	A	A	A	A	A	A	H	A	A	A	A	A	A	A	A	A	A	A	A	A	A	A	A	A	A	A	A		
RM3658	4	5.61	A	A	A	A	A	A	A	A	H	A	A	A	A	A	A	A	A	A	A	A	A	A	A	A	A	A	A	A	A		
RM16554	4	8.68	A	A	A	A	A	A	A	A	H	A	A	A	A	A	A	A	A	A	A	A	A	A	A	A	A	A	A	A	A		
RM5633	4	13.08	A	A	A	A	A	A	A	A	H	H	A	A	A	A	A	A	A	A	A	A	A	A	A	A	A	A	A	A	A		
RM16753	4	16.92	A	A	A	A	A	A	A	A	H	A	A	A	A	A	A	A	A	A	A	A	A	A	A	A	A	A	A	A	A		
RM1354	4	26.30	A	A	A	A	A	A	A	A	A	H	A	A	A	A	A	A	A	A	A	A	A	A	A	A	A	A	A	A	A		
RM6089	4	29.59	A	A	A	A	A	A	A	A	A	H	A	A	A	A	A	A	A	A	A	A	A	A	A	A	A	A	A	A	A		
RM6909	4	32.06	A	A	A	A	A	A	A	A	A	H	H	A	A	A	A	A	A	A	A	A	A	A	A	A	A	A	A	A	A		
RM6006	4	34.89	A	A	A	A	A	-	A	A	A	H	H	A	A	A	A	A	A	A	A	A	A	A	A	A	A	A	H	A	A	red 1/3	red 1/3
RM17686	4	35.28	A	A	A	A	A	H	A	A	A	H	H	A	A	A	A	A	A	A	A	A	A	A	A	A	A	A	H	A	A		
RM1248	5	0.09	A	A	A	A	A	A	A	A	A	A	H	H	A	A	A	A	A	A	A	A	A	A	A	A	A	A	A	A	A		
RM6517	5	2.91	A	A	A	A	A	A	A	A	A	A	H	H	A	A	A	A	A	A	A	A	A	A	A	A	A	A	A	A	A	red 1/2	blue 2/2
RM3322	5	4.26	-	-	-	-	-	-	-	-	-	-	H	-	-	-	-	-	-	-	-	-	-	-	-	-	-	-	-	-	-		
RM2422	5	7.02	A	A	A	A	A	A	A	A	A	A	H	H	A	A	A	A	A	A	A	A	A	A	A	A	A	A	A	A	A		
RM6229	5	13.61	A	A	A	A	A	A	A	A	A	A	H	H	A	A	A	A	A	A	A	A	A	A	A	A	A	A	A	A	A		
RM3838	5	16.55	A	A	A	A	A	A	A	A	A	A	H	A	A	A	A	A	A	A	A	A	A	A	A	A	A	A	A	A	A		
RM5329	5	20.09	A	A	A	A	A	A	A	A	A	A	H	A	A	A	A	A	A	A	A	A	A	A	A	A	A	A	A	A	A		
RM5575	5	23.41	A	A	A	A	A	A	A	A	A	A	H	A	A	A	A	A	A	A	A	A	A	A	A	A	A	A	A	A	A		
RM3616	5	26.34	A	A	A	A	A	A	A	A	A	A	H	A	H	A	A	A	A	A	A	A	A	A	A	A	A	A	A	A	A		
RM1054	5	29.23	A	A	A	A	A	A	A	A	A	A	H	A	H	A	A	A	A	A	A	A	A	A	A	A	A	A	A	A	A	red 1/2	blue 1/2
RM6775	6	0.21	A	H	A	A	A	A	A	A	H	H	A	A	A	A	A	A	A	A	A	A	A	A	A	A	A	A	A	H	A	blue 2/4	
RM8125	6	3.17	A	H	A	A	A	A	A	A	-	-	-	A	-	-	-	A	A	A	A	A	A	A	A	A	A	A	A	H	A		
RM6857	6	6.57	A	H	A	A	A	A	A	A	A	A	H	A	H	H	H	A	A	A	A	A	A	A	A	A	A	A	A	H	A		
RM5963	6	8.82	A	H	A	A	H	A	A	A	A	A	H	A	H	H	H	A	A	A	A	A	A	A	H	A	A	A	A	-	A	red 3/7	
RM19875	6	11.74	A	A	A	A	A	A	A	A	A	A	A	A	A	H	H	A	A	A	A	A	A	A	H	A	A	A	A	A	A		
RM19985	6	13.89	A	A	A	A	A	A	A	A	A	A	A	A	A	H	A	H	A	A	A	A	A	A	A	A	A	A	A	A	A		
RM20084	6	17.07	A	A	A	A	A	A	A	A	A	A	A	A	A	H	A	H	A	A	A	A	A	A	A	A	A	A	A	A	A		
RM3498	6	20.98	A	A	A	A	A	A	A	A	A	A	A	A	A	H	A	H	A	A	A	A	A	A	A	A	A	A	A	A	A		
RM6202	6	24.26	A	A	A	A	A	A	A	A	A	A	A	A	A	H	A	H	A	A	A	A	A	A	A	A	A	A	A	A	A		
RM3138	6	28.47	A	A	A	A	A	A	A	A	A	A	A	A	A	H	A	H	A	A	A	A	A	A	A	A	A	A	A	H	A		red 1/3
RM3509	6	30.97	A	A	A	A	A	A	-	A	A	-	A	A	A	H	A	H	A	A	A	A	A	A	-	A	A	A	A	-	A	red 1/2	
RM3394	7	0.65	A	A	A	A	A	A	A	A	A	A	A	A	A	A	A	A	H	A	A	A	A	A	A	A	A	A	A	A	A	blue 1/1	
RM5711	7	3.14	A	A	A	A	A	A	A	A	A	A	A	A	A	A	A	A	H	A	-	A	A	A	A	A	A	A	A	A	A		red 1/1
RM6728	7	5.73	A	A	A	A	A	A	H	A	A	A	A	A	A	A	A	A	H	-	H	A	A	A	A	A	A	A	A	A	A		
RM3859	7	8.88	A	A	A	A	A	A	A	A	A	A	A	A	A	A	A	A	H	H	H	A	A	A	A	A	A	A	A	A	A		
RM3832	7	12.71	A	A	A	A	A	A	A	A	A	A	A	A	A	A	A	A	H	H	H	A	A	A	A	A	A	A	A	A	A		
RM5481	7	16.20	A	A	A	A	A	A	A	A	A	A	A	A	A	A	A	A	H	H	H	A	A	A	A	A	A	A	A	A	A	red 3/3	
RM5380	7	19.36	A	A	A	A	A	A	A	A	A	A	A	A	A	A	A	A	A	H	A	A	A	A	A	A	A	A	A	A	A		red 1/1
RM6885	7	23.01	-	A	A	A	A	A	A	A	A	A	A	A	A	A	A	A	A	H	A	A	A	A	A	A	A	A	A	A	A		
RM1330	7	25.66	H	A	A	A	A	A	A	A	A	A	A	A	A	A	A	A	A	H	A	A	A	A	A	A	A	A	A	A	A		
RM7601	7	29.04	A	A	A	A	A	A	A	A	A	A	A	A	A	A	A	A	A	H	A	A	A	A	A	A	A	A	A	A	A		
RM6369	8	0.13	A	A	A	A	A	A	A	A	A	A	A	A	A	A	A	A	A	A	H	A	A	A	A	A	A	A	A	A	A	red 1/1	
RM1376	8	3.17	A	A	A	A	A	A	A	A	A	A	A	A	A	A	A	A	A	A	H	A	A	A	A	A	A	A	A	A	A		
RM3507	8	6.16	A	A	A	A	A	A	A	A	A	A	A	A	A	A	A	A	A	A	H	A	A	A	A	A	A	A	A	A	A		
RM3395	8	10.29	A	A	A	A	H	A	A	A	A	A	A	A	A	A	A	A	A	A	H	H	A	A	A	A	A	A	A	A	A		
RM6990	8	14.36	A	A	A	A	H	A	A	A	A	A	A	A	A	A	A	A	A	A	A	H	A	A	A	A	A	A	A	A	A		
RM7285	8	18.76	A	A	A	A	H	A	A	A	A	A	A	A	A	A	A	A	A	A	A	H	A	A	A	A	A	A	A	A	A		
RM3634	8	21.58	A	A	A	A	H	A	A	A	A	A	A	A	A	A	A	A	A	A	A	H	H	A	A	A	A	A	A	A	A	red 1/3	red 1/3
RM8058	8	24.57	A	A	A	A	A	A	A	A	A	A	A	A	A	A	A	A	A	A	A	A	H	A	A	A	A	A	A	A	A		
RM3840	8	27.93	A	A	A	A	A	A	A	A	A	A	A	A	A	A	A	A	A	A	A	A	H	A	A	A	A	A	A	A	A		
RM8219	9	1.55	A	A	A	A	A	A	A	A	H	A	A	A	A	A	A	A	A	A	A	A	A	A	A	A	A	A	A	A	A		
RM5899	9	5.12	A	A	A	A	A	A	A	A	H	A	A	A	A	A	A	A	A	A	A	A	A	H	A	A	A	A	A	A	A		
RM5515	9	7.15	A	A	A	A	A	A	A	A	H	A	A	A	A	A	A	A	A	A	A	A	A	H	A	A	A	A	A	A	A		
RM7387	9	10.14	A	A	A	A	A	A	A	A	H	A	A	A	A	A	A	A	A	A	A	A	A	H	H	A	A	A	A	A	A	red 1/3	
RM6839	9	14.57	A	A	A	A	A	A	A	A	A	A	A	A	A	A	A	A	A	A	A	A	A	A	H	H	A	A	A	A	A		
RM3533	9	17.89	A	A	A	A	A	A	A	A	A	A	A	A	A	A	A	A	A	A	A	A	A	A	H	H	A	A	A	A	A	blue 1/2	
RM5786	9	19.79	A	A	A	A	A	A	A	A	A	A	A	A	A	A	A	A	A	A	A	A	A	A	-	H	A	A	A	A	A		
RM2482	9	22.58	A	A	A	A	A	A	A	A	A	A	A	A	A	A	A	A	A	A	A	A	A	A	A	H	A	A	A	A	A		
RM5095	10	0.05	A	A	A	A	A	A	A	A	A	A	A	A	A	A	A	A	A	A	A	A	A	A	A	A	H	A	A	A	A		
RM25031	10	3.66	A	A	A	A	A	A	A	A	A	A	A	A	A	A	A	A	A	A	A	A	A	A	A	A	H	A	A	A	A		blue 1/1
RM25158	10	7.94	A	A	A	A	A	A	A	A	A	A	A	A	A	A	A	A	A	A	A	A	A	A	A	A	H	A	A	A	A		
RM4455	10	11.74	A	A	A	A	A	A	A	A	A	A	A	A	A	A	A	A	A	A	A	A	A	A	A	A	H	A	A	A	A		
RM8201	10	13.83	A	A	A	A	A	A	A	A	A	A	A	A	A	A	A	A	A	A	A	A	A	A	A	A	H	A	A	A	A		
RM5274	10	17.92	A	A	A	A	A	A	A	A	A	A	A	A	A	A	A	A	A	A	A	A	A	A	A	A	H	H	A	A	H		red 2/3
RM7300	10	20.00	A	A	A	A	A	A	H	A	A	A	A	A	A	A	A	A	A	A	A	A	A	A	A	A	-	H	A	A	H	blue 2/3	
RM4771	10	23.09	A	A	A	A	A	A	A	A	A	A	A	A	A	A	A	A	A	A	A	A	A	A	A	A	A	H	A	A	H		
RM7203	11	1.08	A	A	A	A	A	A	A	A	A	A	A	A	A	A	A	A	A	A	A	A	A	A	A	A	A	A	H	A	A		blue 1/1
RM3339	11	4.14	H	A	A	A	A	A	A	A	A	A	A	A	A	A	A	A	A	A	A	A	A	A	A	A	A	A	H	A	A		
RM3137	11	6.19	H	A	A	A	A	A	A	A	A	A	A	A	A	A	A	A	A	A	A	A	A	A	A	A	A	A	H	-	A		
RM4469	11	6.26	-	A	A	A	A	A	A	A	A	A	A	A	A	A	A	A	A	A	A	A	A	A	A	A	A	A	-	H	A		
RM1206	11	9.77	H	A	A	A	A	A	A	A	A	A	A	A	A	A	A	A	A	A	A	A	A	A	A	A	A	A	H	H	A		
RM7226	11	14.51	H	A	A	A	A	A	A	A	A	A	A	A	A	A	A	A	A	A	A	A	A	A	A	A	A	A	H	H	A		
RM5312	11	17.63	A	A	A	A	A	A	A	A	A	A	A	A	A	A	A	A	A	A	A	A	A	A	A	A	A	A	H	H	A		
RM1219	11	21.52	A	A	A	A	A	A	A	A	A	A	A	A	A	A	A	A	A	A	A	A	A	A	A	A	A	A	A	H	A		
RM7277	11	24.68	A	A	A	A	A	A	A	A	A	A	A	A	A	A	A	A	A	A	A	A	A	A	A	A	A	A	A	H	A		
RM5926	11	28.96	A	A	A	A	A	A	A	-	A	A	A	A	A	A	A	A	H	A	A	A	A	A	A	A	H	H	A	-	H		
RM27414	12	0.40	A	A	A	A	A	A	A	A	A	A	A	A	A	A	A	A	A	A	A	A	A	A	A	A	A	A	A	H	A		
RM6296	12	3.20	A	A	A	A	A	A	A	A	A	A	A	A	A	A	A	A	A	A	A	A	A	A	A	A	A	A	A	A	H		
RM7003	12	6.78	A	A	A	A	A	A	A	A	A	A	A	A	A	A	A	A	A	A	A	H	A	A	A	A	A	A	A	A	H		
RM27900	12	9.57	A	A	A	A	A	A	A	A	A	A	A	A	A	A	A	A	A	A	A	H	A	A	A	A	A	A	A	A	H		
RM27971	12	12.24	A	A	A	A	A	A	A	A	A	A	A	A	A	A	A	A	A	A	A	H	A	A	A	A	A	A	A	A	H	red 2/2	
RM28102	12	15.91	A	A	A	A	A	A	A	A	A	A	A	A	A	A	A	A	A	A	A	H	A	A	A	A	A	A	A	A	H		
RM28305	12	19.96	A	A	A	A	A	A	A	A	A	A	A	A	A	A	A	A	A	A	A	H	A	A	A	A	A	A	A	A	A		
RM6693	12	23.80	A	A	A	A	A	A	A	A	A	A	A	A	A	A	A	A	A	A	A	H	A	A	A	A	A	A	-	A	A		
RM1227	12	27.34	A	A	A	A	A	A	A	A	A	A	A	A	A	A	A	A	A	A	A	H	A	A	A	A	A	A	H	A	A		blue 1/2

[a]Y indicates the presence, and N the absence, of a QTL.

[b]A indicates a homozygous Koshihikari allele; H indicates a heterozygous allele.

[c]QTLs with Koshihikari alleles that decrease trait values are shown in red boxes, while those that increase trait values are shown in blue boxes.
The numbers to the right of each box indicate the number of populations in which the QTL was detected, relative to the total number of populations examined.

Supplemental Table 5. Mean grain length and grain width in 32 NAK-BC$_4$F$_2$ populations, along with graphical genotypes of their parental BC$_4$F$_1$ plants.

Line name			14-2436	14-2437	14-2438	14-2439	14-2440	14-2441	14-2442	14-2443	14-2444	14-2445	14-2447	14-2448	14-2449	14-2450	14-2451	14-2452	14-2453	14-2454	14-2455	14-2456	14-2457	14-2458	14-2459	14-2460	14-2461	14-2462	14-2463	14-2464	14-2465	14-2466	14-2467	14-2468	Positions of QTL[c]	
Grain length	Mean±SD (mm)		7.05±0.31	7.15±0.32	7.02±0.32	7.26±0.33	7.09±0.34	6.94±0.32	7.15±0.35	7.09±0.31	7.07±0.33	7.05±0.34	6.90±0.34	7.15±0.35	7.14±0.33	7.10±0.33	6.81±0.31	7.01±0.32	6.94±0.32	7.06±0.33	7.16±0.33	7.00±0.32	7.00±0.32	7.07±0.33	7.02±0.34	6.99±0.33	7.01±0.35	7.02±0.34	6.93±0.36	6.99±0.35	6.99±0.34	6.87±0.30	7.13±0.36	7.08±0.34		
	QTL detection (Y/N)[a]		N	Y	Y	N	Y	Y	Y	Y	Y	Y	Y	Y	Y	Y	Y	N	Y	Y	Y	Y	N	N	N	N	N	N	N	N	N	Y	Y	Y		
Grain width	Mean±SD (mm)		3.46±0.17	3.38±0.15	3.44±0.14	3.08±0.13	3.24±0.16	3.28±0.17	3.42±0.16	3.27±0.14	3.37±0.15	3.41±0.17	3.36±0.16	3.35±0.17	3.09±0.16	3.34±0.17	3.24±0.15	3.39±0.16	3.39±0.17	3.44±0.15	3.34±0.14	3.43±0.15	3.43±0.15	3.42±0.15	3.37±0.15	3.41±0.16	3.32±0.16	3.38±0.17	3.40±0.16	3.33±0.16	3.40±0.15	3.29±0.15	3.39±0.17	3.41±0.14		
	QTL detection (Y/N)[a]		Y	Y	Y	Y	Y	Y	Y	Y	N	Y	Y	Y	Y	N	Y	N	N	Y	Y	Y	Y	Y	N	Y	N	N	N	Y	Y	Y	Y	Y		
Genotypes[b]																																				
Marker	Chr. No.	Position (Mb)																																	L	W
RM8068	1	1.66	H	A	A	A	A	A	A	A	A	A	A	A	A	A	A	A	A	A	A	A	A	A	A	A	A	A	A	A	A	A	A	A		
RM3521	1	4.40	H	A	A	A	A	A	A	A	A	A	A	A	A	A	A	A	A	A	A	A	A	A	A	A	A	A	A	A	A	A	-	A		1/1 (blue)
RM3234	1	7.57	H	A	A	A	A	A	A	A	A	A	A	A	A	A	A	A	A	A	A	A	A	A	A	A	A	A	A	A	A	A	H	A	1/2 (red)	
RM3627	1	10.31	H	H	A	A	A	A	A	A	A	A	A	A	A	A	A	A	A	A	A	A	A	A	A	A	A	A	A	A	A	A	A	A		
RM5365	1	14.52	A	H	A	A	A	A	A	A	A	A	A	A	A	A	A	A	A	A	A	A	A	A	A	A	A	A	A	A	A	A	A	A		
RM7075	1	15.12	A	H	A	A	A	A	A	A	A	A	A	A	A	A	A	A	A	A	A	A	A	A	A	A	A	A	A	A	A	A	A	A		
RM6092	1	19.12	A	H	A	A	A	A	A	A	A	A	A	A	A	A	A	A	A	A	A	A	A	A	A	A	A	A	A	A	A	-	A	A		
RM1349	1	25.07	A	H	A	A	A	A	A	A	A	A	A	A	A	A	A	A	A	A	A	A	A	A	A	A	A	A	A	A	A	H	A	A		1/2 (blue)
RM3336	1	28.62	A	H	A	A	A	A	A	A	A	A	A	A	A	A	A	A	A	A	A	A	A	A	A	A	A	A	A	A	A	H	A	A		
RM5914	1	31.51	A	H	H	A	A	A	A	A	A	A	A	A	A	A	A	A	A	A	A	A	A	A	A	A	A	A	A	A	A	A	0	A	2/2 (red)	
RM3304	1	34.96	A	H	H	A	A	A	A	A	A	A	A	A	A	A	A	A	A	A	A	A	A	A	A	A	A	A	A	A	A	A	A	A		
RM6504	1	38.99	A	A	H	A	A	A	A	A	A	A	A	A	A	A	A	A	A	A	A	A	A	A	A	A	A	A	A	A	A	A	A	A	1/1 (blue)	
RM6840	1	43.17	A	A	H	A	A	A	A	A	A	A	A	A	A	A	A	A	A	A	A	A	A	A	A	A	A	A	A	A	A	A	A	A		
RM6842	2	0.12	A	A	A	H	A	A	A	A	A	A	A	A	A	A	A	A	A	A	A	A	A	A	A	A	A	A	A	H	A	A	A	A		2/2 (blue)
RM3703	2	3.86	A	A	A	H	A	A	A	A	A	A	A	A	A	A	A	A	A	A	A	A	A	A	A	A	A	A	A	A	A	A	A	A		
RM4702	2	6.22	A	A	A	H	-	A	-	A	A	A	A	A	A	A	A	A	A	A	A	A	A	A	A	A	A	A	A	A	A	A	-	A		
RM6853	2	8.96	A	A	A	H	H	A	H	A	A	A	A	A	A	A	A	A	A	A	A	A	A	A	A	A	A	A	A	A	A	A	-	A		
RM5619	2	12.01	A	A	A	A	H	A	A	A	A	A	A	A	A	A	A	A	A	A	A	A	A	A	A	A	A	A	A	A	A	A	A	A		
RM5812	2	15.90	A	A	A	A	H	A	A	A	A	A	A	A	A	A	A	A	A	A	A	A	A	A	A	A	A	A	A	A	A	A	A	A		
RM5578	2	18.64	A	A	A	A	H	A	A	A	A	A	A	A	A	A	A	A	A	A	A	A	A	A	A	A	A	A	A	A	A	A	A	A		1/1 (blue)
RM5614	2	21.06	A	A	A	A	H	-	A	A	A	A	A	A	A	-	A	A	A	A	A	A	A	A	A	A	A	A	A	A	A	-	A	A		
RM3515	2	24.02	A	A	A	A	H	H	A	A	A	A	A	A	A	A	H	A	A	A	A	A	A	A	A	A	A	A	A	A	A	H	A	A	1/4 (red)	
RM3763	2	27.93	A	A	A	A	H	H	A	A	A	A	H	A	A	A	H	A	A	A	H	A	A	A	A	A	A	A	A	A	A	H	A	H		5/7 (blue)
RM5472	2	30.64	A	A	A	A	-	H	A	A	A	-	-	A	A	A	H	A	A	A	-	A	A	A	A	A	A	A	A	A	A	H	A	-	2/3 (blue)	
RM6627	2	32.75	-	-	-	-	-	-	-	-	-	-	-	-	-	-	-	-	-	-	H	-	-	-	-	-	-	-	-	-	-	-	-	-		
RM5607	2	33.27	A	A	A	A	A	H	A	A	A	-	A	A	A	A	H	A	A	A	A	A	A	A	A	A	A	A	A	A	A	H	A	A		2/3 (blue)
RM6139	2	35.85	A	A	A	A	A	H	A	A	A	A	A	A	A	A	H	A	A	A	A	A	A	A	A	A	A	A	A	A	A	H	A	A		
RM3387	3	0.38	A	A	A	A	A	A	H	A	A	A	A	A	A	A	A	A	A	A	A	A	A	A	A	A	A	A	A	A	A	A	A	A	1/1 (red)	
RM5474	3	3.80	A	A	A	A	A	A	H	A	A	A	A	A	A	A	A	A	A	A	A	A	A	A	A	A	A	A	A	A	A	A	A	A		
RM3134	3	7.24	A	A	A	A	A	A	H	A	A	A	A	A	A	A	A	A	A	A	A	A	A	A	A	A	A	A	A	A	A	A	A	A		
RM7197	3	9.89	A	A	A	A	A	A	-	H	A	A	A	A	A	A	A	A	A	A	A	A	A	A	A	A	A	A	A	A	A	A	A	A		1/1 (blue)
RM5489	3	13.31	A	A	A	A	A	A	A	H	A	A	A	A	A	A	A	A	A	A	A	A	A	A	A	A	A	A	A	A	A	A	A	A		
RM6914	3	17.29	A	A	A	A	A	A	A	H	A	A	A	A	A	A	A	A	A	A	A	A	A	A	A	A	A	A	A	A	A	A	A	A		
RM5488	3	21.39	A	A	A	A	A	A	A	H	A	A	A	A	A	A	A	A	A	A	A	A	A	A	A	A	A	A	A	A	A	A	A	A		
RM6266	3	23.82	A	A	A	A	A	A	A	H	A	A	A	A	A	A	A	A	A	A	A	A	A	A	A	A	A	A	A	A	A	A	A	A		
RM2334	3	26.75	A	A	A	A	A	A	A	H	A	A	A	A	A	H	A	A	A	A	A	-	A	A	A	A	A	A	A	A	A	A	A	A		
RM3525	3	30.39	A	A	A	H	A	A	A	A	H	A	A	A	A	A	A	A	A	A	A	A	A	A	A	A	A	A	A	A	A	A	A	A		
RM6970	3	32.44	A	A	A	H	A	A	A	A	H	A	A	A	A	A	A	A	A	A	A	A	A	A	A	A	A	A	A	A	A	A	A	A	1/2 (blue)	
RM1221	3	35.68	A	A	A	H	A	A	A	A	H	A	A	A	A	A	A	A	A	A	A	A	A	A	A	A	A	A	A	A	A	A	A	A		
RM5414	4	2.03	A	A	A	A	A	A	A	A	A	H	H	A	A	A	A	A	A	A	A	A	A	A	A	A	A	A	H	A	A	A	A	A		
RM7200	4	4.04	A	A	A	A	A	A	A	A	A	H	H	A	A	A	A	A	A	A	A	A	A	A	A	A	A	A	A	A	A	A	A	A	1/2 (blue)	2/2 (red)
RM16540	4	8.22	A	A	A	A	A	A	A	A	A	H	H	A	A	A	A	A	A	A	A	A	A	A	A	A	A	A	A	A	A	A	A	A		
RM5633	4	13.08	A	A	A	A	A	A	A	A	A	H	H	A	A	A	A	A	A	A	A	A	A	A	A	A	A	A	A	A	A	A	A	A		2/2 (red)
RM16753	4	16.92	A	A	A	A	A	A	A	A	A	H	H	A	A	A	A	A	A	A	A	A	A	A	A	A	A	A	A	A	A	A	A	A		
RM16855	4	19.22	A	A	A	A	A	A	A	A	A	A	H	H	A	A	A	A	A	A	A	A	A	A	A	A	A	A	A	A	A	A	A	A		1/2 (blue)
RM3524	4	22.89	A	A	A	A	A	A	A	A	A	A	A	H	A	A	A	A	A	A	A	H	A	A	A	A	A	A	A	A	A	A	A	A	2/2 (red)	
RM1354	4	26.30	A	A	A	A	A	A	A	A	A	A	A	H	A	A	A	A	A	A	A	-	A	A	A	A	A	A	A	A	A	A	A	A		
RM6365	4	29.83	A	A	A	A	A	A	A	A	A	A	A	H	A	A	A	A	A	A	A	A	A	A	A	A	A	A	A	A	A	A	A	A		1/1 (red)
RM6909	4	32.06	A	A	A	A	A	A	A	A	A	A	A	H	A	A	A	A	A	A	A	A	A	A	A	A	A	A	A	A	A	A	A	A		
RM5608	4	35.36	A	A	A	A	A	A	A	A	A	A	A	-	A	A	A	H	H	A	A	A	A	A	A	A	A	A	A	A	A	A	A	A		
RM1248	5	0.09	A	A	A	A	A	A	A	A	A	A	A	A	H	A	A	A	A	A	A	A	A	A	A	A	A	A	A	A	A	A	A	A		
RM3777	5	4.17	A	A	A	H	A	A	A	A	A	A	A	A	H	A	A	A	A	A	A	A	A	A	A	A	A	A	A	A	A	A	A	A	1/2 (red)	2/2 (blue)
RM2998	5	7.24	A	A	A	A	A	A	A	H	A	A	A	A	H	A	A	A	A	A	A	A	A	A	A	A	A	A	A	A	A	A	A	A		
RM8039	5	13.43	A	A	A	A	A	A	A	H	A	A	A	A	H	-	A	A	A	A	A	A	A	A	A	A	A	A	A	A	A	A	A	A	1/2 (red)	
RM6742	5	14.88	A	A	A	A	A	A	A	-	A	A	A	A	H	H	A	A	A	A	A	A	A	A	A	A	A	A	A	A	A	A	A	A		
RM1237	5	18.02	A	A	A	A	A	A	A	A	A	A	A	A	A	H	A	A	A	A	A	A	A	A	A	A	A	A	A	A	A	A	A	A		
RM3351	5	20.76	A	A	A	A	A	A	A	A	A	A	A	A	A	H	A	A	A	A	A	A	A	A	A	A	A	A	A	A	A	A	A	A		
RM6598	5	24.13	A	A	A	A	A	A	A	A	A	A	A	A	A	H	A	A	A	A	A	A	A	A	A	A	A	A	A	A	A	A	A	A		
RM5784	5	27.86	A	A	A	A	A	A	A	A	A	A	A	A	A	H	H	A	A	A	A	A	A	A	A	A	A	A	A	A	A	H	A	A		
RM5907	5	29.61	A	A	A	A	A	A	A	A	A	A	A	A	-	-	H	A	A	A	A	A	A	A	A	A	A	A	A	A	A	H	A	A	1/2 (blue)	
RM6775	6	0.21	A	A	H	H	A	H	A	A	A	A	H	A	A	A	H	A	A	A	A	A	A	A	A	A	A	A	H	A	A	H	A	A	1/7 (blue)	
RM3805	6	2.85	A	A	H	H	A	H	A	A	A	A	H	A	A	A	H	H	A	A	A	A	A	A	A	A	A	A	H	A	A	H	A	A		1/8 (red)
RM2615	6	5.96	A	A	H	H	H	H	A	A	A	H	H	A	A	A	H	H	A	A	H	A	A	A	A	A	A	A	H	A	A	A	A	A		
RM6836	6	9.31	A	A	A	-	A	A	A	A	A	A	A	A	A	A	A	H	A	A	A	A	A	A	A	A	A	A	A	A	A	A	H	A	1/2 (blue)	
RM3183	6	12.45	A	A	A	A	A	A	H	A	A	A	A	A	A	A	A	H	H	A	A	A	A	A	A	A	A	A	A	A	A	A	H	A		1/4 (red)
RM20046	6	16.09	A	A	A	A	A	A	-	A	A	A	A	A	A	A	A	-	H	A	A	A	A	A	A	A	A	A	A	A	A	A	H	A		1/2 (blue)
RM20162	6	19.75	A	A	A	A	A	A	H	A	A	A	A	A	A	A	A	A	H	A	A	A	A	A	A	A	A	A	A	A	A	A	H	A		
RM1340	6	23.34	A	A	A	A	A	A	H	A	A	A	A	A	A	A	A	A	H	A	A	A	A	A	A	A	A	A	A	A	A	A	A	A	1/2 (red)	
RM3430	6	27.43	A	A	A	A	A	A	H	A	A	A	A	A	A	A	A	A	H	A	A	A	A	A	A	A	A	A	A	A	-	A	A	A		
RM3509	6	30.97	A	A	A	A	A	A	A	A	A	A	A	A	A	A	A	A	A	A	A	A	A	A	A	A	A	A	A	A	H	A	A	A		1/1 (red)
RM3831	7	1.17	A	A	A	A	A	A	A	A	A	A	A	A	A	A	A	A	A	H	A	A	A	A	A	A	A	A	A	A	A	A	A	H		
RM7479	7	4.14	A	A	A	A	A	A	A	A	A	A	A	A	A	A	A	A	A	H	A	A	A	A	A	A	A	A	A	A	A	A	A	H	1/2 (red)	2/2 (red)
RM8006	7	7.69	A	A	A	A	A	A	A	A	A	A	A	A	A	A	A	A	A	H	H	A	A	A	A	A	A	A	A	A	A	A	A	-		
RM3635	7	11.09	A	A	A	A	A	A	A	A	A	A	A	A	A	A	A	A	A	H	H	A	A	A	A	A	A	A	A	A	A	A	A	A		
RM3755	7	14.73	A	A	A	A	A	A	A	A	A	A	A	A	A	A	A	A	A	H	H	A	A	A	A	A	A	A	A	A	A	A	A	A		
RM4952	7	17.80	A	A	A	A	A	A	A	A	A	A	A	A	A	A	A	A	A	-	H	A	A	A	A	A	A	A	A	A	A	A	A	A		
RM1279	7	21.61	A	A	A	A	A	A	A	A	A	A	A	A	A	A	A	A	A	A	H	A	A	A	A	A	A	A	A	A	A	A	A	A	1/1 (red)	
RM1330	7	25.66	A	A	A	A	A	A	A	A	A	A	A	A	A	A	A	A	A	A	H	A	A	A	A	A	A	A	A	A	A	A	A	A		
RM7601	7	29.04	A	A	A	A	A	A	A	A	A	A	A	A	A	A	A	A	A	A	H	A	A	A	A	A	A	A	A	A	A	A	A	A		
RM5911	8	0.08	A	A	A	A	A	A	A	A	A	A	A	A	A	A	A	A	A	A	A	H	A	A	A	A	A	A	A	A	A	A	A	A		
RM4955	8	2.96	A	A	A	A	A	A	A	A	H	A	A	A	A	A	A	A	A	A	A	H	A	A	A	A	A	A	A	A	A	A	A	A		1/2 (blue)
RM6208	8	5.79	A	A	A	A	A	A	A	A	H	A	A	A	A	A	A	H	A	A	A	H	H	A	A	A	A	A	A	A	A	A	A	A	1/4 (red)	
RM8243	8	8.94	A	A	A	A	A	A	A	A	A	A	A	A	A	A	A	A	A	A	A	H	H	A	A	A	A	A	A	A	A	A	A	A		
RM3383	8	13.49	A	A	A	A	A	A	A	A	A	A	A	A	A	A	A	A	A	A	A	H	H	A	A	A	A	A	A	A	A	A	A	A		
RM3153	8	18.99	A	A	A	A	A	A	A	A	A	A	A	A	A	A	A	A	A	A	A	A	H	A	A	A	A	A	A	A	A	A	A	A		
RM6635	8	22.17	A	A	A	A	A	A	A	A	A	-	-	A	A	A	A	A	A	A	A	A	H	A	A	A	A	A	A	A	A	A	-	A		1/1 (red)
RM5493	8	26.15	A	A	A	A	A	A	A	A	A	-	A	A	A	A	A	A	A	A	A	A	H	A	A	A	A	A	A	A	A	A	A	A		
RM3840	8	27.93	A	A	A	H	A	A	A	A	A	A	A	A	A	A	A	A	A	A	A	A	A	A	A	A	A	A	A	A	A	A	A	A		
RM8219	9	1.55	A	A	A	A	A	A	A	A	A	A	A	A	A	A	A	A	A	A	A	A	A	A	H	A	A	A	A	A	A	A	A	A		
RM5899	9	5.12	A	A	A	A	A	A	A	A	A	A	A	A	A	A	A	A	A	A	A	A	A	H	H	A	A	A	A	A	A	A	A	A		
RM6920	9	7.01	A	A	A	A	A	A	A	A	A	A	A	A	A	A	A	A	A	A	A	A	A	H	H	A	A	A	A	A	A	A	A	A		
RM3907	9	10.49	A	A	A	A	A	A	A	H	A	A	A	A	A	A	A	A	A	A	A	A	A	H	H	A	A	A	A	A	A	A	A	A		
RM5657	9	14.37	A	A	A	A	A	A	A	H	A	A	A	A	A	A	A	A	A	A	A	A	A	H	A	A	A	A	A	A	A	A	A	A		
RM7048	9	16.94	A	A	A	A	A	A	A	H	A	A	A	A	A	A	A	A	A	A	A	A	A	H	A	H	A	A	A	A	A	A	A	A		
RM3808	9	20.55	A	A	A	A	A	A	A	A	A	A	A	A	A	A	A	A	A	A	A	A	A	H	A	H	A	A	A	A	A	A	A	A		1/2 (red)
RM6797	9	22.71	A	A	A	A	A	A	A	A	A	A	A	A	A	A	A	A	A	A	A	A	A	H	H	H	A	A	H	A	A	A	A	A		
RM5095	10	0.05	H	H	A	A	A	H	A	A	A	A	A	A	A	A	A	A	A	A	A	A	A	A	A	A	H	H	A	A	A	A	A	A		
RM3882	10	2.74	A	A	A	A	A	H	A	A	A	A	A	A	A	A	A	A	A	A	A	A	A	A	A	A	H	H	A	A	A	A	A	A		
RM25151	10	7.62	A	A	A	A	A	H	A	A	A	A	A	A	A	A	A	A	A	A	A	A	A	A	A	A	H	H	A	A	A	A	A	A		
RM6833	10	11.41	A	A	A	A	A	-	A	A	A	A	A	A	A	A	A	A	A	A	A	A	A	A	A	A	H	-	A	A	A	A	A	A		
RM8201	10	13.83	A	A	A	A	A	A	A	A	A	A	A	A	A	A	A	A	A	A	A	A	A	A	A	A	H	A	H	A	A	A	A	A		
RM5620	10	17.47	A	A	A	A	A	A	A	A	A	A	A	A	A	A	A	A	A	A	A	A	A	A	A	A	H	A	H	A	A	A	A	A		
RM4477	10	21.17	A	A	A	A	A	A	A	A	A	A	A	A	A	A	A	A	-	A	A	A	A	A	A	A	H	A	H	A	A	A	A	A		
RM6824	10	23.11	A	A	A	A	A	A	A	A	A	A	A	A	A	A	A	A	H	A	A	A	A	A	A	A	H	A	H	A	A	A	A	A	1/3 (blue)	
RM7203	11	1.08	A	A	A	A	A	A	A	A	A	A	A	A	A	A	A	A	A	A	A	A	A	A	A	A	A	A	A	H	A	A	A	A		
RM3339	11	4.14	A	A	A	A	A	A	A	A	A	A	A	A	A	A	A	A	A	A	A	A	A	A	A	A	A	A	A	H	H	A	A	A		
RM3133	11	6.19	A	A	A	A	A	A	A	A	A	A	A	A	A	A	A	A	A	A	A	A	A	A	A	A	A	A	A	H	H	A	A	A		
RM7283	11	9.12	A	A	A	A	A	A	A	A	A	A	A	A	A	A	A	A	A	A	A	A	A	A	A	A	A	A	A	H	H	A	A	A		
RM5590	11	11.69	A	A	A	A	A	A	A	A	A	A	A	A	A	A	A	A	A	A	A	A	A	A	A	A	A	A	A	H	H	A	A	A		
RM5824	11	14.53	A	A	A	A	A	A	A	A	A	A	A	A	A	A	A	A	A	A	A	A	A	A	A	A	A	A	A	H	H	A	A	A		
RM6680	11	19.08	A	A	A	A	A	A	A	A	A	A	A	A	A	A	A	A	A	A	A	A	A	A	A	A	A	A	A	H	H	A	A	A		
RM2191	11	25.13	A	A	A	A	A	A	A	A	A	A	A	A	A	A	A	A	A	A	A	A	A	A	A	A	A	A	A	A	H	A	A	A		
RM5926	11	28.96	A	A	A	A	A	A	A	A	A	A	A	A	A	A	A	A	A	A	A	A	A	A	A	A	A	A	A	A	H	A	A	A		
RM27400	12	0.11	A	A	A	-	H	A	A	A	A	A	A	A	A	A	A	A	A	A	A	A	A	A	A	A	A	A	A	A	A	H	A	A		
RM27627	12	3.84	H	A	A	H	H	A	A	A	A	A	A	A	A	A	A	A	A	A	A	A	A	A	A	A	A	A	A	A	A	H	A	A	1/4 (red)	
RM6905	12	6.75	A	A	A	H	A	A	A	A	A	A	A	A	A	A	A	A	A	A	A	A	A	A	A	A	A	A	A	A	A	A	H	H		
RM7195	12	9.90	A	A	A	A	A	A	A	A	A	A	A	A	A	A	A	A	A	A	H	A	A	A	A	A	A	A	A	A	A	A	H	H		
RM27971	12	12.24	A	A	A	H	A	A	A	A	A	A	A	A	A	A	A	A	A	A	H	A	A	A	A	A	A	A	A	A	A	A	H	H		
RM28102	12	15.91	A	A	A	A	A	A	A	A	A	A	A	A	A	A	A	A	A	A	H	A	A	A	A	A	A	A	A	A	A	A	H	H		
RM1246	12	19.12	A	A	A	A	A	A	A	A	A	A	A	A	A	A	A	A	A	A	A	A	A	A	A	A	A	A	A	A	A	A	H	A		1/1 (blue)
RM7376	12	23.48	A	A	A	A	A	A	A	A	A	A	A	A	A	A	A	A	A	A	A	A	A	A	A	A	A	A	A	A	A	A	H	A		
RM1227	12	27.34	A	A	A	A	A	A	A	A	A	A	A	A	A	A	H	A	A	A	A	A	A	A	A	A	A	A	A	A	A	H	A	A		1/2 (blue)

[a]Y indicates the presence, and N the absence, of a QTL.

[b]A indicates a homozygous Koshihikari allele; H indicates a heterozygous allele.

[c]QTLs with Koshihikari alleles that decrease trait values are shown in red boxes, while those that increase trait values are shown in blue boxes.
The numbers to the right of each box indicate the number of populations in which the QTL was detected, relative to the total number of populations examined.

Supplemental Table 6. Mean grain length and grain width in 31 BLK-BC$_4$F$_2$ populations, along with graphical genotypes of their parental BC$_4$F$_1$ plants.

Line name			14-2473	14-2474	14-2475	14-2476	14-2477	14-2478	14-2479	14-2480	14-2481	14-2482	14-2483	14-2484	14-2485	14-2486	14-2487	14-2488	14-2489	14-2490	14-2492	14-2493	14-2494	14-2495	14-2496	14-2497	14-2498	14-2499	14-2500	14-2501	14-2502	14-2503	14-2504		
Grain length	Mean±SD (mm)		6.93±0.32	7.26±0.33	6.90±0.34	6.84±0.33	6.98±0.33	6.99±0.31	7.50±0.34	7.28±0.35	6.91±0.33	6.84±0.32	7.16±0.35	7.07±0.35	6.94±0.34	6.97±0.34	7.07±0.32	7.01±0.33	7.00±0.35	7.16±0.35	7.09±0.34	7.14±0.33	7.06±0.31	6.94±0.32	7.29±0.34	6.96±0.34	7.08±0.34	7.02±0.34	6.85±0.31	7.11±0.32	7.08±0.33	7.00±0.36	6.97±0.34		
	QTL detection (Y/N)[a]		N	Y	Y	N	N	N	Y	Y	N	N	Y	Y	Y	Y	Y	Y	Y	Y	Y	Y	Y	N	N	N	Y	N	Y	Y	N	Y	N		
Grain width	Mean±SD (mm)		3.24±0.17	3.42±0.16	3.32±0.18	3.29±0.18	3.36±0.18	3.33±0.18	3.29±0.17	3.41±0.16	3.34±0.17	3.35±0.17	3.33±0.18	3.21±0.18	3.32±0.20	3.31±0.19	3.33±0.15	3.38±0.16	3.36±0.20	3.43±0.17	3.41±0.16	3.38±0.14	3.42±0.16	3.48±0.17	3.40±0.15	3.31±0.17	3.39±0.18	3.39±0.17	3.41±0.15	3.40±0.15	3.42±0.16	3.37±0.16	3.27±0.17		
	QTL detection (Y/N)[a]		Y	Y	N	N	Y	Y	Y	Y	Y	Y	Y	Y	Y	N	Y	Y	Y	Y	Y	Y	Y	Y	N	Y	N	N	Y	Y	Y	N	Y	Positions of QTL[c]	
Genotypes[b]																																		L	W
Marker	Chr. No.	Position (Mb)																																	
RM7383	1	3.48	H	A	A	A	A	A	A	A	A	A	A	A	A	A	A	A	A	A	A	A	A	A	A	A	A	A	A	A	A	A	A		
RM6599	1	7.08	H	A	A	A	A	A	A	A	A	A	A	A	A	A	A	A	A	A	A	A	A	A	A	A	A	A	A	A	A	A	A		
RM3627	1	10.31	H	H	A	A	A	A	A	A	A	A	A	A	A	A	A	A	A	A	A	A	A	A	A	A	A	A	A	A	A	A	A		
RM3412	1	11.58	-	H	A	A	A	A	A	A	A	A	A	A	A	A	A	A	A	A	A	A	A	A	A	A	A	A	A	A	A	A	A		
RM7075	1	15.12	A	H	A	A	A	A	A	A	A	A	A	A	A	A	A	A	A	A	A	A	A	A	A	A	A	A	A	A	A	A	A	1/1 (blue)	
RM6092	1	19.12	A	H	A	A	A	A	A	A	A	A	A	A	A	A	A	A	A	A	A	A	A	A	A	A	A	A	A	A	A	A	A		1/1 (red)
RM5718	1	22.93	A	H	A	A	A	A	A	A	A	A	A	A	A	A	A	A	A	A	A	A	A	A	A	A	A	A	A	A	A	A	A		
RM5461	1	26.91	A	H	A	A	A	A	A	A	A	A	A	A	A	A	A	A	A	A	A	A	A	A	A	A	A	A	A	A	A	A	A	1/1 (red)	
RM7371	1	29.81	A	H	A	A	A	-	A	A	A	A	A	A	A	A	A	A	A	A	A	A	A	A	A	A	A	A	A	A	A	A	A		
RM1003	1	33.48	A	H	H	A	H	H	A	A	A	A	A	A	A	A	A	A	A	A	A	A	A	A	A	A	A	A	A	A	A	A	A		2/4 (red)
RM5448	1	37.40	A	A	H	A	-	-	A	A	A	A	A	A	A	A	A	A	A	A	A	A	A	A	A	A	A	A	-	A	A	A	A	1/1 (blue)	
RM1387	1	40.21	A	A	H	A	A	H	A	A	A	A	A	A	A	A	A	A	A	A	A	A	A	A	A	A	A	A	H	A	A	A	A		1/3 (blue)
RM6840	1	43.17	A	A	H	A	A	A	A	A	A	A	A	A	A	A	A	A	A	A	A	A	A	A	A	A	A	A	H	A	A	A	A		
RM7451	2	0.65	A	A	-	H	A	A	A	A	A	A	A	A	A	A	A	A	A	A	A	A	A	A	A	A	A	A	A	A	A	A	A		
RM3188	2	3.45	A	A	A	H	A	A	A	A	A	A	A	A	A	A	A	A	A	A	A	A	A	A	A	A	A	A	A	A	A	A	A		
RM5340	2	7.48	A	A	A	H	-	A	A	H	A	A	A	A	A	A	A	A	-	A	A	A	A	A	-	-	A	A	A	A	A	A	A		
RM1313	2	11.26	A	A	A	A	H	A	A	H	A	A	A	A	A	A	A	A	A	A	A	A	A	A	A	H	A	A	A	A	A	A	A		
RM3443	2	14.33	A	A	A	A	H	A	A	H	A	A	A	A	A	A	A	A	A	A	A	A	A	A	A	H	A	A	A	A	A	A	A		3/3 (blue)
RM1211	2	18.46	A	A	A	A	H	-	A	A	A	A	A	A	A	A	A	A	A	A	A	A	A	A	A	H	A	A	A	A	A	A	A		
RM5614	2	21.06	A	A	A	A	H	H	A	A	A	A	A	A	A	A	A	A	A	A	A	A	A	A	A	-	A	A	A	A	A	A	A		
RM3515	2	24.02	A	A	A	A	H	H	A	A	A	A	A	A	A	A	A	A	A	A	A	A	A	A	A	A	A	A	A	A	A	A	A		
RM5631	2	28.27	A	A	A	A	A	H	A	A	A	A	A	A	A	A	A	A	A	A	A	A	A	A	A	A	A	A	A	A	A	A	A		
RM6636	2	33.08	A	A	A	A	A	H	A	A	A	A	A	A	A	A	A	A	A	A	A	A	A	A	A	A	A	A	A	A	A	A	H		(blue)
RM6137	2	35.77	A	A	A	A	A	A	A	A	A	A	A	A	A	A	A	A	A	A	A	A	A	A	A	A	A	H	A	A	A	A	A		
RM3387	3	0.38	A	A	A	A	A	A	H	A	A	A	A	A	A	A	A	A	A	A	A	A	A	A	A	A	A	A	A	H	A	A	A	2/2 (red)	1/2 (red)
RM5474	3	3.80	A	A	A	A	A	A	H	A	A	A	A	A	A	A	A	A	A	A	A	A	A	A	A	A	A	A	A	-	A	A	A		
RM3766	3	6.93	A	A	A	A	A	A	H	A	A	A	A	A	A	A	A	A	A	A	A	A	A	A	A	A	A	A	A	A	A	A	A		1/1 (blue)
RM3434	3	10.78	A	A	A	A	A	A	H	A	A	A	A	A	A	A	A	A	A	A	A	A	A	A	A	A	A	A	A	A	A	A	A		
RM3291	3	13.60	A	A	A	A	A	A	H	A	A	A	A	A	A	A	A	A	A	A	A	A	A	A	A	A	A	A	A	A	A	A	A		
RM6914	3	17.29	A	A	A	A	A	A	H	H	A	A	A	A	A	A	A	A	A	A	A	A	A	A	A	A	A	A	A	A	A	A	A	2/2 (red)	1/2 (blue)
RM5488	3	21.39	A	A	A	A	A	A	-	H	A	A	A	A	A	A	A	A	A	A	A	A	A	A	A	A	A	A	A	A	A	A	A		
RM3513	3	25.11	A	A	A	A	A	A	A	H	A	A	A	A	A	A	A	A	A	A	A	A	A	A	A	A	A	A	A	A	A	A	A		
RM2614	3	27.43	A	A	A	A	A	A	A	H	A	A	A	A	A	A	A	A	A	A	A	A	A	A	A	A	A	A	A	A	A	A	A		1/1 (red)
RM5813	3	30.98	A	A	A	A	A	A	A	H	A	A	A	A	A	A	A	H	A	A	A	A	A	A	A	A	A	A	A	A	A	A	A		
RM7076	3	33.66	A	A	A	A	A	A	A	H	A	A	A	A	A	A	A	H	A	A	A	A	A	A	A	A	A	A	A	A	A	A	A		
RM7389	3	36.16	A	A	A	A	A	A	A	H	A	A	A	A	A	A	A	H	A	A	A	A	A	A	A	A	A	A	A	A	A	A	A		
RM16252	4	0.06	A	A	A	A	A	A	A	A	H	A	A	A	A	A	A	A	A	A	A	A	-	A	A	A	A	A	A	A	A	A	A		
RM7200	4	4.04	A	A	A	A	A	A	A	A	H	H	A	A	A	A	A	A	A	A	A	A	A	A	A	A	A	A	A	A	A	A	A		1/2 (red)
RM16540	4	8.22	A	A	A	A	A	A	A	A	H	H	A	A	A	A	A	A	A	A	A	A	A	A	A	A	A	A	A	A	A	A	A		
RM16670	4	13.88	A	A	A	A	A	A	A	A	H	H	A	A	A	A	A	A	A	A	A	A	A	A	A	A	A	A	A	A	A	A	A		
RM16761	4	17.10	A	A	A	A	A	A	A	A	H	H	A	A	A	A	A	A	A	A	A	A	A	A	A	A	A	A	A	A	A	A	A		2/2 (blue)
RM1359	4	20.03	A	A	A	A	A	A	A	A	-	H	A	A	A	A	A	A	A	A	A	A	A	A	A	A	A	A	A	A	A	A	A		
RM3785	4	24.25	A	A	A	A	A	A	A	A	A	H	H	A	A	A	A	A	A	A	A	A	A	A	A	A	A	A	A	A	A	A	A		
RM3781	4	28.76	A	H	A	A	A	A	A	A	A	A	H	A	A	A	A	A	A	A	A	A	A	A	A	A	A	A	A	A	A	A	A		
RM6909	4	32.06	A	H	A	A	A	A	A	A	A	A	H	A	A	A	A	A	A	A	A	A	A	A	A	A	A	A	A	A	A	A	A	1/2 (red)	1/2 (red)
RM7030	4	35.38	A	H	A	A	A	A	A	A	A	A	H	A	A	A	A	A	A	A	A	A	A	A	A	A	A	A	A	A	A	A	A		
RM1248	5	0.09	H	A	A	A	A	A	A	A	A	A	A	H	A	A	A	A	A	A	A	A	A	A	A	A	A	A	A	A	A	A	A		
RM3322	5	4.26	H	A	A	A	A	A	A	A	A	A	A	H	A	A	A	A	A	A	A	A	A	A	A	A	A	A	A	A	A	A	A		2/2 (blue)
RM3683	5	7.18	A	A	A	A	A	A	A	A	A	A	A	H	A	A	A	A	A	A	A	A	A	A	A	A	A	A	A	A	A	A	A		
RM5844	5	9.15	A	A	A	A	A	A	A	A	A	A	-	H	A	A	A	A	A	A	A	A	A	A	A	A	A	A	A	A	A	A	A		
RM2676	5	13.54	A	A	A	A	A	A	A	A	A	A	-	H	A	A	A	A	A	A	A	A	A	A	A	A	A	A	A	A	A	A	A	1/1 (red)	
RM6024	5	17.81	A	A	A	A	A	A	A	A	A	A	H	H	A	A	A	A	A	A	A	A	A	A	A	A	A	A	A	A	A	A	A		
RM3663	5	21.43	A	-	A	A	A	A	A	A	A	A	H	A	-	A	A	A	A	A	A	A	A	A	A	A	A	A	A	A	A	A	A		
RM3348	5	25.15	A	H	A	A	A	A	A	A	A	A	H	A	H	A	A	A	A	A	A	A	A	A	A	A	A	A	A	A	A	A	A		2/3 (blue)
RM6313	5	28.34	A	H	A	A	A	A	A	A	A	A	A	A	H	A	A	A	A	A	A	A	A	A	A	A	A	A	A	A	A	A	A	1/2 (red)	
RM6775	6	0.21	A	A	A	A	A	A	A	A	A	A	A	A	A	H	A	A	A	A	A	A	A	A	A	A	A	A	A	A	A	A	A		
RM3805	6	2.85	A	A	A	A	A	A	A	A	A	A	A	A	A	H	A	A	A	A	A	A	A	A	A	A	A	A	A	A	A	A	A		
RM2615	6	5.96	A	A	A	A	A	A	A	A	A	A	A	A	A	H	H	A	A	A	A	A	A	A	A	A	A	A	A	A	A	A	A		
RM19807	6	9.86	A	A	A	A	A	A	A	A	A	A	A	A	A	A	H	A	A	A	A	A	A	A	A	A	A	A	A	A	A	A	A	1/1 (red)	
RM19980	6	13.69	A	A	A	A	A	A	A	A	A	A	A	A	A	A	H	A	A	A	A	A	A	A	A	A	A	A	A	A	A	A	A		
RM20084	6	17.07	A	A	A	A	A	A	A	A	A	A	A	A	A	A	H	A	A	A	A	A	A	A	A	A	A	A	A	A	A	A	A		1/1 (blue)
RM7193	6	20.26	A	A	A	A	A	A	A	A	A	A	A	A	A	A	H	H	A	A	A	A	A	A	A	A	A	A	A	A	A	A	A		
RM7269	6	23.68	A	A	A	A	A	A	A	A	A	A	A	A	A	A	H	H	A	A	A	A	A	A	A	A	A	A	A	A	A	A	A		
RM6395	6	26.00	A	A	A	A	A	A	A	A	A	A	A	A	A	A	A	H	A	A	A	A	A	A	A	A	A	A	H	A	A	A	A	2/2 (red)	1/2 (red)
RM3394	7	0.65	A	A	A	A	A	A	A	A	A	A	A	A	A	A	A	A	H	A	A	A	A	A	A	A	A	A	H	A	A	A	A		
RM1243	7	3.56	A	A	A	A	A	A	A	A	A	A	A	A	A	A	A	A	H	A	A	A	A	A	A	A	A	A	H	A	A	A	A		1/2 (red)
RM8006	7	7.69	A	A	A	A	A	A	A	A	A	A	A	A	A	A	A	A	H	H	A	A	A	A	A	A	A	A	A	A	A	A	A	2/2 (red)	
RM3635	7	11.09	A	A	A	A	A	A	A	A	A	A	A	A	A	A	A	A	-	H	A	A	A	A	A	A	A	A	A	A	A	A	A		
RM3670	7	13.44	A	A	A	A	A	A	A	A	A	A	A	A	A	A	A	A	A	H	A	A	A	A	A	A	A	A	A	A	A	A	A		
RM6767	7	17.47	A	A	A	A	A	A	A	A	A	A	A	A	A	A	A	A	A	H	H	A	A	A	A	A	A	A	A	A	A	A	A	2/2 (red)	2/2 (red)
RM1279	7	21.61	A	A	A	A	A	A	A	A	A	A	A	A	A	A	A	A	A	A	H	A	A	A	A	A	A	A	A	A	A	A	A		
RM1330	7	25.66	A	A	A	A	A	A	A	A	A	A	A	A	A	A	A	A	A	A	H	A	A	A	A	A	A	A	A	A	-	-	A		
RM7601	7	29.04	A	A	A	A	A	A	A	A	A	A	A	A	A	A	A	A	A	A	H	A	A	A	A	A	A	A	A	A	H	H	A		
RM6369	8	0.13	A	A	A	A	A	A	A	A	A	A	A	A	A	A	A	A	A	A	A	H	A	H	A	A	A	A	A	A	A	A	A		
RM6999	8	3.99	A	A	A	A	A	A	A	A	A	A	A	A	A	A	A	A	A	A	A	H	A	A	A	A	A	A	A	A	A	A	A		
RM2584	8	7.57	A	A	A	A	A	A	A	A	A	A	A	A	A	A	A	A	A	A	A	H	H	A	A	A	A	A	A	A	A	A	A		
RM6032	8	11.90	A	A	A	A	A	A	A	A	A	A	A	A	A	A	A	A	A	A	A	A	H	A	A	A	A	A	A	A	A	A	A		
RM6990	8	14.36	A	A	A	A	A	A	A	A	A	A	A	A	A	A	A	A	A	A	A	A	H	A	A	A	A	A	A	A	A	A	A		
RM7285	8	18.76	A	A	A	A	A	A	A	A	A	A	A	A	A	A	A	A	A	A	A	A	H	H	A	A	A	A	A	A	A	A	A		2/2 (red)
RM6635	8	22.17	A	A	A	A	A	A	A	A	A	A	A	A	A	A	A	A	A	A	A	A	H	H	A	A	A	A	A	A	A	A	A		
RM1345	8	26.15	A	A	A	A	A	A	A	A	A	A	H	A	A	H	A	A	A	A	A	A	H	-	A	A	A	A	A	A	A	A	A	3/3 (red)	
RM4997	8	28.24	A	A	A	A	A	A	A	A	A	A	H	A	A	H	A	A	A	A	A	A	H	A	A	A	A	A	A	A	A	A	A		2/3 (blue)
RM3609	9	1.16	A	A	A	A	A	H	A	A	A	A	A	A	A	A	A	A	A	A	A	H	A	A	A	A	A	A	A	A	A	A	A		
RM5799	9	3.80	A	A	A	A	A	A	A	A	A	A	A	A	A	A	A	A	A	A	A	H	A	A	-	A	A	A	A	A	A	A	A		1/1 (red)
RM5515	9	7.15	A	A	A	A	A	A	A	A	A	A	A	A	A	A	A	A	A	A	A	H	A	A	H	A	A	A	A	A	A	A	A		
RM3769	9	11.75	A	A	A	A	A	A	A	A	A	A	A	A	A	A	A	A	A	A	A	H	A	A	H	-	A	A	A	A	A	-	A	1/2 (red)	
RM5122	9	15.25	A	A	A	A	A	A	A	A	A	A	A	A	A	A	A	A	A	A	A	A	A	A	H	A	A	A	A	A	A	A	A		
RM5661	9	18.96	A	A	A	A	A	A	A	A	A	A	A	A	A	A	A	A	A	A	A	A	A	A	H	A	A	A	A	A	A	A	A		
RM5095	10	0.05	A	A	A	A	A	A	A	A	A	A	A	A	A	A	A	A	A	H	A	A	H	H	A	H	A	A	A	A	A	A	A		
RM3882	10	2.74	A	A	A	A	A	A	A	A	A	A	A	A	A	A	A	A	A	-	A	A	-	-	A	H	A	A	A	A	A	A	A		
RM25101	10	5.72	A	A	A	A	A	A	A	A	A	A	A	A	A	A	A	A	A	A	A	A	A	A	A	H	A	A	A	A	A	A	A		
RM25217	10	9.75	A	A	A	A	A	A	A	A	A	A	A	A	A	A	A	A	A	A	A	A	A	A	A	H	A	A	A	A	A	A	A		
RM5758	10	12.99	A	A	A	A	A	A	A	A	A	A	A	A	A	A	A	A	A	A	A	A	A	A	A	H	A	A	A	A	A	-	A		
RM1375	10	16.72	A	A	A	A	A	A	A	A	A	A	A	A	A	A	A	A	A	A	A	A	A	A	A	H	A	A	A	A	A	H	A		
RM4477	10	21.17	A	A	A	A	A	A	A	A	A	A	A	A	A	A	A	A	A	A	A	A	A	A	A	-	A	A	-	A	-	H	A		
RM6824	10	23.11	A	A	A	A	A	A	A	A	A	A	A	A	A	A	A	A	A	A	A	A	A	A	A	A	A	A	H	A	H	H	A	1/3 (blue)	
RM3863	11	0.90	A	A	A	A	A	A	A	A	A	A	A	A	A	A	A	H	A	A	A	A	A	A	A	A	H	A	A	A	A	A	A		
RM3339	11	4.14	A	A	A	A	A	A	A	A	A	A	A	A	A	A	A	A	A	A	A	A	A	A	A	A	H	A	A	A	A	A	A		
RM3701	11	8.11	A	A	A	A	A	A	A	A	A	A	A	A	A	A	A	A	A	A	A	A	A	A	A	A	H	A	A	A	A	A	A	1/1 (red)	
RM5857	11	11.86	A	A	A	A	A	A	A	A	A	A	A	A	A	A	A	A	A	A	A	A	A	A	A	A	H	A	A	A	A	A	A		
RM5824	11	14.53	A	A	A	A	A	A	A	A	A	A	A	A	A	A	A	A	A	A	A	A	A	A	A	A	H	-	A	A	A	A	A		
RM1355	11	17.67	A	A	A	A	A	A	A	A	A	A	A	A	A	A	A	A	A	A	A	A	A	A	A	A	H	-	A	A	A	A	A		
RM1341	11	20.14	A	A	A	A	A	A	A	A	A	A	A	A	A	A	A	A	A	A	A	A	A	A	A	A	H	H	A	A	A	A	A		
RM4112	11	25.11	A	A	A	A	A	A	A	A	A	A	A	A	A	A	A	A	A	A	A	A	A	A	A	A	A	H	A	A	A	A	A		
RM5926	11	28.96	A	A	A	A	A	A	A	A	A	A	A	A	A	A	A	A	A	A	A	A	A	H	A	A	A	H	H	A	A	A	A		
RM27400	12	0.11	A	A	A	A	A	A	A	A	A	A	A	A	A	A	A	A	A	A	A	A	A	A	A	A	A	A	A	H	-	A	A		
RM27414	12	0.40	A	A	A	A	A	A	A	A	A	A	A	A	A	A	A	A	A	A	A	A	A	A	A	A	A	A	A	H	H	A	A		
RM27574	12	3.29	-	A	A	A	A	A	A	A	A	A	A	A	A	A	A	A	A	A	A	A	A	A	A	A	A	A	A	H	H	A	A		2/2 (red)
RM2935	12	7.43	A	A	A	A	A	A	A	A	A	A	A	A	A	A	A	A	A	A	A	A	A	A	A	A	A	A	A	A	H	A	A		
RM27943	12	10.86	A	A	A	A	A	A	A	A	A	A	A	A	A	A	A	A	A	A	A	A	A	A	A	A	A	A	A	A	H	H	A		
RM28070	12	14.93	A	A	A	A	A	A	A	A	A	A	A	A	A	A	A	A	A	A	A	A	A	A	A	A	A	A	A	A	H	H	A		
RM28195	12	18.11	A	A	A	A	A	A	A	A	A	A	A	A	A	A	A	A	-	A	A	A	A	A	A	A	A	A	A	A	-	H	-		
RM3331	12	23.49	A	A	A	A	A	A	A	A	A	A	A	A	A	A	A	A	H	A	A	A	A	A	A	A	A	A	A	A	A	H	H		1/3 (blue)
RM1226	12	27.34	A	A	A	A	A	A	A	A	A	A	A	A	A	A	A	A	A	A	A	A	A	A	A	H	A	A	A	A	A	A	H		

[a]Y indicates the presence, and N the absence, of a QTL.

[b]A indicates a homozygous Koshihikari allele; H indicates a heterozygous allele.

[c] QTLs with Koshihikari alleles that decrease trait values are shown in red boxes, while those that increase trait values are shown in blue boxes.
The numbers to the right of each box indicate the number of populations in which the QTL was detected, relative to the total number of populations examined.

Supplemental Table 7. Mean grain length and grain width in 33 QZK-BC$_4$F$_2$ populations, along with graphical genotypes of their parental BC$_4$F$_1$ plants.

Line name			14-2509	14-2510	14-2511	14-2512	14-2513	14-2514	14-2515	14-2516	14-2517	14-2518	14-2519	14-2520	14-2521	14-2522	14-2523	14-2524	14-2525	14-2526	14-2527	14-2528	14-2529	14-2530	14-2531	14-2532	14-2533	14-2534	14-2535	14-2537	14-2538	14-2539	14-2540	14-2541	14-2542	Positions of QTL[c] L	Positions of QTL[c] W
Grain length	Mean±SD (mm)		7.06±0.34	6.84±0.32	7.12±0.32	7.08±0.32	7.03±0.34	7.17±0.33	7.10±0.32	6.96±0.32	7.13±0.32	7.03±0.34	7.35±0.34	6.98±0.32	6.98±0.33	7.05±0.32	7.30±0.32	7.11±0.32	7.09±0.32	7.01±0.33	6.89±0.33	7.04±0.33	7.14±0.32	6.93±0.32	7.07±0.32	7.07±0.32	7.19±0.34	7.02±0.32	7.05±0.35	6.90±0.34	6.98±0.32	6.86±0.33	7.13±0.34	7.02±0.33	6.87±0.32		
	QTL detection (Y/N)[a]		N	Y	Y	N	Y	Y	Y	Y	Y	N	Y	N	Y	Y	Y	Y	Y	Y	Y	Y	Y	Y	Y	Y	Y	N	N	N	N	Y	Y	N	Y		
Grain width	Mean±SD (mm)		3.46±0.15	3.33±0.14	3.41±0.15	3.25±0.14	3.31±0.16	3.41±0.15	3.40±0.14	3.37±0.15	3.34±0.16	3.41±0.15	3.09±0.15	3.37±0.17	3.41±0.16	3.47±0.15	3.10±0.17	3.43±0.16	3.46±0.15	3.41±0.15	3.39±0.16	3.48±0.16	3.44±0.15	3.46±0.16	3.49±0.15	3.45±0.15	3.40±0.19	3.37±0.16	3.36±0.16	3.36±0.16	3.37±0.15	3.34±0.15	3.40±0.17	3.36±0.17	3.33±0.17		
	QTL detection (Y/N)[a]		Y	N	N	N	Y	Y	Y	Y	Y	N	Y	Y	Y	Y	Y	Y	Y	Y	N	Y	Y	Y	Y	Y	Y	N	Y	Y	Y	Y	N	N	Y		
Genotypes[b]																																					
Marker	Chr. No.	Position (Mb)																																		L	W
RM3252	1	0.30	H	A	A	A	A	A	A	A	A	A	A	A	A	A	A	A	A	A	A	A	A	A	A	A	A	A	A	A	A	A	A	A	A		1/1 (red)
RM5302	1	4.15	H	A	A	A	A	A	A	A	A	A	A	A	A	A	A	A	A	A	A	A	A	A	A	A	A	A	A	A	A	A	A	A	A		
RM5800	1	7.40	H	A	A	A	A	A	A	A	A	A	A	A	A	A	A	A	A	A	A	A	A	A	A	A	A	A	A	A	A	A	A	A	A		
RM3627	1	10.31	H	A	A	A	A	A	A	A	A	A	A	A	A	A	A	A	A	A	A	A	A	A	A	A	A	A	A	A	A	A	A	A	A		
IDR0045	1	11.63	-	H	A	A	A	A	A	A	A	A	A	A	A	A	A	A	A	A	A	A	A	A	A	A	A	A	A	A	A	A	A	A	H		
RM7075	1	15.12	A	H	A	A	A	A	A	A	A	A	A	A	A	A	A	A	A	A	A	A	A	A	A	A	A	A	A	A	A	A	A	A	H	2/2 (blue)	
RM5422	1	18.76	A	H	A	A	A	A	A	A	A	A	A	A	A	A	A	A	A	A	A	A	A	A	A	A	A	A	A	A	A	A	A	A	H		
RM1196	1	21.90	H	H	H	A	A	A	H	A	A	A	A	A	A	A	A	A	A	H	A	A	A	A	A	A	A	A	A	A	A	A	A	A	H	2/6 (blue)	
RM7266	1	24.45	-	-	H	-	A	A	-	A	-	A	A	A	A	A	A	-	A	-	A	A	A	A	A	A	A	A	A	A	A	A	A	A	-		
RM5461	1	26.91	A	A	H	H	A	A	A	A	H	A	A	A	A	A	A	H	A	A	A	A	A	A	A	A	A	A	A	A	A	A	A	A	A		1/4 (red)
IDR0129	1	29.90	A	A	H	H	A	A	A	A	H	A	A	A	A	A	A	H	A	A	A	A	A	A	A	A	A	A	A	A	A	A	A	A	A	3/4 (red)	
RM3416	1	32.89	A	A	H	H	A	A	A	A	H	A	A	A	A	A	A	H	A	A	A	A	A	A	A	A	A	A	A	A	A	A	A	A	A		
RM6547	1	34.69	A	A	H	H	A	A	A	A	H	A	A	A	A	A	A	-	A	A	A	A	A	A	A	A	A	A	A	A	A	A	A	A	A		1/3 (blue)
RM5448	1	37.40	A	A	H	H	A	A	A	A	H	A	A	A	A	A	H	A	A	A	A	A	A	A	A	A	A	A	A	A	A	A	A	A	A	3/4 (blue)	
RM5310	1	41.20	A	A	A	H	A	A	A	A	-	A	A	A	A	A	H	A	H	A	A	A	A	A	A	A	A	A	A	A	A	A	A	A	A		
RM6831	1	43.18	A	A	A	H	A	A	A	A	A	A	A	A	A	A	-	A	-	A	A	H	A	A	A	A	A	A	A	A	A	A	A	A	A		
RM3703	2	3.86	A	A	A	A	H	A	A	A	A	A	A	A	A	A	A	A	A	A	A	A	A	A	A	A	A	A	A	A	A	A	A	A	A		
RM5340	2	7.48	A	A	A	A	H	A	A	A	A	A	A	A	A	A	A	A	A	A	A	A	A	A	A	A	A	A	A	A	A	A	A	A	A		1/1 (blue)
IDR0237	2	10.81	A	A	A	A	H	H	A	A	A	A	A	A	A	A	A	A	A	A	A	A	A	A	A	A	H	A	A	A	A	A	A	A	A	2/3 (red)	
IDR0248	2	13.59	A	A	A	A	H	H	A	A	A	A	A	A	A	A	A	A	A	A	A	A	A	A	A	A	H	A	A	A	A	A	A	A	A		3/3 (blue)
IDR0271	2	16.25	A	A	A	A	-	H	-	A	A	A	A	A	A	A	A	A	A	A	A	A	A	A	A	A	-	A	A	A	A	A	A	A	A		
RM3858	2	19.19	A	A	A	A	A	H	H	A	A	A	A	A	A	A	A	A	A	A	A	A	A	A	A	A	A	A	A	A	A	A	A	A	A		2/2 (blue)
IDR0301	2	22.05	A	A	A	A	A	A	H	A	A	A	A	A	A	A	A	A	A	A	A	A	A	A	A	A	A	A	A	A	A	A	A	A	A		
IDR0315	2	26.39	A	A	A	A	A	A	H	A	A	A	A	A	A	A	A	A	A	A	A	A	A	A	A	A	A	A	A	A	A	A	A	A	A		
RM6933	2	29.31	A	A	A	A	A	A	H	H	A	A	A	A	A	A	A	A	A	A	A	A	A	A	A	A	A	A	A	A	A	A	A	A	A	1/2 (red)	
RM6636	2	33.08	A	A	A	A	A	A	A	H	A	A	A	A	A	A	A	A	A	A	A	A	A	A	A	A	A	A	A	A	A	A	A	A	A		1/1 (blue)
IDR0351	2	35.93	A	A	A	A	A	A	A	H	A	A	A	A	A	A	A	A	A	A	A	A	A	A	A	A	A	A	A	A	A	A	A	A	A		
IDR0352	3	0.12	A	A	A	A	A	A	A	A	H	A	A	A	A	A	H	A	H	A	A	A	A	A	A	A	A	A	A	A	A	A	A	A	A	3/3 (red)	3/3 (red)
RM6849	3	3.29	A	A	A	A	A	A	A	A	H	A	-	A	A	A	H	A	A	A	A	A	A	A	A	A	A	A	A	A	A	A	A	A	A		
RM1022	3	7.23	A	A	A	A	A	A	A	A	H	A	H	A	A	A	H	A	A	A	A	A	A	A	A	A	A	A	A	A	A	A	A	A	A		
RM1284	3	10.62	A	A	A	A	A	A	A	A	H	A	H	A	A	A	-	A	A	A	A	A	A	A	A	A	A	A	A	A	A	A	A	A	A		2/2 (blue)
RM6080	3	13.93	A	A	A	A	A	A	A	A	H	A	H	A	A	A	A	A	A	A	A	A	A	A	A	A	A	A	A	A	A	A	A	A	A		
IDR0421	3	15.87	A	A	A	A	A	A	A	A	H	A	H	A	A	A	A	A	A	A	A	A	A	A	A	A	A	A	A	A	A	A	A	A	A		
RM7431	3	19.52	A	A	A	A	A	A	A	A	A	H	H	A	A	A	A	A	A	A	A	A	A	A	A	A	A	A	A	A	A	A	A	A	A		
RM6832	3	22.40	A	A	A	A	A	A	A	A	A	H	H	A	A	A	A	A	A	A	A	A	A	A	A	A	A	A	A	A	A	A	A	A	A		
IDR0460	3	24.17	A	A	A	A	A	A	A	A	A	H	H	A	A	A	A	A	A	A	A	A	A	A	A	A	A	A	A	A	A	A	A	A	A		
RM2334	3	26.75	A	A	A	A	A	A	A	A	A	H	H	A	A	A	A	A	A	A	A	A	A	A	A	A	A	A	A	A	A	A	A	A	A		
RM1350	3	28.68	A	A	A	A	A	A	A	A	A	A	H	A	A	A	A	A	A	A	A	A	A	A	A	A	A	A	A	A	A	A	A	A	A	1/1 (red)	1/1 (red)
RM3525	3	30.39	A	A	A	A	A	A	A	A	A	A	H	A	A	A	A	A	A	A	A	A	A	A	A	A	A	A	A	A	A	A	A	A	A		
RM3564	3	33.43	A	A	A	A	A	A	A	A	A	A	H	A	A	A	A	A	A	A	A	A	A	A	A	A	A	A	A	A	A	A	A	A	A		
IDR0514	3	36.41	A	A	A	A	A	A	A	A	A	A	H	A	A	A	A	A	A	A	A	A	A	A	A	A	A	A	A	A	A	A	A	A	A		
RM16252	4	0.06	A	A	A	A	A	A	A	A	A	A	A	H	-	-	A	A	A	A	A	A	A	A	A	A	A	A	A	A	A	A	A	A	A		1/1 (red)
IDR0531	4	3.65	A	A	A	A	A	A	A	A	A	A	A	H	A	A	A	A	A	A	A	A	A	A	A	A	A	A	A	A	A	A	A	A	A		
IDR0541	4	5.99	A	A	A	A	A	A	A	A	A	A	A	H	H	A	A	A	A	A	A	A	A	A	A	A	A	A	A	A	A	A	A	A	A		
IDR0561	4	9.72	A	A	A	A	A	A	A	A	A	A	A	H	H	A	A	A	A	A	A	A	A	A	A	A	A	A	A	A	A	A	A	A	A		
RM5633	4	13.08	A	A	A	A	A	A	A	A	A	A	A	H	H	A	A	A	A	A	A	A	A	A	A	A	A	A	A	A	A	A	A	A	A		
IDR0596	4	16.34	A	A	A	A	A	A	A	A	A	A	A	H	H	A	A	A	A	A	A	A	A	A	A	A	A	A	A	A	A	A	A	A	A		
IDR0606	4	18.14	A	A	A	A	A	A	A	A	A	A	A	A	H	A	A	A	A	A	A	A	A	A	A	A	A	A	A	A	A	A	A	A	A		
RM3308	4	19.18	A	A	A	A	A	A	A	A	A	A	A	H	H	A	A	A	A	A	A	A	A	A	A	A	A	A	A	A	A	A	A	A	A		2/2 (blue)
RM3708	4	20.83	A	A	A	A	A	A	A	A	A	A	A	A	H	H	A	A	A	A	A	A	A	A	A	A	A	A	A	A	A	A	A	A	A		
RM3524	4	22.89	A	A	A	A	A	A	A	A	A	A	A	A	H	H	A	A	A	A	A	A	A	A	A	A	A	A	A	A	A	A	A	A	A	1/2 (red)	
IDR0636	4	25.83	A	A	A	A	A	A	A	A	A	A	A	A	H	H	A	A	A	A	A	A	A	A	A	A	A	A	A	A	A	A	A	A	A		
RM3916	4	28.76	A	A	A	A	A	A	A	A	A	A	A	A	H	H	A	A	A	A	A	A	A	A	A	A	A	A	A	A	A	A	A	A	A		
RM6909	4	32.06	A	A	A	A	A	A	A	A	A	A	A	A	H	H	A	A	A	A	A	A	A	A	A	A	A	A	A	A	A	A	A	A	A		2/2 (red)
RM1248	5	0.09	A	A	A	A	A	A	A	A	A	A	A	A	A	A	H	A	A	A	A	A	A	A	A	A	H	A	A	A	A	A	A	A	A		
IDR0688	5	3.31	A	A	A	A	A	A	A	A	A	A	H	A	A	A	H	A	A	A	A	A	A	A	A	A	A	A	A	A	A	A	A	A	A		
IDR0702	5	5.79	A	A	A	A	A	A	A	A	A	A	-	A	A	A	H	-	A	A	A	A	A	A	A	A	A	A	A	A	A	A	A	A	A	1/1 (red)	1/1 (blue)
RM6082	5	8.90	A	A	A	A	A	A	A	A	A	A	A	A	A	A	H	H	A	A	A	A	A	A	A	A	A	A	A	A	A	A	A	A	A		
RM6724	5	13.83	A	A	A	A	A	A	A	A	A	A	A	A	A	A	H	H	A	A	A	A	A	A	A	A	A	A	A	A	A	A	A	A	A		
IDR0755	5	16.74	A	A	A	A	A	A	A	A	A	A	A	A	A	A	A	H	H	A	A	A	A	A	A	A	A	A	A	A	A	A	A	A	A	1/2 (red)	
IDR0769	5	19.98	A	A	A	A	A	A	A	A	A	A	A	A	A	A	A	A	H	A	A	A	A	A	A	A	A	A	A	A	A	A	A	A	A		
RM3870	5	22.96	A	A	A	A	A	A	A	A	A	A	A	A	A	A	A	A	H	A	A	A	A	A	A	A	A	A	A	A	A	A	A	A	A		
IDR0793	5	26.02	A	A	A	A	A	A	A	A	A	A	A	A	A	A	A	A	H	A	A	A	A	A	A	A	A	A	A	A	A	A	A	A	A		
IDR0806	5	28.97	A	A	A	A	A	A	A	A	A	A	A	A	A	A	A	A	H	A	A	A	A	A	A	A	A	A	A	A	A	A	A	A	A		
RM7158	6	0.22	A	A	A	A	A	A	H	A	A	A	A	A	A	A	A	A	A	H	A	A	A	A	H	A	A	A	A	A	A	A	A	A	A	3/3 (blue)	
RM3805	6	2.85	A	A	A	A	A	A	H	A	A	A	A	A	A	A	A	A	A	H	-	A	A	A	H	A	A	A	A	A	A	A	A	A	A		2/3 (red)
IDR0829	6	5.43	A	A	A	A	A	A	H	A	A	A	A	A	A	A	A	A	A	H	H	A	A	A	A	A	A	A	A	A	A	A	A	A	A		
RM5963	6	8.82	A	A	A	A	A	A	A	A	H	A	A	A	H	A	A	H	H	A	H	A	A	A	A	H	A	A	A	A	A	A	A	A	A		2/6 (red)
IDR0858	6	12.25	A	A	A	A	A	A	A	H	A	A	A	A	A	A	A	A	A	A	H	A	A	A	A	H	A	A	A	A	A	A	A	A	A	3/3 (blue)	
IDR0863	6	13.49	A	A	A	A	A	A	A	H	A	A	A	A	A	A	A	A	A	A	H	A	A	A	A	H	A	A	A	A	A	A	A	A	A		
RM6818	6	16.58	A	A	A	A	A	A	A	H	A	A	A	A	A	A	A	A	A	A	H	A	A	A	A	H	A	A	A	A	A	A	A	A	A		1/3 (blue)
RM3498	6	20.98	A	A	A	A	A	A	A	H	A	A	A	A	A	A	A	A	A	A	H	A	A	A	A	-	A	A	A	A	A	A	A	A	A	2/2 (blue)	
RM7434	6	23.94	A	A	A	A	A	A	A	-	A	A	A	A	A	A	A	A	A	A	A	H	A	A	A	A	A	A	A	A	A	A	A	A	A		
IDR0931	6	27.02	A	A	A	A	A	A	A	A	A	A	A	A	A	A	A	A	A	A	A	H	A	A	A	A	A	A	A	A	A	A	A	A	A	1/1 (red)	
RM6811	6	29.23	A	A	A	A	A	A	A	A	A	A	A	A	A	A	A	A	A	A	A	H	A	A	A	A	A	A	A	A	A	A	A	A	A		1/1 (red)
IDR0948	6	31.17	A	A	A	A	A	A	A	A	A	A	A	A	A	A	A	A	A	A	A	H	A	A	A	A	A	A	A	A	A	A	A	A	A		
RM5711	7	3.14	A	A	A	A	A	A	A	A	A	A	A	A	A	A	A	A	A	A	A	A	H	H	A	A	A	A	A	H	A	A	A	A	A		1/3 (red)
RM1253	7	6.97	A	A	A	A	A	A	A	A	A	A	A	A	A	A	A	A	A	A	A	A	H	H	A	A	A	A	A	A	A	A	A	A	A		
RM5499	7	9.99	A	A	A	A	A	A	A	A	A	A	A	A	A	A	A	A	A	A	A	A	H	H	A	A	A	A	A	A	A	A	A	A	A		
RM7273	7	13.43	A	A	A	A	A	A	A	A	A	A	A	A	A	A	A	A	A	A	A	A	H	H	A	A	A	A	A	A	A	A	A	A	A	2/2 (blue)	
IDR1024	7	17.00	A	A	A	A	A	A	A	A	A	A	A	A	A	A	A	A	A	A	A	A	H	A	A	A	-	A	A	A	A	A	-	A	A		
IDR1040	7	20.13	A	A	A	A	A	A	A	A	A	A	A	A	A	A	A	A	A	A	A	A	H	A	A	A	H	A	A	A	A	A	H	A	A		
RM5508	7	23.56	A	A	A	A	A	A	A	A	A	A	A	A	A	A	A	A	A	A	A	A	H	A	A	A	A	A	A	A	A	A	A	A	A	1/1 (red)	
RM6216	7	26.40	A	A	A	A	A	A	A	A	A	A	A	A	A	A	A	A	A	A	A	A	H	A	A	A	A	A	A	A	A	A	A	A	A		
IDR1081	7	29.43	A	A	A	A	A	A	A	A	A	A	A	A	A	A	A	A	A	A	A	A	H	A	A	A	A	A	A	A	A	A	A	A	A		
RM5911	8	0.08	A	A	A	A	A	A	A	A	A	H	A	A	A	A	A	A	A	A	A	A	A	A	A	A	H	A	A	A	A	A	A	A	A		
RM3819	8	3.02	A	A	A	A	A	A	A	A	A	H	A	A	A	A	A	A	A	A	A	A	A	A	A	A	A	A	A	A	A	A	A	A	A		
RM6838	8	5.85	A	A	A	A	A	A	A	A	A	H	A	A	A	A	A	A	A	A	A	A	A	A	H	A	A	A	A	A	A	A	A	A	A		
RM6027	8	9.11	A	A	A	A	A	A	A	A	A	-	A	A	A	A	A	A	A	A	A	A	A	A	H	A	A	A	A	A	A	A	A	A	A		
IDR1139	8	12.46	A	A	A	A	A	A	A	A	A	A	A	A	A	A	A	A	A	A	A	A	A	A	H	A	A	A	A	A	A	A	A	A	A		
IDR1161	8	15.50	A	A	A	A	A	A	A	A	A	A	A	A	A	A	A	A	A	A	A	A	A	A	H	A	A	A	A	A	A	A	A	A	A		
RM3491	8	19.62	A	A	A	A	A	A	A	A	A	A	A	A	A	A	A	A	A	A	A	A	A	A	H	A	A	A	A	A	A	A	A	A	A		1/1 (red)
IDR1186	8	22.48	A	A	A	A	A	H	A	A	A	A	A	A	A	A	A	A	A	A	A	A	A	A	H	A	A	A	A	A	A	A	A	A	A	1/2 (red)	
IDR1200	8	25.74	A	A	A	-	A	H	A	A	A	A	A	A	A	A	A	A	A	A	A	A	A	A	A	A	A	A	A	A	A	A	A	A	A		
IDR1209	8	28.10	A	A	A	H	A	H	A	A	A	A	A	A	A	A	A	A	A	A	A	A	A	A	A	A	A	A	A	A	A	A	A	A	A		
IDR1212	9	0.50	A	A	A	A	A	A	A	A	A	A	A	A	A	A	A	A	A	A	A	A	A	A	A	H	A	A	A	A	A	A	A	A	A		
IDR1231	9	3.91	A	A	A	A	A	A	A	A	A	A	A	A	A	A	A	A	A	A	A	A	A	A	A	H	A	A	A	A	A	A	A	A	A		1/1 (red)
IDR1250	9	7.64	A	A	A	A	A	A	A	A	A	A	A	A	A	A	A	A	A	A	A	A	A	A	A	H	A	A	A	A	A	A	A	A	A		
IDR1269	9	11.20	A	A	A	A	A	A	A	A	A	A	A	A	A	A	A	A	A	A	A	A	A	A	A	H	A	A	A	A	A	A	A	A	A	1/1 (red)	
RM3700	9	15.43	A	A	A	A	A	A	A	A	A	A	A	A	A	A	A	A	A	A	A	H	A	A	A	H	H	A	A	A	A	A	A	A	A		1/3 (red)
IDR1301	9	18.57	A	A	A	A	A	A	A	A	A	A	A	A	A	A	A	A	A	A	A	H	A	A	A	-	H	A	A	A	A	A	A	A	A		
RM3808	9	20.55	A	A	A	A	A	A	A	A	A	A	A	A	A	A	A	A	A	A	A	H	A	A	A	A	H	A	A	A	A	A	A	A	A	1/2 (blue)	
IDR1314	9	22.91	A	A	A	A	A	A	A	A	A	A	A	A	A	A	A	A	A	A	A	A	A	A	A	A	H	A	A	A	A	A	A	A	A		
RM7492	10	0.05	A	A	A	A	A	A	A	A	H	A	A	A	A	A	A	A	A	A	A	A	A	A	A	A	A	H	A	A	A	A	A	A	A		
RM24955	10	2.16	A	A	A	A	A	A	A	A	A	A	A	A	A	A	A	A	A	A	A	A	A	A	A	A	A	H	A	A	A	A	A	A	A		
IDR1336	10	4.85	A	A	A	A	A	A	A	A	A	A	A	A	A	A	A	A	A	A	A	A	A	A	A	A	A	A	H	A	A	A	A	A	A		
IDR1356	10	7.55	A	A	A	A	A	A	A	A	A	A	A	A	A	A	A	A	A	A	A	A	A	A	A	A	A	A	H	A	A	A	A	A	A		
RM25158	10	7.94	A	A	A	A	A	A	A	A	A	A	A	A	A	A	A	A	A	A	A	A	A	A	A	A	A	A	H	A	A	A	A	A	A		
RM25302	10	12.03	A	A	A	A	A	A	A	A	A	A	A	A	A	A	A	A	A	H	A	A	A	A	A	A	A	A	H	A	A	H	A	A	A		2/3 (blue)
IDR1389	10	15.24	A	A	A	A	A	A	-	A	A	A	A	A	A	A	A	A	A	A	A	A	A	A	A	A	A	A	H	A	A	H	A	A	A		
RM6737	10	18.78	A	A	A	A	A	A	A	A	A	A	A	A	A	A	A	A	A	A	A	A	A	A	A	A	A	A	H	A	A	H	A	A	A		
IDR1413	10	21.65	A	A	A	A	A	A	A	A	A	A	A	A	A	A	A	A	A	A	A	A	A	A	A	A	A	A	H	A	A	H	A	A	A		
RM6824	10	23.11	A	A	A	A	A	A	A	A	A	A	A	A	A	A	A	A	A	A	A	A	A	A	A	A	A	A	H	A	A	H	A	A	A		
IDR1420	11	0.22	A	A	A	A	A	A	A	A	A	A	A	A	A	A	A	A	A	A	A	A	A	A	A	A	A	A	A	H	A	A	A	A	A		1/1 (blue)
IDR1431	11	3.25	A	A	A	A	A	A	A	A	A	A	A	A	A	A	A	A	A	A	A	A	A	A	A	A	A	A	A	H	A	A	A	A	A		
RM3137	11	6.19	A	A	A	A	A	A	A	A	A	A	A	A	A	A	A	A	A	A	A	A	A	A	A	A	A	A	A	H	-	A	A	A	A		
RM7283	11	9.12	A	A	A	A	A	A	A	A	A	A	A	A	A	A	A	A	A	A	A	A	A	A	A	A	A	A	A	H	H	A	A	A	A		
IDR1473	11	12.84	A	A	A	A	A	A	A	A	A	A	A	A	A	A	A	A	A	A	A	A	A	A	A	A	A	A	A	H	H	A	A	A	A		
IDR1487	11	15.37	A	A	A	A	A	A	A	A	A	A	A	A	A	A	A	A	A	A	A	A	A	A	A	A	A	A	A	H	H	A	A	A	A		
RM1355	11	17.67	A	A	A	A	A	A	A	A	A	A	A	A	A	A	A	A	A	A	A	A	A	A	A	A	A	A	A	H	H	A	A	A	A		
RM1219	11	21.52	A	A	A	A	A	A	A	A	A	A	A	A	A	A	A	A	A	A	A	A	A	A	A	A	A	A	A	-	H	A	A	A	A		
IDR1519	11	23.15	A	A	A	A	A	A	A	A	A	A	A	A	A	A	A	A	A	A	A	A	A	A	A	A	A	A	A	A	H	A	A	A	A		
IDR1533	11	26.03	A	A	A	A	A	A	A	A	A	A	A	A	A	A	A	A	A	A	A	A	A	A	A	A	A	A	A	A	H	A	A	A	A		1/1 (blue)
IDR1548	11	28.99	A	A	A	A	A	A	A	A	A	A	A	A	A	A	A	A	A	A	A	A	A	A	A	A	A	A	A	A	A	H	A	A	A		
IDR1549	12	0.10	A	A	A	A	A	A	A	A	A	A	A	A	A	A	A	A	A	A	A	A	A	A	A	H	A	A	A	A	A	A	H	A	A		
RM27593	12	3.43	A	A	A	A	A	A	A	A	A	A	A	A	A	A	A	A	A	A	A	A	A	A	A	H	A	A	A	A	A	A	H	A	A		
RM27792	12	7.10	A	A	A	A	A	A	A	A	A	A	A	A	A	A	A	A	A	A	A	A	A	A	A	A	A	A	A	A	A	A	H	A	A		
RM27924	12	10.21	A	A	A	A	A	A	A	A	A	A	A	A	A	A	A	A	A	A	A	A	A	A	A	A	A	A	A	A	A	A	H	H	H		
RM28037	12	13.74	A	A	A	A	A	A	A	A	A	A	A	A	A	A	A	A	A	A	A	A	A	A	A	A	A	A	A	A	A	A	H	H	H		
IDR1629	12	16.58	A	A	A	A	A	A	A	A	A	A	A	A	A	A	A	A	A	A	A	A	A	A	A	A	A	A	A	A	A	A	H	H	H		
RM28305	12	19.96	A	A	A	A	-	A	A	A	A	A	A	A	A	A	A	A	A	A	A	A	A	A	A	A	A	A	A	A	A	A	H	H	H		
RM6947	12	24.01	A	A	A	A	-	A	A	A	A	A	A	A	A	A	A	A	A	A	A	A	A	A	A	A	A	A	A	A	A	H	-	-	H	1/2 (blue)	2/2 (blue)
IDR1672	12	27.52	A	A	A	A	A	A	A	A	A	A	A	A	A	A	A	A	A	A	A	A	A	A	A	A	A	A	A	A	A	-	A	A	H		

[a]Y, presence, and N, absence of a QTL.

[b]A, Homozygous Koshihikari allele; H, heterozygous.

[c]QTLs with Koshihikari alleles that decrease the values are shown in red boxes, while those that increase the values are shown in blue boxes.
The numbers to the right of each box indicate the number of populations in which QTLs were detected versus the number of populations examined.

Supplemental Table 8. Mean grain length and grain width in 34 TUK-BC$_4$F$_2$ populations, along with graphical genotypes of their parental BC$_4$F$_1$ plants.

Line name			13-2447	13-2448	13-2449	13-2450	13-2451	13-2452	13-2453	13-2454	13-2455	13-2456	13-2457	13-2458	13-2459	13-2460	13-2461	13-2462	13-2463	13-2464	13-2465	13-2466	13-2467	13-2468	13-2469	13-2470	13-2471	13-2472	13-2473	13-2474	13-2475	13-2476	13-2477	13-2478	13-2479	13-2480	Positions of QTL[c] L	Positions of QTL[c] W
Grain length	Mean±SD (mm)		7.21±0.35	7.16±0.32	7.27±0.33	6.78±0.32	6.95±0.33	7.10±0.32	7.24±0.32	7.40±0.33	7.24±0.32	6.91±0.31	6.89±0.31	6.99±0.29	6.98±0.29	7.27±0.34	7.17±0.32	7.16±0.32	6.80±0.32	7.15±0.32	7.02±0.31	7.08±0.34	7.19±0.33	6.80±0.31	7.03±0.32	7.21±0.32	7.94±0.31	7.02±0.30	7.01±0.30	7.14±0.35	6.83±0.32	6.73±0.33	7.05±0.32	7.08±0.30	7.13±0.32	6.99±0.34		
	QTL detection (Y/N)[a]		Y	Y	Y	Y	Y	Y	Y	Y	Y	N	Y	Y	Y	Y	Y	Y	Y	N	N	Y	Y	Y	N	Y	Y	Y	Y	N	Y	Y	Y	N	Y	N		
Grain width	Mean±SD (mm)		3.50±0.15	3.39±0.14	3.38±0.16	3.32±0.15	3.35±0.15	3.29±0.15	3.38±0.14	3.50±0.15	3.43±0.15	3.41±0.15	3.34±0.13	3.44±0.14	3.32±0.15	3.22±0.15	3.18±0.15	3.49±0.14	3.42±0.15	3.33±0.15	3.44±0.14	3.37±0.14	3.48±0.14	3.39±0.13	3.45±0.15	3.39±0.14	3.30±0.14	3.45±0.14	3.20±0.14	3.37±0.15	3.37±0.14	3.35±0.15	3.15±0.15	3.45±0.14	3.39±0.14	3.37±0.14		
	QTL detection (Y/N)[a]		Y	N	Y	Y	Y	Y	Y	Y	Y	N	Y	Y	Y	Y	Y	N	N	Y	N	Y	Y	N	N	Y	Y	Y	Y	Y	Y	Y	Y	N	Y	Y		
Genotypes[b]																																						
Marker	Chr. No.	Position (Mb)																																				
RM6470	1	1.64	H	A	A	A	A	A	A	A	A	A	A	A	A	A	A	A	A	A	A	A	A	A	A	H	A	A	A	A	A	A	A	A	A	A		1/2 (red)
RM1329	1	3.95	-	A	A	A	A	A	A	A	A	A	A	A	A	A	A	A	A	A	A	A	A	A	A	H	A	A	A	A	A	A	A	A	A	A		
RM3746	1	6.16	H	A	A	A	A	A	A	A	A	A	A	A	A	A	A	A	A	A	A	A	A	A	A	H	A	A	A	A	A	A	A	A	A	A		
RM8071	1	9.49	H	H	A	A	A	A	A	A	A	A	A	A	A	A	A	A	A	A	A	A	A	A	A	A	A	A	A	A	A	A	A	A	A	A		
RM3627	1	10.31	A	H	A	A	A	A	A	A	A	A	A	A	A	A	A	A	A	A	A	A	A	A	A	A	A	A	A	A	A	A	A	A	A	A		
RM5365	1	14.52	A	H	A	A	A	A	A	A	A	A	A	A	A	A	A	A	A	A	A	A	A	A	A	A	A	A	A	A	A	A	A	A	A	A		
RM5964	1	17.58	A	H	A	A	A	A	A	A	A	A	A	A	A	A	A	A	A	A	A	A	A	A	A	A	A	A	A	A	A	A	A	A	A	A		
RM1196	1	21.90	A	H	A	A	A	A	A	A	A	A	A	A	A	A	A	A	A	A	A	A	A	A	A	A	A	A	A	A	A	A	A	A	A	A		
RM7124	1	24.39	A	H	A	A	A	A	A	A	A	A	A	A	A	A	A	A	A	A	A	A	A	A	A	A	A	A	A	A	A	A	A	A	A	A		
RM3143	1	26.82	A	H	H	A	A	A	A	A	A	A	A	A	A	A	A	A	A	A	A	A	A	A	A	A	A	A	A	A	A	A	A	A	A	A		
RM1152	1	30.09	A	H	H	A	A	A	A	A	A	A	A	A	A	A	A	A	A	A	A	A	A	A	A	A	A	A	A	A	A	A	A	A	A	A	2/2 (red)	1/2 (blue)
RM6696	1	38.22	A	A	H	A	A	A	A	A	A	A	A	A	A	A	A	A	A	A	A	A	A	A	A	A	A	A	A	A	A	A	A	A	A	A		
RM6840	1	43.17	A	A	H	A	A	A	A	A	A	A	A	A	A	A	A	A	A	A	A	A	A	A	A	A	A	A	A	A	A	A	A	A	A	A		
RM2770	2	0.81	A	A	A	H	A	A	A	A	A	A	A	A	A	A	A	A	A	A	-	A	A	A	A	A	A	A	A	A	H	H	A	A	A	A		3/3 (blue)
RM7562	2	1.88	A	A	A	-	A	A	A	A	A	A	A	A	A	A	A	A	A	A	-	A	A	A	A	A	A	A	A	A	-	H	A	A	A	A		
RM3865	2	4.41	A	A	A	H	A	A	A	A	A	A	A	A	A	A	A	A	A	A	A	A	A	A	A	A	A	A	A	A	A	-	A	A	A	A	1/1 (red)	
RM5897	2	6.73	A	A	A	H	H	H	A	A	A	A	A	A	A	A	A	A	A	A	A	A	A	A	A	A	A	A	A	A	A	A	A	A	A	A		
RM3505	2	7.54	A	A	A	H	H	-	A	A	A	A	A	A	A	A	A	A	A	A	A	A	A	A	A	A	A	A	A	A	A	A	A	A	A	A		
RM6639	2	9.42	A	A	A	H	H	-	A	A	A	A	A	A	A	A	A	A	A	A	A	A	A	A	A	A	A	A	A	A	A	A	A	A	A	A		
RM1234	2	11.34	A	A	A	H	H	H	A	A	A	A	A	A	A	A	A	A	A	A	A	A	A	A	A	A	A	A	A	A	A	A	A	A	A	A		2/3 (blue)
RM1037	2	14.59	A	A	A	-	H	H	A	A	A	A	A	A	A	A	A	A	A	A	A	A	A	A	A	A	A	A	A	A	A	A	A	A	A	A	2/2 (red)	
RM1211	2	18.46	A	A	A	A	H	H	A	A	A	A	A	A	A	A	A	A	A	A	A	A	A	A	A	A	A	A	A	A	A	A	A	A	A	A		
RM5363	2	23.00	A	A	A	A	A	H	A	A	A	A	A	A	A	A	A	A	A	A	A	A	A	A	A	A	A	A	A	A	A	A	A	A	A	A		
RM3508	2	27.08	A	A	A	A	A	H	A	A	A	A	A	A	A	A	A	A	A	A	A	A	A	A	A	A	A	A	A	A	A	A	A	A	A	A		
RM5472	2	30.64	A	A	A	A	A	H	A	A	A	A	A	A	A	A	A	A	A	A	A	A	A	A	A	A	A	A	H	A	A	A	A	A	A	A		
RM5607	2	33.27	A	A	A	A	A	H	A	A	A	-	A	A	A	A	A	A	A	A	A	A	A	A	A	A	A	A	A	A	A	A	A	A	A	A		
RM7388	2	35.48	A	A	A	A	A	H	A	A	A	A	A	A	A	A	A	A	A	A	A	A	A	A	A	A	A	A	A	A	A	A	A	A	A	A		1/1 (blue)
RM7332	3	0.41	A	A	A	A	A	A	H	H	A	A	A	A	A	A	A	H	A	A	A	A	A	H	A	A	A	A	A	A	A	A	A	A	A	A	3/4 (red)	1/4 (red)
RM6829	3	2.88	A	A	A	A	A	A	H	-	A	A	A	A	A	A	A	-	A	A	A	A	A	-	A	A	A	A	A	A	A	A	A	A	A	A		
RM4352	3	4.32	A	A	A	A	A	A	H	A	A	A	A	A	-	A	A	A	A	A	A	A	A	A	A	A	A	A	A	A	A	A	A	A	A	A		
RM5442	3	5.53	A	A	A	A	A	A	H	A	A	A	A	A	H	A	A	A	A	A	A	A	A	A	A	A	A	A	H	A	A	A	A	A	A	A	2/3 (red)	
RM5347	3	6.43	A	A	A	A	A	A	H	A	A	A	A	A	-	A	A	A	A	A	A	A	A	A	A	A	A	A	A	A	-	A	A	A	A	A		1/1 (blue)
RM5639	3	8.20	A	A	A	A	A	A	H	A	A	A	A	A	A	A	A	A	A	A	A	A	A	A	A	A	A	A	A	A	H	A	A	A	A	A		
RM5178	3	10.66	A	A	A	A	A	A	H	A	A	A	A	A	A	A	A	A	A	A	A	A	A	A	A	A	A	A	A	A	H	A	A	A	A	A		
RM5748	3	12.33	A	A	A	A	A	A	H	A	A	A	A	A	A	A	A	A	A	A	A	A	A	A	A	A	A	A	A	A	-	A	A	A	A	A		
RM6676	3	14.49	A	A	A	A	A	A	H	H	A	A	A	A	A	A	A	A	A	A	A	A	A	A	A	A	A	A	A	A	A	A	A	A	A	A		
RM1334	3	17.42	A	A	A	A	A	A	A	H	H	A	A	A	A	A	A	A	A	A	A	-	A	A	A	H	A	A	A	A	A	A	A	A	A	A		
RM3698	3	21.78	A	A	A	A	A	A	A	H	H	A	A	A	A	A	A	A	A	A	A	-	A	A	A	H	A	A	A	A	A	A	A	A	A	A		
RM3513	3	25.11	A	A	A	A	A	A	A	H	H	A	A	A	A	A	A	A	A	A	A	A	A	A	A	H	A	A	A	A	A	A	A	A	A	A		
RM1350	3	28.68	A	A	A	A	A	A	A	A	H	A	A	A	A	A	A	A	A	A	A	A	H	A	A	A	A	A	A	A	A	A	A	A	A	A	2/2 (red)	1/2 (red)
RM1238	3	32.08	A	A	A	A	A	A	A	A	H	A	A	A	A	A	A	A	A	A	A	A	H	A	A	A	A	A	A	A	A	A	A	A	A	A		
RM6970	3	32.44	H	A	A	A	A	A	A	A	H	A	A	A	A	A	A	A	A	A	A	A	-	A	A	A	A	A	A	A	A	A	A	A	A	A		
RM6135	3	35.26	H	A	A	A	A	A	A	A	H	A	A	A	A	A	A	A	A	A	A	A	A	A	A	A	A	A	A	A	A	A	A	A	A	A		
RM16252	4	0.06	A	A	A	A	A	A	A	A	A	H	A	A	A	A	H	A	A	A	A	A	A	A	A	A	A	A	A	A	A	A	A	A	A	A		
RM16354	4	2.06	A	A	A	A	A	A	A	A	A	H	H	A	A	A	H	A	A	A	A	A	A	A	A	A	A	A	A	A	A	A	A	A	A	A		1/3 (red)
RM16434	4	4.67	A	A	A	A	A	A	A	A	A	H	H	A	A	A	-	A	A	A	A	A	A	A	A	A	A	A	A	A	A	A	A	A	A	A		
RM16540	4	8.22	A	A	A	A	A	A	A	A	A	H	H	A	A	A	A	A	A	A	A	A	A	A	A	A	A	A	A	A	A	A	A	A	A	A		
RM7279	4	13.77	A	A	A	A	A	A	A	A	A	H	H	A	-	A	A	A	A	A	A	A	A	A	A	A	A	A	A	A	A	A	A	A	A	A		
RM16757	4	17.07	A	A	A	A	A	A	A	A	A	H	H	A	-	A	A	A	A	A	A	A	A	A	A	A	A	A	-	A	A	A	A	A	A	A		
RM16868	4	19.46	A	A	A	A	A	A	A	A	A	-	-	A	H	A	A	A	A	A	A	A	A	A	A	A	A	A	-	A	A	A	A	A	A	A		
RM1359	4	20.03	A	A	A	A	A	A	A	A	A	-	H	A	A	A	A	A	A	A	A	A	A	A	A	A	A	A	A	A	A	A	H	A	A	A		
RM1165	4	23.84	A	A	A	A	A	A	A	A	A	A	H	H	A	A	A	A	A	A	A	A	A	A	A	A	A	A	A	A	A	A	A	A	A	A	1/2 (red)	1/2 (red)
RM1354	4	26.30	A	A	A	A	A	A	A	A	A	A	-	H	A	A	A	A	A	A	A	A	A	A	A	A	A	A	A	A	A	A	A	A	A	A		
RM3916	4	28.76	A	A	A	A	A	A	A	A	A	A	A	H	H	A	A	A	A	A	A	A	A	A	A	A	A	A	-	A	A	A	A	A	A	A		
RM3836	4	31.81	A	A	-	A	A	A	A	A	A	A	A	A	H	A	A	A	A	A	A	A	A	A	A	A	A	A	A	A	A	A	A	-	A	A		1/2 (blue)
RM3332	4	34.90	A	A	H	A	A	A	A	A	A	A	A	A	H	A	A	A	A	A	A	A	A	A	A	A	A	A	A	A	A	A	A	-	A	A		
RM6320	5	0.47	A	A	A	A	A	A	A	A	A	A	A	A	A	H	H	A	A	A	A	A	A	A	A	A	A	A	A	A	A	A	A	A	A	A		
RM6517	5	2.91	A	A	A	A	A	A	A	A	A	A	A	A	A	-	-	A	A	A	A	A	A	A	A	A	A	A	A	A	A	A	H	A	A	A		
RM3193	5	4.97	A	A	A	A	A	A	A	A	A	A	A	A	A	H	H	A	A	A	A	A	A	A	A	A	A	A	A	A	A	A	H	A	A	A	3/3 (red)	3/3 (blue)
RM5844	5	9.15	A	A	A	A	A	A	A	A	A	A	A	A	A	H	H	A	A	A	A	A	A	A	A	A	A	A	A	A	A	A	H	A	A	A		
RM6742	5	14.88	A	A	A	A	A	A	A	A	A	A	A	A	A	H	H	A	A	A	A	A	A	A	A	A	A	A	A	A	A	A	H	A	A	A		
RM1868	5	18.46	A	A	A	A	A	A	A	A	A	A	A	A	A	H	A	-	A	A	A	A	A	A	A	A	A	A	A	A	A	A	A	A	A	A		
RM3800	5	21.45	A	A	A	A	A	A	A	A	A	A	A	A	A	H	A	H	A	A	A	A	A	A	A	A	A	A	A	A	A	A	A	A	A	-		
RM3476	5	23.91	A	A	A	A	A	A	A	A	A	A	A	A	A	-	A	-	A	A	A	A	A	A	A	A	A	A	A	A	A	A	A	A	A	H		
RM6598	5	24.13	A	A	A	A	A	A	A	A	A	A	A	A	A	H	A	H	A	H	A	A	A	A	A	A	A	A	A	A	A	A	A	A	A	-	1/3 (red)	
RM6400	5	26.55	A	A	A	A	A	A	A	A	A	A	A	A	A	A	A	H	A	H	A	A	A	A	A	A	A	A	A	A	A	A	A	A	A	H		
RM3286	5	29.54	A	A	A	A	A	A	A	A	A	A	A	A	A	A	A	H	A	H	A	A	A	A	A	A	A	A	A	A	A	A	A	A	A	H		1/3 (red)
RM6775	6	0.21	A	A	A	A	A	A	A	A	A	A	A	A	A	A	A	A	H	A	A	A	A	H	A	A	A	A	A	A	A	A	A	A	A	A	1/2 (blue)	
RM3805	6	2.85	A	A	A	A	A	A	A	A	A	A	A	A	A	A	A	A	H	-	A	A	A	-	A	A	A	A	A	A	A	A	A	A	A	A		
RM4608	6	4.30	A	A	A	A	A	A	A	A	A	A	A	A	A	A	A	A	H	-	A	A	A	H	A	A	A	A	A	A	A	A	A	A	A	A		
RM6359	6	6.44	A	A	A	A	A	A	A	A	A	A	A	A	A	A	A	A	H	-	A	A	A	A	A	A	A	A	A	A	A	A	A	A	A	A		
RM3431	6	8.75	A	A	A	A	A	A	A	A	A	A	A	A	A	A	A	A	-	H	A	A	A	A	A	A	A	A	A	A	A	A	A	A	A	A		
RM19890	6	11.95	A	A	A	A	A	A	A	A	A	A	A	A	A	A	A	A	A	H	A	A	A	A	A	A	A	A	A	A	A	A	A	A	A	A		1/1 (blue)
RM7193	6	20.26	A	A	H	A	A	A	A	A	A	A	A	A	A	A	A	A	A	H	A	A	A	A	A	A	A	A	A	A	A	A	A	A	A	A		
RM5371	6	25.83	H	A	H	A	A	A	A	A	A	A	A	A	A	A	A	A	A	-	A	A	A	A	A	A	A	A	A	A	A	A	A	H	A	A	2/3 (red)	1/3 (red)
RM3509	6	30.97	H	A	H	A	A	A	A	A	A	A	A	A	A	A	A	A	A	A	A	A	A	A	A	A	A	A	A	A	A	A	A	A	A	A		
RM3394	7	0.65	A	A	A	A	A	A	A	A	A	A	A	A	A	A	A	A	A	A	H	A	A	A	A	A	A	A	A	A	A	A	A	A	A	A		
RM1134	7	3.59	A	A	A	A	A	A	A	A	A	A	A	A	A	A	A	A	A	A	H	A	A	A	A	A	A	A	A	A	A	A	A	A	A	A		
RM5672	7	6.38	A	A	A	A	A	A	A	A	A	A	A	A	A	A	A	A	A	A	H	A	A	A	A	A	A	A	A	A	A	A	A	A	A	A		
RM3859	7	8.88	A	A	A	A	A	A	A	A	A	A	A	A	A	A	A	A	A	A	H	H	A	A	A	A	A	A	A	A	A	A	A	A	A	A		
RM3832	7	12.71	A	A	A	A	A	A	A	A	A	A	A	A	A	A	A	A	A	A	H	H	A	A	A	A	A	A	A	A	A	A	A	A	A	A	1/2 (red)	
RM5543	7	15.75	A	A	A	A	A	A	A	A	A	A	A	A	A	A	A	A	A	A	-	H	-	A	A	A	A	A	A	A	A	A	A	A	A	A		
RM6394	7	18.39	A	A	A	A	A	A	A	A	A	A	A	A	A	A	A	A	A	A	A	H	H	A	A	A	A	A	A	A	A	A	A	A	A	A		2/2 (red)
RM3404	7	20.11	A	A	A	A	A	A	A	A	A	A	A	A	A	A	A	A	A	A	A	H	H	A	A	A	A	A	A	A	A	A	A	A	A	A		
RM6885	7	23.01	A	A	A	A	A	A	A	A	A	A	A	A	A	A	A	A	A	A	A	H	H	A	A	A	A	A	A	A	A	A	A	A	A	A		
RM5426	7	25.93	A	A	A	A	A	A	A	A	A	A	-	A	A	A	A	A	A	A	A	-	H	A	A	A	A	A	A	A	A	A	A	A	A	A		
RM7601	7	29.04	A	A	A	A	A	A	A	A	A	A	H	A	A	A	A	A	A	A	A	-	H	A	A	A	A	A	A	A	A	A	A	A	A	A	2/2 (blue)	(blue)
RM5911	8	0.08	A	A	A	A	A	A	A	A	A	A	A	A	A	A	A	A	A	H	A	A	A	H	A	A	A	A	A	A	A	A	A	A	A	A		
RM6999	8	3.99	A	A	A	A	A	A	A	A	A	A	A	A	A	A	A	A	A	A	A	A	A	H	A	A	A	A	A	A	A	A	A	A	A	A		
RM2584	8	7.57	A	A	A	A	A	A	A	A	A	A	A	A	A	A	A	A	A	A	A	A	A	H	H	A	A	A	A	A	A	A	A	A	A	A		
RM3395	8	10.29	A	A	A	A	A	A	A	A	A	A	A	A	A	A	A	A	A	A	A	A	A	H	H	A	A	A	A	A	A	A	A	A	A	A		
RM3153	8	18.99	A	A	A	A	A	A	A	A	A	A	A	A	A	A	A	A	A	A	A	A	A	H	A	A	A	A	A	A	A	A	A	A	A	A		
RM7556	8	22.21	A	A	A	A	A	A	A	A	A	A	A	A	A	A	A	A	A	A	A	A	A	H	A	H	A	A	A	A	A	A	A	A	A	A		
RM3452	8	24.78	A	A	A	A	A	A	A	A	A	A	A	A	A	A	A	A	A	A	A	A	A	-	A	H	A	A	A	A	A	A	A	A	A	A		
RM3840	8	27.93	A	A	A	A	A	A	A	A	A	A	A	A	A	A	A	A	A	A	A	A	A	-	A	H	H	A	A	A	A	A	A	A	A	A	1/2 (red)	1/2 (blue)
RM23668	9	0.65	A	A	A	A	A	A	A	A	A	A	A	A	H	A	A	A	A	A	A	A	A	A	A	A	H	H	A	A	A	A	A	A	A	A		
RM23736	9	2.46	A	A	A	A	A	A	A	A	A	A	A	A	H	A	A	A	A	A	A	A	A	A	A	A	H	H	A	A	A	A	A	A	H	A		
RM5899	9	5.12	A	A	A	A	A	A	A	A	A	A	A	A	H	A	A	A	A	A	A	A	A	A	A	A	H	H	A	A	A	A	A	A	H	A	3/4 (blue)	
RM6920	9	7.01	A	A	A	A	A	A	A	A	A	A	A	A	-	A	A	A	A	A	A	A	A	A	A	A	H	H	A	A	A	A	A	A	-	A		
RM23941	9	7.82	A	A	A	A	A	A	A	A	A	A	A	A	H	A	A	A	A	A	A	A	A	A	A	A	-	-	A	A	A	A	A	A	-	A		
RM1328	9	9.21	A	A	A	A	A	A	A	A	A	A	A	A	-	-	A	A	A	A	A	A	A	A	A	A	H	H	A	A	A	A	A	A	-	A		
RM7390	9	10.47	A	A	A	A	A	A	A	A	A	A	A	A	A	-	A	A	A	A	A	A	A	A	A	A	A	-	A	A	A	A	A	A	H	A		1/1 (red)
RM6475	9	12.84	A	A	A	A	A	A	A	A	A	A	A	A	A	A	A	A	A	A	A	A	A	A	A	A	A	H	A	A	A	A	A	A	A	A		
RM3492	9	16.50	A	A	A	A	A	A	A	A	A	A	A	A	A	A	A	A	A	A	A	A	A	A	A	A	A	H	A	A	A	A	A	A	A	A		
RM5786	9	19.79	A	A	A	A	A	A	A	A	A	A	A	A	A	A	A	A	A	A	A	A	A	A	A	A	A	H	A	A	A	A	A	A	A	A		1/1 (red)
RM2482	9	22.58	A	A	A	A	A	A	A	A	A	A	A	A	A	A	A	A	A	A	A	A	A	A	A	A	A	H	A	A	A	A	A	A	A	A		
RM7492	10	0.05	A	A	A	A	A	A	A	A	A	H	H	A	H	A	A	A	A	A	A	A	A	A	A	A	A	A	H	A	A	A	A	A	A	A		
RM3882	10	2.74	A	A	A	A	A	A	A	A	A	H	H	A	-	A	A	A	A	A	A	A	A	A	A	A	A	A	-	A	A	A	A	A	A	A		
RM25101	10	5.72	A	A	A	A	A	A	A	A	A	-	-	A	A	A	A	A	A	A	A	A	A	A	A	A	A	A	H	A	A	A	A	A	A	A		
RM25209	10	9.50	A	A	A	A	A	A	A	A	A	A	A	A	A	A	A	A	A	A	A	A	A	A	A	A	A	A	H	A	A	A	A	A	A	A		
RM8201	10	13.83	A	A	A	A	A	A	A	A	A	A	A	A	A	A	A	A	A	A	A	A	A	A	A	A	A	A	H	A	A	A	A	A	A	A		
RM5756	10	18.00	A	A	A	A	A	A	A	A	A	A	A	A	A	A	A	A	A	A	A	A	A	A	A	A	A	A	H	A	A	A	A	A	A	A		
RM6673	10	23.08	A	A	A	A	A	A	A	A	A	A	A	A	A	A	A	A	A	A	A	A	A	A	A	A	A	A	H	A	A	A	A	A	A	A	1/1 (blue)	1/1 (blue)
RM7203	11	1.08	A	A	A	A	A	A	A	A	A	A	A	A	A	A	A	A	A	A	-	A	A	A	A	A	A	A	A	H	A	A	A	A	A	A		
RM5918	11	3.03	A	A	A	A	A	A	A	A	A	A	A	A	A	A	A	A	A	A	-	A	A	A	A	A	A	A	A	H	-	A	A	A	A	A		
RM3137	11	6.19	A	A	A	A	A	A	A	A	A	A	A	A	A	A	A	A	A	A	-	A	A	A	A	A	A	A	A	H	H	A	A	A	A	A		
RM5731	11	9.96	A	A	A	A	A	A	A	A	A	A	A	A	A	A	A	A	A	A	A	A	A	A	A	A	A	A	A	A	H	H	A	A	A	A		
RM7226	11	14.51	A	A	A	A	A	A	A	A	A	A	A	A	A	A	A	A	A	A	A	A	A	A	A	A	A	A	A	A	H	H	A	A	A	A		
RM5960	11	17.49	A	A	A	A	A	A	A	A	A	A	A	A	A	A	A	A	A	A	A	A	A	A	A	A	A	A	A	A	H	H	A	A	A	A		
RM6897	11	18.67	A	A	A	A	A	A	A	A	A	A	A	A	A	A	A	A	A	A	A	A	A	A	A	A	A	A	A	A	H	H	A	A	A	A		
RM1341	11	20.14	A	A	A	A	A	A	A	A	A	A	A	A	A	A	A	A	A	A	A	A	A	A	A	A	A	A	A	A	A	H	-	A	A	A		
RM5716	11	23.47	A	A	A	A	A	A	A	A	A	A	A	A	A	A	A	A	A	A	A	A	A	A	A	A	A	A	A	A	A	H	H	A	A	A	2/2 (blue)	1/2 (blue)
RM2191	11	25.13	A	A	A	A	A	A	A	A	A	A	A	A	A	A	A	A	A	A	A	A	A	A	A	A	A	A	A	A	A	A	H	-	A	A		
RM2064	11	27.49	A	A	A	A	A	A	A	A	A	A	A	A	A	A	A	A	A	A	A	A	A	A	A	A	A	A	A	A	A	A	-	H	A	A		
RM6094	11	28.93	A	A	A	A	A	A	A	A	A	A	A	A	A	A	A	A	A	A	A	A	A	A	A	A	A	A	A	A	A	A	A	H	A	A		
RM5367	12	0.17	A	A	A	A	A	A	A	A	A	A	A	A	A	A	A	A	A	A	A	A	A	A	A	A	A	A	A	A	A	A	A	A	H	A		
RM7619	12	4.83	A	A	A	A	A	A	A	A	A	A	A	A	A	A	A	A	A	A	A	A	A	A	A	A	A	A	A	A	A	A	A	A	H	A		
RM3246	12	9.10	A	A	A	A	A	A	A	A	A	A	A	A	A	A	A	A	A	A	A	A	A	A	A	A	A	A	A	A	A	A	A	A	H	A	1/1 (red)	
RM28037	12	13.74	A	A	A	A	A	A	A	A	A	A	A	A	A	A	A	A	A	A	A	A	A	A	A	A	A	A	A	A	A	A	A	A	H	A		
RM28166	12	17.61	A	A	A	A	A	A	A	A	A	A	A	A	A	A	A	A	A	A	A	A	A	A	A	A	A	A	A	A	A	A	A	A	H	A		
RM1246	12	19.12	A	A	A	A	A	A	A	A	A	A	A	A	A	A	A	A	A	A	A	A	A	A	A	A	A	A	A	A	A	A	A	A	H	H		
RM5479	12	24.41	A	A	A	A	A	A	A	A	A	A	A	A	A	A	A	A	A	A	A	H	A	A	A	A	A	A	A	H	A	A	A	A	H	H		
RM1227	12	27.34	A	A	A	A	A	A	A	A	A	A	A	A	A	A	A	A	A	H	A	A	A	A	A	A	A	A	A	H	A	A	A	A	A	H		2/3 (blue)

[a]Y indicates the presence, and N the absence, of a QTL.

[b]A indicates a homozygous Koshihikari allele; H indicates a heterozygous allele.

[c]QTLs with Koshihikari alleles that decrease trait values are shown in red boxes, while those that increase trait values are shown in blue boxes.
The numbers to the right of each box indicate the number of populations in which the QTL was detected, relative to the total number of populations examined.

Supplemental Table 9. Mean grain length and grain width in 36 MUK-BC$_4$F$_2$ populations, along with graphical genotypes of their parental BC$_4$F$_1$ plants.

Line name			15-1803	15-1804	15-1805	15-1806	15-1807	15-1808	15-1809	15-1810	15-1811	15-1812	15-1813	15-1814	15-1815	15-1816	15-1817	15-1818	15-1819	15-1820	15-1821	15-1822	15-1823	15-1824	15-1825	15-1826	15-1827	15-1828	15-1829	15-1830	15-1831	15-1832	15-1833	15-1834	15-1835	15-1836	15-1837	15-1838	Positions of QTL[c] L	Positions of QTL[c] W
Grain length	Mean±SD (mm)		7.27±0.32	6.92±0.31	7.10±0.30	7.17±0.32	7.25±0.31	7.09±0.29	7.14±0.31	7.33±0.34	7.34±0.32	7.09±0.30	7.28±0.33	6.97±0.34	6.92±0.30	7.08±0.30	7.23±0.31	7.27±0.31	7.33±0.31	6.87±0.29	7.08±0.32	6.85±0.31	7.02±0.32	7.21±0.33	7.06±0.30	7.31±0.31	7.05±0.31	7.12±0.31	7.09±0.30	7.02±0.29	7.12±0.31	7.22±0.29	7.09±0.30	6.98±0.30	6.94±0.28	7.29±0.35	7.10±0.33	6.81±0.31		
	QTL detection (Y/N)[a]		Y	Y	Y	Y	Y	Y	Y	Y	Y	Y	Y	N	Y	Y	Y	Y	Y	Y	Y	Y	Y	Y	Y	Y	Y	Y	Y	Y	Y	Y	Y	Y	N	Y	Y	Y		
Grain width	Mean±SD (mm)		3.44±0.16	3.42±0.16	3.40±0.14	3.52±0.15	3.40±0.15	3.34±01.5	3.42±0.15	3.40±0.16	3.45±0.15	3.27±0.14	3.34±0.15	3.30±0.17	3.48±0.14	3.35±0.16	3.26±0.14	3.45±0.15	3.45±0.14	3.34±0.14	3.40±0.16	3.32±0.14	3.40±0.14	3.48±0.15	3.34±0.13	3.30±0.14	3.39±0.15	3.34±0.14	3.41±0.13	3.41±0.14	3.39±0.14	3.47±0.14	3.48±0.14	3.40±0.14	3.51±0.14	3.49±0.15	3.40±0.15	3.34±0.15		
	QTL detection (Y/N)[a]		N	Y	N	Y	N	Y	Y	Y	Y	Y	Y	Y	N	Y	Y	Y	Y	Y	Y	N	Y	Y	Y	Y	Y	Y	N	N	Y	N	Y	Y	N	N	N	Y		
Genotypes[b]																																								
Marker	Chr. No.	Position (Mb)																																						
RM3252	1	0.30	H	-	-	-	-	-	-	-	-	-	-	-	H	-	-	-	-	-	-	-	-	-	-	-	-	-	-	-	-	-	-	-	-	-	-	-		
RM7383	1	3.48	H	-	-	-	-	-	-	-	-	-	-	-	H	-	-	-	-	-	-	-	-	-	-	-	-	-	-	-	-	-	-	-	-	-	-	-		2/2 (red)
RM5302	1	4.15	-	H	-	-	-	-	-	-	-	-	-	-	-	-	-	-	-	-	-	-	-	-	-	-	-	-	-	-	-	-	-	-	-	-	-	-		
RM5443	1	6.23	H	H	A	H	A	A	A	A	A	A	A	A	H	A	A	A	A	A	A	A	A	A	A	A	A	A	A	A	A	A	A	A	A	A	A	A		
RM8133	1	9.39	A	H	A	A	A	A	A	A	A	A	A	A	A	A	A	A	A	A	A	A	A	A	A	A	A	A	A	A	A	A	A	A	A	A	A	A		
RM8095	1	11.24	A	H	A	A	A	A	A	A	A	A	A	A	A	A	A	A	A	A	A	A	A	A	A	A	A	A	A	A	A	A	A	A	A	A	A	A		
RM5365	1	14.52	A	H	A	A	A	A	A	A	A	A	A	A	A	A	A	A	A	A	A	A	A	A	A	A	A	A	A	A	A	A	A	A	A	A	A	A	1/1 (blue)	
RM6740	1	17.28	A	H	-	A	A	A	A	A	A	A	A	A	A	A	A	A	A	A	A	A	A	A	A	A	A	A	A	A	A	A	A	A	A	A	A	A		
RM5638	1	20.94	A	H	H	A	A	A	A	A	A	A	A	A	A	A	A	A	A	A	A	A	A	A	A	A	A	A	A	A	A	A	A	A	A	A	A	A		1/2 (blue)
RM7124	1	24.39	A	A	H	H	A	A	A	A	H	A	A	A	A	A	A	A	A	A	A	A	A	H	H	A	A	A	A	A	A	A	A	A	H	A	A	A		
RM3143	1	26.82	A	A	-	-	A	H	A	A	-	A	A	A	A	A	A	A	A	A	A	A	A	-	H	A	A	A	A	A	A	A	H	H	H	A	A	A		
RM7566	1	27.77	A	A	H	H	A	A	A	A	H	A	A	A	A	A	A	A	A	A	A	A	A	H	-	A	A	A	A	A	A	A	-	-	-	A	A	A		
RM5914	1	31.51	A	A	H	H	A	A	A	A	H	A	A	A	A	A	A	A	A	A	A	A	A	H	A	A	A	A	A	A	A	A	A	A	A	A	A	A	2/4 (red)	3/4 (red)
RM8137	1	42.93	A	A	A	A	H	A	A	A	A	A	A	A	A	A	A	A	A	A	A	A	A	A	A	A	A	A	A	A	A	A	A	A	A	A	A	A		
RM7562	2	1.88	A	A	A	A	H	A	A	A	A	A	A	A	A	A	A	A	A	A	A	A	A	A	A	A	A	A	A	A	A	A	A	-	A	A	A	A	1/1 (blue)	
RM4702	2	6.22	A	A	A	A	A	H	A	A	A	A	A	A	A	A	A	A	A	A	A	A	A	A	A	A	A	A	A	A	A	A	A	H	A	A	A	A		
RM7288	2	9.03	A	A	A	A	A	H	A	A	A	A	A	A	A	A	A	A	A	A	A	A	A	A	A	A	A	A	A	A	A	A	A	H	A	A	A	A		
RM5619	2	12.01	A	A	A	A	A	H	A	A	A	A	A	A	A	A	A	A	A	A	A	A	A	A	A	A	A	A	A	A	A	A	A	H	A	A	A	A	1/2 (red)	
RM6374	2	15.19	A	A	A	A	A	H	H	A	A	A	A	A	A	A	A	A	A	A	A	A	A	A	-	A	A	A	A	A	A	A	A	H	A	A	A	A		
RM1211	2	18.46	A	A	A	A	A	H	H	A	A	A	A	A	A	A	A	A	A	A	A	A	A	A	A	H	A	A	A	A	A	A	A	H	A	A	A	A		2/3 (blue)
RM5614	2	21.06	A	A	A	A	A	A	H	A	A	A	A	A	A	A	A	A	A	A	A	A	A	A	A	H	A	A	A	A	A	A	A	-	A	A	A	A		
RM3515	2	24.02	A	A	A	A	A	A	H	A	A	A	A	A	A	A	A	A	A	A	A	A	A	A	A	H	A	A	A	A	A	A	A	A	A	A	A	-		
RM6636	2	33.08	H	A	A	A	H	A	A	H	A	A	A	A	A	A	A	A	A	A	A	A	A	A	A	A	A	A	A	A	A	A	A	A	A	A	A	H		
RM6923	2	35.70	H	A	A	A	H	A	A	H	A	A	A	A	A	A	A	A	A	A	A	A	A	A	A	A	A	A	A	A	A	A	A	A	A	A	A	H		1/4 (blue)
RM7332	3	0.41	A	A	A	A	A	A	A	A	H	A	A	A	A	A	A	A	A	A	A	A	A	A	A	A	A	A	H	A	H	A	A	A	A	A	A	A		
RM6829	3	2.88	A	A	A	A	A	A	A	A	H	A	A	A	A	A	A	A	A	A	A	A	A	A	A	A	A	A	A	A	A	A	A	A	A	A	A	A	1/1 (red)	
RM6959	3	14.51	A	A	A	A	A	A	A	A	A	H	A	A	A	A	A	A	A	A	A	A	A	A	A	A	A	A	A	A	A	A	A	A	A	-	A	A	1/1 (blue)	
RM3400	3	17.27	A	A	A	A	A	A	A	A	A	A	H	A	A	A	A	A	A	A	A	A	A	A	A	A	H	A	A	A	A	A	A	A	A	H	A	A	3/3 (red)	
RM7370	3	21.15	A	A	A	A	A	A	A	A	A	A	H	A	A	A	A	A	A	A	A	A	A	A	A	A	A	A	A	A	A	A	A	-	A	H	A	A		
RM3513	3	25.11	A	-	A	A	A	A	A	A	A	A	H	-	A	A	A	A	A	A	A	A	A	A	A	A	A	A	A	A	A	A	A	-	A	H	A	A		
RM2334	3	26.75	A	H	A	A	A	A	A	A	A	A	-	H	A	A	A	A	A	A	A	A	A	A	A	A	A	A	A	A	A	A	A	H	A	-	A	A		
RM1350	3	28.68	A	A	A	A	A	A	A	A	A	A	H	H	A	A	A	A	A	A	A	A	A	A	A	A	A	A	A	A	A	A	A	-	A	A	A	A		
RM6817	3	31.93	A	A	A	A	A	A	A	A	A	A	H	H	A	A	A	A	A	A	A	A	A	A	A	A	A	A	A	A	A	A	A	A	A	A	A	A		
RM1373	3	33.72	A	A	A	A	A	A	A	A	A	A	H	H	A	A	A	A	A	A	A	A	A	A	A	A	A	A	A	A	A	A	A	A	A	A	A	A		2/2 (blue)
RM7324	3	36.35	A	A	A	A	A	A	A	A	A	A	H	-	A	A	A	A	A	A	A	A	A	A	A	A	A	A	A	A	A	A	A	A	A	A	A	A		
RM1867	4	0.92	A	A	A	A	H	A	A	A	A	A	A	A	A	A	A	A	A	A	A	A	A	A	A	A	A	A	A	A	A	A	A	A	A	A	A	A		
RM5414	4	2.03	A	A	A	A	H	A	A	A	A	A	A	A	A	A	A	A	A	A	A	A	A	A	A	A	A	A	A	A	A	A	A	-	A	A	A	A		
RM1305	4	5.66	A	A	A	A	H	A	A	A	A	A	A	A	H	A	A	A	A	A	A	A	A	A	A	A	A	A	A	A	A	A	A	-	A	A	A	A		
RM16606	4	11.57	A	A	A	A	H	A	A	A	A	A	A	A	H	A	A	A	A	A	A	A	A	A	A	A	A	A	A	A	A	A	A	-	A	A	A	A	1/2 (blue)	
RM7279	4	13.77	A	A	A	A	H	A	A	A	A	A	A	A	H	H	A	A	A	A	A	A	A	A	A	A	A	A	A	A	A	A	A	-	A	A	A	A		
RM5586	4	19.90	A	A	A	A	-	A	A	A	A	A	A	A	H	H	A	A	A	-	-	A	A	A	A	A	A	A	A	A	A	A	A	-	A	A	A	A		
RM1359	4	20.03	A	A	A	A	A	A	A	A	A	A	A	A	-	-	A	A	A	H	H	A	A	A	A	A	A	A	A	A	A	A	A	A	A	A	A	A		1/2 (blue)
RM5757	4	21.42	A	A	A	A	A	A	A	A	A	A	A	A	H	H	A	A	A	-	-	A	A	A	A	A	A	A	A	A	A	A	A	A	A	A	A	A	1/2 (red)	
RM3367	4	24.28	A	A	A	A	A	A	A	A	A	A	A	A	A	H	A	A	A	A	A	A	A	A	A	A	A	A	A	A	A	A	A	A	A	A	A	A		
RM2441	4	28.05	A	A	A	A	A	A	A	A	A	A	A	A	A	H	A	A	A	A	A	A	A	A	A	A	-	A	-	A	A	A	A	A	A	A	A	A		
RM6480	4	29.88	A	A	A	A	A	A	A	A	A	A	A	A	A	-	A	A	A	A	A	A	A	A	A	A	H	A	H	A	A	A	A	A	A	A	A	A		
RM3843	4	31.68	A	A	A	A	A	A	A	A	A	A	A	A	A	H	A	A	A	A	A	A	A	A	A	A	-	A	-	A	A	A	A	A	A	A	A	A		
RM5478	4	33.48	A	A	A	A	A	A	A	A	A	A	A	A	A	-	A	A	A	A	A	A	A	A	A	A	A	A	H	A	A	A	A	A	A	A	A	A		
RM3332	4	34.90	A	A	A	A	A	A	A	A	A	A	A	A	A	H	A	A	A	A	A	A	A	A	A	A	A	A	-	A	A	A	A	A	A	A	A	A		
RM1841	4	35.10	A	A	A	A	A	A	A	A	A	A	A	A	A	H	A	A	A	A	A	A	A	A	A	A	A	A	A	A	A	A	A	A	A	A	A	A		
RM2488	5	2.15	A	A	A	A	A	A	A	A	A	A	A	A	A	A	H	A	A	A	A	A	A	A	A	A	A	A	A	A	A	A	A	A	A	A	A	A		
RM3777	5	4.17	A	A	A	A	A	A	A	A	A	H	A	A	A	A	H	A	A	A	A	A	A	A	A	A	A	A	A	A	A	A	A	A	A	A	A	A		
IDR0697	5	5.03	A	A	A	A	A	A	A	A	A	H	A	A	A	A	H	A	A	A	A	A	A	A	A	A	A	A	A	A	A	A	A	A	A	A	A	A	2/2 (red)	2/2 (blue)
RM2422	5	7.02	A	A	A	A	A	A	A	A	A	A	A	A	A	A	-	A	A	A	A	A	A	A	A	A	A	A	A	H	H	A	A	A	A	A	A	A		
RM6229	5	13.61	A	A	A	A	A	A	A	A	A	A	A	A	A	A	H	A	A	A	A	A	A	A	A	A	A	A	A	H	H	A	A	A	A	A	A	A		
RM1237	5	18.02	A	A	A	A	A	A	A	A	A	A	A	A	A	A	H	A	A	A	A	A	A	A	A	A	-	A	A	-	A	A	A	A	A	-	A	A		
RM3800	5	21.45	A	A	A	A	A	A	A	A	A	A	A	A	A	A	H	A	A	A	A	A	A	A	A	A	H	A	A	A	A	A	A	A	A	H	A	A		
RM7452	5	26.99	A	A	A	A	A	A	A	A	A	A	A	A	A	A	A	H	A	A	A	A	A	A	A	A	A	A	A	A	A	A	A	A	A	A	A	A		
RM5907	5	29.61	A	A	A	A	A	A	A	A	A	A	A	A	A	A	A	H	A	A	A	A	A	A	A	A	A	A	A	A	A	A	A	A	A	A	A	A		
RM6273	6	0.13	A	A	A	A	A	A	H	A	A	A	A	A	A	A	A	A	A	A	A	A	A	A	A	A	A	A	A	A	A	A	A	A	A	A	A	A		
RM3805	6	2.85	A	-	A	A	A	A	H	A	A	A	A	A	A	A	A	A	A	A	A	A	A	A	A	A	A	A	A	A	A	A	A	A	A	A	A	-		1/1 (red)
RM8258	6	4.74	A	A	A	A	A	A	H	A	A	A	A	A	A	A	A	A	A	A	A	A	A	A	A	A	A	A	A	A	A	A	A	A	A	A	A	H		
RM4128	6	6.65	A	H	A	A	A	A	-	A	A	A	A	A	A	A	A	-	A	-	A	-	A	A	A	A	A	A	A	A	A	A	A	A	A	A	A	-		
RM3431	6	8.75	A	H	A	A	A	A	A	A	A	A	A	A	A	A	A	H	H	H	A	H	A	A	A	A	A	A	A	A	A	A	A	A	A	A	A	A	5/5 (blue)	1/5 (red)
RM3183	6	12.45	A	-	A	A	A	A	A	A	A	A	A	A	A	A	A	H	H	H	A	H	A	A	A	A	A	A	A	A	A	A	A	A	A	A	A	A		3/4 (blue)
RM5087	6	16.68	A	A	A	A	A	A	A	A	A	A	A	A	A	A	A	H	H	H	A	H	A	A	A	A	A	A	A	A	A	A	A	A	A	A	A	A		
RM7193	6	20.26	A	A	A	A	A	A	A	A	A	A	A	A	A	A	A	H	H	H	A	H	A	A	A	A	A	A	A	A	A	A	A	A	A	A	A	A		
RM7434	6	23.94	A	A	A	A	A	A	A	A	A	A	A	A	A	A	A	A	H	A	A	A	A	A	A	A	A	A	A	A	A	A	A	A	A	A	A	A		
RM6395	6	26.00	A	A	A	A	A	A	A	A	A	A	A	A	A	A	A	A	H	A	A	A	A	A	A	A	A	A	A	A	A	A	A	A	A	A	A	A	1/1 (red)	
RM7243	6	27.57	A	A	A	A	A	A	A	A	A	A	A	A	A	A	A	H	-	A	A	A	A	A	A	A	A	A	A	A	A	A	A	A	A	A	A	A		
RM3509	6	30.97	A	A	A	A	A	A	A	A	A	A	A	A	A	A	A	H	A	A	A	A	A	A	A	A	A	A	A	A	A	A	A	A	A	A	A	A		
RM3394	7	0.65	A	A	A	A	A	A	A	A	A	A	A	A	A	A	A	A	A	H	A	A	A	A	A	A	A	A	A	A	A	A	H	A	A	A	A	A		
RM5752	7	2.57	A	A	A	A	A	A	A	A	A	A	A	A	A	A	A	A	A	-	A	A	A	A	A	A	A	A	A	A	A	A	H	A	A	A	A	A		
RM1134	7	3.59	A	A	A	A	A	A	A	A	A	A	A	A	A	A	A	A	A	H	A	A	A	A	A	A	A	A	A	A	A	A	-	A	A	A	A	A		
RM1253	7	6.97	A	A	A	A	-	A	A	A	A	A	A	A	A	A	A	A	A	H	-	A	A	A	A	A	A	A	A	A	A	A	A	A	A	A	A	A		
RM3635	7	11.09	A	A	A	A	A	H	A	A	A	A	A	A	A	A	A	A	A	H	H	A	A	A	A	A	A	A	A	A	A	A	A	A	A	A	A	A		
RM7273	7	13.43	A	A	A	A	A	-	A	A	A	A	A	A	A	A	A	A	A	H	-	A	A	A	A	A	A	A	A	A	A	A	A	A	A	A	A	A		
RM3755	7	14.73	A	A	A	A	A	H	A	A	A	A	A	A	A	A	A	A	A	-	H	-	A	A	A	A	A	A	A	A	A	A	A	A	A	A	A	A		
RM6394	7	18.39	A	A	A	A	A	H	A	A	A	A	A	A	A	A	A	A	A	A	H	H	A	A	A	A	A	A	A	A	A	A	A	A	A	A	A	A		
RM6152	7	22.13	A	A	A	A	A	A	A	A	A	A	A	A	A	A	A	A	A	A	H	H	A	A	A	A	A	A	A	A	A	A	A	A	A	A	A	A	1/2 (red)	
RM1330	7	25.66	A	A	A	A	A	A	A	A	A	A	A	A	A	A	A	A	A	A	-	H	-	A	A	A	A	A	A	A	A	A	A	A	A	A	A	A		
RM5455	7	26.46	A	A	A	A	A	A	A	A	A	A	A	A	A	A	A	A	A	A	-	-	H	A	A	A	A	A	A	A	A	A	A	A	A	A	A	A		
RM6063	7	27.61	A	A	A	A	A	A	A	A	A	A	A	A	A	A	A	A	A	A	A	H	H	A	A	A	A	A	A	A	A	A	A	A	A	A	A	A	2/2 (blue)	1/2 (blue)
RM7601	7	29.04	A	A	A	A	A	A	A	A	A	A	A	A	A	A	A	A	A	A	A	H	H	A	A	A	A	A	A	A	A	A	A	A	A	A	A	A		
IDR1082	7	29.63	A	A	A	A	A	A	A	A	A	A	A	A	A	A	A	A	A	A	-	H	A	A	A	A	A	A	A	A	A	A	A	A	A	A	A	A		
RM6369	8	0.13	A	A	A	A	A	A	A	A	A	A	A	A	A	A	A	H	A	A	A	A	H	A	A	A	A	A	A	H	A	A	A	A	A	-	A	A		
RM5434	8	3.13	A	A	A	A	A	A	A	A	A	A	A	A	A	A	A	A	A	A	A	A	H	-	A	A	A	A	A	A	A	A	A	A	A	H	-	A		
RM6999	8	3.99	-	-	-	-	-	-	-	-	-	-	-	-	-	-	-	-	-	-	-	-	H	-	-	-	-	-	-	-	-	-	-	-	-	-	-	-		
RM3507	8	6.16	A	A	A	H	A	A	A	A	A	A	A	A	A	A	A	A	A	A	A	A	H	H	A	A	A	A	A	A	A	A	A	A	A	H	H	A		
RM8019	8	7.49	-	-	-	-	-	-	-	-	-	-	-	-	-	-	-	-	-	-	-	-	-	-	-	-	-	-	-	-	-	-	-	-	-	-	H	-	1/1 (blue)	
RM6027	8	9.11	A	A	A	H	A	A	A	A	A	A	A	A	A	A	A	A	A	A	A	A	H	H	A	A	A	A	A	A	A	A	A	A	A	-	H	-		
RM6032	8	11.90	A	A	A	H	A	A	A	A	A	A	A	A	A	A	A	A	A	A	A	A	H	H	A	A	A	A	A	A	A	A	A	A	A	H	H	A		
RM6990	8	14.36	A	A	A	H	A	A	A	A	A	A	A	A	A	A	A	A	A	A	A	A	-	-	-	A	A	A	A	A	A	A	A	A	A	H	H	A		
RM5767	8	18.82	A	A	A	A	A	A	A	A	A	A	A	A	A	A	A	A	A	A	A	A	H	-	H	A	A	A	A	A	A	A	A	A	A	-	-	A		
RM3491	8	19.62	A	A	A	A	A	A	A	A	A	A	A	A	A	A	A	A	A	A	A	A	A	H	-	A	A	A	A	A	A	A	A	A	A	-	-	A		
RM3634	8	21.58	A	A	A	A	A	A	A	A	A	A	A	A	A	A	A	A	A	A	A	A	A	-	H	A	A	A	A	A	A	A	A	A	A	A	A	A	1/1 (red)	
RM3452	8	24.78	A	A	A	A	A	A	A	A	A	A	A	A	A	A	A	A	A	A	A	A	A	A	H	A	-	A	A	A	A	A	A	A	A	A	A	A		
RM4997	8	28.24	A	A	A	A	A	A	A	A	A	A	A	A	A	A	A	A	A	A	A	A	A	A	H	H	A	H	A	A	A	A	A	A	A	A	A	A	1/3 (red)	3/3 (blue)
RM8219	9	1.55	A	A	A	A	A	A	H	A	A	A	A	A	A	A	A	A	A	A	A	A	A	A	A	H	A	A	A	A	A	A	A	A	A	A	A	A	1/2 (red)	1/2 (red)
RM5899	9	5.12	A	A	A	A	A	A	H	A	A	A	A	A	A	A	A	A	A	A	A	A	A	A	A	H	A	A	A	A	A	A	A	A	A	A	A	A		
RM6920	9	7.01	A	A	A	A	A	A	H	A	A	A	A	A	A	A	A	A	A	A	A	A	A	A	A	-	A	A	A	A	A	A	A	A	A	A	A	A		
RM1328	9	9.21	A	A	A	A	A	A	-	A	A	A	-	A	A	A	A	A	A	A	A	A	A	A	A	H	A	-	A	A	A	A	A	A	A	A	A	A	1/1 (red)	
RM1896	9	11.77	A	A	A	A	A	A	A	A	A	A	H	A	A	A	A	A	A	A	A	A	A	A	A	H	A	H	A	A	A	A	A	A	A	A	A	A		
RM5652	9	14.79	A	A	A	A	A	A	A	A	A	A	A	A	A	A	A	A	A	A	A	A	A	A	A	H	H	H	A	A	A	A	A	A	A	A	A	A		
RM3533	9	17.89	A	A	A	A	A	A	A	A	A	A	A	A	A	A	A	A	A	A	A	A	A	A	A	A	H	H	A	A	A	A	A	A	A	A	A	A	2/2 (blue)	1/2 (blue)
RM3787	9	20.04	A	A	A	A	A	A	A	A	A	A	A	A	A	A	A	A	A	A	A	A	A	A	A	A	A	H	A	A	A	A	A	A	A	A	A	A		1/1 (red)
RM2482	9	22.58	-	-	-	-	-	-	-	-	-	-	-	-	-	-	-	-	-	-	-	-	-	-	-	-	-	H	-	-	-	-	-	-	-	-	-	-		
RM6364	10	0.07	A	A	A	A	A	A	A	A	A	A	A	A	A	A	A	A	H	A	A	A	A	A	A	A	A	A	H	A	A	A	A	A	A	A	A	A		
RM3882	10	2.74	A	A	A	A	A	A	A	A	A	A	A	A	A	A	A	A	A	A	A	A	A	A	A	A	A	A	H	A	H	A	A	A	A	A	A	A	1/2 (blue)	
RM7217	10	4.63	A	A	A	A	A	A	A	A	A	-	A	A	A	A	A	A	A	-	A	A	A	A	A	A	A	A	-	H	-	A	A	A	A	A	A	A		
RM2504	10	7.60	A	A	A	A	A	A	A	A	A	-	A	A	A	A	A	A	A	-	A	A	A	A	A	A	A	A	H	H	H	A	A	A	A	A	A	A		
RM4455	10	11.74	A	A	A	A	A	A	A	A	A	A	A	A	A	A	A	A	A	A	A	A	A	A	A	A	A	A	H	H	H	A	A	A	A	A	A	A	1/2 (red)	
RM5806	10	14.56	A	A	A	A	-	A	A	-	A	A	A	A	A	A	A	A	A	A	A	A	-	A	A	A	A	A	-	-	H	-	A	A	A	A	A	A		
RM5620	10	17.47	A	A	A	A	H	A	A	H	A	A	A	A	A	A	A	A	A	A	A	A	H	A	A	A	A	A	A	A	H	H	A	A	A	A	A	A	1/5 (red)	1/5 (blue)
RM4477	10	21.17	A	A	A	A	A	A	A	A	A	A	A	A	A	A	A	A	A	A	A	A	A	A	A	A	A	A	A	A	H	A	A	A	A	A	A	A		
RM6673	10	23.08	A	A	A	A	A	A	A	A	A	A	A	A	A	A	A	A	A	A	A	A	A	A	A	A	A	A	A	A	H	A	A	A	A	A	A	A		
RM3863	11	0.90	H	A	A	A	H	A	A	H	A	A	A	A	A	A	A	A	A	A	A	A	A	A	A	A	A	A	A	A	A	H	H	A	A	A	A	A		
RM5599	11	3.83	H	A	A	A	H	A	A	H	A	A	A	A	A	A	A	H	A	A	A	A	A	A	A	A	A	A	A	A	A	H	H	-	A	A	A	A	3/6 (red)	1/6 (red)
RM5128	11	5.48	H	A	A	A	-	A	A	H	A	A	A	A	A	A	A	H	A	A	A	A	A	A	A	A	A	A	A	A	A	H	H	-	A	A	A	A		
RM3701	11	8.11	H	A	A	A	A	A	A	H	A	A	A	A	A	A	A	H	A	A	A	A	A	A	A	A	A	A	A	A	A	H	H	A	A	A	A	A		
RM5857	11	11.86	-	A	A	A	A	A	A	-	A	A	A	A	A	A	A	-	A	A	A	A	A	A	A	A	A	A	A	A	A	-	H	A	A	A	A	A		
RM5824	11	14.53	A	A	A	A	A	A	A	A	A	A	A	A	A	A	A	A	A	A	A	A	A	A	A	A	A	A	A	A	A	A	H	A	A	A	A	A		
RM5960	11	17.49	A	A	A	A	A	A	A	A	A	A	A	A	-	A	A	A	A	A	A	A	A	A	A	A	A	A	A	A	A	A	H	H	A	A	A	A		
RM5349	11	19.65	A	A	A	A	A	A	A	A	A	A	A	A	H	A	A	A	A	A	A	A	A	A	A	A	A	A	A	A	A	A	-	-	A	A	A	A	1/1 (blue)	
RM1341	11	20.14	A	A	A	A	A	A	A	A	A	A	A	A	-	A	A	A	A	A	A	A	A	A	A	A	A	A	A	A	A	A	H	H	A	A	A	A		1/2 (red)
RM5997	11	23.18	A	A	A	A	A	A	A	-	A	A	A	A	H	A	-	A	A	A	A	A	A	A	A	A	A	A	A	A	A	A	-	-	A	A	A	A		
RM6499	11	24.08	A	A	A	A	A	A	A	A	A	A	A	A	-	A	A	A	A	A	A	A	A	A	A	A	A	A	A	A	A	A	H	H	A	A	A	A	1/2 (blue)	
RM5926	11	28.96	A	A	A	A	A	A	A	A	A	A	A	A	A	A	A	A	A	A	A	A	A	A	A	A	A	A	A	A	A	A	H	H	A	A	A	A		
RM1080	12	0.91	A	A	A	A	A	A	A	A	A	A	A	A	A	A	A	A	A	A	A	A	A	A	A	A	A	A	A	A	A	A	A	A	H	H	A	A		
RM3747	12	2.31	A	A	A	A	A	A	A	A	A	A	A	A	A	A	A	A	A	A	A	A	A	A	A	A	A	A	A	A	A	A	A	A	H	A	A	A		
RM6998	12	4.75	A	A	A	A	A	A	A	A	A	A	A	A	A	A	A	A	A	A	A	A	A	A	A	A	A	A	A	A	A	A	A	A	A	H	H	A		
RM2935	12	7.43	A	A	A	A	A	A	A	A	A	A	A	A	A	A	A	A	A	A	A	A	A	A	A	A	A	A	A	A	A	A	A	A	A	H	H	A		
RM6973	12	10.08	A	A	A	A	A	A	A	A	A	A	A	A	A	A	A	A	A	A	A	A	A	A	A	A	A	A	A	A	A	A	A	A	A	H	H	A		
RM7344	12	17.95	A	A	A	A	A	A	A	A	A	A	A	A	A	A	A	A	A	A	A	A	A	A	A	A	A	A	A	A	A	A	A	A	A	A	H	A		
RM28305	12	19.96	A	A	A	A	A	A	A	A	A	A	A	A	A	A	A	A	A	A	A	A	A	A	A	A	A	A	A	A	A	A	A	A	A	A	H	H	1/2 (blue)	
RM5479	12	24.41	A	A	A	A	A	A	A	A	A	A	A	H	H	A	A	A	A	A	A	A	A	A	A	A	A	A	A	A	A	A	A	A	A	A	A	H		3/3 (blue)
RM2197	12	27.38	A	A	H	A	A	A	A	A	H	A	A	A	A	A	A	A	A	A	H	H	A	A	A	A	A	A	A	A	A	A	A	H	A	A	A	H		

[a]Y indicates the presence, and N the absence, of a QTL.

[b]A indicates a homozygous Koshihikari allele; H indicates a heterozygous allele.

[c]QTLs with Koshihikari alleles that decrease trait values are shown in red boxes, while those that increase trait values are shown in blue boxes.
The numbers to the right of each box indicate the number of populations in which the QTL was detected, relative to the total number of populations examined.

Supplemental Table 10. Mean grain length and grain width in 36 BAK-BC_4F_2 populations, along with graphical genotypes of their parental BC_4F_1 plants.

Line name			15-1843	15-1844	15-1845	15-1847	15-1848	15-1849	15-1850	15-1851	15-1852	15-1853	15-1854	15-1855	15-1856	15-1857	15-1858	15-1859	15-1860	15-1861	15-1862	15-1863	15-1864	15-1865	15-1866	15-1867	15-1868	15-1869	15-1870	15-1871	15-1872	15-1873	15-1874	15-1875	15-1876	15-1877	15-1878	15-1879	Positions of QTL[c] L	Positions of QTL[c] W
Grain length	Mean±SD (mm)		7.08±0.30	6.96±0.32	7.07±0.31	7.08±0.30	6.83±0.29	6.93±0.31	7.11±0.34	7.08±0.31	6.92±0.30	6.97±0.31	7.24±0.32	7.22±0.32	6.91±0.30	7.03±0.30	6.91±0.31	6.81±0.30	7.05±0.29	6.97±0.30	6.89±0.29	6.95±0.30	6.78±0.29	6.94±0.29	6.89±0.29	7.21±0.33	7.11±0.29	7.05±0.31	6.97±0.29	7.04±0.29	7.10±0.29	7.15±0.31	6.98±0.29	7.13±0.30	6.92±0.30	7.36±0.33	7.06±0.34	6.94±0.29		
	QTL detection (Y/N)[a]		N	Y	Y	Y	Y	Y	N	N	N	N	Y	Y	N	Y	Y	Y	Y	Y	N	Y	Y	N	Y	Y	Y	N	N	Y	Y	N	N	Y	Y	Y	N	Y		
Grain width	Mean±SD (mm)		3.28±0.18	3.26±0.18	3.32±0.15	3.39±0.15	3.35±0.17	3.25±0.17	3.21±0.20	3.24±0.14	3.43±0.17	3.29±0.14	3.20±0.15	3.23±0.16	3.27±0.19	3.39±0.18	3.34±0.17	3.27±0.17	3.25±0.16	3.42±0.15	3.43±0.16	3.41±0.15	3.26±0.14	3.39±0.17	3.37±0.14	3.30±0.17	3.39±0.15	3.29±0.14	3.41±0.13	3.37±0.14	3.42±0.14	3.31±0.17	3.46±0.15	3.40±0.14	3.35±0.15	3.25±0.16	3.32±0.16	3.41±0.16		
	QTL detection (Y/N)[a]		N	Y	Y	Y	Y	Y	Y	Y	Y	Y	Y	Y	Y	N	Y	Y	Y	Y	Y	Y	Y	Y	Y	N	Y	Y	N	Y	N	N	N	N	Y	Y	Y	N		
Genotypes[b]																																							L	W
Marker	Chr. No.	Position (Mb)																																						
IDR0002	1	0.38	-	-	-	-	-	-	-	-	-	-	-	-	-	-	-	-	-	-	-	-	-	-	-	-	-	-	H	-	-	-	-	-	-	-	-	-		red 1/1
RM7383	1	3.48	-	-	-	-	-	-	-	-	-	-	-	-	-	-	-	-	-	-	-	-	-	-	-	-	-	-	H	-	-	-	-	-	-	-	-	-		
RM8071	1	9.49	H	A	A	A	A	A	A	A	A	A	A	A	A	H	A	A	A	A	A	A	A	A	A	A	A	A	A	A	A	A	A	A	A	A	A	A		
IDR0049	1	12.12	H	H	A	A	A	A	A	A	A	A	A	A	A	H	A	A	H	A	A	A	A	A	A	A	A	A	A	A	A	A	A	A	A	A	A	A		
RM7075	1	15.12	A	H	A	A	A	A	A	A	A	A	A	A	A	A	A	A	H	A	A	A	A	A	A	A	A	A	A	A	A	A	A	A	A	A	A	A		
RM5422	1	18.76	A	H	H	A	A	A	A	A	A	A	A	A	A	A	A	A	H	A	A	A	A	A	A	A	A	A	A	A	A	A	A	A	A	A	A	A		
RM5853	1	22.74	A	H	H	H	A	A	A	A	A	A	A	A	A	A	A	A	-	A	A	A	A	A	A	A	A	A	A	A	A	A	A	A	A	A	A	A		
IDR0110	1	25.51	A	H	H	H	A	A	A	A	A	A	A	A	A	A	A	A	A	A	A	A	A	A	A	A	A	A	A	A	A	A	A	A	A	A	A	A	red 3/4	
RM1244	1	28.69	A	H	H	H	A	A	A	A	A	A	A	A	A	A	A	A	H	A	A	A	A	A	A	A	A	A	A	A	A	A	A	A	A	A	A	A		
RM5914	1	31.51	A	H	H	H	A	A	A	A	A	A	A	A	A	A	A	A	-	A	A	A	A	A	A	A	A	A	A	A	A	A	A	A	A	A	A	A		
IDR0151	1	34.17	A	H	A	H	A	A	A	A	A	A	A	A	A	A	A	A	A	A	A	A	A	A	A	A	A	A	A	A	A	A	A	A	A	A	A	A		blue 2/2
RM5448	1	37.40	A	H	A	H	A	A	A	A	A	A	A	A	A	A	A	A	A	A	A	A	A	A	A	A	A	A	A	A	A	A	A	A	A	A	A	A		
RM1387	1	40.21	A	H	A	H	A	A	A	A	A	A	A	A	A	A	A	A	A	A	A	A	A	A	A	A	A	A	A	A	A	A	A	A	A	A	A	A	blue 2/2	
RM6840	1	43.17	A	H	A	-	A	A	A	A	A	A	A	A	A	A	A	A	A	A	A	A	A	A	A	A	A	A	A	A	A	A	A	A	A	A	A	A		
RM6842	2	0.12	A	A	A	A	H	H	A	A	A	A	A	A	A	A	A	A	A	A	A	A	A	A	A	A	A	A	A	A	A	A	A	A	A	A	A	A		
IDR0197	2	0.71	A	A	A	A	H	H	A	A	A	A	A	A	A	A	A	A	A	A	A	A	A	A	A	A	A	A	A	A	A	A	A	A	A	A	A	A		
RM1075	2	3.83	A	A	A	A	H	H	A	A	A	A	A	A	A	A	A	A	A	A	A	A	A	A	A	A	A	A	A	A	A	A	A	A	A	A	A	A	blue 2/2	blue 1/2
RM5340	2	7.48	A	-	A	A	-	H	A	A	A	A	-	A	A	A	A	A	A	A	A	A	A	A	A	A	A	A	A	A	A	A	A	A	A	A	A	A		
RM1234	2	11.34	A	H	H	A	A	H	A	A	A	A	H	A	A	A	A	A	H	A	A	A	A	H	A	A	A	A	A	A	A	A	A	A	A	A	A	A		
RM6374	2	15.19	A	H	H	A	A	H	A	A	A	A	-	A	A	A	A	A	H	A	A	A	A	H	A	A	A	A	A	A	A	A	A	A	A	A	A	A		
RM1211	2	18.46	A	H	H	A	A	H	A	A	A	A	A	A	H	A	A	A	H	A	A	A	A	H	A	A	A	A	A	A	A	A	A	A	A	A	A	A		blue 6/6
RM5614	2	21.06	A	H	H	A	A	H	A	A	A	A	A	A	H	A	A	A	H	A	A	A	A	H	A	A	A	A	A	A	A	A	A	A	A	A	A	A		
RM6379	2	23.57	A	H	H	A	A	H	H	A	A	A	A	A	H	A	A	A	H	A	A	A	A	H	A	A	A	A	A	A	A	A	A	A	A	A	A	A		
RM3793	2	26.84	A	-	-	A	A	-	H	A	A	A	A	A	-	A	A	A	-	A	A	A	A	-	A	A	A	A	A	A	A	A	A	A	A	A	A	A		
RM3421	2	29.92	A	A	A	A	A	A	H	A	A	A	A	A	A	A	A	A	A	A	A	-	A	A	A	A	A	A	A	A	A	A	A	A	A	A	A	A		
IDR0343	2	33.60	A	A	A	A	A	A	H	A	A	A	A	A	A	A	A	A	A	A	A	H	A	A	A	A	A	A	A	A	A	A	A	A	A	A	A	A		
IDR0351	2	35.93	A	A	A	A	A	A	H	A	A	A	A	A	A	A	A	A	A	A	A	H	A	A	A	A	A	A	A	A	A	A	A	A	A	A	A	A		
IDR0352	3	0.12	A	A	A	A	A	A	-	A	A	A	A	A	H	A	A	A	H	A	A	A	A	A	A	A	A	A	A	A	A	H	A	A	A	A	A	A		
RM3807	3	3.53	A	A	A	A	A	A	H	A	A	A	A	A	H	A	A	A	H	A	A	A	A	A	A	A	A	A	A	A	A	H	A	A	A	A	A	A		
RM3029	3	6.71	A	A	A	A	A	A	H	A	A	A	A	A	A	A	A	A	A	A	A	A	A	A	A	A	A	A	A	A	A	H	A	A	A	H	A	A		blue 1/2
RM4321	3	10.17	A	A	A	A	A	A	H	A	A	A	A	A	A	A	A	A	A	A	A	A	A	A	A	A	A	A	A	A	A	H	A	A	A	H	A	A		
RM6929	3	12.93	A	A	A	-	A	A	H	-	A	A	A	A	A	-	A	A	A	A	A	A	A	A	A	A	-	-	A	A	A	-	A	A	A	-	A	A		
RM6959	3	14.51	A	A	A	H	A	A	H	H	A	A	A	A	A	H	A	A	A	A	A	A	A	A	A	A	H	H	A	A	A	A	A	A	A	A	A	A		
RM1334	3	17.42	A	A	A	A	A	A	-	H	A	A	A	A	A	A	A	A	A	A	A	A	A	A	A	A	A	-	A	A	A	A	A	A	A	A	A	A		
IDR0429	3	18.07	A	A	A	A	A	A	H	H	A	A	A	A	A	A	A	A	A	A	A	A	A	A	A	A	H	A	A	A	A	A	A	A	A	A	A	A		
RM5488	3	21.39	A	A	A	A	A	A	H	H	A	A	A	A	A	A	A	A	A	A	A	A	A	A	A	A	H	H	A	A	A	A	A	A	A	A	A	A		blue 2/4
RM3513	3	25.11	A	A	A	A	A	A	-	H	A	A	A	A	A	A	A	A	A	A	A	A	A	A	A	A	H	-	A	A	A	A	A	A	A	H	A	A	red 1/3	
RM1292	3	27.33	A	A	A	A	A	A	A	H	A	A	A	A	A	A	A	A	A	A	A	A	A	A	A	A	A	A	A	A	A	A	A	A	A	H	H	A		
IDR0489	3	29.99	A	A	A	A	A	A	A	H	A	-	A	A	A	A	A	A	A	A	A	A	A	A	A	-	A	A	A	A	A	A	A	A	A	H	H	A		
RM6970	3	32.44	A	A	A	A	A	A	A	H	A	H	A	A	A	A	A	A	A	A	A	A	A	A	A	-	A	A	A	A	A	A	A	A	A	H	H	A		blue 4/4
RM7389	3	36.16	A	A	A	A	A	A	A	A	A	H	A	A	A	A	A	A	A	A	A	A	A	A	A	-	A	A	A	A	A	A	A	H	A	A	-	A	red 1/2	
IDR0518	4	1.01	A	A	A	A	A	A	A	A	H	A	A	A	A	A	A	A	A	A	A	A	A	A	A	A	A	A	A	A	A	A	A	A	A	A	A	A		
RM7200	4	4.04	A	A	A	A	A	A	A	A	H	A	A	A	A	A	A	A	A	A	A	A	A	A	A	A	A	A	A	A	A	A	A	A	A	A	A	A		
RM7472	4	7.10	A	-	A	A	A	A	A	A	H	A	A	A	A	A	A	A	A	A	A	A	A	A	A	A	A	A	A	A	A	A	A	A	A	A	A	A		
IDR0579	4	13.62	A	H	A	A	A	A	A	A	H	A	A	A	A	A	A	A	A	A	A	A	A	A	A	A	A	A	A	A	A	A	A	A	A	A	A	A		
IDR0596	4	16.34	A	H	A	A	A	A	A	A	-	A	A	A	A	A	A	A	A	A	A	A	A	A	A	A	A	A	A	A	A	A	A	A	A	A	A	-		
IDR0612	4	19.48	A	H	A	A	A	A	A	A	A	A	A	A	A	A	A	A	A	A	A	A	A	A	A	A	A	A	A	A	A	A	A	A	A	A	A	-		
RM6629	4	31.74	A	-	A	A	A	A	A	A	A	H	A	A	A	A	A	-	A	A	A	A	A	A	A	A	A	H	A	A	A	A	A	A	A	A	A	H		
RM1272	4	35.30	A	A	A	A	A	A	A	A	A	H	A	A	A	A	A	A	A	A	A	A	A	A	A	A	A	-	A	A	A	A	A	A	A	A	A	-		
RM3529	5	0.11	A	A	A	A	A	A	A	A	A	H	H	A	A	A	A	A	A	A	A	A	A	A	A	-	A	A	A	A	A	A	A	A	A	A	A	A		
IDR0688	5	3.31	A	A	A	A	A	A	A	A	A	H	H	-	A	A	A	A	A	A	A	A	A	A	A	A	A	A	A	A	A	A	A	A	A	A	A	A		
IDR1751	5	3.50	A	A	A	A	A	A	A	A	A	H	H	A	A	A	A	A	A	A	A	A	A	A	A	A	A	A	A	A	A	A	A	A	A	A	A	A		
IDR1752	5	5.45	A	A	A	A	A	A	A	A	A	A	H	H	A	A	A	A	A	A	A	A	A	A	A	A	A	A	A	A	A	A	A	A	A	A	A	A	red 2/2	blue 2/2
RM2422	5	7.02	A	A	A	A	A	A	A	A	A	A	H	H	A	A	A	A	A	A	A	A	A	A	A	A	A	A	A	A	A	A	A	A	A	A	A	A		
RM5844	5	9.15	A	A	A	A	A	A	A	A	A	A	H	H	A	A	A	A	A	A	A	A	A	A	A	A	A	A	A	A	A	A	A	A	A	A	A	A		
RM2676	5	13.54	A	A	A	A	A	A	A	A	A	A	H	H	A	A	A	A	A	A	A	A	A	A	A	A	A	A	A	A	A	A	A	A	A	A	A	A		
RM3838	5	16.55	A	A	A	A	A	A	A	A	A	A	H	H	A	A	A	A	A	A	A	A	A	A	A	A	A	A	A	A	A	A	A	A	A	A	A	A	red 2/2	
RM5329	5	20.09	A	A	A	A	A	A	A	A	A	A	H	H	A	A	A	A	-	A	A	A	A	A	A	A	A	A	A	A	A	A	A	A	A	A	A	A		
RM3476	5	23.91	A	A	A	A	A	A	A	A	A	A	H	-	H	H	A	A	H	A	A	A	A	A	A	A	A	A	A	A	A	A	A	A	A	A	A	A		
IDR0801	5	27.49	A	A	A	H	A	A	A	-	A	A	H	H	H	H	A	A	A	A	A	A	A	A	A	A	A	A	A	A	A	A	A	A	A	A	A	A		blue 1/5
RM5907	5	29.61	A	A	A	H	A	A	A	A	A	A	A	A	H	H	A	A	A	A	A	A	A	A	A	A	A	A	A	A	A	A	A	A	A	A	A	A	red 1/3	
RM7158	6	0.22	A	H	A	A	H	A	A	A	A	A	H	H	A	A	A	A	A	A	A	A	A	A	A	A	A	A	A	A	A	A	A	A	A	A	A	H	blue 3/5	
RM5199	6	2.27	A	H	A	A	-	A	A	A	A	A	-	-	A	A	A	H	A	A	A	A	A	A	A	A	A	A	A	A	A	A	A	A	A	A	A	-		
RM5754	6	5.23	A	H	A	A	-	A	A	A	A	A	H	H	A	A	H	H	A	A	A	A	A	A	A	A	A	H	A	A	A	A	A	A	A	A	A	H		
RM5963	6	8.82	A	A	A	A	A	A	A	A	A	A	-	A	A	A	H	H	A	A	-	A	H	A	A	A	A	A	A	A	A	A	A	A	H	A	A	A	blue 4/4	blue 4/4
IDR0854	6	11.35	A	A	A	A	A	A	A	A	A	A	A	A	-	A	H	H	A	A	-	A	H	A	A	A	A	A	A	A	A	A	A	A	-	A	A	A		
RM1161	6	13.75	A	A	A	A	A	A	A	A	A	A	A	A	A	A	H	H	-	A	A	A	-	A	A	A	A	A	A	A	A	A	A	A	A	A	A	A		
RM5087	6	16.68	A	A	A	A	A	A	A	A	A	A	A	A	A	A	H	H	H	A	A	A	A	A	A	A	A	A	A	A	A	A	A	A	A	A	A	A		blue 2/3
RM20185	6	20.32	A	A	A	A	A	A	A	A	A	A	A	A	A	A	-	A	H	A	A	A	A	A	A	A	A	A	A	A	A	A	A	A	A	A	A	A		
IDR0922	6	24.63	H	A	A	A	A	A	A	A	A	A	A	A	A	A	A	A	H	H	A	H	A	A	A	A	A	A	A	A	A	A	A	A	A	A	A	A	red 2/3	
IDR0925	6	25.42	-	-	-	-	-	-	-	-	-	-	-	-	-	-	-	-	H	-	-	-	-	-	-	-	-	-	-	-	-	-	-	-	-	-	-	-		
RM1370	6	28.53	-	A	A	A	A	A	A	A	H	A	A	A	A	A	A	A	A	H	A	H	A	A	A	A	A	A	A	A	A	A	A	A	A	A	A	A		red 2/3
RM5753	6	30.97	A	A	A	A	A	A	A	A	H	A	A	A	A	A	A	A	A	-	A	-	A	A	A	A	A	A	A	A	A	A	A	A	A	A	A	A		
IDR0950	7	0.36	A	A	A	A	A	A	A	A	A	A	A	A	A	A	A	A	A	A	H	A	A	A	A	A	A	A	A	A	A	A	A	A	A	A	A	A		
IDR0964	7	3.37	A	A	A	A	A	A	A	A	A	A	A	A	A	A	A	A	A	A	H	A	A	A	A	A	A	A	A	A	A	A	A	A	A	A	A	A		
RM5672	7	6.38	A	A	A	A	A	A	A	A	A	A	A	A	A	A	A	A	A	A	H	H	A	A	A	A	A	A	A	A	A	A	A	A	A	A	A	A		
IDR0991	7	9.83	-	A	A	A	A	A	A	A	A	A	A	A	A	A	A	A	A	A	H	H	A	A	A	A	A	A	A	A	A	A	A	A	A	A	A	A		red 1/2
RM3832	7	12.71	-	A	A	A	A	A	A	A	A	A	A	A	A	A	A	A	A	A	H	-	A	A	A	A	A	A	A	A	A	A	A	A	A	A	A	A		
RM5543	7	15.75	-	A	A	A	A	A	A	A	A	A	A	A	A	A	A	A	A	A	H	H	A	A	A	A	A	A	A	A	A	A	A	A	A	A	A	A		
RM7087	7	19.36	H	A	A	A	A	A	A	A	A	A	A	A	A	A	A	A	A	A	A	H	H	A	A	A	A	A	A	A	A	A	A	A	A	A	A	A	red 1/2	red 1/2
IDR1050	7	22.05	A	A	A	A	A	A	A	A	A	A	A	A	A	A	A	A	A	A	A	A	H	A	-	A	A	A	A	A	A	A	A	A	A	A	A	A		
RM6326	7	24.20	A	A	A	A	A	A	A	A	A	A	A	A	A	A	A	A	A	A	A	A	H	A	H	A	A	A	A	A	A	A	A	A	A	A	A	A		
RM5455	7	26.46	A	A	A	A	A	A	-	A	A	A	A	A	A	A	A	A	A	A	A	A	H	A	H	A	A	A	A	A	A	A	A	A	A	A	A	A	blue 2/2	blue 2/2
IDR1082	7	29.63	A	A	A	A	A	A	H	A	A	A	A	A	A	A	A	A	A	A	A	A	H	A	A	A	-	A	A	A	A	A	A	A	A	A	A	A		
RM5911	8	0.08	A	A	A	A	A	A	A	A	A	A	A	A	A	A	H	A	A	A	A	A	A	A	A	A	A	A	A	A	A	A	A	A	A	A	A	A		
RM6999	8	3.99	A	A	A	A	A	A	A	A	A	A	A	A	A	A	H	A	A	A	A	A	A	A	A	A	A	-	A	A	A	A	A	A	A	A	A	A		
IDR1130	8	10.36	A	A	A	A	A	A	A	A	A	A	A	A	A	A	H	A	A	A	A	A	A	H	A	A	A	H	A	A	A	A	A	A	A	A	A	A		
IDR1151	8	14.05	A	A	A	A	A	A	A	A	A	A	A	A	A	A	H	A	A	A	A	A	A	H	A	A	A	H	A	A	A	A	A	A	A	A	A	A		
IDR1170	8	17.82	A	A	A	A	A	A	A	A	A	A	A	A	A	A	A	A	A	A	A	A	A	H	A	A	A	-	A	A	A	A	A	A	A	A	A	A		red 1/1
RM5887	8	20.98	A	A	A	A	A	A	A	-	A	A	A	A	A	A	A	A	A	A	A	A	A	H	A	A	A	A	A	A	A	A	A	A	A	A	A	A		
RM5891	8	24.63	A	A	A	A	A	A	A	H	A	A	A	A	A	A	A	A	A	A	A	A	A	H	H	A	A	A	A	A	A	A	A	A	A	A	A	A		blue 2/3
IDR1203	8	26.40	-	-	-	-	-	-	-	-	-	-	-	-	-	-	-	-	-	-	-	-	-	H	-	-	-	-	-	-	-	-	-	-	-	-	-	-		
RM4997	8	28.24	A	A	A	A	-	-	A	H	A	A	-	A	A	A	A	A	A	A	A	A	A	H	A	A	A	A	A	A	A	A	A	A	A	A	A	A		
IDR1211	9	0.00	-	-	-	-	-	-	-	-	-	-	-	-	-	-	-	-	-	-	-	-	-	-	A	-	-	-	-	-	-	-	-	-	-	-	-	-		
IDR1224	9	2.56	A	A	A	A	A	A	A	A	A	A	A	A	A	A	A	A	A	A	A	A	A	A	H	A	A	A	A	A	A	A	A	A	A	A	A	A		
IDR1242	9	5.77	A	A	A	A	A	A	A	A	A	A	A	A	A	A	A	A	A	A	A	A	A	A	H	A	A	A	A	A	A	A	A	A	A	A	A	A		
IDR1261	9	9.61	A	A	A	A	A	H	A	A	A	A	A	A	A	A	A	A	A	A	A	A	A	A	H	H	A	A	A	A	A	A	A	A	A	A	A	A		
IDR1277	9	13.10	A	A	A	A	A	H	A	A	A	A	A	A	A	A	A	A	A	A	A	A	A	A	A	H	A	A	A	A	A	A	A	A	A	A	A	A		
RM3025	9	16.39	A	A	A	A	A	H	A	A	A	A	A	A	A	A	A	A	A	A	A	A	A	A	A	H	H	A	A	A	A	A	A	A	A	A	A	A		
RM3533	9	17.89	A	A	A	A	A	-	A	A	A	A	A	A	A	A	A	A	A	A	A	A	A	A	A	-	H	A	A	H	A	A	A	A	A	A	A	A	red 2/2	
RM5786	9	19.79	A	A	A	A	A	H	A	A	A	A	A	A	A	A	A	A	A	A	A	A	A	A	A	-	H	A	A	H	A	A	A	A	A	A	A	A		
IDR1309	9	21.26	A	A	A	A	A	-	A	A	A	A	A	A	A	A	A	A	A	A	A	A	A	A	A	-	H	A	A	H	A	A	A	A	A	A	A	A		
IDR1314	9	22.91	A	A	A	A	A	H	A	A	A	A	A	A	A	A	A	A	A	A	A	A	A	A	A	-	H	A	A	A	A	A	A	A	A	A	A	A		
RM7492	10	0.05	A	A	A	A	A	A	A	A	A	A	A	A	A	A	A	A	A	A	A	A	A	A	A	A	A	H	A	A	A	A	A	A	A	A	A	A		
IDR1326	10	2.85	A	A	A	A	A	A	A	A	A	A	A	A	A	A	A	A	A	A	A	A	A	A	A	A	A	H	A	A	A	A	A	A	A	A	A	A		
IDR1341	10	6.01	A	A	A	H	A	A	A	A	A	A	A	A	A	A	A	A	A	A	A	A	A	A	A	A	A	H	H	A	A	A	A	A	A	H	A	A		
RM1126	10	9.77	A	A	A	A	A	A	A	A	A	A	A	A	A	A	A	A	A	A	A	A	A	A	A	A	A	H	H	A	A	A	A	A	A	A	A	A		
RM5758	10	12.99	A	A	A	A	A	A	A	A	A	A	A	A	A	A	A	A	A	A	A	A	A	A	A	A	A	H	-	A	A	A	A	A	A	A	A	A		
IDR1392	10	15.92	A	A	A	A	A	A	A	A	A	A	A	A	A	A	A	A	A	A	A	A	A	A	A	A	A	H	A	A	A	A	A	A	A	A	A	A		
IDR1403	10	19.08	A	A	A	A	A	A	A	H	A	A	A	A	A	A	A	A	A	A	A	A	A	A	A	A	A	H	A	H	A	A	A	A	A	A	A	A		
RM6824	10	23.11	A	A	A	A	A	A	A	H	A	A	A	A	A	A	A	A	A	H	A	H	A	A	A	A	A	A	A	H	A	A	A	A	A	A	A	A	blue 2/4	blue 2/4
RM3668	11	1.46	A	A	A	A	A	A	A	A	A	A	A	A	A	A	A	A	A	A	A	A	A	A	A	A	A	A	A	A	H	A	A	A	A	A	A	A		
IDR1433	11	3.83	A	A	A	A	-	A	A	A	A	A	A	A	A	A	A	A	A	A	A	A	A	A	A	A	A	A	A	A	H	A	A	A	A	H	A	A		
IDR1449	11	7.54	A	A	A	A	H	A	A	A	A	A	A	A	A	A	A	A	A	A	A	A	A	A	A	-	A	A	A	A	H	H	A	A	A	H	A	A		
IDR1467	11	11.58	A	A	A	A	-	A	A	A	A	A	A	A	A	A	A	A	A	A	A	A	A	A	A	A	A	A	A	A	H	H	H	A	A	H	A	A		
RM5824	11	14.53	A	A	A	A	A	A	A	A	A	A	A	A	A	A	A	A	A	A	A	A	A	A	A	A	A	A	A	A	H	H	H	A	A	H	A	A	red 1/4	
RM5582	11	18.31	A	A	A	-	A	A	A	A	A	A	A	A	A	A	A	A	A	A	A	A	A	A	A	A	A	A	A	A	-	H	H	A	A	-	A	A		
IDR1516	11	22.31	A	A	A	-	A	A	A	A	A	A	A	A	A	A	A	A	A	A	A	A	A	A	A	A	A	A	A	A	A	H	H	A	A	A	A	A		
IDR1530	11	25.19	A	A	A	H	A	A	A	A	A	A	A	A	A	A	A	A	A	A	A	A	A	A	A	A	A	A	A	A	A	H	H	A	A	A	A	A		red 1/3
IDR1548	11	28.99	A	A	A	A	A	A	A	A	A	A	A	A	A	A	A	A	A	A	A	A	A	A	A	A	A	A	A	A	A	A	H	A	A	A	A	A		
RM1080	12	0.91	A	A	A	A	A	A	A	-	A	A	A	A	A	A	A	A	-	A	A	A	A	A	A	A	A	A	A	A	-	A	A	H	A	H	H	A		
RM3747	12	2.31	A	A	A	A	A	A	A	H	A	A	A	A	A	A	A	A	H	A	A	A	A	A	A	A	A	A	A	A	H	A	A	H	A	H	H	A		
RM6998	12	4.75	A	A	A	A	A	A	A	H	A	A	A	A	A	A	A	A	-	A	A	A	A	A	A	A	A	A	A	A	A	A	A	H	A	H	A	A	red 2/3	
RM2529	12	7.57	A	A	A	A	A	A	-	-	A	A	A	A	A	A	A	A	A	A	A	A	A	A	A	A	A	A	A	A	A	A	A	H	-	H	A	A		
IDR1595	12	10.07	A	A	A	A	A	A	H	A	A	A	A	A	A	A	A	A	A	A	A	A	A	A	A	A	A	A	A	A	A	A	A	H	H	H	A	A		
IDR1614	12	13.80	A	A	A	A	A	A	A	A	A	A	A	A	A	A	A	A	A	A	A	A	A	A	A	A	A	A	A	A	A	A	A	H	H	A	A	A		
RM1261	12	17.54	A	A	A	A	A	A	A	A	A	A	A	A	A	A	A	A	A	A	A	A	A	A	A	A	A	A	A	A	A	A	-	-	H	A	A	A		
IDR1649	12	21.01	A	A	A	A	A	A	A	A	A	A	A	A	A	A	A	A	A	A	A	A	A	A	A	A	A	A	A	A	A	A	-	A	H	A	A	A		
RM2197	12	27.38	A	A	A	A	A	A	A	A	A	A	A	A	A	A	A	A	A	A	A	A	A	A	A	A	A	A	A	A	A	A	A	A	-	A	-	H		

[a]Y indicates the presence, and N the absence, of a QTL.

[b]A indicates a homozygous Koshihikari allele; H indicates a heterozygous allele.

[c]QTLs with Koshihikari alleles that decrease trait values are shown in red boxes, while those that increase trait values are shown in blue boxes.
The numbers to the right of each box indicate the number of populations in which the QTL was detected, relative to the total number of populations examined.

Supplemental Table 11. Mean grain length and grain width in 31 LAK-BC_4F_2 populations, along with graphical genotypes of their parental BC_4F_1 plants.

Line name			12-9603	12-9604	12-9605	12-9606	12-9607	12-9608	12-9609	12-9610	12-9611	12-9612	12-9613	12-9614	12-9615	12-9616	12-9617	12-9618	12-9619	12-9620	12-9621	12-9622	12-9623	12-9624	12-9625	12-9626	12-9627	12-9628	12-9629	12-9630	12-9631	12-9632	12-9633	Positions of QTL[c]	
Grain length	Mean±SD (mm)		7.07±0.31	7.18±0.30	7.25±0.32	7.03±0.37	7.17±0.37	7.08±0.32	7.08±0.30	7.28±0.33	7.65±0.39	7.64±0.39	7.24±0.31	7.11±0.35	7.15±0.35	7.24±0.30	7.11±0.30	7.52±0.35	7.19±0.31	7.02±0.31	7.18±0.35	7.21±0.32	7.14±0.31	7.21±0.32	7.08±0.30	7.17±0.32	7.06±0.30	7.12±0.33	7.16±0.35	7.19±0.33	7.24±0.36	7.29±0.33	7.36±0.37		
	QTL detection (Y/N)[a]		Y	N	Y	Y	Y	Y	Y	Y	Y	Y	Y	Y	Y	N	Y	Y	N	Y	N	N	N	Y	Y	Y	N	Y	N	N	Y	Y	Y		
Grain width	Mean±SD (mm)		3.45±0.16	3.50±0.16	3.46±0.14	3.32±0.16	3.37±0.16	3.40±0.15	3.33±0.16	3.43±0.18	3.38±0.17	3.30±0.19	3.52±0.18	3.38±0.17	3.41±0.16	3.40±0.17	3.43±0.16	3.28±0.16	3.44±0.15	3.44±0.15	3.45±0.18	3.44±0.76	3.38±0.15	3.45±0.15	3.42±0.14	3.41±0.16	3.42±0.15	3.43±0.16	3.40±0.17	3.42±0.16	3.44±0.18	3.37±0.16	3.34±0.16		
	QTL detection (Y/N)[a]		Y	N	Y	Y	Y	N	N	N	Y	Y	Y	Y	Y	Y	N	Y	N	N	N	N	N	N	Y	Y	N	Y	N	N	Y	N	Y		
Genotypes[b]																																		L	W
Marker	Chr. No.	Position (Mb)																																	
RM8047	1	0.30	H	A	A	A	A	A	A	A	A	A	A	A	A	A	A	A	A	A	A	A	A	A	A	A	A	A	A	A	A	A	A		
RM3148	1	0.75	H	A	A	A	A	A	A	A	A	A	A	A	A	A	A	A	A	A	A	A	A	A	A	A	A	A	A	A	A	A	A		
RM5423	1	2.17	H	A	A	A	A	A	A	A	A	A	A	A	A	A	A	A	A	A	A	A	A	A	A	A	A	A	A	A	A	A	A		
RM3740	1	2.80	H	A	A	A	A	A	A	A	A	A	A	A	A	A	A	A	A	A	A	A	A	A	A	A	A	A	A	A	A	A	A		
RM5336	1	3.37	H	A	A	A	A	A	A	A	A	A	A	A	A	A	A	A	A	A	A	A	A	A	A	A	A	A	A	A	A	A	A		
RM5641	1	4.97	H	A	A	A	A	A	A	A	A	A	A	A	A	A	A	A	A	A	A	A	A	A	A	A	A	A	A	A	A	A	A	1/1	1/1
RM5443	1	6.23	H	A	A	A	A	A	A	A	A	A	A	A	A	A	A	A	A	A	A	A	A	A	A	A	A	A	A	A	A	A	A		
RM3598	1	7.35	H	A	A	A	A	A	A	A	A	A	A	A	A	A	A	A	A	A	A	A	A	A	A	A	A	A	A	A	A	A	A		
RM8133	1	9.39	H	A	A	A	A	A	A	A	A	A	A	A	A	A	A	A	A	A	A	A	A	A	A	A	A	A	A	A	A	A	A		
RM8094	1	11.24	H	A	A	A	A	A	A	A	A	A	A	A	A	A	A	A	A	A	A	A	A	A	A	A	A	A	A	A	A	A	A		
RM10812	1	12.94	H	A	A	A	A	A	A	A	A	A	A	A	A	A	A	A	A	A	A	A	A	A	A	A	A	A	A	A	A	A	A		
RM6681	1	15.90	H	A	A	A	A	A	A	A	A	A	A	A	A	A	A	A	A	A	A	A	A	A	A	A	A	A	A	A	A	A	A		
RM5422	1	18.76	H	-	A	A	A	A	A	A	A	A	A	A	A	A	A	A	A	A	A	A	A	A	A	A	A	A	A	A	A	A	A		
RM11069	1	19.37	H	H	A	A	A	A	A	A	A	A	A	A	A	A	A	A	A	A	A	A	A	A	A	A	A	A	A	A	A	A	A		
RM6716	1	23.12	A	H	A	A	A	A	A	A	A	A	A	A	A	A	A	A	A	A	A	A	A	A	A	-	-	A	A	A	A	A	-		
RM7266	1	24.45	A	H	-	A	A	A	A	A	A	A	A	A	A	A	A	A	A	A	A	A	A	A	A	H	H	A	A	A	A	A	H		
RM1349	1	25.07	A	-	H	A	A	A	A	A	A	A	A	A	A	A	A	A	A	A	A	A	A	A	A	-	-	A	A	A	A	A	-		
RM5461	1	26.91	A	H	H	A	A	A	A	A	A	A	A	A	A	A	A	A	A	A	A	A	A	A	A	A	A	A	A	A	A	A	H		
RM8126	1	29.28	A	-	H	A	A	A	A	A	A	A	A	A	A	A	A	A	A	A	A	A	A	A	A	A	A	A	A	A	A	A	H		1/2
RM5914	1	31.51	A	-	H	A	A	A	A	A	A	A	A	A	A	A	A	A	A	A	A	A	A	A	A	A	A	A	A	A	A	A	H	2/2	
RM3416	1	32.89	A	A	H	A	A	A	A	A	A	A	A	A	A	A	A	A	A	A	A	A	A	A	A	A	A	A	A	A	A	A	H		
RM5501	1	34.55	A	A	H	A	A	A	A	A	A	A	A	A	A	A	A	A	A	A	A	A	A	A	A	A	A	A	A	A	A	A	A		
RM5781	1	37.52	A	A	H	A	A	A	A	A	A	A	A	A	A	A	A	A	A	A	A	A	A	A	A	A	A	A	A	A	A	A	A		
RM11998	1	38.79	A	A	H	A	A	A	A	A	A	A	A	A	A	A	A	A	A	A	A	A	A	A	A	A	A	A	A	A	A	A	A		
RM1387	1	40.21	A	A	H	A	A	A	A	A	A	A	A	A	A	A	A	A	A	A	A	A	A	A	A	A	A	A	A	A	A	A	A		
RM8087	1	41.68	A	A	H	-	A	-	A	A	A	-	A	A	-	A	A	A	A	A	A	A	A	A	A	A	A	A	A	A	A	A	A		
RM8137	1	42.93	A	A	H	H	A	H	A	A	A	H	A	A	H	A	A	A	A	A	A	A	A	A	A	A	A	A	A	A	A	A	A		2/5
RM7622	2	0.29	-	-	-	H	A	H	H	-	-	-	-	A	-	-	-	-	-	-	A	-	-	-	A	-	-	-	-	-	-	H	-		
RM6367	2	1.13	A	A	A	-	A	H	H	A	A	A	A	A	A	A	A	A	A	A	A	A	A	A	H	A	A	A	A	A	A	H	A		
RM7562	2	1.88	A	A	A	H	A	H	H	A	A	A	A	A	A	A	A	A	A	A	A	A	A	A	H	A	A	A	A	A	A	H	A	4/5	
RM5345	2	4.21	A	A	A	H	A	-	H	A	A	A	A	A	A	A	A	A	A	A	A	A	A	A	H	A	A	A	A	A	A	-	A		
RM5897	2	6.73	A	A	A	H	A	A	H	A	A	A	A	A	-	A	A	A	A	A	A	A	A	A	A	A	A	A	A	A	A	A	A		
RM12729	2	6.75	A	A	A	H	A	A	H	A	A	A	A	A	A	A	A	A	A	A	A	A	A	A	A	A	A	A	A	A	A	A	A		
RM5699	2	8.98	A	A	A	H	H	A	-	A	A	-	A	A	A	A	A	A	A	A	A	A	A	A	A	A	A	A	A	A	A	A	A		2/2
RM1234	2	11.34	A	A	A	H	H	A	A	A	A	H	A	A	A	A	A	A	A	A	A	A	A	A	A	A	A	A	A	A	A	A	A		
RM13039	2	11.67	A	A	A	H	H	A	A	A	A	A	A	A	A	A	A	A	A	A	A	A	A	A	A	A	A	A	A	A	A	A	A	1/3	
RM13084	2	12.94	A	A	A	H	H	A	A	A	A	H	A	A	A	A	A	A	A	A	A	A	A	A	A	A	A	A	A	A	A	A	A		
RM13105	2	13.51	A	A	A	H	H	A	A	A	A	H	A	A	A	A	A	A	A	A	A	A	A	A	A	A	A	A	A	A	A	A	A		
RM13113	2	13.96	A	A	A	H	H	A	A	A	A	H	A	A	A	A	A	A	A	A	A	A	A	A	A	A	A	A	A	A	A	A	A		2/3
RM6374	2	15.19	A	A	A	H	H	A	A	A	A	H	A	A	A	A	A	A	A	A	A	A	A	A	A	A	A	A	A	A	A	A	A		
RM13228	2	17.10	A	A	A	H	H	A	A	A	A	H	A	A	A	A	A	A	A	A	A	A	A	A	A	A	A	A	A	A	A	A	A		
RM5578	2	18.64	A	A	A	-	H	-	A	A	A	H	A	A	A	A	A	A	A	A	A	A	A	A	A	A	A	A	A	A	A	A	A		
RM3858	2	19.19	A	A	A	H	H	H	A	A	A	H	A	A	A	A	A	A	A	A	A	A	A	A	A	A	A	A	A	A	A	A	A		
RM6509	2	19.34	A	A	A	A	-	H	A	A	A	-	A	A	A	A	A	A	A	A	A	A	A	A	A	A	A	A	A	A	A	A	A		
RM1379	2	20.06	A	A	A	A	H	H	A	A	A	H	A	A	A	A	A	A	A	A	A	A	A	A	A	A	A	A	A	A	A	A	A		
RM13534	2	22.76	A	A	A	A	-	H	A	A	A	H	A	A	A	A	A	A	A	A	A	A	-	A	A	A	A	A	A	-	-	A	A		
RM3515	2	24.02	A	A	A	A	A	H	A	A	A	H	A	A	A	A	A	A	A	A	A	A	H	A	A	A	A	A	A	H	H	A	A		
RM13728	2	26.81	A	A	A	A	A	H	H	A	A	A	A	A	A	A	A	A	A	A	A	A	A	A	A	A	A	H	A	H	H	A	A		
RM3212	2	28.87	A	A	A	A	A	A	-	A	A	A	A	A	A	A	A	A	A	A	A	A	A	A	A	A	A	-	A	H	-	A	A		
RM6933	2	29.31	A	A	A	A	A	-	H	A	A	A	A	A	A	A	A	A	A	A	A	A	A	A	A	A	A	-	A	H	H	A	A		
RM3421	2	29.92	A	A	A	A	A	A	-	A	A	A	A	A	A	-	A	A	A	A	A	A	A	A	A	-	-	A	A	H	-	A	A		
RM13958	2	31.20	A	A	A	A	A	A	H	A	A	A	A	A	A	H	A	A	A	A	A	A	A	A	A	H	H	A	A	H	H	A	A		
RM7286	2	33.00	A	A	A	A	A	A	H	A	A	A	A	A	A	-	A	A	A	A	A	A	A	A	A	H	H	A	A	H	H	A	A		
RM3850	2	35.43	A	A	A	A	A	A	H	H	A	A	H	A	A	A	A	A	A	A	A	A	A	A	A	H	H	A	A	A	A	A	A		
RM4108	3	0.54	A	A	A	A	A	A	A	H	A	A	A	A	A	A	A	A	A	A	A	A	A	A	A	A	A	A	A	A	A	A	A	1/1	
RM7072	3	2.57	A	A	A	A	A	A	A	H	-	A	A	A	A	A	A	A	A	A	A	A	A	A	A	A	A	A	A	A	A	A	A		
RM14407	3	3.05	A	A	A	A	A	A	A	H	H	A	A	A	A	A	A	A	A	A	A	A	A	A	A	A	A	A	A	A	A	A	A		
RM2791	3	4.57	A	A	A	A	A	A	A	H	-	A	A	A	A	A	A	A	A	A	A	A	A	A	A	-	A	A	A	A	A	A	A		1/4
RM3864	3	5.86	A	A	A	A	A	A	A	H	H	H	A	A	A	A	A	A	A	A	A	A	A	A	A	H	A	A	A	A	A	A	A		
RM5513	3	6.28	A	A	A	A	A	A	A	H	-	H	A	A	A	A	A	A	A	A	A	A	A	A	A	-	A	A	A	A	A	A	A		
RM3872	3	6.85	A	A	A	A	A	A	A	H	H	-	A	A	A	A	A	A	A	A	A	A	A	A	A	-	A	A	A	A	A	A	A		
RM5639	3	8.20	A	A	A	A	A	A	A	H	-	A	A	A	A	A	A	A	A	A	A	A	A	A	A	A	A	A	A	A	A	A	A		
RM4321	3	10.17	A	A	A	A	A	A	A	H	H	A	A	A	A	A	A	A	A	A	A	A	A	A	A	A	A	A	A	A	A	A	A		
RM5551	3	12.93	A	A	A	A	A	A	A	A	H	A	A	A	A	A	A	A	A	A	A	A	A	A	A	A	A	A	A	A	A	A	A		
RM2917	3	14.39	A	A	A	A	A	A	A	A	H	A	A	A	A	A	A	A	A	A	A	A	A	A	A	A	A	A	A	A	A	A	A		
RM3204	3	14.99	A	A	A	A	A	A	A	A	H	A	A	A	A	A	A	A	A	A	A	A	A	A	A	A	A	A	A	A	A	A	A		
RM6914	3	17.29	A	A	A	A	A	A	A	A	H	H	A	A	A	A	-	H	A	A	A	A	A	A	A	A	A	A	A	A	A	A	A		
RM5684	3	18.41	A	A	A	A	A	A	A	A	H	-	A	A	A	A	H	H	A	A	A	A	A	A	A	A	A	A	A	A	A	A	A	3/4	
RM2453	3	20.25	A	A	A	A	A	A	A	A	-	H	A	A	A	A	-	H	A	A	A	A	A	A	A	A	A	A	A	A	A	A	A		
RM15381	3	22.15	A	A	A	A	A	A	A	A	H	H	A	A	A	A	A	A	A	A	A	A	A	A	A	A	A	A	A	A	A	A	A		
RM3513	3	25.11	A	A	A	A	A	A	A	A	A	H	H	A	A	A	A	A	A	A	A	A	A	A	A	A	A	A	A	A	A	A	A		
RM15630	3	26.08	A	A	A	A	-	A	A	A	A	-	H	-	A	A	A	A	A	A	A	A	A	A	A	A	A	A	A	A	A	A	A	1/1	
RM2334	3	26.75	A	A	A	A	H	A	A	A	A	H	H	H	A	A	A	A	A	A	A	A	A	A	A	A	A	A	A	A	A	A	A		
RM2614	3	27.43	A	A	A	A	H	A	A	A	A	H	H	-	A	A	A	A	A	A	A	A	A	A	A	A	A	A	A	A	A	A	A		
RM1350	3	28.68	A	A	A	-	H	A	A	A	A	H	H	H	A	A	A	A	A	A	A	A	A	A	A	A	A	A	A	A	A	A	A		2/5
RM3525	3	30.39	A	A	A	H	H	A	A	A	A	H	-	H	A	A	A	A	A	A	A	A	A	A	A	A	A	A	A	A	A	A	A		
RM6970	3	32.44	A	A	A	H	H	A	A	A	A	H	A	H	A	A	A	A	A	A	A	A	A	A	A	A	A	A	A	A	A	A	A		1/4
RM1373	3	33.72	A	A	A	-	-	A	A	A	A	-	A	H	A	A	A	A	A	A	A	A	A	A	A	A	A	A	A	A	A	A	A	1/1	
RM3329	3	35.60	A	A	A	A	A	A	A	A	A	H	A	H	A	A	A	A	A	A	A	A	A	A	A	A	A	A	A	A	A	A	A		
RM7389	3	36.16	A	A	A	A	A	A	A	A	A	H	A	H	A	A	A	A	A	A	A	A	A	A	A	A	A	A	A	A	A	A	A		
RM7535	4	1.17	A	A	A	A	A	A	A	A	A	A	A	A	H	A	A	A	A	A	A	A	A	A	A	A	A	H	A	A	A	A	A		
RM5414	4	2.03	A	A	A	A	A	A	A	A	A	A	A	A	H	A	A	A	A	A	A	A	A	A	A	A	A	H	A	A	A	A	A	2/2	
RM7200	4	4.04	A	A	A	A	A	A	A	A	A	A	A	A	H	A	A	A	A	A	A	A	A	A	A	A	A	H	A	A	A	A	A		
RM16507	4	6.94	A	A	A	A	A	A	A	A	A	A	A	A	H	A	A	A	A	A	A	A	A	A	A	A	A	-	A	A	A	A	A		
RM5953	4	9.39	A	A	A	A	A	A	A	A	A	A	A	A	H	A	A	A	A	A	A	A	A	A	A	A	A	A	A	A	A	A	A	1/1	
RM16621	4	12.36	A	A	A	A	A	A	A	A	A	A	A	A	H	A	A	A	A	A	A	A	A	A	A	A	A	A	A	A	A	A	A		
RM16670	4	13.88	A	A	A	A	A	A	A	A	A	A	A	A	H	A	A	A	A	A	A	A	A	A	A	A	A	A	A	A	A	A	A		
RM1236	4	15.35	A	A	A	A	A	A	A	A	A	A	A	A	H	A	A	A	A	A	A	A	A	A	A	A	A	A	A	A	A	A	A		
RM5586	4	19.90	A	A	A	A	A	A	A	A	A	A	A	A	H	A	A	A	A	A	A	A	A	A	A	A	A	A	A	A	A	A	A		
RM3742	4	19.92	A	A	A	A	A	A	A	A	A	A	A	A	H	H	A	A	A	A	A	A	A	A	-	A	A	A	A	A	A	-	A		
RM1359	4	20.03	A	A	A	A	A	A	A	A	A	A	A	A	H	H	A	A	A	A	A	A	A	A	H	A	A	A	A	A	A	H	A	1/3	
RM6997	4	21.27	A	A	A	A	A	A	A	A	A	A	A	A	H	H	A	A	A	-	A	A	A	A	-	A	A	A	A	A	A	H	A		
RM2521	4	23.22	A	A	A	A	A	A	A	A	A	A	A	A	H	H	A	A	A	H	A	A	A	A	A	A	A	A	A	A	A	A	A		
RM3785	4	24.25	A	A	A	A	A	A	A	A	A	A	A	A	-	H	A	A	A	-	A	A	A	A	A	-	A	A	A	A	A	A	A		1/1
RM1354	4	26.30	A	A	A	A	A	A	A	A	A	A	A	A	A	H	A	A	A	A	A	A	A	A	A	H	A	A	A	A	A	A	A		
RM1703	4	29.61	A	A	A	A	A	A	A	A	A	A	A	A	A	H	A	A	A	A	A	A	A	A	A	-	A	A	A	A	A	A	A		
RM3474	4	29.89	A	A	A	A	A	A	A	A	A	A	A	A	A	H	-	A	A	H	A	A	A	A	A	H	A	A	A	A	A	A	A	2/3	
RM6909	4	32.06	A	A	A	A	A	A	A	A	A	A	A	A	A	H	H	A	A	H	A	A	A	A	A	H	A	A	A	A	A	A	A		
RM3648	4	33.31	A	A	A	A	A	A	A	A	A	A	A	A	A	A	H	A	A	H	A	A	A	A	A	-	A	A	A	A	A	A	A		
RM17632	4	34.35	A	A	A	A	A	A	A	A	-	A	A	A	A	-	H	A	A	-	A	A	A	A	A	-	-	A	A	A	A	A	A		
RM2431	4	35.10	A	A	A	A	A	A	A	A	A	A	A	A	A	A	H	A	A	A	A	A	A	A	A	A	A	A	A	A	A	A	A		
RM3529	5	0.11	A	A	A	A	A	A	A	A	A	A	A	A	A	A	-	H	A	A	A	A	A	A	A	A	A	A	A	A	A	A	A		
RM5579	5	1.92	A	A	A	A	A	A	A	A	A	A	A	A	A	A	A	H	A	A	A	A	A	A	A	A	A	A	A	A	A	A	A		
RM1366	5	2.92	A	A	A	A	A	A	A	A	A	A	A	A	A	A	A	H	A	A	A	A	A	A	A	A	A	A	A	A	A	A	A		
RM18055	5	5.88	A	A	A	A	A	A	A	A	A	A	A	A	A	A	A	H	A	A	A	A	A	A	A	A	A	A	A	A	A	A	A		1/1
RM7118	5	6.09	A	A	A	A	A	A	A	A	A	A	A	A	A	A	A	H	A	A	A	A	A	A	A	A	A	A	A	A	A	A	A		
RM2744	5	8.30	A	A	A	A	A	A	A	A	A	A	A	A	A	A	A	H	A	A	A	A	A	A	A	A	A	A	A	A	A	A	A		
RM18239	5	10.67	A	A	A	A	A	A	A	A	A	A	A	A	A	A	A	H	A	A	A	A	A	A	A	A	A	A	A	A	A	A	-		
RM6229	5	13.61	A	A	A	A	A	A	A	A	A	A	A	A	A	A	A	H	A	A	A	A	A	A	A	A	A	A	A	A	A	A	A		
RM6724	5	13.83	A	A	A	A	A	A	A	A	A	A	A	A	A	A	A	H	-	A	A	A	A	A	A	A	A	A	A	A	A	A	A		
RM18467	5	16.63	A	A	A	A	A	A	A	A	A	A	A	A	A	A	A	H	H	A	A	A	A	A	A	A	A	A	A	A	A	A	A		
RM1386	5	20.06	A	A	A	A	A	A	A	A	A	A	A	A	A	A	A	H	H	A	A	A	A	A	A	A	A	A	A	A	A	A	A		
RM3295	5	22.33	A	A	A	A	A	A	A	A	H	A	A	A	A	A	A	H	H	H	A	A	A	A	A	A	A	A	A	A	A	A	A		

Line name			12-9603	12-9604	12-9605	12-9606	12-9607	12-9608	12-9609	12-9610	12-9611	12-9612	12-9613	12-9614	12-9615	12-9616	12-9617	12-9618	12-9619	12-9620	12-9621	12-9622	12-9623	12-9624	12-9625	12-9626	12-9627	12-9628	12-9629	12-9630	12-9631	12-9632	12-9633		
RM3476	5	23.91	A	A	A	A	A	A	A	A	H	A	A	A	A	A	A	H	-	-	A	A	A	A	A	A	A	A	A	A	A	A	A		
RM18959	5	25.23	A	A	A	A	A	A	A	A	-	A	-	A	A	A	A	H	H	H	A	A	A	A	A	A	A	A	A	A	A	A	A		
RM3809	5	26.65	A	A	A	A	A	A	A	A	A	A	A	A	A	A	A	-	-	H	A	A	A	A	A	A	A	A	A	A	A	A	A		
RM5784	5	27.86	A	A	A	A	A	A	A	A	A	A	A	A	A	A	A	H	H	H	A	A	A	A	A	A	A	A	A	A	A	A	A		
RM6339	5	28.15	A	A	A	A	A	A	A	A	A	A	A	A	A	A	A	-	-	H	A	A	A	A	A	A	A	A	A	A	A	A	A		
RM1054	5	29.23	A	A	A	A	A	A	A	A	A	A	A	A	A	A	A	A	A	H	A	A	A	A	A	A	A	A	A	A	A	A	A		
RM4332	6	0.72	A	A	A	A	A	A	A	A	A	A	A	A	A	H	A	A	A	A	H	A	A	A	A	A	H	A	-	-	-	A	A		
RM2615	6	5.96	A	A	A	A	A	A	A	A	A	A	A	A	A	A	A	A	A	A	H	H	A	A	A	A	A	A	A	A	A	A	A		
RM5855	6	7.41	A	A	A	A	A	A	A	A	A	A	A	A	A	A	A	A	A	A	-	H	A	A	A	A	A	A	A	A	A	-	A		
RM5963	6	8.82	A	A	A	A	A	A	A	A	A	A	A	A	A	A	A	A	A	A	A	H	A	A	A	A	A	A	A	A	A	H	A		
RM7213	6	10.61	A	A	A	A	A	A	A	A	A	A	A	A	A	A	A	A	A	A	A	H	A	A	A	A	A	A	A	A	A	H	A	1/2 (red)	
RM19985	6	13.89	A	A	A	A	A	A	A	A	A	A	A	A	A	A	A	A	A	A	A	H	-	A	A	A	A	A	A	A	A	-	A		
RM20039	6	16.04	A	A	A	A	A	A	A	A	A	A	A	A	A	A	A	A	A	A	A	H	H	A	A	A	A	A	A	A	A	A	A		
RM20084	6	17.07	A	A	A	A	A	A	A	A	A	A	A	A	A	A	A	A	A	A	A	H	-	A	A	A	A	A	A	A	A	A	A		
RM7193	6	20.26	A	A	A	A	A	A	A	A	A	A	A	A	A	A	A	A	A	A	A	H	H	A	A	A	A	A	A	A	A	A	A		
RM3498	6	20.98	A	A	A	A	A	A	A	A	A	A	A	A	A	A	A	A	A	A	A	H	-	-	A	A	A	A	A	A	A	A	A		
RM1340	6	23.34	A	A	A	A	A	A	A	A	A	A	A	A	A	A	A	A	A	A	A	H	H	H	A	A	A	A	A	A	A	A	A		
RM20518	6	27.12	A	A	A	A	A	A	A	A	A	A	A	A	A	A	A	A	A	A	A	A	H	H	A	A	A	H	A	A	A	A	A		
RM1370	6	28.53	A	A	A	A	A	A	A	A	A	A	A	A	A	A	A	A	A	A	A	A	-	H	A	A	A	H	A	A	A	A	A		
RM3765	6	29.72	A	A	A	A	A	A	A	A	A	A	A	A	A	A	A	A	A	-	A	A	A	H	A	A	-	-	A	A	A	A	A		
RM3509	6	30.97	A	A	A	A	A	A	A	A	A	A	A	A	A	A	A	A	A	H	A	A	A	H	A	A	H	A	A	A	A	A	A	1/2 (red)	
RM4584	7	0.50	A	A	A	A	A	A	A	A	H	H	A	A	A	A	A	A	A	A	A	A	A	A	H	A	A	A	A	A	A	A	A		
RM5752	7	2.57	A	A	A	A	A	A	A	A	H	-	A	A	A	A	A	A	A	A	A	A	A	A	H	A	A	A	A	A	A	A	A		
RM6872	7	4.66	A	A	A	A	A	A	A	A	H	A	A	A	A	A	A	A	A	A	A	A	A	A	H	A	A	A	A	A	A	A	A		
RM6728	7	5.73	A	A	A	A	A	A	A	A	-	A	A	A	A	A	A	A	A	A	A	A	A	A	H	A	A	A	A	A	A	A	A		
RM8007	7	7.71	A	A	A	A	A	A	A	A	A	A	A	A	A	A	A	A	A	A	A	A	A	A	H	A	A	A	A	A	A	A	A		
RM7074	7	9.01	A	A	A	A	A	A	A	A	A	A	A	A	A	A	A	A	A	A	A	A	A	A	H	A	A	A	A	A	A	A	A		
RM2256	7	9.15	A	A	A	A	A	A	A	A	A	A	A	A	A	A	A	A	A	A	A	A	A	A	H	A	A	A	A	A	A	A	A		
RM5499	7	9.99	A	A	A	A	A	A	A	A	A	A	A	A	A	A	A	A	A	A	A	A	A	A	H	A	A	A	A	A	A	A	A		
RM21364	7	10.25	A	A	A	A	A	A	A	A	A	A	A	A	A	A	A	A	A	A	A	A	A	A	H	A	A	A	A	A	A	A	A		
RM8025	7	13.00	A	A	A	A	A	A	A	A	A	A	A	A	A	A	A	A	A	A	A	A	A	A	H	A	A	A	A	A	A	A	A		
RM5481	7	16.20	A	A	A	A	A	A	A	A	A	A	A	A	A	A	A	A	A	A	A	A	A	A	H	A	A	A	A	A	A	A	A		
RM6767	7	17.47	A	A	A	A	A	A	A	A	-	A	A	A	A	A	A	A	A	A	A	A	A	A	H	A	A	A	A	A	A	A	A	1/1 (red)	1/1 (red)
RM21701	7	19.58	A	A	A	A	A	A	A	A	H	A	A	A	A	A	A	A	A	A	A	A	A	A	H	A	A	A	A	A	A	A	A		
RM3404	7	20.11	A	A	A	A	A	A	A	A	H	A	A	A	A	A	-	A	A	A	A	A	A	A	H	A	A	A	A	A	A	A	A		
RM6152	7	22.13	A	A	A	A	A	A	A	A	-	A	A	A	A	A	-	A	A	A	A	A	A	A	H	A	A	A	A	A	A	A	A		
RM5847	7	23.65	A	A	A	A	A	A	A	A	H	A	A	A	A	A	H	A	A	A	A	A	A	A	A	A	A	A	A	A	A	A	A		
RM1364	7	26.78	A	A	A	A	A	A	A	A	H	A	A	A	A	A	H	A	A	A	A	A	A	A	A	A	A	A	A	A	A	A	A		
RM7601	7	29.04	A	A	A	A	A	A	A	A	H	A	A	A	A	A	A	A	A	A	A	A	A	A	A	A	A	-	A	A	A	A	H		
RM6369	8	0.13	A	A	A	A	A	A	A	A	A	A	A	A	A	A	A	-	-	A	A	-	-	-	A	H	A	A	A	A	A	A	A		
RM8018	8	2.17	A	A	A	A	A	A	A	A	A	A	A	A	A	A	A	A	A	A	A	A	A	A	A	H	A	A	A	A	A	A	H		
RM4955	8	2.96	A	A	A	A	A	A	A	A	A	A	A	A	A	A	A	A	A	A	A	A	A	A	A	H	A	A	A	A	A	A	H		
RM1148	8	3.74	A	A	A	A	A	A	A	A	A	A	A	A	A	A	A	A	A	A	A	A	A	A	A	H	A	H	A	A	A	A	H		
RM22634	8	7.33	A	A	A	A	A	A	A	A	A	A	A	A	A	A	A	A	A	A	A	A	A	A	A	H	A	A	A	A	A	A	A		
RM3395	8	10.29	A	A	A	A	A	A	A	A	A	A	A	A	A	A	A	A	A	A	A	A	A	A	A	H	A	A	A	A	A	A	A		
RM22837	8	12.38	A	A	A	A	A	A	A	A	A	A	A	A	A	A	A	A	A	A	A	A	A	A	A	H	A	A	A	A	A	A	A		
RM22934	8	15.97	A	A	A	A	A	A	A	A	A	H	A	A	A	A	A	A	A	A	A	A	A	A	A	H	A	A	A	A	A	A	A		
RM3153	8	18.99	A	A	A	A	A	A	A	A	A	H	A	-	A	A	A	A	A	A	A	A	A	A	A	H	A	A	A	A	A	A	A		
RM23251	8	22.48	A	A	A	A	A	A	A	A	A	H	A	H	A	A	A	A	A	A	A	A	A	A	A	H	A	A	A	A	A	A	A		
RM5891	8	24.63	A	A	A	A	A	A	A	A	A	-	A	A	A	A	A	A	A	A	A	A	A	A	A	H	A	A	A	A	A	A	A		
RM4154	8	28.25	A	A	A	A	A	A	A	A	A	A	A	A	A	A	A	A	A	A	A	A	A	A	A	H	A	A	A	A	A	A	A		
RM23914	9	7.24	A	A	A	H	H	A	A	A	A	A	A	A	A	A	A	A	A	A	A	A	A	A	A	A	H	A	A	A	A	A	A		
RM5526	9	7.31	A	A	A	H	H	A	A	A	A	A	A	A	A	A	A	A	A	A	A	A	A	A	A	-	-	A	A	A	A	A	A		
RM1328	9	9.21	A	A	A	H	H	A	A	A	A	A	A	A	A	A	A	A	A	A	A	A	A	A	A	H	H	A	A	A	A	A	A		
RM3907	9	10.49	A	A	A	H	H	A	A	A	A	A	A	A	A	A	A	A	A	A	A	A	A	A	A	-	-	A	A	A	A	A	A		
RM1817	9	10.62	A	A	A	H	H	A	A	A	A	A	A	A	A	A	A	A	A	A	A	A	A	A	A	-	-	A	A	A	A	A	A	1/2 (blue)	1/2 (blue)
RM5657	9	14.37	A	A	A	H	H	A	H	A	A	A	A	A	A	A	A	A	A	A	A	A	A	A	H	H	H	A	A	A	A	H	A		
RM3700	9	15.43	A	A	A	H	H	A	-	A	A	A	A	A	A	A	A	A	A	A	A	A	A	A	-	-	-	A	A	A	A	-	A		
RM24559	9	18.45	A	A	A	A	A	A	A	A	A	A	A	A	A	H	A	A	A	A	A	A	A	A	H	H	H	A	A	A	A	H	A		
RM3909	9	19.54	A	A	A	A	A	A	A	A	A	A	A	A	A	-	A	A	A	H	A	A	A	A	-	-	-	A	A	A	A	-	A		1/1 (red)
RM1553	9	21.00	A	A	A	A	A	A	A	A	A	A	A	A	A	H	A	A	A	-	A	A	A	A	A	-	H	A	A	A	A	A	A		
RM6971	9	21.89	A	A	A	A	A	A	A	A	A	A	A	A	A	H	A	A	A	H	A	A	A	A	A	A	H	A	A	A	A	A	A		
RM7492	10	0.05	A	A	A	A	A	A	A	A	A	A	A	A	A	A	A	A	A	A	A	A	A	A	A	A	A	H	A	A	A	A	A		
RM3882	10	2.74	A	A	A	A	A	A	A	A	A	A	A	A	A	A	A	A	A	A	A	A	A	A	A	A	A	H	A	A	A	A	A		
RM25101	10	5.72	A	A	A	A	A	A	A	A	A	A	A	A	A	A	A	A	A	A	A	A	A	A	A	A	A	H	A	A	A	A	A		
RM25151	10	7.62	A	A	A	A	A	A	A	A	A	A	A	A	A	A	A	A	A	A	A	A	A	A	A	A	A	H	A	A	A	A	A		
RM4455	10	11.74	A	A	A	A	A	A	A	A	A	A	A	A	A	A	A	A	A	A	A	A	A	A	A	A	A	H	A	A	A	A	A		
RM25425	10	14.94	A	A	A	A	A	A	A	A	A	A	A	H	A	A	A	A	A	A	A	A	A	A	A	A	A	H	A	A	A	A	A		
RM5620	10	17.47	A	A	A	A	A	A	A	A	A	A	A	H	A	A	A	A	A	A	A	A	A	A	A	A	A	H	A	A	A	A	A		
RM3773	10	19.97	A	A	A	A	A	A	A	A	A	A	A	H	A	A	A	A	A	A	A	A	A	A	A	A	A	H	A	A	A	A	A	1/2 (blue)	1/2 (blue)
RM4771	10	23.09	A	A	A	A	A	A	A	A	A	A	A	H	A	A	A	A	A	A	A	A	A	A	A	A	A	H	A	A	A	A	A		
RM1761	11	0.31	A	A	A	A	A	A	A	A	A	A	A	A	A	H	A	A	A	H	H	A	A	A	H	H	H	A	H	H	A	H	A		1/6 (blue)
RM1812	11	2.41	A	A	A	A	A	A	A	A	A	A	A	A	A	H	A	A	A	H	H	A	A	A	H	H	-	A	H	H	A	H	A		
RM26123	11	3.08	A	A	A	A	A	A	A	A	A	A	A	A	A	H	A	A	A	H	A	A	A	A	A	H	A	A	H	H	A	A	A		
RM4469	11	6.26	A	A	A	A	A	A	A	A	A	A	A	A	A	A	A	A	A	A	A	A	A	A	A	A	A	A	H	H	-	A	A		
RM4862	11	9.99	A	A	A	A	A	A	A	A	A	A	A	A	A	A	A	A	A	A	A	A	A	A	A	A	A	A	A	H	H	A	A		
RM5857	11	11.86	A	A	A	A	A	A	A	A	A	A	A	A	A	A	A	A	A	A	A	A	A	A	A	A	A	A	A	-	H	A	A		
RM26567	11	13.39	A	A	A	A	A	A	A	A	A	A	A	A	A	A	A	A	A	A	A	A	A	A	A	A	A	A	A	H	H	A	A		
RM7226	11	14.51	A	A	A	A	A	A	A	A	A	A	A	A	A	A	A	A	A	A	A	A	A	A	A	A	A	A	A	-	H	A	A		
RM1355	11	17.67	A	A	A	A	A	A	A	A	A	A	A	A	A	A	A	A	A	A	A	A	A	A	A	A	A	A	A	H	H	A	A	1/2 (red)	1/2 (red)
RM5582	11	18.31	A	A	A	A	A	A	A	A	A	A	A	A	A	A	A	A	A	A	A	A	A	A	A	A	A	A	A	-	H	A	A		
RM1341	11	20.14	A	A	A	A	A	A	A	A	A	A	A	A	A	A	A	A	A	A	A	A	A	A	A	A	A	A	A	H	H	A	A		
RM26969	11	21.97	A	A	A	A	A	A	A	A	A	A	A	A	A	A	A	A	A	A	A	A	A	A	A	A	A	A	A	H	H	A	A	1/2 (blue)	
RM27096	11	24.33	A	A	A	A	A	A	A	A	A	A	A	A	A	A	A	A	A	A	A	A	A	A	A	A	A	A	A	-	H	A	A		
RM2064	11	27.49	A	A	A	A	A	A	A	A	A	A	A	A	A	A	A	A	A	A	A	A	A	A	A	A	A	A	A	A	H	A	A		
RM1880	12	0.75	A	A	A	A	A	A	A	A	A	A	A	A	H	A	A	A	A	A	A	A	A	A	A	A	A	A	A	A	A	H	A		
RM27505	12	2.19	A	A	A	A	A	A	A	A	A	A	A	A	H	A	A	A	A	A	A	A	A	A	A	A	A	A	A	A	A	H	A		
RM6998	12	4.75	A	A	A	A	A	A	A	A	A	A	A	A	A	A	A	A	H	A	A	A	A	A	A	A	A	A	A	A	A	H	H		
RM27723	12	5.47	A	A	A	A	A	A	A	A	A	A	A	A	A	A	A	A	H	A	A	A	A	A	A	A	A	A	A	A	A	A	H		
RM1036	12	8.80	A	A	A	A	A	A	A	A	A	A	A	A	A	A	A	A	A	A	A	A	A	A	A	A	A	A	A	A	A	A	H		
RM6973	12	10.08	A	A	A	A	A	A	A	A	A	A	A	A	A	A	A	A	A	A	A	A	A	A	A	A	A	A	A	A	A	A	H		
RM28112	12	16.28	A	A	A	A	A	A	A	A	A	A	A	A	A	A	A	A	A	A	A	A	A	A	A	A	A	A	A	A	A	A	H		
RM1246	12	19.12	A	A	A	A	A	A	A	A	A	A	A	A	A	A	A	A	A	A	A	A	A	A	A	A	A	A	A	A	A	A	H		
RM1986	12	21.25	A	A	A	A	A	A	A	A	A	A	A	A	A	A	A	A	A	A	A	A	A	A	A	A	A	A	A	A	A	A	H		
RM1300	12	26.00	A	A	A	A	A	A	A	A	A	A	A	A	A	A	A	A	A	A	A	A	A	A	A	A	A	A	A	A	A	H	H		
RM2197	12	27.38	A	A	A	A	A	A	A	A	A	A	A	A	A	A	A	A	A	A	A	A	A	A	A	A	A	A	A	A	A	A	H		

[a]Y indicates the presence, and N the absence, of a QTL.

[b]A indicates a homozygous Koshihikari allele; H indicates a heterozygous allele.

[c]QTLs with Koshihikari alleles that decrease trait values are shown in red boxes, while those that increase trait values are shown in blue boxes.
The numbers to the right of each box indicate the number of populations in which the QTL was detected, relative to the total number of populations examined.

Supplemental Table 12. Mean grain length and grain width in 29 OWK-BC_4F_2 populations, along with graphical genotypes of their parental BC_4F_1 plants.

Line name			12-9638	12-9639	12-9640	12-9641	12-9642	12-9643	12-9644	12-9645	12-9646	12-9647	12-9648	12-9649	12-9650	12-9651	12-9652	12-9653	12-9654	12-9655	12-9656	12-9657	12-9658	12-9659	12-9660	12-9661	12-9662	12-9663	12-9664	12-9665	12-9666	Positions of QTL[c] L	Positions of QTL[c] W
Grain length	Mean±SD (mm)		7.21±0.33	7.22±0.36	7.15±0.31	7.25±0.32	7.21±0.34	7.19±0.33	7.17±0.31	7.07±0.35	7.20±0.34	7.12±0.37	7.04±0.33	7.11±0.33	7.09±0.34	7.21±0.35	7.21±0.36	7.24±0.32	7.11±0.35	7.16±0.38	7.15±0.35	7.17±0.32	7.13±0.33	7.07±0.31	7.27±0.32	7.07±0.33	7.22±0.34	7.11±0.34	7.19±0.34	7.21±0.34	7.15±0.34		
	QTL detection (Y/N)[a]		Y	Y	Y	Y	Y	Y	Y	Y	Y	Y	Y	Y	Y	Y	Y	Y	N	Y	Y	N	N	N	Y	N	Y	N	N	N	N		
Grain width	Mean±SD (mm)		3.43±0.17	3.44±0.16	3.38±0.15	3.37±0.16	3.32±0.18	3.37±0.17	3.38±0.15	3.36±0.19	3.35±0.17	3.40±0.18	3.34±0.17	3.37±0.15	3.39±0.16	3.41±0.16	3.42±0.17	3.46±0.15	3.46±0.17	3.33±0.22	3.38±0.18	3.39±0.17	3.37±0.16	3.42±0.15	3.41±0.19	3.38±0.18	3.39±0.15	3.42±0.17	3.35±0.16	3.41±0.16	3.37±0.17		
	QTL detection (Y/N)[a]		Y	Y	N	N	Y	Y	N	Y	Y	Y	Y	N	Y	N	N	Y	Y	Y	N	N	N	Y	N	Y	Y	N	Y	Y	Y		
Genotypes[b]																																	
Marker	Chr. No.	Position (Mb)																															
RM1331	1	1.67	H	A	A	A	A	A	A	A	A	A	A	A	A	A	A	A	A	A	A	A	A	A	A	A	A	A	A	A	A	1/1	
RM7383	1	3.48	-	A	A	A	A	A	A	A	A	A	A	A	A	A	A	A	A	A	A	A	A	A	H	A	A	A	A	A	A		
RM3521	1	4.40	H	A	A	A	A	A	A	A	A	A	A	A	A	A	A	A	A	A	A	A	A	A	H	A	A	A	A	A	A	■	
RM5443	1	6.23	H	H	A	A	A	A	A	A	A	A	A	A	A	A	A	A	A	A	A	A	A	A	-	A	A	A	A	H	A		
RM8078	1	7.28	H	H	A	A	A	A	A	A	A	A	A	A	A	A	A	A	A	A	A	A	A	A	A	A	A	A	A	H	A		3/3
RM3780	1	8.02	H	H	A	A	A	A	A	A	A	A	A	A	A	A	A	A	A	A	A	A	A	A	A	A	A	A	A	H	A		
RM8046	1	9.94	-	H	H	A	A	A	A	A	A	A	A	A	A	A	A	A	A	A	A	A	A	A	A	A	A	A	A	-	A		
RM7075	1	15.12	A	H	H	A	A	A	A	A	A	A	A	A	A	A	A	A	A	A	A	A	A	A	A	A	A	A	A	A	A		
RM11099	1	20.06	A	H	H	A	A	A	A	A	A	A	A	A	A	A	A	A	A	A	A	A	A	A	A	A	A	A	A	A	A		
RM5718	1	22.93	A	-	-	H	A	A	A	A	A	A	A	A	A	A	A	A	A	A	A	A	A	A	A	A	A	A	A	A	A		
RM6716	1	23.12	A	A	H	H	A	A	A	A	A	A	A	A	A	A	A	A	A	A	A	A	A	A	A	A	A	A	A	A	A		
RM5919	1	24.74	A	A	H	H	A	A	A	A	A	A	A	A	A	A	A	A	A	A	A	A	A	A	A	A	A	A	A	A	-	■	
RM3440	1	27.19	A	A	H	H	A	A	A	A	A	A	A	A	A	A	A	A	A	A	A	A	A	A	A	A	A	A	A	A	H		1/3
RM3817	1	28.90	A	A	H	-	A	A	A	A	A	A	A	A	A	A	A	A	A	A	A	A	A	A	A	A	A	A	A	A	-		
RM5931	1	29.28	A	A	-	H	A	A	H	A	A	A	A	A	A	A	A	H	A	H	A	A	A	A	A	A	A	A	A	A	A		
RM5954	1	30.00	A	A	A	-	A	A	H	A	A	A	A	A	A	A	A	-	A	-	A	A	A	A	A	A	A	A	A	A	A		
ID18-24	1	31.90	A	A	A	H	A	A	H	A	A	A	A	A	A	A	A	H	A	H	A	A	A	A	A	A	A	A	A	A	A	1/1	
RM1003	1	33.48	A	A	A	-	A	A	H	A	A	A	A	A	A	A	A	H	A	H	A	A	A	A	A	A	A	A	A	A	A		
RM5501	1	34.55	A	A	A	H	A	A	H	A	A	A	A	A	A	A	A	H	A	H	A	A	A	A	A	A	A	A	A	A	A		
RM6292	1	39.22	A	A	A	H	A	A	H	A	A	A	A	A	A	A	A	H	A	H	A	A	A	A	A	A	A	A	A	A	A	1/4	
RM8137	1	42.93	A	A	A	H	A	A	A	A	A	A	A	A	A	A	H	H	A	A	A	A	A	A	A	A	A	A	A	A	A		
RM2770	2	0.81	A	A	A	A	H	A	A	A	A	A	A	A	A	A	A	A	A	A	A	A	A	A	A	A	A	A	A	A	A		
RM5529	2	2.22	A	A	A	A	H	A	A	A	A	A	A	A	A	A	A	A	A	A	A	A	A	A	A	A	A	A	A	A	A		
RM1075	2	3.83	A	A	A	A	H	A	A	A	A	A	A	A	A	A	A	A	A	A	A	A	A	A	A	A	A	A	A	A	A	1/1	
RM5862	2	6.01	A	A	A	A	H	A	A	A	A	A	A	A	A	A	A	A	A	A	A	A	A	A	A	A	A	A	A	A	A		
RM5340	2	7.48	A	A	A	A	H	A	A	A	A	A	A	A	A	A	A	A	A	A	A	A	A	A	A	A	A	A	A	A	A		
RM6375	2	9.56	A	A	A	A	H	A	A	A	A	A	A	A	A	A	A	A	A	A	A	A	A	A	A	A	A	A	A	A	A		
RM1313	2	11.26	A	A	A	A	H	A	A	A	A	A	A	A	A	A	A	A	A	A	A	A	A	A	A	A	A	A	A	A	A	1/1	
RM6374	2	15.19	A	A	A	A	H	A	A	A	A	A	A	A	A	A	A	A	A	A	A	A	A	A	A	A	A	A	A	A	A		
RM5578	2	18.64	A	A	A	A	H	A	A	A	A	A	A	A	A	A	A	A	A	A	A	A	A	A	A	A	A	A	A	A	A		
RM5430	2	22.80	A	A	A	A	H	H	A	A	A	A	A	A	A	A	A	A	A	A	A	A	A	A	A	A	A	A	A	A	A		2/2
RM263	2	25.87	A	A	A	A	H	H	A	A	A	A	A	A	A	A	A	A	A	A	A	A	A	A	A	A	A	A	A	A	A		
RM1385	2	26.65	A	A	A	A	-	H	A	A	A	A	A	A	A	A	A	A	A	A	A	A	A	A	A	A	A	A	A	A	A	1/1	
RM3512	2	27.32	A	A	A	A	A	H	A	A	A	A	A	A	A	A	A	A	A	A	A	A	A	A	A	A	A	A	A	A	A		
RM3421	2	29.92	A	A	A	A	A	H	-	A	A	A	A	A	A	A	A	A	A	A	A	A	A	A	A	A	A	A	A	A	A		
RM8030	2	32.49	A	A	A	H	A	H	H	A	A	A	A	A	A	A	A	A	A	A	A	A	A	A	A	A	A	A	A	A	A		
RM1255	2	35.26	A	A	A	-	A	-	H	A	A	A	A	A	A	A	A	-	-	-	A	A	A	A	A	A	A	A	A	A	A		
RM6923	2	35.70	A	A	A	H	A	A	H	A	A	A	A	A	A	A	A	H	H	H	A	A	A	A	A	A	A	A	A	A	A		
RM14311	3	1.16	A	A	A	A	A	H	A	H	A	A	A	A	H	A	A	A	A	A	A	A	A	A	A	A	H	A	A	A	A	1/4	1/4
RM7072	3	2.57	A	A	A	A	A	-	A	H	A	A	A	A	-	A	A	A	A	A	A	A	A	A	A	-	-	A	A	A	A		
RM3392	3	3.83	A	A	A	A	A	A	A	H	A	A	A	A	A	A	A	A	A	A	A	A	A	A	A	H	A	A	A	A	A		
RM4992	3	4.72	A	A	A	A	A	A	A	-	A	A	A	A	A	A	A	A	A	H	A	A	A	A	A	-	A	A	A	A	A		
RM3467	3	6.00	A	A	A	A	A	A	A	H	A	A	A	A	A	A	A	A	A	H	A	A	A	A	A	H	A	A	A	A	A		1/3
RM3766	3	6.93	A	A	A	A	A	A	A	-	A	A	A	A	A	A	A	A	A	H	A	A	A	A	A	-	A	A	A	A	A		
RM1338	3	8.39	A	A	A	A	A	A	A	H	A	A	A	A	A	A	A	A	A	H	A	A	A	A	A	A	A	A	A	-	A		
RM5896	3	8.55	A	A	A	A	A	A	A	H	A	A	A	A	A	A	A	A	A	-	A	A	A	A	A	A	A	A	A	-	A	■	
RM14778	3	10.05	A	A	A	A	A	A	A	H	A	A	A	A	A	A	A	A	A	H	A	A	A	A	A	A	A	A	A	H	A		
RM3562	3	11.68	A	A	A	A	A	A	A	H	A	A	A	A	A	A	A	A	A	-	A	A	A	A	A	A	A	A	A	-	A		
RM1350	3	28.68	A	A	A	A	A	A	A	A	H	A	A	A	A	A	A	A	A	A	A	A	A	A	A	A	A	A	A	A	A		1/1
RM5475	3	30.58	A	A	A	A	A	A	A	A	H	A	A	A	A	A	A	A	A	A	A	A	A	A	A	A	A	A	A	A	A		
RM3867	3	31.75	A	-	A	A	A	A	A	A	H	A	A	A	-	A	A	A	A	A	A	A	A	A	A	A	-	A	A	A	A		
RM7076	3	33.66	A	H	A	A	A	A	A	A	H	A	A	A	H	A	A	A	A	A	A	A	A	A	A	A	H	A	A	A	A	3/4	3/4
RM4404	3	35.60	A	H	A	A	A	A	A	A	H	A	A	A	H	A	A	A	A	A	A	A	A	A	A	A	H	A	A	A	A		
RM1221	3	35.68	A	H	A	A	A	A	A	A	H	A	H	A	H	A	A	A	A	A	A	A	A	A	A	A	A	A	A	A	A		
ID15-45	4	0.10	A	A	A	A	A	A	A	A	A	H	A	A	A	A	A	A	A	A	A	A	A	A	A	A	A	A	-	A	A		1/1
RM7535	4	1.17	A	A	A	A	A	A	A	A	A	H	A	A	A	A	A	A	A	A	A	A	A	A	A	A	A	A	A	A	A		
RM5414	4	2.03	A	A	A	A	A	A	A	A	A	H	A	A	A	A	A	A	A	A	A	A	A	A	A	A	A	A	A	A	A	1/1	
RM16449	4	4.91	A	A	A	A	A	A	A	A	A	H	H	A	A	A	A	-	A	A	A	A	A	A	A	A	A	A	-	A	A		
RM7504	4	7.25	A	A	A	A	A	A	A	A	A	H	A	A	A	A	A	H	A	A	A	A	A	A	A	A	A	A	A	A	A		
RM5953	4	9.39	A	A	A	A	A	A	A	A	A	H	H	A	A	A	A	H	A	A	A	A	A	A	A	A	A	A	-	A	A		
RM16616	4	12.05	H	A	A	A	A	A	A	A	A	H	H	A	A	A	A	H	A	A	A	A	A	A	A	A	A	A	-	A	A		
RM16621	4	12.36	H	A	A	A	A	A	A	A	A	H	H	A	A	A	A	H	A	A	A	A	A	A	A	A	A	A	H	A	A		
RM16670	4	13.88	H	A	A	A	A	A	A	A	A	-	H	A	A	A	A	-	A	A	A	A	A	A	A	A	A	A	-	A	A	2/2	
RM16752	4	16.90	-	A	A	A	A	A	A	A	A	H	H	A	A	A	A	A	A	A	H	A	A	A	A	A	A	A	H	A	A		
RM16828	4	18.79	A	A	A	A	A	A	A	A	A	-	H	A	A	A	A	A	A	A	-	A	A	A	A	A	A	A	H	A	A		
RM6679	4	19.90	A	A	A	A	A	A	A	A	A	H	H	A	A	A	A	A	A	A	H	A	A	A	A	A	A	A	H	A	A	1/4	
RM5979	4	20.77	A	A	A	A	A	A	A	A	A	-	H	A	A	A	A	A	A	A	-	A	A	A	A	A	A	A	H	A	A		
RM2521	4	23.22	A	A	A	A	A	A	A	A	A	H	H	A	A	A	A	A	A	A	A	A	A	A	A	A	A	A	H	A	A		2/3
RM1388	4	25.22	A	A	A	A	A	A	A	A	A	-	H	A	A	A	A	A	A	A	A	A	A	A	A	A	A	A	H	A	A		
RM5714	4	27.47	A	A	A	A	A	A	A	A	A	-	H	A	A	A	A	A	A	A	A	A	A	A	A	A	A	A	H	A	A		
RM3474	4	29.89	A	A	A	A	A	A	A	H	A	A	-	A	A	A	A	A	A	A	A	A	A	A	A	A	A	A	-	A	A	1/1	
RM5503	4	30.36	A	A	A	A	A	A	A	H	A	-	H	A	A	A	A	A	A	A	A	A	A	A	A	A	A	A	H	A	A		
RM6748	4	30.87	A	A	A	A	A	A	A	H	A	A	H	A	A	A	A	A	A	A	A	A	A	A	A	A	A	A	H	A	A		
RM1153	4	33.03	A	A	A	A	A	A	A	A	A	A	H	A	A	A	A	A	A	A	A	A	A	A	A	A	A	A	H	A	A		2/2
RM2799	4	34.32	A	A	A	A	A	A	A	A	A	A	H	A	A	A	A	A	A	A	A	A	A	A	A	A	A	A	A	A	A		
RM4777	5	0.45	H	A	A	A	A	A	A	A	A	A	A	H	A	A	A	A	A	A	A	A	A	A	A	A	A	A	A	A	A		1/2
RM18107	5	6.83	-	A	A	A	A	A	A	A	A	A	A	-	-	A	A	A	A	A	A	A	A	A	A	A	A	A	A	A	A		
RM18353	5	14.01	A	A	A	A	A	A	A	A	A	A	A	H	H	A	A	A	A	A	A	A	A	A	A	A	A	A	A	A	A		
RM18465	5	16.62	A	A	A	A	A	A	A	A	A	A	A	H	H	A	A	A	A	A	A	A	A	A	A	A	A	A	A	A	A		
RM2185	5	18.53	A	A	A	A	A	A	A	A	A	A	A	A	H	A	A	A	A	A	A	A	A	A	A	A	A	A	A	A	A		
RM3969	5	19.00	A	A	A	A	A	A	A	A	A	A	A	A	H	A	A	A	A	A	A	A	A	A	A	A	A	A	A	A	A	1/1	
RM4501	5	22.11	A	A	A	A	A	A	A	A	A	A	A	A	H	A	A	A	A	A	A	A	A	A	A	A	A	-	A	A	A		
RM18926	5	24.48	A	A	A	A	A	A	A	A	A	A	A	A	H	A	A	A	A	A	A	A	A	A	A	A	A	H	A	A	A		
RM6360	5	28.14	A	H	A	A	A	A	A	A	A	A	A	H	H	A	A	A	H	A	A	A	A	A	A	A	A	H	A	A	A		
RM1054	5	29.23	A	H	A	A	A	A	A	A	A	A	A	H	H	A	A	A	A	A	A	A	A	A	A	A	A	A	A	A	A		
RM8120	6	0.48	A	A	A	A	A	A	A	A	A	A	A	A	A	H	A	A	A	A	A	A	A	A	H	A	A	A	A	H	A		
RM5199	6	2.27	A	A	A	A	A	A	A	A	A	A	A	A	A	H	A	A	A	A	A	A	A	A	-	A	A	A	-	-	A	1/1	
RM1163	6	4.19	A	A	A	A	A	A	A	A	A	A	A	A	A	H	A	A	A	A	A	A	A	A	A	A	A	A	H	A	A		
RM19725	6	8.12	A	A	A	A	A	A	A	A	A	A	A	A	A	H	H	A	A	A	A	A	A	A	A	A	A	A	A	A	H		
RM19863	6	11.45	A	A	A	A	A	A	A	A	A	A	A	A	A	-	H	A	A	A	A	A	A	A	A	A	A	A	A	A	-		
RM19985	6	13.89	A	A	A	A	A	A	A	A	A	A	A	A	A	H	H	A	A	A	A	A	A	A	A	A	A	A	A	A	-	1/2	
RM20023	6	15.84	A	A	A	A	A	A	A	A	A	A	A	A	A	-	H	A	A	A	A	A	A	A	A	A	A	A	A	A	A		
RM20045	6	16.05	A	A	A	A	A	A	A	A	A	A	A	A	A	H	H	A	A	A	A	A	A	A	A	A	A	A	A	A	A		
RM20182	6	20.23	A	A	A	A	A	A	A	A	A	A	A	A	A	H	H	A	A	A	A	A	A	A	A	A	A	A	A	A	A		
RM3272	6	23.62	A	A	A	A	A	A	A	A	A	A	A	A	A	A	H	A	A	A	A	A	A	A	A	A	A	A	A	A	A		
RM5371	6	25.83	A	A	A	A	A	A	A	A	A	A	A	A	A	A	H	H	A	A	A	A	A	A	A	A	A	A	A	A	A		
RM6926	6	28.59	A	A	A	A	A	A	A	-	A	A	A	A	A	A	A	H	A	A	A	A	A	A	A	A	A	A	A	A	A	1/1	
RM1150	6	30.38	A	A	A	A	A	A	A	H	A	-	-	A	A	A	A	H	A	A	A	A	A	A	A	A	A	A	-	A	A		1/2
RM20788	7	0.16	A	A	A	H	A	A	A	A	A	A	A	A	A	A	A	A	A	A	A	A	A	A	A	A	A	A	A	A	A		
RM2381	7	2.42	A	A	A	-	A	A	A	A	A	A	A	A	A	A	A	-	H	A	A	A	A	A	A	A	A	A	A	A	A		
RM5055	7	2.65	A	A	A	H	A	A	A	A	A	A	A	A	A	A	A	H	H	A	A	A	A	A	A	A	A	A	A	A	A		
RM5711	7	3.14	A	A	A	-	A	A	A	A	A	A	A	A	A	A	A	-	H	A	A	A	A	A	A	A	A	A	A	A	A		
RM1353	7	3.31	A	A	A	H	A	A	A	A	A	A	A	A	A	A	A	-	H	-	A	A	A	A	A	A	A	A	A	A	A		
RM6872	7	4.66	A	A	A	-	A	A	A	A	A	A	A	A	A	A	A	A	H	-	A	A	A	A	A	A	A	A	A	A	H		
RM21237	7	6.75	A	A	A	A	A	A	A	A	A	A	A	A	A	A	A	A	H	H	A	A	A	A	A	A	A	A	A	A	H		
RM3859	7	8.88	A	A	A	A	A	A	A	A	A	A	A	A	A	A	A	A	-	-	A	A	A	A	A	A	A	A	A	A	H		
RM7074	7	9.01	A	A	A	A	A	A	A	A	A	A	A	A	A	A	A	A	-	H	A	A	A	A	A	A	A	A	A	A	-		
RM2256	7	9.15	A	A	A	A	A	A	A	A	A	A	A	A	A	A	A	A	H	H	A	A	A	A	A	A	A	A	A	A	H		

Line name			12-9638	12-9639	12-9640	12-9641	12-9642	12-9643	12-9644	12-9645	12-9646	12-9647	12-9648	12-9649	12-9650	12-9651	12-9652	12-9653	12-9654	12-9655	12-9656	12-9657	12-9658	12-9659	12-9660	12-9661	12-9662	12-9663	12-9664	12-9665	12-9666	QTL (1)	QTL (2)
RM3670	7	13.44	A	A	A	A	A	A	A	A	A	A	A	A	A	A	A	A	H	H	A	A	A	A	A	A	A	A	A	A	A		1/1 (red)
RM4952	7	17.80	A	A	A	A	A	A	A	A	A	A	A	A	A	A	A	A	-	H	A	A	A	A	A	A	A	A	A	A	A		
RM3826	7	20.81	A	A	A	A	A	A	A	A	A	A	A	A	A	A	A	A	A	H	A	A	A	A	A	A	A	A	A	A	A		
RM3753	7	23.66	A	A	A	A	A	A	A	A	A	A	A	A	A	A	A	A	A	H	A	A	A	A	A	A	A	A	A	A	A		
RM5426	7	25.93	A	A	A	A	A	A	A	A	A	A	A	A	A	A	A	A	A	-	A	A	A	A	A	A	A	A	A	H	A		
RM1364	7	26.78	A	A	A	A	A	A	A	A	A	A	A	A	A	A	A	A	A	H	A	A	A	A	A	A	A	A	A	H	A		
RM5720	7	28.67	A	A	A	A	A	A	A	A	A	A	A	A	A	A	A	A	A	-	A	A	A	A	A	A	A	A	A	H	A		
RM1306	7	28.95	A	A	A	A	A	A	A	A	A	A	A	A	A	A	A	A	A	H	A	A	A	A	A	A	A	A	A	H	A		
RM5911	8	0.08	A	A	A	A	A	A	A	A	A	A	A	A	A	A	A	A	A	A	H	A	A	A	A	A	A	A	A	A	A		
RM1381	8	0.42	A	A	-	A	A	A	A	A	A	A	A	A	A	A	A	-	-	-	H	A	A	A	A	A	A	A	A	A	A	1/2 (blue)	
RM22502	8	4.64	A	A	H	A	A	A	A	A	A	A	A	A	A	A	A	A	A	H	A	A	A	A	A	A	A	A	A	A	A		
RM22634	8	7.33	A	A	A	A	A	A	A	A	A	A	A	A	A	A	A	A	A	H	A	A	A	A	A	A	A	A	A	A	A		
RM7267	8	10.19	A	A	A	A	A	A	A	A	A	A	A	A	A	A	A	A	-	H	-	A	A	A	A	A	A	A	A	A	A		
RM6032	8	11.90	A	A	A	A	A	A	A	A	A	A	A	A	A	A	A	A	H	H	H	A	A	A	A	A	A	A	A	A	A		
RM6990	8	14.36	A	A	A	A	A	A	A	A	A	A	A	A	A	A	A	A	H	H	H	A	A	A	A	A	A	A	A	A	A		
RM7285	8	18.76	A	A	A	A	A	A	A	A	A	A	A	A	A	A	A	A	-	H	-	A	A	A	A	A	A	A	A	A	A		
RM4815	8	19.01	A	A	A	A	A	A	A	A	A	A	A	A	A	A	A	A	H	H	H	A	A	A	A	A	A	A	A	A	A		
RM3452	8	24.78	A	A	A	A	A	A	A	A	A	A	A	A	A	A	A	A	A	A	H	H	A	A	A	A	A	A	A	A	A		
RM5493	8	26.15	A	A	A	A	A	A	A	A	A	A	A	A	A	A	A	A	A	A	A	H	A	A	A	A	A	A	A	A	A		
RM4997	8	28.24	A	A	A	A	A	A	A	A	A	A	A	A	A	A	A	A	A	A	A	H	A	A	A	A	A	A	A	A	A		
RM4413	9	1.17	A	A	A	A	A	A	A	A	A	A	A	A	A	A	A	A	A	A	A	A	H	A	A	A	A	A	A	A	A		
RM2855	9	3.84	A	A	A	A	A	A	A	A	A	A	A	A	A	A	A	A	A	A	A	A	H	A	A	A	A	A	A	A	A		
RM23823	9	5.13	A	A	-	A	A	A	A	A	A	A	A	A	A	A	A	A	A	A	A	A	H	A	A	A	A	A	A	A	A		
RM23924	9	7.43	A	A	H	A	A	A	A	A	A	A	A	A	A	A	A	A	A	A	A	A	H	A	A	A	A	A	A	A	A		
RM7364	9	9.56	A	A	-	A	A	A	A	A	A	A	A	A	A	A	A	A	A	A	A	-	H	-	A	A	A	A	A	A	A		
RM7390	9	10.47	A	A	H	A	A	A	A	A	A	A	A	A	A	A	A	A	A	A	A	H	H	H	A	A	A	A	A	A	A		
RM1896	9	11.77	A	A	-	A	A	A	A	A	A	A	A	A	A	A	A	A	A	A	A	H	H	-	A	A	A	A	A	A	A		
RM409	9	14.37	A	A	H	A	A	A	A	A	A	A	A	A	A	A	A	A	A	A	A	H	-	H	A	A	A	A	A	A	A		
RM1189	9	16.27	A	A	H	A	A	A	A	A	A	A	A	A	A	A	A	A	A	A	A	H	A	H	A	A	A	A	A	A	A		1/3 (red)
RM278	9	19.03	A	A	A	A	A	A	A	A	A	A	A	A	A	A	A	A	A	A	A	A	A	H	A	A	A	A	A	A	A		
RM2482	9	22.28	A	A	A	A	A	A	A	A	A	A	A	A	A	A	A	A	A	A	A	A	A	H	A	A	A	A	A	A	A		1/1 (red)
RM6370	10	0.35	A	A	A	A	A	A	A	A	A	A	A	A	A	A	A	A	A	A	A	A	A	A	H	A	A	A	A	A	A	1/1 (red)	
RM7545	10	3.81	A	A	A	A	A	A	A	A	A	A	A	A	A	A	A	A	A	A	A	A	A	A	H	H	A	A	A	A	A		
RM6271	10	4.78	A	A	A	A	A	A	A	A	A	A	A	A	A	A	A	A	A	A	A	A	A	A	H	H	A	A	A	A	A		
RM25074	10	4.87	A	A	A	A	A	A	A	A	A	A	A	-	A	A	A	A	A	A	A	A	A	A	-	H	A	A	A	A	A		
RM2504	10	7.60	A	A	A	A	A	A	A	A	A	A	A	H	A	A	A	A	A	A	A	A	A	A	H	H	A	A	A	A	A		
RM25188	10	9.18	A	A	A	A	A	A	A	A	A	A	A	-	A	A	A	A	A	A	A	A	A	A	H	H	A	A	A	A	A		
ID11-43	10	11.14	A	A	A	A	A	A	A	A	A	A	A	H	A	A	A	A	A	A	A	A	A	A	H	H	A	A	A	A	A		1/3 (blue)
RM25302	10	12.03	A	A	A	A	A	A	A	A	A	A	A	-	A	A	A	A	A	A	A	A	A	A	H	H	A	A	A	A	A		
RM5689	10	13.55	A	A	A	A	A	A	A	A	A	A	A	A	A	A	A	A	A	A	A	A	A	A	H	H	A	A	A	A	A		
RM1937	10	18.00	A	A	A	A	-	A	A	A	A	A	A	A	A	A	A	A	A	A	A	A	A	A	A	H	A	A	A	A	A		
RM2371	10	19.65	A	A	A	A	H	A	A	A	A	A	A	A	A	A	A	A	A	A	A	A	A	A	A	H	A	A	A	A	A		
RM4771	10	23.09	A	A	A	A	A	A	A	A	A	A	A	A	A	A	A	A	A	A	A	A	A	A	A	H	A	A	A	A	A		
RM6327	11	0.37	A	A	A	A	A	A	A	A	A	A	A	A	A	A	A	A	A	A	A	A	A	A	A	A	H	A	A	A	A		
RM2459	11	2.41	A	A	A	A	A	A	A	A	A	A	A	A		A	A	A	A	A	A	A	A	A	A	A	H	A	A	A	A		
ID07-43	11	4.02	A	A	A	A	A	A	A	A	A	A	A	A	A	A	A	A	A	A	A	A	A	A	A	A	A	H	A	A	A		
RM3625	11	6.67	A	A	A	A	A	A	A	A	A	A	A	A	A	A	A	A	A	A	A	A	A	A	A	A	A	H	A	A	A		
RM26341	11	7.73	A	A	A	A	A	A	A	A	A	A	A	A	A	A	A	A	A	A	A	A	A	A	A	A	A	H	A	A	A		
RM3701	11	8.11	A	A	A	A	A	A	A	A	A	A	A	A	A	A	A	A	A	A	A	A	A	A	A	A	A	H	A	A	A		
RM5590	11	11.69	A	A	A	A	A	A	A	A	A	A	A	A	A	A	A	A	A	A	A	A	A	A	A	A	A	H	A	A	A		
RM5824	11	14.53	A	A	A	A	A	A	A	A	A	A	A	A	A	A	A	A	A	A	A	A	A	A	A	A	A	H	A	A	A		
RM209	11	18.27	A	A	A	A	A	A	A	A	A	A	A	A	A	A	A	A	A	A	A	A	A	A	A	A	A	H	A	A	A		
RM4601	11	23.11	A	A	A	A	A	A	A	A	A	A	A	A	A	A	A	A	A	A	A	A	A	A	A	A	A	H	A	A	A		
RM144	11	28.80	A	A	A	A	A	A	A	A	A	A	A	A	A	A	A	A	A	A	A	A	A	A	A	A	A	A	H	A	A		
RM27413	12	0.40	A	A	A	A	A	A	A	A	A	A	A	A	A	A	A	A	A	A	A	A	A	A	A	A	A	A	A	H	A		
ID12-20	12	0.38	A	A	A	A	A	A	A	A	A	A	A	A	A	A	A	A	A	A	A	A	A	A	A	A	A	A	A	H	A		
RM27724	12	5.51	A	A	A	A	A	A	A	A	A	A	A	A	A	A	A	A	A	A	A	A	A	A	A	A	A	A	A	H	A		
RM6905	12	6.75	A	A	A	A	A	A	A	A	A	A	A	A	A	A	A	A	A	A	A	A	A	A	A	A	A	A	A	H	A		
ID12-44	12	8.80	A	A	A	A	A	A	A	A	A	A	A	A	A	A	A	A	A	A	A	A	A	A	A	A	A	A	A	H	A		
RM27920	12	10.14	A	A	A	A	A	A	A	A	A	A	A	A	A	A	A	A	A	A	A	A	A	A	A	A	A	A	A	H	A		
RM7102	12	13.21	A	A	A	A	A	A	A	A	A	A	A	A	A	A	A	A	A	A	A	A	A	A	A	A	A	A	A	H	A		
RM28068	12	14.74	A	A	A	A	A	A	A	A	A	A	A	A	A	A	A	A	A	A	-	A	A	A	A	A	A	A	A	H	A		
ID13-02	12	17.01	A	A	A	A	A	A	A	A	A	A	A	A	A	A	A	A	A	A	H	A	A	A	A	A	A	A	A	H	H		
RM1246	12	19.12	A	A	A	A	A	A	A	A	A	A	A	A	A	A	A	A	A	A	-	A	A	A	A	A	A	A	A	H	H		
RM1986	12	21.25	A	A	A	A	A	A	A	A	A	A	A	A	A	-	-	A	A	A	A	A	A	A	A	A	A	A	A	-	-		
RM6693	12	23.80	A	A	A	A	A	A	A	A	A	A	A	A	A	A	A	A	A	A	A	A	A	A	A	A	A	A	A	A	H		
RM28585	12	24.42	A	A	A	A	A	A	A	A	A	A	A	A	A	A	A	A	A	A	A	A	A	A	A	A	A	A	A	A	H		1/1 (blue)
RM1300	12	26.00	A	A	A	A	A	A	A	A	A	A	A	A	A	A	A	A	A	A	A	A	A	A	A	A	A	A	A	A	H		
RM1227	12	27.34	A	A	A	A	A	A	A	A	A	A	A	A	A	A	A	A	A	A	A	A	A	A	A	A	A	A	A	A	H		

[a]Y indicates the presence, and N the absence, of a QTL.

[b]A indicates a homozygous Koshihikari allele; H indicates a heterozygous allele.

[c]QTLs with Koshihikari alleles that decrease trait values are shown in red boxes, while those that increase trait values are shown in blue boxes.
The numbers to the right of each box indicate the number of populations in which the QTL was detected, relative to the total number of populations examined.

Supplemental Table 13. Mean grain length and grain width in 33 KNK-$BC_{3\text{-}5}F_2$ populations, along with graphical genotypes of their parental $BC_{3\text{-}5}F_1$ plants.

Line name			13-2485	13-2486	13-2487	13-2488	13-2489	13-2490	13-2492	13-2493	13-2494	13-2495	13-2496	13-2497	13-2498	13-2499	13-2500	13-2501	13-2502	13-2503	13-2504	13-2505	13-2506	13-2507	13-2508	13-2509	13-2510	13-2511	13-2512	13-2513	13-2514	13-2515	13-2516	13-2517	13-2518	Positions of QTL[c] L	W
Grain length	Mean±SD (mm)		7.15±0.33	6.97±0.31	6.92±0.32	7.04±0.35	6.86±0.33	6.92±0.32	7.25±0.35	6.87±0.35	7.02±0.37	7.07±0.34	6.91±0.33	6.99±0.34	6.88±0.32	6.85±0.33	7.02±0.34	7.05±0.34	6.88±0.35	6.90±0.33	7.05±0.34	6.91±0.34	6.97±0.32	7.11±0.33	7.22±0.40	6.98±0.32	6.99±0.33	6.85±0.33	6.92±0.32	7.03±0.31	6.83±0.32	6.98±0.33	6.98±0.33	6.90±0.32	7.16±0.35		
	QTL detection (Y/N)[a]		Y	Y	N	N	Y	Y	Y	Y	Y	Y	N	Y	N	Y	N	N	N	N	N	Y	Y	Y	Y	N	N	N	N	N	Y	N	N	N	N		
Grain width	Mean±SD (mm)		3.45±0.20	3.40±0.17	3.41±0.18	3.43±0.20	3.35±0.18	3.42±0.17	3.48±0.21	3.33±0.19	3.40±0.18	3.45±0.16	3.42±0.18	3.34±0.19	3.40±0.20	3.43±0.18	3.44±0.18	3.47±0.18	3.46±0.18	3.37±0.17	3.43±0.18	3.44±0.17	3.41±0.17	3.53±0.16	3.44±0.20	3.40±0.16	3.43±0.18	3.38±0.18	3.40±0.17	3.41±0.16	3.36±0.17	3.38±0.16	3.44±0.17	3.38±0.15	3.46±0.18		
	QTL detection (Y/N)[a]		Y	N	Y	N	Y	Y	Y	Y	N	N	Y	N	N	Y	Y	Y	Y	N	N	Y	N	Y	N	Y	N	N	Y	N	Y	Y	N	N	N		
Genotypes[b]																																				L	W
Marker	Chr. No.	Position (Mb)																																			
RM4959	1	0.46	H	A	A	A	A	A	H	A	A	A	A	A	A	A	A	A	A	A	A	A	A	H	A	A	A	A	A	A	A	A	A	A	A		
RM6902	1	3.80	H	A	A	A	A	A	H	A	A	A	A	A	A	A	A	A	A	A	A	A	A	-	A	A	A	A	A	A	A	A	A	A	A	2/2 (red)	2/2 (red)
RM5443	1	6.23	H	A	A	A	A	A	H	A	A	A	A	A	A	A	A	A	A	A	A	A	A	A	A	A	A	A	A	A	A	A	A	A	A		
RM8046	1	9.94	H	A	A	A	A	A	A	A	A	A	A	A	A	A	A	A	A	A	A	A	A	A	A	A	A	A	A	A	A	A	A	A	A		
RM10850	1	13.96	H	A	A	A	A	A	A	A	A	A	A	A	A	A	A	A	A	A	H	A	A	A	A	A	A	H	A	A	A	A	A	A	A		
RM7075	1	15.12	H	A	A	A	A	A	A	A	A	A	A	A	A	A	A	A	A	A	H	A	A	A	A	A	A	H	A	A	A	A	A	A	A		
RM5422	1	18.76	H	H	A	A	A	A	A	A	A	A	A	A	A	A	A	A	A	A	H	A	A	A	A	A	A	H	H	A	A	A	A	A	A		
RM8090	1	21.43	-	H	A	A	A	A	A	A	A	A	A	A	A	A	A	A	A	A	-	A	A	A	A	A	A	-	H	A	A	A	A	A	A		
RM7266	1	24.45	A	H	A	A	A	A	A	A	A	A	A	A	A	A	A	A	A	A	A	A	A	A	A	A	A	A	H	A	A	A	A	A	A		1/2 (blue)
RM5497	1	27.54	A	H	A	A	A	A	A	A	A	A	A	A	A	A	A	A	A	A	A	A	A	A	A	A	A	A	-	A	A	A	A	A	A		
RM3709	1	31.95	A	H	A	A	A	A	A	A	A	A	A	A	A	A	A	A	A	H	A	A	A	A	A	A	A	A	-	A	A	A	A	A	A		
RM3825	1	36.47	A	-	H	A	A	A	A	A	A	A	A	A	A	A	A	A	A	A	A	A	A	A	A	A	A	A	A	A	A	A	A	A	A		
RM1387	1	40.21	A	A	H	H	A	A	A	A	A	A	A	A	A	A	A	A	A	A	A	A	A	A	A	A	A	A	A	A	A	A	A	A	A		1/2 (red)
RM6840	1	43.17	A	A	A	H	A	A	A	A	A	A	A	A	A	A	A	A	A	A	A	A	A	A	A	A	A	A	A	A	A	A	A	A	A		
RM6938	2	0.98	A	A	A	A	H	A	A	A	A	A	A	A	A	A	A	A	A	A	A	A	A	A	A	A	A	A	A	A	A	A	A	A	A		
RM7562	2	1.88	A	A	A	A	H	A	A	H	A	A	A	A	A	A	A	A	A	A	A	A	A	A	A	A	A	A	A	A	A	A	A	A	A	2/2 (blue)	1/2 (blue)
RM4355	2	4.26	A	A	A	A	H	H	A	H	A	A	A	A	A	A	A	A	A	A	A	A	A	A	A	A	A	A	A	A	A	A	A	A	A		
RM12811	2	7.77	A	A	A	A	A	H	A	A	A	A	A	A	A	A	A	A	A	A	A	A	A	A	A	A	A	A	A	A	A	A	A	A	A		
RM3501	2	10.19	A	A	A	A	A	H	A	A	A	A	A	A	A	A	A	A	A	A	A	A	A	A	A	A	A	A	A	A	A	A	A	A	A	1/1 (blue)	
RM13106	2	13.52	A	A	A	A	A	H	A	A	A	A	A	A	A	A	A	A	A	A	A	A	A	A	A	A	A	A	A	A	A	A	A	A	A		
RM6942	2	17.44	A	A	A	A	A	H	A	A	A	A	A	A	A	A	A	A	A	A	A	A	A	A	A	A	A	A	A	A	A	A	A	A	A		
RM2634	2	20.50	A	A	A	A	A	H	H	A	A	A	A	A	A	A	A	A	A	A	A	A	A	A	A	A	A	A	A	A	A	A	A	A	A		
RM1920	2	25.47	A	A	A	A	A	A	H	A	A	A	A	A	A	A	A	A	A	A	A	A	A	A	A	A	A	A	A	A	A	A	A	A	A		
RM5631	2	28.27	A	A	A	A	A	A	H	A	A	A	A	A	A	A	A	A	A	A	A	A	A	A	A	A	A	A	A	A	A	A	A	A	A		
RM8029	2	32.49	A	A	A	A	A	A	H	A	A	A	A	A	A	A	A	A	A	A	A	A	A	A	A	A	A	A	A	A	A	A	A	A	A		
RM2265	2	35.41	A	A	A	A	A	A	H	A	A	A	A	A	A	A	A	A	H	A	A	A	A	A	A	A	A	A	A	A	A	A	A	A	A		
RM4108	3	0.54	A	A	A	A	A	H	A	H	A	A	A	A	A	A	H	A	A	A	A	A	A	A	A	A	A	A	A	A	A	A	A	-	A		1/3 (red)
RM6883	3	3.10	A	A	A	A	A	A	A	H	A	A	A	A	A	A	H	A	A	A	A	A	A	A	A	A	A	A	A	A	A	A	A	-	A		
RM5755	3	5.96	A	A	A	A	A	A	-	H	A	A	A	A	A	A	-	A	A	A	A	A	A	A	-	A	A	A	A	A	A	A	A	A	A	1/1 (red)	
RM4996	3	8.48	A	A	A	A	A	A	H	H	A	A	A	A	A	A	A	A	A	A	A	A	A	A	H	A	A	A	A	A	A	A	A	A	A		1/3 (red)
RM5178	3	10.66	A	A	A	A	A	A	A	-	H	A	A	A	A	A	A	A	A	A	A	A	A	A	H	A	A	A	A	A	A	A	A	A	A		
RM5489	3	13.31	A	A	A	A	A	A	A	A	H	A	A	A	A	A	A	A	A	A	A	A	A	A	H	A	A	A	A	A	A	A	A	A	A		
GS3 UD00	3	16.73	A	A	A	A	A	A	A	A	H	A	A	A	A	A	A	A	A	A	A	A	A	A	H	A	A	A	A	A	A	A	A	A	A	2/2 (red)	1/2 (red)
RM6914	3	17.29	A	A	A	A	A	A	A	A	H	A	A	A	A	A	A	A	A	A	A	A	A	A	H	A	A	A	A	A	A	A	A	A	A		
RM5488	3	21.39	A	A	A	A	A	A	A	A	H	A	A	A	A	A	A	A	A	A	A	A	A	A	H	A	A	A	A	A	A	A	A	A	A		
RM3513	3	25.11	A	A	A	A	A	A	A	A	H	H	A	A	A	A	A	A	A	A	A	A	A	A	H	A	A	A	A	A	A	A	A	A	H		
RM1350	3	28.68	A	A	A	A	A	A	H	A	A	H	A	A	A	A	A	A	A	A	A	A	A	A	A	A	A	A	A	A	A	A	A	A	A		
RM6970	3	32.44	A	A	A	A	A	A	H	A	A	H	A	A	A	A	A	A	A	A	A	A	A	A	A	A	A	A	A	A	A	A	A	A	A	1/2 (red)	
RM16254	4	0.11	A	A	A	A	A	A	A	A	A	A	H	A	A	A	A	A	A	A	A	A	A	A	A	A	A	A	A	A	A	A	A	A	A		
RM16367	4	2.41	A	A	A	A	A	A	A	A	A	A	H	A	A	A	A	A	A	A	A	A	A	A	A	A	A	A	A	A	A	A	A	A	A		1/1 (red)
RM16449	4	4.91	A	A	A	A	A	A	A	A	A	A	H	A	A	A	A	A	A	A	A	A	A	A	A	A	A	A	A	A	A	A	A	A	A		
RM16534	4	8.04	A	A	A	A	A	A	A	A	A	A	H	A	A	A	A	A	A	A	A	-	A	A	A	A	A	A	A	A	A	A	A	A	A		
RM7279	4	13.77	A	A	A	A	A	A	A	A	A	A	H	H	A	A	A	A	A	A	-	A	A	A	A	A	A	A	A	A	A	A	A	A	A		
RM6679	4	19.90	A	A	A	A	A	A	A	A	A	A	A	H	A	A	A	A	A	A	A	A	A	A	-	H	A	A	A	A	A	A	A	A	A		
RM3524	4	22.89	A	A	A	A	A	A	A	A	A	A	A	H	A	A	A	A	A	A	A	A	A	A	A	H	A	A	A	A	A	A	A	A	A	1/2 (red)	
RM5714	4	27.47	A	-	A	A	A	A	A	A	A	A	A	H	-	A	A	A	A	A	A	A	A	A	A	A	A	A	A	A	A	A	A	A	A		
RM5511	4	31.11	A	A	A	A	A	A	A	A	A	A	A	A	H	A	A	A	A	A	A	A	A	A	A	A	A	A	A	A	A	A	A	A	A		
RM2431	4	35.10	A	-	-	A	A	-	A	A	A	A	A	A	H	A	A	A	A	A	A	H	A	A	A	A	A	A	A	A	A	A	A	A	A		
RM17726	5	0.17	A	A	A	A	A	A	A	A	A	A	A	A	A	H	A	A	A	A	A	A	A	A	A	A	A	A	A	A	A	A	A	A	A	1/1 (blue)	
RM17867	5	2.22	A	A	A	A	A	A	A	A	A	A	A	A	A	H	A	A	A	A	A	A	A	A	A	A	A	A	A	A	A	A	A	A	A		
RM18055	5	5.88	A	A	A	A	A	A	A	A	A	A	A	A	A	H	A	A	A	A	A	A	A	A	A	A	A	A	A	A	A	A	A	A	A		1/1 (red)
RM18194	5	9.35	A	A	A	A	A	A	A	A	A	A	A	A	A	H	H	A	A	A	A	A	A	A	A	A	A	A	A	A	A	A	A	A	A		
RM8039	5	13.43	A	A	A	A	A	A	A	A	A	A	A	A	A	H	H	A	A	A	A	A	A	A	A	A	A	A	A	A	A	A	A	A	A		
RM18465	5	16.62	A	A	A	A	A	A	A	A	A	A	A	A	A	-	H	A	A	A	A	A	A	A	A	A	A	A	A	A	A	A	A	A	A		
RM1386	5	20.06	A	A	A	A	A	A	A	A	A	A	A	A	A	A	H	H	A	A	A	A	A	A	A	A	A	A	A	A	A	A	A	A	A		
RM3476	5	23.91	A	A	A	A	A	A	A	A	A	A	A	A	A	A	A	H	A	A	A	A	A	A	A	A	A	A	A	A	A	A	A	A	A		
RM19062	5	27.30	A	A	A	A	A	A	A	A	A	A	A	A	A	A	A	H	A	A	A	A	A	A	A	A	A	A	A	A	A	A	A	A	A		1/1 (red)
RM5907	5	29.61	A	A	A	A	A	A	A	A	A	A	A	A	A	A	A	H	A	A	A	A	A	A	A	A	A	A	A	A	A	A	A	A	A		
RM8125	6	3.17	A	A	A	A	A	A	A	A	A	A	A	A	A	A	A	A	H	A	A	A	A	A	A	A	A	A	A	A	A	A	A	-	A		
RM2126	6	5.91	A	A	A	A	A	A	A	A	A	A	A	A	A	A	A	A	H	H	A	A	A	A	A	A	A	A	A	A	A	A	A	A	A		
RM2523	6	8.92	A	A	A	A	A	A	A	A	A	A	A	A	A	A	A	A	H	H	A	A	A	A	A	A	A	A	A	A	A	A	A	A	A		
RM19890	6	11.95	A	A	A	A	A	A	A	A	A	A	A	A	A	A	A	A	A	H	A	A	A	A	A	A	A	A	A	A	A	A	A	A	A		
RM20045	6	16.05	H	A	A	A	A	A	A	A	A	A	A	A	A	A	A	A	H	H	A	A	A	A	A	A	A	A	A	A	A	A	A	A	A		
RM7193	6	20.26	A	A	A	A	A	A	A	A	A	A	A	A	A	A	A	A	A	H	H	A	A	A	A	A	A	A	A	A	A	A	A	A	A		
RM1340	6	23.34	A	A	A	A	A	A	A	A	A	A	A	A	A	A	A	A	A	H	H	A	A	A	A	-	A	A	A	A	A	A	A	A	A		
RM5509	6	27.83	A	A	A	A	A	A	A	A	A	A	A	A	A	A	A	A	A	A	H	A	A	A	A	A	A	A	A	A	A	A	A	A	A		
RM5753	6	30.97	A	A	A	A	A	A	A	A	A	A	A	A	A	A	A	A	A	A	H	A	A	A	A	A	A	A	A	A	A	A	A	A	A		
RM3394	7	0.65	A	A	A	A	A	A	A	A	A	A	A	A	A	A	A	A	A	A	A	H	A	A	A	A	A	A	A	A	A	A	A	A	A	1/1 (blue)	
RM2006	7	4.20	A	A	A	A	A	A	A	A	A	A	A	A	A	A	A	A	A	A	A	H	A	A	A	A	A	A	A	A	A	A	A	A	A		
RM6776	7	6.84	A	A	A	A	A	A	A	A	A	A	A	A	A	A	A	A	A	A	A	H	A	A	A	A	A	A	A	A	A	A	A	A	A		
RM21324	7	8.84	A	A	A	A	A	A	A	A	A	A	A	A	A	A	A	H	A	A	A	-	A	A	A	A	A	A	A	A	A	A	A	A	A		
RM5499	7	9.99	A	A	A	A	A	A	A	A	A	A	A	A	A	A	A	A	A	A	A	H	A	A	A	A	A	A	A	A	A	A	A	A	A		
RM3832	7	12.71	A	A	A	A	A	A	A	A	A	A	-	A	A	A	A	A	A	A	A	H	A	A	A	A	A	A	A	A	A	A	A	A	A		
RM21560	7	16.99	A	A	A	A	A	A	A	A	A	A	H	A	A	A	A	A	A	A	H	H	A	A	A	A	A	A	A	A	A	A	A	A	A		
RM3404	7	20.11	A	A	A	A	A	A	A	A	A	A	H	A	A	A	A	A	A	A	H	A	H	A	A	A	A	A	A	A	A	A	A	A	A		
RM5847	7	23.65	A	A	A	A	A	A	A	A	A	A	A	A	A	A	A	A	A	A	A	A	H	A	A	A	A	A	A	A	A	A	A	A	A	1/1 (blue)	
RM1364	7	26.78	A	A	A	A	A	A	A	A	A	A	A	A	A	A	A	A	H	A	A	A	H	H	A	A	A	A	A	A	A	A	A	A	A	1/3 (red)	
RM1306	7	28.95	A	A	A	A	A	A	A	A	A	A	A	A	A	A	A	A	A	A	A	A	A	H	A	A	A	A	A	A	A	A	A	A	A		1/1 (red)
RM7034	8	0.15	A	A	H	H	A	H	A	A	A	A	A	A	A	A	A	A	A	A	A	A	A	A	H	A	A	H	H	A	A	A	A	A	A		
RM3819	8	3.02	A	A	A	A	A	H	A	A	A	A	A	A	A	A	A	A	A	A	A	A	A	A	H	A	A	A	A	A	A	A	A	A	A		
RM2584	8	7.57	A	A	A	A	A	H	A	A	A	A	A	A	A	A	A	A	A	A	A	A	A	A	A	-	A	A	A	A	A	A	A	A	A		1/1 (red)
RM5514	8	10.41	A	A	A	A	A	H	A	A	A	A	A	A	A	A	A	A	A	A	A	A	A	A	A	H	A	A	A	A	A	A	A	A	A		
RM22837	8	12.38	A	A	A	A	A	H	A	A	A	A	A	A	A	A	A	A	A	A	A	A	A	A	A	H	A	A	A	A	A	A	A	A	A		
RM22965	8	16.64	A	A	A	A	A	H	A	A	A	A	A	A	A	A	A	A	A	A	A	A	A	A	A	H	A	A	A	A	A	A	A	A	A		
RM3459	8	20.65	A	A	A	A	A	-	A	A	A	A	A	A	A	-	A	A	A	A	A	A	A	A	A	H	-	A	A	A	A	A	A	A	A		
RM3452	8	24.78	A	A	A	A	A	A	A	A	A	A	A	A	A	H	A	A	A	A	A	A	A	A	A	H	H	A	A	A	A	A	A	A	A		
RM4997	8	28.24	A	A	A	A	A	A	A	A	A	H	A	A	A	A	A	A	A	A	A	A	A	A	A	A	H	A	A	A	A	A	A	A	A		
RM23668	9	0.65	A	H	A	H	A	A	A	A	A	A	A	A	A	A	A	A	A	A	A	H	A	A	A	A	H	H	A	A	A	A	A	A	A		
RM23766	9	3.64	A	H	A	H	A	A	A	A	A	-	A	A	A	A	A	A	A	A	A	H	A	A	A	A	-	H	A	A	A	A	A	A	A	1/4 (blue)	1/4 (red)
RM7212	9	6.60	A	H	A	H	A	A	A	A	A	A	A	A	A	A	A	A	A	A	A	H	A	A	A	A	A	H	A	A	A	A	A	A	A		
RM3907	9	10.49	A	H	A	H	A	A	A	A	A	A	A	A	A	A	A	A	A	A	A	H	A	A	A	A	A	H	A	A	A	A	A	A	A		
RM5652	9	14.79	A	A	A	A	A	A	A	A	A	A	A	A	A	A	A	A	A	A	A	A	A	A	A	A	A	A	H	A	A	A	A	A	A		
RM7289	9	17.26	A	A	A	-	A	A	A	A	A	A	A	A	A	A	A	A	A	A	A	A	A	A	A	A	A	A	H	A	A	A	A	A	A		
RM3909	9	19.54	A	A	A	H	A	A	A	A	A	A	A	A	A	A	A	A	A	A	A	A	A	A	A	A	A	A	H	A	A	A	A	A	A		
RM5384	9	22.07	A	A	A	H	A	A	A	A	A	A	A	A	A	A	A	A	A	A	A	A	A	A	A	A	A	A	H	A	A	A	A	A	A		1/2 (blue)
RM5095	10	0.05	A	A	A	A	A	A	A	A	A	A	A	A	A	A	A	A	H	A	A	A	A	A	A	A	A	A	A	H	A	A	A	A	A		
RM25031	10	3.66	A	A	A	A	A	A	A	A	A	A	A	A	A	A	A	A	-	A	A	A	A	A	A	A	A	A	A	H	A	A	A	A	A		
RM25158	10	7.94	A	A	A	A	A	A	A	A	A	A	A	A	A	A	A	A	A	A	A	A	A	A	A	A	A	A	A	H	A	A	A	A	A		
RM25261	10	10.93	A	A	A	A	A	A	A	A	A	A	A	A	A	A	A	A	A	A	A	A	A	A	A	A	A	A	A	H	A	A	A	A	A		
RM5708	10	14.53	A	A	A	A	A	A	A	A	A	A	A	A	A	A	A	A	A	A	A	A	A	A	A	A	A	A	A	H	A	A	A	A	A		
RM3773	10	19.97	A	A	A	A	A	A	A	H	A	A	A	A	A	A	A	A	A	A	A	A	A	A	A	H	A	A	A	H	H	A	A	A	A	1/4 (blue)	2/4 (blue)
RM4771	10	23.09	A	A	A	A	A	A	A	H	A	A	A	A	A	A	A	A	A	A	A	A	A	A	H	A	A	A	A	A	H	A	A	A	A		
RM1761	11	0.31	A	A	A	A	A	A	A	A	A	A	A	A	A	A	A	A	A	A	A	A	A	A	A	A	A	A	A	A	A	H	A	A	A		
RM1812	11	2.41	A	A	A	A	A	A	A	A	A	A	A	A	A	A	A	A	A	A	A	A	A	A	A	A	A	A	A	A	A	H	A	A	A		1/1 (blue)
RM5704	11	5.48	A	A	A	A	A	A	A	A	A	A	A	A	A	A	A	A	A	A	A	A	A	A	A	A	A	A	A	A	A	H	A	A	A		
RM5824	11	14.53	A	A	A	A	A	A	A	A	A	A	A	A	A	A	A	A	A	A	A	A	A	A	A	A	A	A	A	A	A	H	H	A	A		
RM5312	11	17.63	A	A	A	A	A	A	A	A	A	A	A	A	A	A	A	A	A	A	A	A	A	A	A	A	A	A	A	A	A	-	H	A	A		
RM6534	11	21.52	A	A	A	A	A	A	A	A	A	A	A	A	A	A	A	A	A	A	A	A	A	A	A	A	A	A	A	A	A	A	H	A	A		
RM2191	11	25.13	A	A	A	A	A	A	A	A	A	A	A	A	A	A	A	A	A	A	A	A	A	A	A	A	A	A	A	A	A	A	H	A	A		
RM2136	11	28.93	A	A	A	A	A	A	A	A	A	A	A	A	A	A	A	A	A	A	A	A	A	A	A	A	A	A	A	A	A	A	H	A	A		
RM27414	12	0.40	A	A	A	A	A	A	A	A	A	A	A	A	H	A	A	A	A	A	A	A	H	A	H	A	A	A	A	A	A	A	A	A	A		
RM5927	12	2.20	A	A	A	A	A	A	A	A	A	A	A	A	H	A	A	A	A	A	A	A	H	A	A	A	A	A	A	A	A	A	A	H	A		
RM5317	12	5.04	A	A	A	A	A	A	A	A	A	A	A	A	A	A	A	A	A	A	A	A	A	A	A	A	A	A	A	A	A	A	A	H	A		
RM27900	12	9.57	A	A	A	A	A	A	A	A	A	A	A	A	A	A	A	A	A	A	A	A	A	A	A	A	A	A	A	A	A	A	A	H	A		
RM28037	12	13.74	A	A	A	A	A	A	A	A	A	A	A	A	A	A	A	A	A	A	A	A	A	A	A	A	A	A	A	A	A	A	A	H	A		
RM28166	12	17.61	A	A	A	A	A	A	A	A	A	A	A	A	A	A	A	A	A	A	A	A	A	A	A	A	A	A	A	A	A	A	A	H	A		
RM1986	12	21.25	A	A	A	A	A	A	A	A	A	A	A	A	A	A	A	A	A	A	A	A	A	A	A	A	A	A	A	A	A	A	A	H	H		
RM5715	12	25.03	A	A	A	A	A	A	A	A	A	A	A	A	A	A	A	A	A	A	A	A	A	A	A	A	H	A	A	A	A	A	A	H	H		
RM1227	12	27.34	A	A	A	A	A	A	A	A	A	H	A	H	A	A	A	A	H	A	A	A	A	A	A	A	A	A	A	A	A	A	H	A	H		

[a]Y indicates the presence, and N the absence, of a QTL.

[b]A indicates a homozygous Koshihikari allele; H indicates a heterozygous allele.

[c]QTLs with Koshihikari alleles that decrease trait values are shown in red boxes, while those that increase trait values are shown in blue boxes.
The numbers to the right of each box indicate the number of populations in which the QTL was detected, relative to the total number of populations examined.

Supplemental Table 14. Mean grain length and grain width in 35 KMK-BC$_{3-5}$F$_2$ populations, along with graphical genotypes of their parental BC$_{3-5}$F$_1$ plants.

Line name			13-2523	13-2524	13-2525	13-2526	13-2527	13-2528	13-2529	13-2530	13-2531	13-2532	13-2533	13-2534	13-2535	13-2537	13-2538	13-2539	13-2540	13-2541	13-2542	13-2543	13-2544	13-2545	13-2546	13-2547	13-2548	13-2549	13-2550	13-2551	13-2552	13-2553	13-2554	13-2555	13-2556	13-2557	13-2558	Positions of QTL[c] L	W
Grain length	Mean±SD (mm)		6.82±0.31	7.16±0.34	7.00±0.32	6.98±0.30	6.97±0.33	6.88±0.33	7.06±0.32	7.02±0.32	7.29±0.41	6.94±0.35	6.92±0.36	6.88±0.34	7.08±0.35	6.97±0.34	6.98±0.37	6.86±0.35	6.90±0.34	7.19±0.35	6.93±0.33	7.02±0.34	6.91±0.35	6.98±0.34	7.07±0.37	6.90±0.34	7.11±0.34	7.03±0.32	7.00±0.33	7.00±0.34	6.88±0.34	6.90±0.33	6.98±0.32	6.84±0.33	6.99±0.35	7.04±0.38	6.95±0.36		
	QTL detection (Y/N)[a]		Y	Y	N	Y	Y	Y	Y	N	Y	Y	Y	Y	N	N	Y	N	Y	Y	N	Y	Y	N	Y	N	N	Y	N	N	N	Y	N	Y	Y	N	N		
Grain width	Mean±SD (mm)		3.36±0.16	3.56±0.20	3.43±0.15	3.38±0.16	3.41±0.17	3.33±0.16	3.37±0.17	3.44±0.17	3.38±0.22	3.37±0.23	3.31±0.24	3.34±0.22	3.28±0.21	3.33±0.18	3.41±0.21	3.37±0.20	3.37±0.20	3.42±0.17	3.42±0.16	3.48±0.17	3.39±0.19	3.42±0.22	3.35±0.23	3.38±0.22	3.35±0.18	3.45±0.16	3.39±0.16	3.42±0.17	3.36±0.16	3.39±0.16	3.44±0.15	3.42±0.17	3.51±0.19	3.41±0.21	3.38±0.21		
	QTL detection (Y/N)[a]		Y	Y	N	N	N	Y	N	Y	Y	Y	N	Y	N	N	Y	N	Y	Y	N	Y	Y	Y	N	Y	N	Y	N	N	N	N	Y	N	N	Y	Y		
Genotypes[b]																																							
Marker	Chr. No.	Position (Mb)																																				L	W
RM6842	1	0.12	H	A	A	A	A	-	A	A	A	A	A	A	A	A	A	A	A	A	A	A	A	A	A	A	A	A	A	A	A	A	A	A	A	A	A		
RM1329	1	3.95	A	H	A	A	A	A	A	A	A	A	A	A	A	A	A	A	A	A	A	A	A	A	A	A	A	A	A	A	A	A	A	A	A	A	A	1/1 (red)	1/1 (red)
RM8142	1	7.46	A	H	A	A	A	A	A	A	A	A	A	A	A	A	A	A	A	A	A	A	A	A	A	A	A	A	A	A	A	A	A	A	A	A	A		
RM8046	1	9.94	A	H	H	A	A	A	A	A	A	A	A	A	A	A	H	A	A	A	A	A	A	A	A	A	A	A	A	A	A	A	A	A	A	A	A		1/3 (red)
RM8095	1	11.24	A	-	H	A	A	A	A	A	A	A	A	A	A	A	-	A	-	A	A	A	A	A	A	A	A	A	A	A	A	A	A	A	A	A	A		
RM7075	1	15.12	A	A	H	A	A	A	A	A	A	A	A	A	A	A	A	A	-	A	A	A	A	A	A	A	A	A	A	A	A	A	A	A	A	A	A		
RM6092	1	19.12	A	A	H	A	A	A	A	A	A	A	A	A	A	A	A	A	A	A	A	A	A	A	A	A	A	A	A	A	A	A	A	A	A	A	A		
RM5718	1	22.93	A	A	H	A	A	A	A	A	A	A	A	A	A	A	A	A	A	A	A	A	A	A	A	A	A	A	A	A	A	A	A	A	A	A	A		
RM5461	1	26.91	A	A	H	H	A	A	A	A	A	A	A	A	A	A	A	A	A	A	A	A	A	A	A	A	A	A	A	A	A	A	A	A	A	A	A		
RM8126	1	29.28	A	A	H	H	A	A	A	A	A	A	A	A	A	A	A	A	A	A	A	A	A	A	A	A	A	A	A	A	A	A	A	A	A	A	A		
RM3416	1	32.89	A	A	A	H	A	A	A	A	A	A	A	A	A	A	A	A	A	A	A	A	A	A	A	A	A	A	A	A	A	A	A	A	A	A	A	1/1 (red)	
RM3403	1	35.00	A	A	A	H	A	A	A	A	A	A	A	A	A	A	A	A	A	A	A	A	A	A	A	A	A	A	A	A	A	A	A	A	A	A	A		
RM6696	1	38.22	A	A	A	H	H	A	A	A	A	A	A	A	A	A	A	A	A	A	A	A	A	A	A	A	A	A	A	A	A	A	A	A	A	A	A	2/2 (blue)	
RM1387	1	40.21	A	A	A	-	H	A	A	A	A	A	A	A	A	A	A	A	A	H	A	A	A	A	A	A	A	A	A	A	A	A	A	A	A	A	A		
RM6840	1	43.17	A	A	A	A	H	A	A	A	A	A	A	A	A	A	A	A	H	H	A	A	A	A	A	A	A	A	A	A	A	A	A	A	H	H	A		
RM6842	2	0.12	-	-	-	-	-	H	-	-	-	-	-	-	-	-	-	-	-	-	-	-	-	-	-	-	-	-	-	-	-	-	-	-	-	-	-		
RM3703	2	3.86	H	A	A	A	A	H	A	A	A	A	A	A	A	A	A	A	A	A	A	A	A	A	A	A	A	A	A	A	A	A	A	A	A	A	A	1/2 (blue)	
RM5340	2	7.48	H	A	A	A	-	H	A	A	A	A	A	-	-	A	A	A	A	A	A	A	A	A	A	A	A	A	A	A	A	A	A	A	A	A	A		
RM1358	2	10.19	H	A	A	A	H	H	A	A	A	A	A	H	H	A	A	A	A	A	A	A	A	A	A	A	A	A	A	A	A	A	A	A	A	A	A		2/5 (blue)
RM6374	2	15.19	H	A	A	A	A	H	A	A	A	A	A	H	H	A	A	A	A	A	A	A	A	A	A	A	A	A	A	A	A	A	A	A	A	A	A		
RM3858	2	19.19	A	A	A	A	A	H	A	A	A	A	A	A	A	A	A	A	A	A	A	A	A	A	A	A	A	A	A	A	A	A	H	H	A	A	A		
RM5430	2	22.80	A	A	A	A	A	H	A	A	A	A	A	A	A	A	A	A	A	A	A	A	A	A	A	A	A	A	A	A	A	A	-	-	A	A	A		
RM3515	2	24.02	A	A	A	A	A	H	H	A	A	A	A	A	A	A	A	A	A	A	A	A	A	A	A	A	A	A	A	A	A	A	A	A	A	A	A		
RM3730	2	26.24	A	A	A	A	A	-	-	A	A	A	A	A	A	A	A	A	A	A	A	A	A	A	A	A	A	H	A	A	A	A	A	A	A	A	A		
RM3512	2	27.32	A	A	A	A	A	A	H	A	A	A	A	A	A	A	A	A	A	A	A	A	A	A	A	A	A	H	A	A	A	A	A	A	A	A	A		
RM5472	2	30.64	A	A	A	A	A	A	H	A	A	A	A	A	A	A	A	A	A	A	A	H	A	A	A	A	A	H	A	A	A	A	A	A	A	A	A		
RM6636	2	33.08	A	A	A	A	A	A	H	A	A	A	A	A	A	A	A	A	A	A	A	H	A	A	A	A	A	H	A	A	A	A	A	A	A	A	A	2/3 (red)	
RM3789	2	34.69	-	A	A	A	A	A		H	A	A	-	A	A	A	A	A	A	A	A	A	A	A	-	A	A	A	A	A	A	A	A	A	A	A	A		
RM6923	2	35.70	-	A	A	A	A	A		H	A	A	H	A	A	A	A	A	A	A	A	A	A	A	H	A	A	A	A	A	A	A	A	A	A	A	A		
RM7332	3	0.41	A	A	A	A	A	A	A	H	A	A	A	A	H	A	A	A	A	A	A	A	A	A	A	A	A	A	A	A	A	A	A	A	A	A	A		
RM3392	3	3.83	A	A	A	A	A	A	A	H	A	A	A	A	A	A	A	A	A	A	A	A	A	A	A	A	A	A	A	A	A	A	A	A	A	A	A		
RM5477	3	6.55	-	A	A	A	A	A	A	H	A	A	A	A	A	A	A	A	-	A	A	A	A	A	A	A	A	A	A	A	A	A	A	A	A	A	A		
RM1371	3	9.94	H	A	A	A	A	A	A	H	H	A	A	A	A	A	A	A	H	A	A	A	A	A	A	A	A	A	A	A	A	A	A	A	A	A	A		
RM5551	3	12.93	-	A	A	A	A	A	A	A	H	A	A	A	A	A	A	A	-	A	A	A	A	A	A	A	A	A	A	A	A	A	A	A	A	A	A		
RM2917	3	14.39	A	A	A	A	A	A	A	A	H	A	A	A	A	A	A	A	A	A	A	A	A	A	A	A	A	A	A	A	A	A	A	A	A	A	A		
GS3 UD00	3	16.73	A	A	A	A	A	A	A	A	H	A	A	A	A	A	A	A	A	A	A	A	A	A	A	A	A	A	A	A	A	A	A	A	A	A	A	1/1 (red)	
RM1940	3	18.16	A	A	A	A	A	A	A	A	H	A	A	A	A	A	A	A	A	A	A	A	A	A	A	A	A	A	A	A	A	A	A	A	A	A	A		
RM3698	3	21.78	A	A	A	A	A	A	A	A	H	A	A	A	A	A	A	A	A	A	A	A	A	A	A	A	A	A	A	A	A	A	A	A	A	A	A		1/1 (blue)
RM3513	3	25.11	A	A	A	A	A	A	A	A	H	H	A	A	A	A	A	A	A	A	A	A	A	A	A	A	A	A	A	A	A	A	A	A	A	A	A		
RM3856	3	28.78	A	A	A	A	A	A	A	A	A	H	A	A	A	A	A	A	A	A	A	A	A	A	A	A	A	A	A	A	A	A	A	A	A	A	A	1/1 (red)	1/1 (red)
RM6970	3	32.44	A	A	A	A	A	A	A	A	A	H	H	A	A	A	A	A	A	A	A	A	A	A	A	A	A	A	A	A	A	A	A	A	A	A	A		
RM7389	3	36.16	A	A	A	A	A	A	A	A	A	A	H	A	A	A	A	A	A	A	A	A	A	A	A	A	A	A	A	A	A	A	A	A	A	A	A		
RM16254	4	0.11	A	A	A	A	A	A	A	A	A	A	A	H	A	A	A	A	A	H	A	A	A	A	A	A	A	A	A	A	A	A	A	A	A	A	A		
RM16367	4	2.41	A	A	A	A	A	A	A	A	A	A	A	H	A	A	A	A	A	A	A	A	A	A	A	A	A	A	A	A	A	A	A	A	A	A	A		
RM7472	4	7.10	A	A	A	A	A	A	A	A	A	A	A	H	A	A	A	A	A	A	A	A	A	A	A	A	A	A	A	A	A	A	A	A	A	A	A		
RM5953	4	9.39	A	A	A	A	A	A	A	A	A	A	A	H	A	A	A	A	A	A	A	A	A	A	A	A	A	A	A	A	A	A	A	A	A	A	A		
RM5893	4	13.77	A	A	A	A	A	A	A	A	A	A	A	H	A	A	A	A	A	A	A	A	A	A	A	A	A	A	A	A	A	A	A	A	A	A	A	1/1 (blue)	
RM16791	4	18.12	A	A	A	A	A	A	A	A	A	A	A	H	A	A	A	A	A	A	A	A	A	A	A	A	A	A	A	A	A	A	A	A	A	A	A		1/1 (blue)
RM1359	4	20.03	A	A	A	A	A	A	A	A	A	A	A	H	H	A	A	A	A	A	A	A	A	A	A	A	A	A	A	A	A	A	A	A	A	A	A		
RM3042	4	23.02	A	A	A	A	A	A	A	A	A	A	A	H	H	A	A	A	A	A	A	A	A	A	A	A	A	A	A	A	A	A	A	A	A	A	A		
RM1272	4	35.30	A	A	A	A	A	A	A	A	A	A	A	A	A	A	A	A	A	A	A	A	A	H	A	A	A	A	A	A	A	A	A	A	H	A	A		
RM5693	5	0.46	A	A	A	A	A	A	A	A	A	A	A	A	A	H	A	A	A	A	A	A	A	A	A	A	A	A	A	A	A	A	A	A	A	A	A		
RM2010	5	1.18	-	A	A	A	A	A	A	A	A	A	A	A	A	H	A	A	A	A	A	A	A	A	A	A	A	A	A	A	A	A	A	A	A	A	A		
RM5541	5	5.21	A	A	A	A	A	A	A	A	A	A	A	A	A	H	H	A	A	A	A	A	A	A	A	A	A	A	A	A	A	A	A	A	A	A	A		
RM2676	5	13.54	A	A	A	A	A	A	A	A	A	A	A	A	A	A	H	A	A	A	A	A	A	A	A	A	A	A	A	A	A	A	A	A	A	A	A		
RM18465	5	16.62	A	A	A	A	A	A	A	A	A	A	A	A	A	A	H	A	A	A	A	A	A	A	A	A	A	A	A	A	A	A	A	A	A	A	A		
RM1386	5	20.06	A	A	A	A	A	A	A	A	A	A	A	A	A	A	H	A	A	A	A	A	A	A	A	A	A	A	A	A	A	A	A	A	A	A	A		1/1 (red)
RM3476	5	23.91	A	A	A	A	A	A	A	A	A	A	A	A	A	A	-	H	A	A	A	A	A	A	A	A	A	A	H	A	A	A	A	A	A	A	A	1/2 (red)	
RM5784	5	27.86	A	A	H	A	A	A	A	A	A	A	A	A	A	A	A	-	A	A	A	A	A	A	A	A	A	A	A	A	A	A	A	A	A	A	A		
RM5907	5	29.61	A	A	H	A	A	A	A	A	A	A	A	A	A	A	A	-	A	A	A	A	A	A	A	A	A	A	A	A	A	A	A	A	A	A	A		
RM8107	6	0.48	A	A	A	A	A	A	A	A	A	A	A	A	A	A	A	A	H	A	A	A	A	A	A	A	A	A	A	A	A	A	A	A	A	A	A		
RM3805	6	2.85	A	A	A	A	A	A	A	A	A	A	A	A	A	A	A	A	H	A	A	A	A	A	A	A	A	A	A	A	A	A	A	A	A	A	A		
RM3431	6	8.75	A	A	A	A	A	A	A	A	A	A	A	A	A	A	A	-	H	A	A	A	A	A	A	A	A	A	A	A	A	A	A	A	A	A	A	1/1 (blue)	
RM19890	6	11.95	A	A	A	A	A	A	A	A	A	A	A	A	A	A	A	A	H	A	A	A	A	A	A	A	A	A	A	A	A	A	A	A	A	A	A		1/1 (blue)
RM20045	6	16.05	A	A	A	A	A	A	A	A	A	A	A	A	-	A	A	A	H	A	A	A	A	A	A	A	A	A	A	A	A	A	A	A	A	A	A		
RM7193	6	20.26	A	A	A	A	A	A	A	A	A	A	A	A	H	A	A	A	H	H	A	A	A	A	A	A	A	A	A	A	A	A	A	A	A	A	A		
RM1340	6	23.34	A	A	A	A	A	A	A	A	A	A	A	A	H	A	A	A	H	H	A	A	A	A	A	A	A	A	A	A	A	A	A	A	A	A	A		
RM3567	6	26.00	A	A	A	A	A	A	A	A	A	A	A	A	-	A	A	A	-	H	A	A	A	A	A	A	A	A	A	A	A	A	A	A	A	A	A	1/1 (red)	1/1 (red)
RM3765	6	29.72	A	A	A	A	A	A	A	A	A	A	A	A	A	A	A	A	A	H	A	A	A	A	A	A	A	A	A	A	A	A	A	A	A	A	A		
RM1031	6	31.09	A	A	A	A	A	A	A	A	A	A	A	A	A	A	A	A	A	H	A	A	A	A	A	A	A	A	A	A	A	A	A	A	A	A	A		
RM4584	7	0.50	A	A	A	A	A	A	H	A	A	A	A	A	A	A	A	A	A	A	H	A	A	A	A	A	A	A	A	A	A	A	A	A	A	A	A		
RM1353	7	3.31	A	A	A	A	A	A	A	A	A	A	A	A	A	A	A	A	A	A	H	H	A	A	A	A	A	A	A	A	A	A	A	A	A	A	A		
RM6728	7	5.73	A	A	A	A	A	A	A	A	A	A	A	A	A	A	A	A	A	A	H	H	A	A	A	A	A	A	A	A	A	A	A	A	A	A	A		
RM2256	7	9.15	A	A	A	A	A	A	A	A	A	A	A	A	A	A	A	A	A	A	H	H	A	A	A	A	A	A	A	A	A	A	A	A	A	A	A		1/2 (red)
RM8025	7	13.00	A	A	A	A	A	A	A	A	A	A	A	A	A	A	A	A	A	A	H	H	A	A	A	A	A	A	A	A	A	A	A	A	A	A	A		
RM3795	7	17.19	A	A	A	A	A	A	A	A	A	A	A	A	A	A	A	A	A	A	A	H	H	H	A	A	A	A	A	A	H	A	A	A	A	A	A	1/4 (red)	
RM1048	7	20.17	A	A	A	A	A	A	A	A	A	A	A	A	A	A	A	-	A	A	A	H	H	H	A	A	A	A	A	A	A	A	A	A	A	A	A		
RM5847	7	23.65	A	A	A	A	A	A	A	A	A	A	A	A	A	A	A	A	A	A	A	H	H	H	A	A	A	A	A	A	A	A	A	A	A	A	A		3/3 (red)
RM1364	7	26.78	A	A	A	A	A	A	A	H	A	A	A	A	A	A	A	A	A	A	A	A	H	H	A	A	A	A	A	A	A	A	A	A	A	A	A		
RM7601	7	29.04	A	A	A	A	A	A	A	A	A	A	A	A	A	A	A	A	A	A	A	A	H	H	A	A	A	A	A	A	A	A	A	A	A	A	A	1/2 (red)	
RM1381	8	0.42	A	A	A	A	A	A	A	A	A	A	A	A	A	A	A	A	A	A	A	A	A	A	H	A	A	A	A	A	A	H	A	A	A	A	A		
RM6670	8	3.00	A	A	A	A	A	H	A	A	H	A	A	A	A	A	A	A	A	A	A	A	A	A	H	H	A	A	A	A	A	A	A	A	A	A	A	2/4 (red)	
RM3507	8	6.16	A	A	A	A	A	A	A	A	A	A	A	A	A	A	A	A	A	A	A	A	A	A	A	H	A	A	A	A	A	A	A	A	A	A	A		1/1 (red)
RM6027	8	9.11	A	A	A	A	A	A	A	A	A	A	A	A	A	A	A	A	A	A	A	A	A	A	A	H	A	A	A	A	A	A	A	A	A	A	A		
RM6032	8	11.90	A	A	A	A	A	A	A	A	A	A	A	A	A	A	A	A	A	A	A	A	A	A	A	H	A	A	A	A	A	A	A	A	A	A	A		
RM6990	8	14.36	A	A	A	A	A	A	A	A	A	A	A	A	A	A	A	A	A	A	A	A	A	A	A	H	A	A	A	A	A	A	A	A	A	A	A		
RM3459	8	20.65	A	A	A	A	A	A	A	A	A	A	A	A	A	A	A	A	A	A	A	A	A	A	A	H	H	A	A	A	A	A	A	A	A	A	A		
RM5485	8	24.07	A	A	A	A	A	A	A	A	A	A	A	A	A	A	A	A	A	A	A	A	A	A	A	H	H	A	A	A	A	A	A	A	A	A	A		
RM5493	8	26.15	A	A	A	A	A	A	A	A	A	A	A	A	A	A	A	A	A	A	A	A	A	A	A	-	H	A	A	A	A	A	A	A	A	A	A		
RM4997	8	28.24	A	A	A	A	A	A	A	A	A	A	A	A	A	A	A	A	A	A	A	A	A	A	A	A	H	A	A	A	A	A	A	A	A	A	A		
RM3609	9	1.16	A	A	A	A	A	A	H	A	A	A	A	A	A	A	A	A	A	A	A	A	A	H	A	A	A	A	A	H	A	A	A	A	A	A	A		
RM3502	9	5.03	A	A	A	A	A	A	H	A	A	A	A	A	A	A	A	A	A	A	A	A	A	H	A	A	A	H	A	H	A	A	A	A	A	A	A		
RM5526	9	7.31	A	A	A	A	A	A	H	A	A	A	A	A	A	A	A	A	A	A	A	A	A	H	A	A	A	H	A	H	A	A	A	A	A	A	A		
RM6854	9	14.37	A	A	A	A	A	A	A	A	A	A	A	A	A	A	A	A	A	A	A	A	A	A	A	A	A	A	A	A	A	A	H	A	A	A	A		
RM3165	9	17.81	A	A	A	A	A	A	A	A	A	A	A	A	A	A	A	A	A	A	A	A	A	A	-	A	A	H	A	A	A	A	H	A	-				
RM24650	9	20.14	A	A	A	A	A	A	A	A	A	A	A	A	A	A	A	A	A	A	A	A	A	A	H	A	A	H	A	A	A	A	H	A	H	A	A		2/4 (red)
RM2255	9	22.59	A	A	A	A	A	A	A	A	A	A	A	A	A	A	A	A	A	A	A	A	A	A	H	A	A	H	A	A	A	A	H	A	H	A	A		
RM5095	10	0.05	A	-	A	A	A	A	A	A	A	A	A	H	A	A	A	A	A	A	A	A	A	A	A	A	A	A	H	A	A	A	A	A	A	A	A		
RM25031	10	3.66	A	-	A	A	A	A	A	A	A	A	A	A	A	A	A	A	A	H	A	A	A	A	A	A	A	A	H	A	A	A	A	A	A	A	A		
RM25151	10	7.62	A	-	A	A	A	A	A	A	A	A	A	A	A	A	A	A	A	H	A	A	A	A	A	A	A	A	H	H	A	A	A	A	A	A	A		
RM25261	10	10.93	A	H	A	A	A	A	A	A	A	A	A	A	A	A	A	A	A	H	A	A	A	A	A	A	A	A	H	H	A	A	A	A	A	A	A		
RM5708	10	14.53	A	-	A	A	A	A	A	A	A	A	A	A	A	A	A	A	A	A	A	A	A	A	A	A	A	A	-	H	A	A	A	A	A	A	A		
RM1873	10	17.89	A	A	A	A	A	A	A	A	A	A	A	A	A	A	A	A	A	A	A	A	A	A	A	A	A	A	A	H	A	A	A	A	A	A	A		
RM3773	10	19.97	A	H	A	A	A	H	A	A	A	A	A	A	A	A	A	A	A	A	A	A	A	A	A	A	A	A	A	H	A	A	A	A	A	A	A		
RM25771	10	20.71	A	A	A	A	A	-	A	A	A	A	A	A	A	A	H	A	A	A	A	A	A	A	A	A	A	A	A	A	A	A	A	A	A	A	A	1/1 (blue)	
RM4771	10	23.09	A	A	A	A	A	A	A	A	A	A	A	A	A	A	H	A	A	A	A	A	A	A	A	A	A	A	A	A	A	A	A	A	A	A	A		
RM1761	11	0.31	A	A	A	A	A	A	A	A	A	A	A	A	A	A	A	A	H	A	A	A	H	A	A	A	A	A	A	A	A	A	A	A	A	A	A		
RM5704	11	5.48	A	A	H	A	A	A	A	A	A	A	A	A	A	A	A	A	A	A	A	A	A	H	A	A	A	A	A	A	-	A	A	A	A	A	A		
RM3701	11	8.11	A	A	H	A	A	A	H	A	A	A	A	A	A	A	A	A	A	A	A	H	A	H	H	A	A	A	A	A	H	H	A	A	A	A	A		
RM5857	11	11.86	A	A	-	A	A	A	-	A	A	A	A	A	A	A	A	A	A	A	A	-	A	H	-	A	A	A	A	A	H	H	A	A	A	A	A		
RM5824	11	14.53	A	A	A	A	A	A	A	A	A	A	A	A	A	A	A	A	A	A	A	A	A	H	A	A	A	A	A	A	H	H	A	A	A	A	A		
RM1355	11	17.67	A	A	A	A	A	A	A	A	A	A	A	A	A	A	A	A	A	A	A	A	A	H	A	A	A	A	A	A	-	H	A	A	A	A	A		
RM1341	11	20.14	A	A	A	A	A	A	A	A	A	A	A	A	A	A	A	A	A	A	A	A	A	H	A	A	A	A	A	A	A	H	A	H	A	A	A		
RM4601	11	23.11	A	A	A	A	A	A	A	A	A	A	A	A	A	A	A	A	A	A	A	A	A	-	A	A	A	A	A	A	A	H	A	H	A	A	A	2/2 (blue)	
RM7654	11	27.67	A	A	A	A	A	A	A	A	A	A	A	A	A	A	A	A	A	A	A	A	A	A	H	A	A	A	A	A	A	-	A	H	A	A	A		
RM5926	11	28.96	A	A	A	A	A	A	A	A	A	A	A	A	A	A	A	A	A	A	A	A	A	A	H	A	A	A	A	A	A	A	A	H	A	A	A		
RM27412	12	0.40	A	A	A	A	A	A	A	A	A	A	A	A	A	A	A	A	A	A	A	A	A	A	A	A	A	A	A	A	A	A	A	H	H	A	H		
RM27589	12	3.38	A	A	A	A	A	A	A	A	A	A	A	A	A	A	A	A	A	A	A	A	A	A	A	A	A	A	A	A	A	A	A	A	H	H	A	1/2 (red)	
RM6905	12	6.75	A	A	A	A	A	A	A	A	A	A	A	A	A	A	A	A	A	A	A	A	A	A	A	A	A	A	A	A	A	A	A	A	-	H	A		
RM27920	12	10.14	A	A	A	A	A	A	A	A	A	A	A	A	A	A	A	A	A	A	A	A	A	A	A	A	A	A	A	A	A	A	A	A	A	H	A		
RM28053	12	14.34	A	A	A	A	A	A	A	A	A	A	A	A	A	A	A	A	A	A	A	A	A	A	A	A	A	A	A	A	A	A	A	A	A	H	A		1/1 (blue)
RM28166	12	17.61	A	A	A	A	A	A	A	A	A	A	A	A	A	A	A	A	A	A	A	A	A	A	A	A	A	A	A	A	A	A	A	A	A	H	A		
RM28305	12	19.96	A	A	A	A	A	A	A	A	A	A	A	A	A	A	A	A	A	A	A	A	A	A	A	A	A	A	A	A	A	A	A	A	A	H	H		
RM1986	12	21.25	A	A	A	A	A	A	A	A	A	A	A	A	A	A	A	A	A	A	A	A	A	-	A	A	A	A	A	A	A	A	A	A	A	-	H		
RM5479	12	24.41	A	A	A	A	A	A	A	A	A	A	A	A	A	A	A	A	A	A	A	A	A	H	A	A	A	A	A	A	A	A	A	A	A	A	H		1/2 (blue)
RM2197	12	27.38	A	A	A	A	A	A	A	A	A	A	A	A	A	A	A	A	A	A	A	A	A	H	A	A	A	A	A	A	A	A	A	A	A	A	H		

[a]Y indicates the presence, and N the absence, of a QTL.

[b]A indicates a homozygous Koshihikari allele; H indicates a heterozygous allele.

[c]QTLs with Koshihikari alleles that decrease trait values are shown in red boxes, while those that increase trait values are shown in blue boxes.
The numbers to the right of each box indicate the number of populations in which the QTL was detected, relative to the total number of populations examined.
